# Supplementary material for: Diastereo-divergent synthesis of chiral hindered ethers via a synergistic calcium(II)/gold(I) catalyzed cascade hydration/1,4-addition reaction
Source: Nat Commun. 2024 May 1;15:3683. doi: 10.1038/s41467-024-47951-9 (PMC11063041; doi:10.1038/s41467-024-47951-9)
Supplement: Supplementary file 1 — Supplementary Information [file 41467_2024_47951_MOESM1_ESM.pdf]

Supplementary Information

Diastereo-divergent Synthesis of Chiral Hindered Ether by Calcium(II)/Gold(I) Catalyzed Cascade Hydration/1, 4-addition

Xiangfeng Lin,[1,2] Xia Mu,[4] Hongqiang Cui,[3,4] Qian Li,[1,3] Zhaochi Feng,[1] Yan Liu,*[1] Guohui Li,*[3] Can Li*[1]

[1]State Key Laboratory of Catalysis, Dalian Institute of Chemical Physics, Chinese Academy of Sciences, Dalian 116023, P. R. China

[2]Key Laboratory of Biopesticide and Chemical Biology (Ministry of Education), College of Plant Protection, Fujian Agriculture and Forestry University, Fuzhou 350002, P. R. China

[3]University of Chinese Academy of Sciences, Beijing 100039, P. R. China

[4]State key Laboratory of Molecular Reaction Dynamics, Dalian Institute of Chemical Physics, Chinese Academy of Science, Dalian 116023, P. R. China

*e-mail: yanliu503@dicp.ac.cn, ghli@dicp.ac.cn, canli@dicp.ac.cn

Contents

1. General information …………………………………………………………………………….S2
2. Experimental procedure …………………………………………………………………………...S3
3. Optimization of reaction……………………………………………………………………..……..S7
4. FTMS result…………………………………………………………………….......................S9
5. Spectral Data of Substrates and Catalysts…………………………………………….………….S10
6. Spectral data of products ………………………………………………………………………....S18
7. X-ray crystallographic data of **6b**………………..……………………………………………..…...S35
8. CD experimental and theory computational spectra of **3a** and **4j**……………………………..…….S52
9. ^{1}H NMR and ^{13}C NMR spectra ……………………………………………………………..…S62
10. HPLC spectra ……..………..………………………………………………………….…S137
11. References………………………...…………………………………………..………………..S178

1. General Information

Reactions were monitored by thin layer chromatography (TLC), and column chromatography purifications were carried out using silica gel. ^1H and ^{13}C spectra were recorded on a 400 MHz spectrometer (101 MHz for ^{13}C) or 700 MHz spectrometer (176 MHz for ^{13}C). The following abbreviations were used to designate chemical shift multiplicities: s = singlet, d = doublet, t = triplet, q = quartert, m = multiplet, br = broad. All first-order splitting patterns were assigned on the basis of the appearance of the multiplet. Splitting patterns that could not be easily interpreted are designated as multiplet (m) or broad (br). Column chromatography was performed on silica gel (300-400 mesh). HPLC analysis was performed on Agilent HPLC 1100 equipped with Daicel chiral AD-H column. High resolution mass spectra for all the new compounds were done by an LTQ-Orbitrap instrument (ESI) (Thermo Fisher Scientific, USA). Catalysts were purchased from J&K Scientific (China) Co., LTD. Substrates **1**, **2**, calcium phosphates and Au(I) complexes were synthesized by following the published procedures.[1-10]

2. Experimental Procedure

General Experimental Procedure of Asymmetric Cascade Reaction

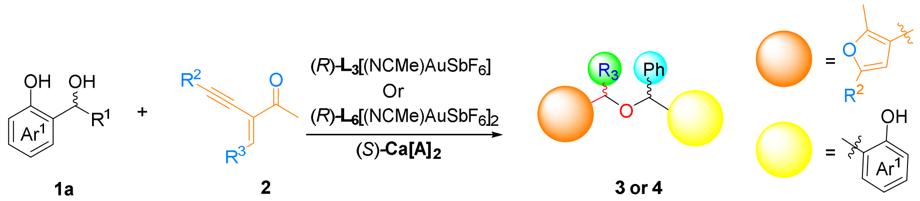

To a 10-mL test-tube were sequentially added (*R*)-**L**$_3$[(NCMe)AuSbF$_6$] (0.0075 mmol, 7.5 mg) or (*R*)-**L**$_6$[(NCMe)AuSbF$_6$]$_2$ (0.00375 mmol, 8.0 mg), **Ca[A]**$_2$ (0.015 mmol, 21.6 mg) and DCE (2.0 mL). The mixture was stirred at -25°C. Substrate **2** (0.16 mmol, 1.05 eq.) and *o*-QM precursor **1** (0.15 mmol) were added in turn to the solution at -25°C. The reaction was monitored by TLC. Upon completion, the residual was purified by silica gel flash chromatography (petroleum ether : ethyl acetate = 20 : 1) to afford the desired product **3** or **4**. The racemic examples were prepared by the catalysis of JohnphosAu(NCMe)SbF$_6$ and Sc(OTf)$_3$ in r.t..

General Experimental Procedure of Scale-up Asymmetric Cascade Reaction

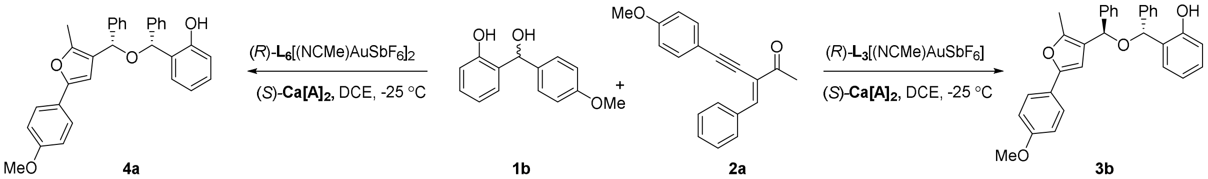

To a 100-mL round-bottom flask were sequentially added (*R*)-**L**$_3$[(NCMe)AuSbF$_6$] (0.0215 mmol, 21.5 mg) or (*R*)-**L**$_6$[(NCMe)AuSbF$_6$]$_2$ (0.01075 mmol, 22.9 mg), **Ca[A]**$_2$ (0.43 mmol, 618.3 mg) and DCE (50.0 mL). The mixture was stirred at -25°C. Substrate **2a** (4.8 mmol, 1.31 g, 1.1 eq.) and *o*-QM precursor **1b** (4.3 mmol, 1.00 g) were added in turn to the solution at -25°C. The reaction was monitored by TLC. Upon completion, The mixture was concentrated under reduced pressure, and the residual was purified by silica gel flash chromatography (petroleum ether : ethyl acetate = 20 : 1) to afford the desired product **3** or **4**. The racemic examples were prepared by the catalysis of JohnphosAu(NCMe)SbF$_6$ and Sc(OTf)$_3$ in r.t..

Experimental Procedure for Synthesis of 6a

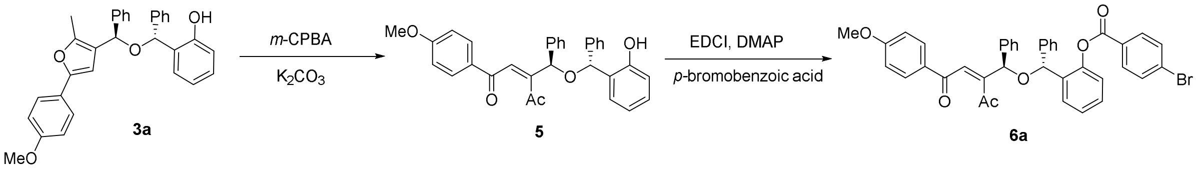

To a 10-mL test-tube were sequentially added **3a** (0.2 mmol, 95.2 mg) (90 % *ee*), *m*-CPBA (0.3 mmol, 190.4 mg)， K$_2$CO$_3$ (0.3 mmol, 41.4 mg) and DCM (5.0 mL) in 0°C. Upon completion (5 h) the residual was purified by silica gel flash chromatography (petroleum ether:ethyl acetate, 5:1) to afford the desired product **5**.

To a 10-mL test-tube were sequentially added **5** (0.1 mmol, 49.2 mg) (90 % *ee*), DMAP (0.12 mmol, 14.7 mg), EDCI (0.12 mmol, 22.9 mg), *p*-bromobenzoic acid (0.12 mmol, 24.2 mg) and DCM (5.0 mL) in r.t.. Upon completion (5 h) the residual was purified by silica gel flash chromatography (petroleum ether : ethyl acetate = 10 : 1) to afford the desired product **6a**.

Experimental Procedure for Synthesis of 7

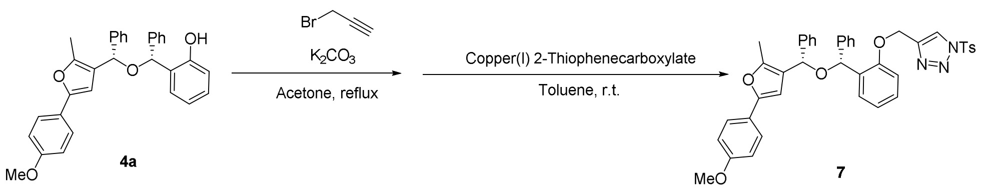

At room temperature, to a solution of **4a** (0.2 mmol, 101.2 mg) in acetone (2 mL) were sequentially added propargyl bromide (0.3 mmol, 35.7 mg, 3.0 eq.) and K_2CO_3 (0.4 mmol, 55.2 mg). The reaction mixture was stirred at 60 °C for 12 h. After cooling to room temperature, the reaction mixture was filtered through a pad of celite. The filtrate was concentrated under reduced pressure, and was used for next reaction directly without further purifying.

A flame dried 5 mL vial, equipped with a stir bar, was charged with the product of propargylation and freshly distilled toluene (1.5 mL). Copper(I) thiophene-2-carboxylate (0.01 mmol, 1.9 mg) and tosyl azide (0.22 mmol, 43.3 mg) was added to the solution at room temperature. After stirring for 12 h, the mixture was concentrated under reduced pressure, and the residue was purified by silica gel column chromatography (petroleum ether : ethyl acetate = 5 : 1) to afford the desired product **7**.

Experimental Procedure for Synthesis of 8

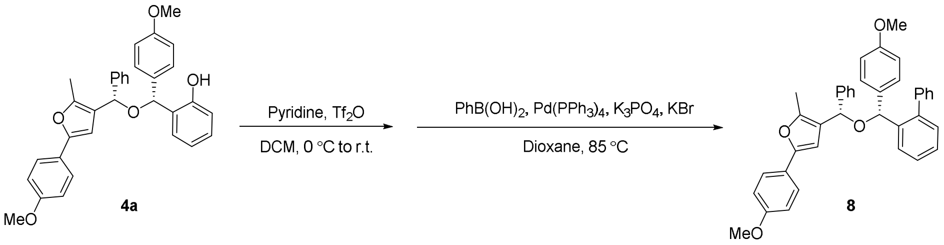

At 0°C, to a stirred solution of **4a** (0.2 mmol, 101.2 mg) in DCM (5 mL) was added pyridine (0.4 mmol, 32 μL) and Tf_2O (0.3 mmol, 50 μL). The mixture was warmed to room temperature and stirred for 5 h. The solvent was removed under reduced pressure, and was used for next reaction directly without further purifying.

To a stirred suspension of $Pd(PPh_3)_4$ (0.01 mmol, 11.6 mg), K_3PO_4 (0.3 mmol, 63.6 mg), KBr (0.22 mmol, 26.1 mg), $PhB(OH)_2$ (0.4 mmol, 48.8 mg) in dioxane (4 mL) was added a solution of triflate in dioxane (4 mL). The reaction was heated at 85 °C with stirring for 24 h. After cooling to room temperature, the reaction mixture was filtered through a pad of celite. The filtrate was concentrated under reduced pressure, and the residue was purified by silica gel column chromatography (petroleum ether : ethyl acetate = 20 : 1) to afford the desired product **8**.

Controlled Experiment Procedure

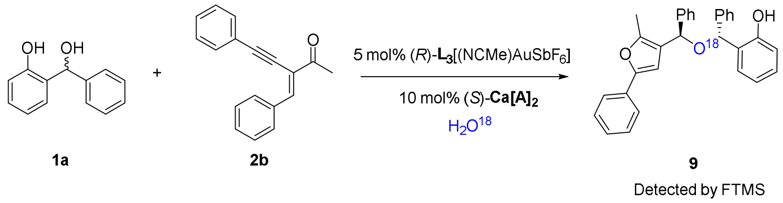

To a 10-mL test-tube were sequentially added (*R*)-**L**$_3$[(NCMe)AuSbF$_6$] (0.0075 mmol, 7.5 mg), Ca[A]$_2$ (0.015 mmol, 21.6 mg) and DCE (2.0 mL). The mixture was stirred at -25°C. Substrate **2b** (0.16 mmol, 1.05 eq., 39.4 mg), H_2O^{18} (0.15 mmol, 3.0 mg) and *o*-QM precursor **1a** (0.15 mmol, 30.0 mg) were added in turn to the solution at -25°C. The reaction mixture was monitored by TLC. Upon completion, the residual was purified by silica gel flash chromatography (petroleum ether : ethyl acetate = 20 : 1) to afford the desired mixture product **3a** and **9**. The compound **9** was detected by FTMS.

Procedure for Synthesis of 1

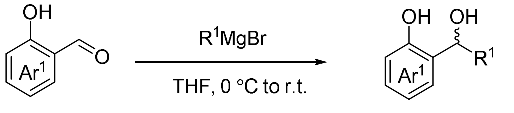

1

To a solution of corresponding salicylaldehyde (10.0 mmol) in THF (20.0 mL) was added a solution of 1M arylmagnesium bromide in THF (30.0 mmol, 3.0 eq., 30 mL) dropwise at 0 °C. The resulting solution was allowed to warm to room temperature and stirred for 2 hours and monitored by TLC. After completion of reaction, the reaction mixture was quenched with saturated aqueous NH_4Cl solution (30.0 ml). It was then extracted with DCM. The combined organic layers were washed with brine, dried over Na_2SO_4, and concentrated under reduced pressure. The residual was purified by silica gel flash chromatography (petroleum ether : ethyl acetate = 5 : 1) to afford desired product **1**.

Procedure for Synthesis of 2

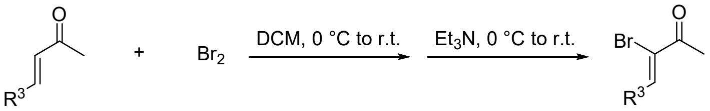

A solution of bromine (21 mmol, 1.05 eq., 1.08 mL) in DCM (20 mL) was added dropwise to a suspension of *α,β*-unsaturated ketone (20 mmol) in DCM (20 mL) at 0 °C. The mixture was stirred for 30 min before removal of the solvent under reduced pressure. Triethylamine (24 mmol, 1.2 eq., 3.34 mL) was added dropwise to the solution and the mixture was stirred overnight at ambient temperature. Upon completion, the reaction mixture was diluted with H_2O. The aqueous phase was extracted with DCM, and the combined organic phase was dried over Na_2SO_4. Then, the mixture was concentrated under reduced pressure. The *α*-bromo-*α,β*-enone was isolated by silica gel flash chromatography (petroleum ether : ethyl acetate = 20 : 1).

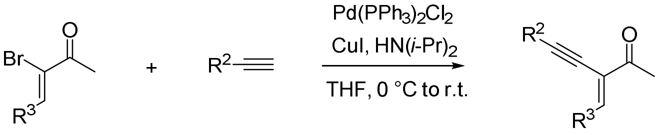

2

The purified *α*-bromo-*α,β*-enone (10 mmol) was dissolved in anhydrous THF (50.0 mL), followed by addition of $Pd(PPh_3)_2Cl_2$ (0.2 mmol, 0.02 eq., 140.3 mg) and CuI (0.4 mmol, 0.04 eq., 75.9 mg). The reaction mixture was cooled to 0 °C and degassed with argon. Alkyne (15 mmol, 1.5 eq.) and diisopropylamine (30 mmol, 3.0 eq., 4.2 mL) was added and the mixture was stirred overnight at ambient temperature. Upon completion, the reaction mixture was diluted with H_2O. The aqueous phase was extracted with DCM, washed with brine and the combined organic phase was dried over $MgSO_4$. Then, the mixture was concentrated under reduced pressure. The residual was purified by silica gel flash chromatography (petroleum ether : ethyl acetate = 20 : 1) to afford desired product **2**.

Procedure for Synthesis of Calcium Phosphates CaA*

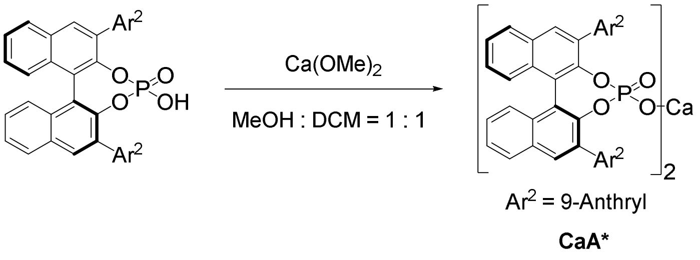

To a 20-mL test-tube were sequentially added (*S*)-3,3'-Bis(anthracenyl-9-yl)-1,1'-binapthyl-2,2'-diyl hydrogenphosphate (1 mmol, 700.7 mg), $Ca(OMe)_2$ (0.5 mmol, 51.0 mg), DCM (10.0 mL) and MeOH (10.0 mL). The mixture was stirred at room temperature for 12 h. Upon completion, the mixture was concentrated under reduced pressure to afford the **CaA***.

Procedure for Synthesis of (*R*)-L$_3$[(NCMe)AuSbF$_6$]

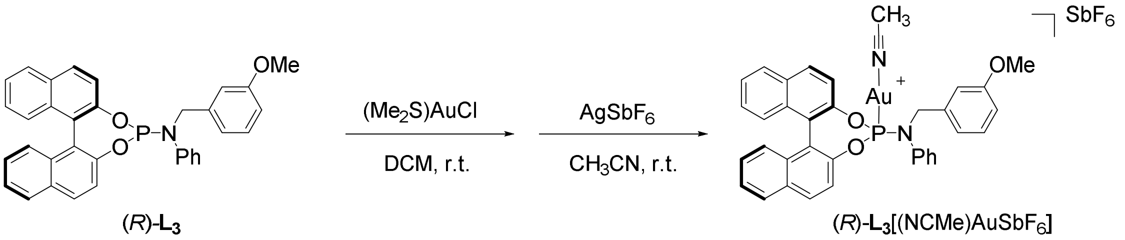

To a 10-mL test-tube were sequentially added (*R*)-**L$_3$** (0.1 mmol, 52.7 mg), $(Me_2S)AuCl$ (0.1 mmol, 29.4 mg) and DCM (1.0 mL). After stirring at room temperature for 2 h, the mixture was concentrated under reduced pressure. The residue and $AgSbF_6$ (0.1 mmol, 34.3 mg) were dissolved in 1.0 mL CH_3CN. The mixture was stirred at room temperature for 12 h. Upon completion, the mixture was filtered through a pad of celite. The filtrate was concentrated under reduced pressure to afford the (*R*)-**L$_3$**[(NCMe)AuSbF$_6$].

Procedure for Synthesis of (*R*)-L$_6$[(NCMe)AuSbF$_6$]$_2$

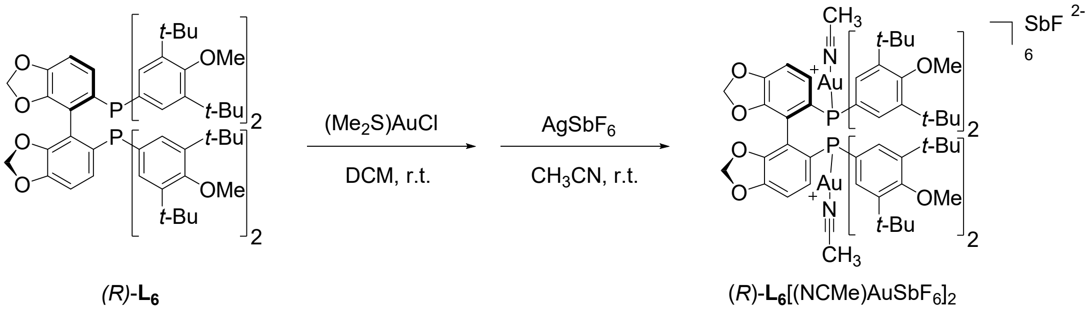

To a 10-mL test-tube were sequentially added (*R*)-**L$_6$** (0.1 mmol, 118.0 mg), $(Me_2S)AuCl$ (0.2 mmol, 58.8 mg) and DCM (1.0 mL). After stirring at room temperature for 2 h, the mixture was concentrated under reduced pressure. The residue and $AgSbF_6$ (0.2 mmol, 68.6 mg) were dissolved in 1.0 mL CH_3CN. The mixture was stirred at room temperature for 12 h. Upon completion, the mixture was filtered through a pad of celite. The filtrate was concentrated under reduced pressure to afford the (*R*)-**L$_6$**[(NCMe)AuSbF$_6$]$_2$.

3. Optimization of Reaction

Supplementary Table 1 Optimization of reaction

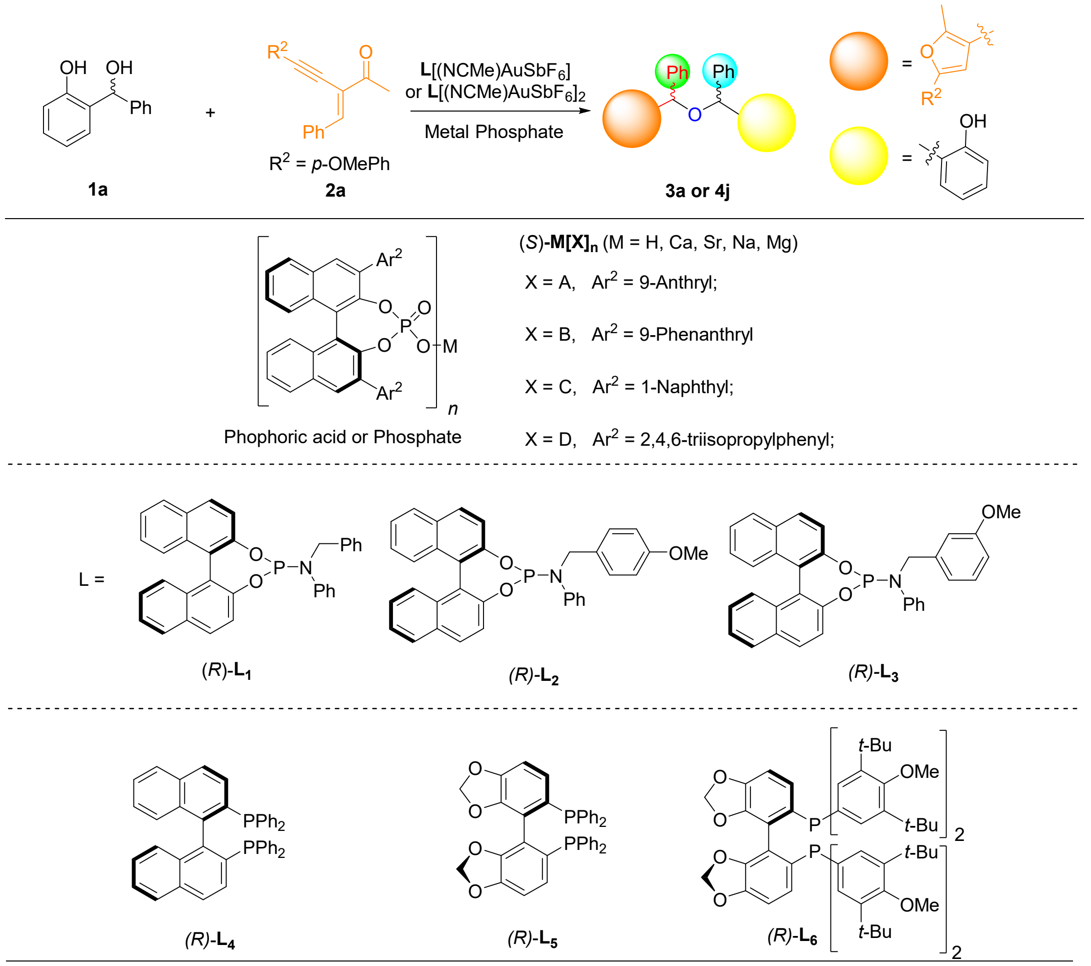

Entry	*(S)*-Phosphate	L	Solvent	Yield /%[a]	*Ee*/% (major)[b]	D.r. (*anti*:*syn*)[c]
1	**H[A]**	(*R*)-**L**$_1$	DCE	83	5	10/1
2	**H[B]**	(*R*)-**L**$_1$	DCE	80	0	11/1
3	**H[C]**	(*R*)-**L**$_1$	DCE	75	0	12/1
4	**H[D]**	(*R*)-**L**$_1$	DCE	60	0	11/1
5[d]	**H[A]***	(*R*)-**L**$_1$	DCE	62	80	10/1
6	**Li[A]**	(*R*)-**L**$_1$	DCE	0	N.D.	N.D.
7	**Na[A]**	(*R*)-**L**$_1$	DCE	0	N.D.	N.D.
8	**Mg[A]$_2$**	(*R*)-**L**$_1$	DCE	0	N.D.	N.D.
9	**Ca[A]$_2$**	(*R*)-**L**$_1$	DCE	72	79	10/1
10	**Sr[A]$_2$**	(*R*)-**L**$_1$	DCE	66	60	10/1
11	**Ca[B]$_2$**	(*R*)-**L**$_1$	DCE	70	42	10/1

12	**Ca[C]$_2$**	(*R*)-**L$_1$**	DCE	73	60	8/1
13	**Ca[D]$_2$**	(*R*)-**L$_1$**	DCE	0	N.D.	N.D.
14[e]	**Ca[A]$_2$**	(*R*)-**L$_1$**	DCE	0	N.D.	N.D.
15[f]	**Ca[A]$_2$**	(*R*)-**L$_1$**	DCE	0	N.D.	N.D.
16	**Ca[A]$_2$**	(*R*)-**L$_2$**	DCE	65	81	15/1
17	**Ca[A]$_2$**	(*R*)-**L$_3$**	DCE	68	88	10/1
18	**Ca[A]$_2$**	(*S*)-**L$_3$**	DCE	66	85	10/1
19	**Ca[A]$_2$**	(*R*)-**L$_3$**	DCM	61	77	10/1
20	**Ca[A]$_2$**	(*R*)-**L$_3$**	$CHCl_3$	30	75	8/1
21[g]	**Ca[A]$_2$**	(*R*)-**L$_3$**	DCE	67	90	10/1
22[h]	**Ca[A]$_2$**	(*R*)-**L$_4$**	DCE	40	N.D.	1/1
23[h]	**Ca[A]$_2$**	(*R*)-**L$_5$**	DCE	64	N.D.	1/1
24[h]	**Ca[A]$_2$**	(*R*)-**L$_6$**	DCE	65	93	1/4
25[h]	**Ca[A]$_2$**	(*S*)-**L$_6$**	DCE	54	-93	1/4

All reactions were carried out on a 0.1 mmol scale with 1 eq precursor of *o*-QMs **1a**, 1.05 eq **2a**, 10 mol % of phosphate and 10 mol % of **L**[(NCMe)AuSbF$_6$] in DCE (1 mL) at -25°C. [a]Isolated yield; [b]Determined by chiral HPLC; [c]Determined by by crude ^{1}H-NMR; [d]H[A]* was purified on silican gel without washing with HCl; [e]5 mol % of **L**AuCl and Ag(OTf); [f]5 mol % of **L**AuCl and AgSbF$_6$; [g]5 mol % of **L**[(NCMe)AuSbF$_6$]; [h]2.5 mol % of **L**[(NCMe)AuSbF$_6$]$_2$.

4. FTMS Results

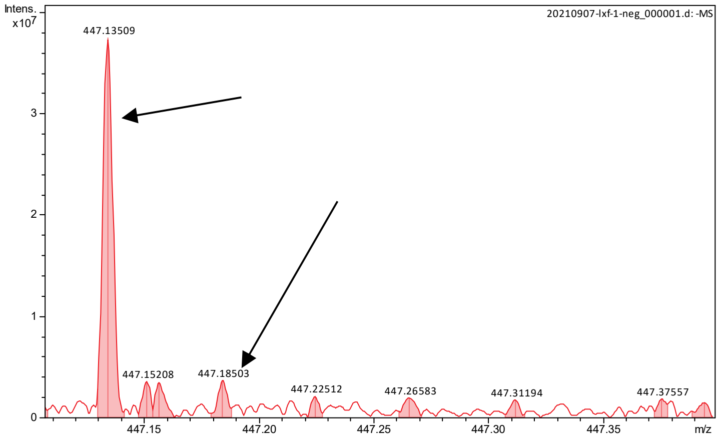

observ.	ppm	relat.	I	theory intensity	S/N
445.18139	1.06		24432354	100	75.8
447.18503	-5.38	15.14%	3698888	6.191	9.6

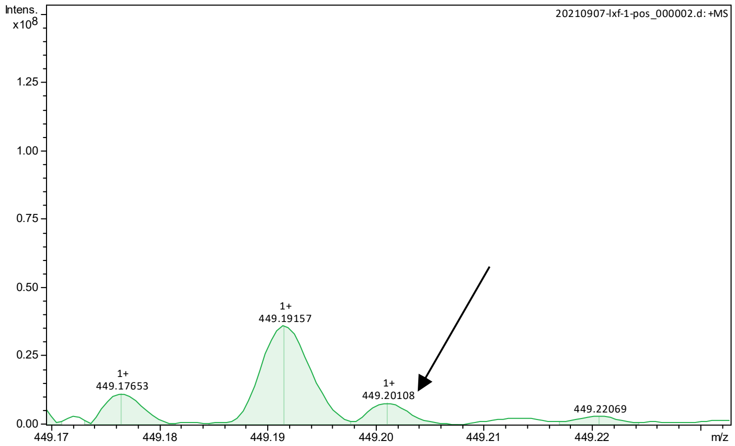

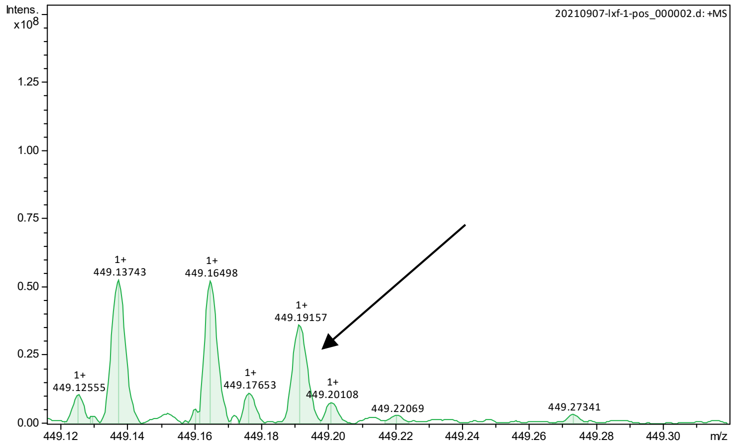

observ.	ppm	relat.	I	theory intensity	S/N
447.19706	3.55		20848630	100	35.7
449.20108	-2.03	38.33%	7990424	6.199	12.8

Supplementary Fig. 1 FTMS results.

5. Spectral Data of Substrates and Catalysts

2-(Hydroxy(phenyl)methyl)phenol 1a

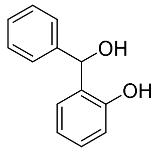

1a

Colourless oil, 1.84 g, 92% yield, ^{1}H NMR (400 MHz, $CDCl_3$) δ 7.94 (s, 1H), 7.53 – 7.30 (m, 5H), 7.25 – 7.18 (m, 1H), 6.96 – 6.80 (m, 3H), 6.03 (s, 1H), 3.01 (s, 1H). ^{13}C NMR (101 MHz, $CDCl_3$) δ 155.2, 141.9, 129.3, 128.8, 128.4, 128.2, 126.9, 126.9, 120.1, 117.2, 76.7.

This is known compound[1]

2-(Hydroxy(4-methoxyphenyl)methyl)phenol 1b

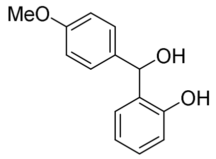

1b

Colourless oil, 2.10 g, 91% yield, ^{1}H NMR (400 MHz, DMSO-d_6) δ 9.34 (s, 1H), 7.35 (dd, J = 7.7, 1.7 Hz, 1H), 7.24 (d, J = 8.4 Hz, 2H), 7.01 (td, J = 7.6, 1.7 Hz, 1H), 6.85 – 6.70 (m, 4H), 5.91 (d, J = 4.3 Hz, 1H), 5.59 (d, J = 4.3 Hz, 1H), 3.70 (s, 3H). ^{13}C NMR (101 MHz, DMSO-d_6) δ 158.3, 154.1, 138.1, 132.5, 128.0, 127.8, 127.1, 119.3, 115.4, 113.6, 68.5, 55.5.

This is known compound[2]

2-((4-Ethylphenyl)(hydroxy)methyl)phenol 1c

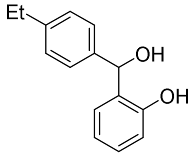

1c

Colourless oil, 1.96 g, 86% yield, ^{1}H NMR (400 MHz, $CDCl_3$) δ 8.10 (s, 1H), 7.32 (d, J = 7.9 Hz, 2H), 7.26 – 7.17 (m, 3H), 6.94 – 6.80 (m, 3H), 5.99 (s, 1H), 3.15 – 2.97 (m, 1H), 2.68 (q, J = 7.6 Hz, 2H), 1.26 (t, J = 7.6 Hz, 3H). ^{13}C NMR (101 MHz, $CDCl_3$) δ 155.5, 144.4, 139.2, 129.2, 128.3, 126.9, 126.7, 119.9, 117.3, 77.1, 28.6, 15.6.

This is known compound[1]

2-(Hydroxy(*p*-tolyl)methyl)phenol 1d

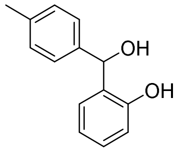

1d

Colourless oil, 1.87 g, 87% yield, ^{1}H NMR (400 MHz, $CDCl_3$) δ 8.01 (s, 1H), 7.30 (d, J = 7.9 Hz, 2H), 7.25 – 7.17 (m, 3H), 6.95 – 6.80 (m, 3H), 6.01 (s, 1H), 2.91 (s, 1H), 2.38 (s, 3H). ^{13}C NMR (101 MHz, $CDCl_3$) δ 155.6, 138.9, 138.2, 129.5, 129.3, 128.3, 126.9, 126.6, 119.9, 117.3, 77.1, 21.2.

This is known compound[1]

2-(Hydroxy(*m*-tolyl)methyl)phenol 1e

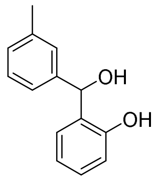

1e

Colourless oil, 1.82 g, 85% yield, ^{1}H NMR (400 MHz, $CDCl_3$) δ 7.98 (s, 1H), 7.31 – 7.12 (m, 5H), 6.96 – 6.79 (m, 3H), 6.01 (s, 1H), 2.90 (s, 1H), 2.37 (s, 3H). ^{13}C NMR (101 MHz, $CDCl_3$) δ 155.6, 141.8, 138.6, 129.3, 129.1, 128.7, 128.3, 127.5, 126.5, 123.9, 119.9, 117.3, 77.3, 21.5.

This is known compound[1]

2-(Hydroxy(*o*-tolyl)methyl)phenol 1f

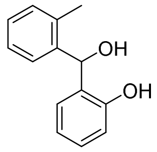

1f

White solid, 1.88 g, 88% yield, ^{1}H NMR (400 MHz, $CDCl_3$) δ 8.10 (s, 1H), 7.42 – 7.35 (m, 1H), 7.32 – 7.19 (m, 4H), 6.95 (dd, J = 8.1, 1.4 Hz, 1H), 6.79 (t, J = 7.5 Hz, 1H), 6.64 (d, J = 7.7 Hz, 1H), 6.28 (s, 1H), 2.99 (s, 1H), 2.36 (s, 3H). ^{13}C NMR (101 MHz, $CDCl_3$) δ 155.9, 139.4, 136.2, 130.9, 129.4, 128.4, 128.0, 127.4, 126.5, 126.0, 120.0, 117.0, 73.5, 19.3.

This is known compound[3]

2-((4-Chlorophenyl)(hydroxy)methyl)phenol 1g

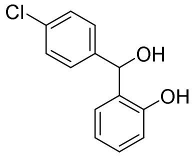

1g

White solid, 2.13 g, 91% yield, ^{1}H NMR (400 MHz, $CDCl_3$) δ 7.72 (s, 1H), 7.43 – 7.25 (m, 4H), 7.22 (td, J = 7.6, 2.1 Hz, 1H), 6.94 – 6.83 (m, 3H), 5.96 (s, 1H), 3.34 (s, 1H). ^{13}C NMR (101 MHz, $CDCl_3$) δ 155.1, 140.3, 133.9, 129.6, 128.8, 128.2, 128.2, 126.6, 120.2, 117.3, 75.9.

This is known compound[1]

2-((3-Chlorophenyl)(hydroxy)methyl)phenol 1h

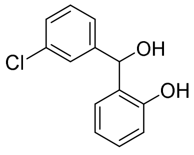

1h

Colourless oil, 2.18 g, 93% yield, ^{1}H NMR (400 MHz, $CDCl_3$) δ 7.92 (s, 1H), 7.39 (s, 1H), 7.35 – 7.16 (m, 4H), 7.00 – 6.81 (m, 3H), 5.88 (s, 1H), 4.04 (s, 1H). ^{13}C NMR (101 MHz, $CDCl_3$) δ 154.7, 144.0, 134.5, 129.9, 129.6, 128.3, 128.1, 126.9, 126.8, 124.9, 120.5, 117.1, 75.4.

This is known compound[4]

2-((4-Fluorophenyl)(hydroxy)methyl)phenol 1i

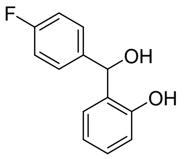

1i

Colourless oil, 1.83 g, 84% yield, ^{1}H NMR (400 MHz, $CDCl_3$) δ 7.86 (s, 1H), 7.36 (dd, *J* = 8.6, 5.4 Hz, 2H), 7.22 (ddd, *J* = 8.5, 6.9, 2.1 Hz, 1H), 7.11 – 7.00 (m, 2H), 6.97 – 6.79 (m, 3H), 5.99 (s, 1H), 3.32 (s, 1H). ^{13}C NMR (101 MHz, $CDCl_3$) δ 162.5 (d, *J* = 246.9 Hz), 155.2, 137.7, 137.7, 129.5, 128.6 (d, *J* = 8.3 Hz), 128.2, 126.7, 120.1, 117.3, 115.6 (d, *J* = 21.5 Hz), 76.1. ^{19}F NMR (376 MHz, $CDCl_3$) δ -114.01.

This is known compound[1]

2-([1,1'-Biphenyl]-4-yl(hydroxy)methyl)phenol 1j

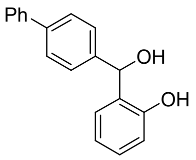

1j

White solid, 2.21 g, 80% yield, ^{1}H NMR (400 MHz, $CDCl_3$) δ 7.96 (s, 1H), 7.65 – 7.58 (m, 4H), 7.52 – 7.43 (m, 4H), 7.39 (t, *J* = 7.4 Hz, 1H), 7.24 (td, *J* = 7.7, 1.7 Hz, 1H), 6.99 – 6.92 (m, 2H), 6.87 (t, *J* = 7.5 Hz, 1H), 6.07 (s, 1H), 3.12 (s, 1H). ^{13}C NMR (101 MHz, $CDCl_3$) δ 155.5, 141.2, 140.8, 140.6, 129.4, 128.9, 128.3, 127.5, 127.3, 127.1, 126.6, 120.01, 117.4, 76.8.

This is known compound[1]

2-(Hydroxy(naphthalen-2-yl)methyl)phenol 1k

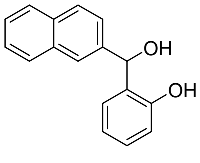

1k

White solid, 2.25 g, 90% yield, ^{1}H NMR (400 MHz, $CDCl_3$) δ 8.19 – 8.07 (m, 2H), 7.97 – 7.85 (m, 2H), 7.58 – 7.42 (m, 4H), 7.23 (td, *J* = 8.1, 7.6, 1.8 Hz, 1H), 7.00 (d, *J* = 8.1 Hz, 1H), 6.79 – 6.63 (m, 3H), 3.23 (s, 1H). ^{13}C NMR (101 MHz, $CDCl_3$) δ 155.8, 136.6, 134.1, 130.9, 129.5, 129.3, 129.0, 128.5, 126.6, 126.0, 125.9, 125.9, 125.5, 124.1, 120.1, 117.1, 74.2.

This is known compound[1]

2-(Hydroxy(naphthalen-1-yl)methyl)phenol 1k

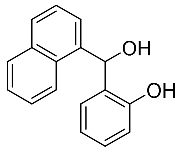

1l

White solid, 2.20 g, 88% yield, ^{1}H NMR (400 MHz, $CDCl_3$) δ 8.02 (s, 1H), 7.93 – 7.80 (m, 4H), 7.60 – 7.48 (m, 3H), 7.22 (td, *J* = 7.7, 1.7 Hz, 1H), 7.04 – 6.76 (m, 3H), 6.15 (s, 1H), 3.25 (s, 1H). ^{13}C NMR (101 MHz, $CDCl_3$) δ 155.5, 139.2, 133.2, 133.1, 129.4, 128.7, 128.4, 128.2, 127.7, 126.6, 126.4, 126.4, 125.6, 124.8, 120.0, 117.3, 77.07.

This is known compound[5]

2-(Hydroxy(phenyl)methyl)-4-methoxyphenol 1m

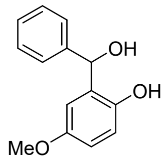

1m

White solid, 1.95 g, 85% yield, ^{1}H NMR (400 MHz, $CDCl_3$) δ 7.57 (s, 1H), 7.43 – 7.30 (m, 5H), 6.81 (d, J = 8.8 Hz, 1H), 6.74 (dd, J = 8.8, 2.9 Hz, 1H), 6.46 (d, J = 2.9 Hz, 1H), 5.92 (s, 1H), 3.69 (s, 3H). ^{13}C NMR (101 MHz, $CDCl_3$) δ 152.9, 149.1, 141.8, 128.7, 128.2, 127.7, 126.8, 117.7, 114.1, 114.0, 76.6, 55.8.

This is known compound[1]

2-(Hydroxy(phenyl)methyl)-4-methylphenol 1n

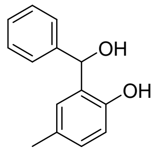

1n

White solid, 2.01 g, 94% yield, ^{1}H NMR (400 MHz, $CDCl_3$) δ 7.79 (s, 1H), 7.47 – 7.31 (m, 5H), 7.01 (d, J = 8.5 Hz, 1H), 6.81 (d, J = 8.2 Hz, 1H), 6.71 (s, 1H), 5.95 (s, 1H), 3.24 (s, 1H), 2.24 (s, 3H). ^{13}C NMR (101 MHz, $CDCl_3$) δ 153.0, 142.0, 129.8, 129.2, 128.7, 128.7, 128.2, 126.8, 126.4, 117.1, 77.0, 20.5.

This is known compound[3]

4-Bromo-2-(hydroxy(phenyl)methyl)phenol 1o

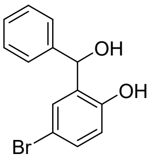

1o

White solid, 1.99 g, 72% yield, ^{1}H NMR (400 MHz, DMSO-d_6) δ 9.67 (s, 1H), 7.36 – 7.18 (m, 6H), 7.07 (d, J = 3.0 Hz, 1H), 6.61 (dd, J = 8.6, 3.0 Hz, 1H), 6.01 (d, J = 4.3 Hz, 1H), 5.85 (d, J = 4.0 Hz, 1H). ^{13}C NMR (101 MHz, DMSO-d_6) δ 157.5, 145.8, 144.2, 133.3, 128.5, 127.5, 127.2, 116.8, 115.9, 110.5, 73.3.

This is known compound[3]

6-(Cyclohexyl(hydroxy)methyl)benzo[d][1,3]dioxol-5-ol 1p

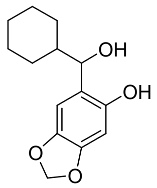

1p

White solid, 1.92 g, 77% yield, ^{1}H NMR (400 MHz, DMSO-d_6) δ 8.94 (s, 1H), 6.72 (s, 1H), 6.36 (s, 1H), 5.86 (d, J = 8.6 Hz, 2H), 4.89 (d, J = 4.8 Hz, 1H), 4.53 (t, J = 5.5 Hz, 1H), 1.87 – 1.53 (m, 4H), 1.51 – 0.89 (m, 7H). ^{13}C NMR (101 MHz, DMSO-d_6) δ 149.0, 146.0, 139.9, 123.4, 107.3, 100.8, 97.6, 71.3, 44.6, 29.5, 28.3, 26.7, 26.4, 26.2.

This is known compound[1]

(*E*)-3-benzylidene-5-(4-methoxyphenyl)pent-4-yn-2-one 2a

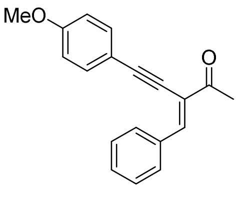

2a

Yellow solid, 1.76 g, 64% yield, ^{1}H NMR (400 MHz, $CDCl_3$) δ 8.12 (d, J = 7.6 Hz, 2H), 7.80 (s, 1H), 7.65 – 7.40 (m, 5H), 6.95 (d, J = 8.4 Hz, 2H), 3.86 (s, 3H), 2.63 (s, 3H). ^{13}C NMR (101 MHz, $CDCl_3$) δ 196.5, 160.2, 142.0, 134.7, 132.9, 130.7, 130.6, 128.6, 120.3, 114.9, 114.3, 99.4, 86.0, 55.0, 28.2.

This is known compound[6]

(*E*)-3-benzylidene-5-phenylpent-4-yn-2-one 2b

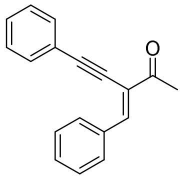

2b

Yellow oil, 1.72 g, 70% yield, ^{1}H NMR (400 MHz, $CDCl_3$) δ 8.18 – 8.09 (m, 2H), 7.85 (s, 1H), 7.62 – 7.55 (dd, J = 6.6, 3.0 Hz, 2H), 7.54 – 7.38 (m, 6H), 2.65 (s, 3H). ^{13}C NMR (101 MHz, $CDCl_3$) δ 196.3, 143.0, 134.5, 131.4, 130.8, 129.0, 128.6, 122.8, 120.0, 99.1, 87.0, 28.2.

This is known compound[6]

(*E*)-3-benzylidene-5-(*p*-tolyl)pent-4-yn-2-one 2c

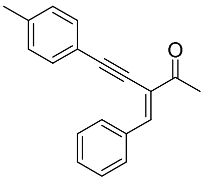

2c

Yellow oil, 1.76 g, 68% yield, ^{1}H NMR (400 MHz, $CDCl_3$) δ 8.13 (d, J = 7.8 Hz, 2H), 7.84 (s, 1H), 7.55 – 7.39 (m, 5H), 7.24 (d, J = 7.8 Hz, 2H), 2.65 (s, 3H), 2.42 (s, 3H). ^{13}C NMR (101 MHz, $CDCl_3$) δ 196.4, 142.5, 139.3, 134.6, 131.3, 130.8, 130.7, 129.4, 128.6, 120.1, 119.8, 99.5, 86.5, 28.2, 21.6.

This is known compound[6]

(*E*)-3-benzylidene-5-(*m*-tolyl)pent-4-yn-2-one 2d

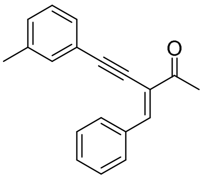

2d

Yellow oil, 1.66 g, 64% yield, ^{1}H NMR (400 MHz, $CDCl_3$) δ 8.13 (d, J = 8.0 Hz, 2H), 7.85 (s, 1H), 7.51 – 7.43 (m, 3H), 7.41 – 7.37 (m, 2H), 7.32 (t, J = 7.8 Hz, 1H), 7.24 (d, J = 7.7 Hz, 1H), 2.65 (s, 3H), 2.41 (s, 3H). ^{13}C NMR (101 MHz, $CDCl_3$) δ 196.4, 142.7, 138.4, 134.6, 131.9, 130.8, 130.8, 129.9, 128.6, 128.5, 128.5, 122.7, 120.0, 99.4, 86.7, 28.2, 21.3.

This is known compound[7]

(*E*)-3-benzylidene-5-(4-bromophenyl)pent-4-yn-2-one 2e

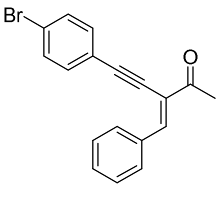

2e

Yellow solid, 2.36 g, 73% yield, ^{1}H NMR (400 MHz, $CDCl_3$) δ 8.08 (dd, J = 6.8, 3.0 Hz, 2H), 7.86 (s, 1H), 7.55 (d, J = 8.3 Hz, 2H), 7.51 – 7.36 (m, 5H), 2.62 (s, 3H). ^{13}C NMR (101 MHz, $CDCl_3$) δ 195.9, 143.6, 134.4, 132.8, 131.9, 130.9, 130.7, 128.7, 123.3, 121.7, 119.8, 97.9, 88.1, 28.2.

This is known compound[7]

(*E*)-3-benzylidene-5-(naphthalen-2-yl)pent-4-yn-2-one 2f

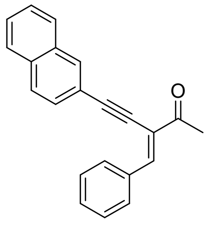

2f

Yellow oil, 2.01 g, 68% yield, ^{1}H NMR (400 MHz, $CDCl_3$) δ 8.18 (d, J = 7.9 Hz, 2H), 8.09 (s, 1H), 7.93 – 7.85 (m, 4H), 7.65 – 7.44 (m, 6H), 2.70 (s, 3H). ^{13}C NMR (101 MHz, $CDCl_3$) δ 196.3, 143.1, 134.6, 133.1, 133.0, 131.6, 130.9, 128.7, 128.4, 127.9, 127.9, 127.2, 126.9, 120.1, 120.0, 99.6, 87.4, 28.3. HRMS (ESI) *m/z* calcd for $C_{22}H_{16}O$, $[M+H]^+$: 297.1279, found: 297.1276.

(*E*)-3-(4-methylbenzylidene)-5-phenylpent-4-yn-2-one 2g

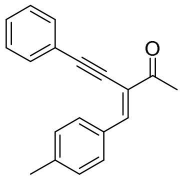

2g

Yellow oil, 1.82 g, 70% yield, ^{1}H NMR (400 MHz, $CDCl_3$) δ 8.04 (d, J = 8.0 Hz, 2H), 7.83 (s, 1H), 7.58 (dd, J = 6.7, 3.0 Hz, 2H), 7.48 – 7.39 (m, 3H), 7.28 (d, J = 8.0 Hz, 2H), 2.64 (s, 3H), 2.43 (s, 3H). ^{13}C NMR (101 MHz, $CDCl_3$) δ 196.4, 143.1, 141.5, 131.8, 131.4, 130.9, 129.4, 128.9, 128.6, 123.0, 119.0, 99.0, 87.3, 28.2, 21.7.

This is known compound[7]

(*E*)-3-(4-bromobenzylidene)-5-phenylpent-4-yn-2-one 2h

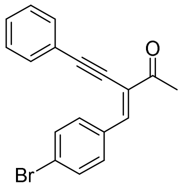

2h

Yellow solid, 2.39 g, 74% yield, ^{1}H NMR (400 MHz, $CDCl_3$) δ 7.96 (d, J = 8.4 Hz, 2H), 7.75 (s, 1H), 7.64 – 7.50 (m, 3H), 7.45 – 7.40 (m, 2H), 2.63 (s, 3H). ^{13}C NMR (101 MHz, $CDCl_3$) δ 195.9, 141.3, 133.4, 132.0, 131.9, 131.4, 129.2, 128.7, 125.1, 122.6, 120.4, 99.9, 86.8, 28.2.

This is known compound[8]

(*E*)-3-(4-bromobenzylidene)-5-(4-methoxyphenyl)pent-4-yn-2-one 2i

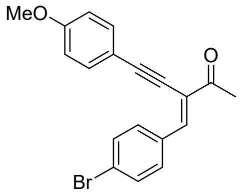

2i

Yellow solid, 2.58 g, 73% yield, ^{1}H NMR (400 MHz, $CDCl_3$) δ 7.96 (d, *J* = 8.2 Hz, 2H), 7.70 (s, 1H), 7.57 (d, *J* = 8.2 Hz, 2H), 7.49 (d, *J* = 8.4 Hz, 2H), 6.94 (d, *J* = 8.5 Hz, 2H), 3.87 (s, 3H), 2.62 (s, 3H). ^{13}C NMR (101 MHz, $CDCl_3$) δ 196.2, 160.3, 140.3, 133.6, 133.0, 131.9, 131.8, 124.8, 120.8, 114.6, 114.4, 100.2, 85.7, 55.4, 28.2. HRMS (ESI) *m/z* calcd for $C_{19}H_{15}BrO_2$, $[M+H]^+$: 355.0334, found: 355.0335.

(*E*)-3-(4-chlorobenzylidene)-5-(4-methoxyphenyl)pent-4-yn-2-one 2i

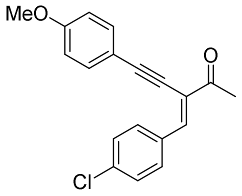

2j

Yellow solid, 2.09 g, 67% yield, ^{1}H NMR (400 MHz, $CDCl_3$) δ 8.04 (d, *J* = 8.5 Hz, 2H), 7.72 (s, 1H), 7.49 (d, *J* = 8.5 Hz, 2H), 7.41 (d, *J* = 8.6 Hz, 2H), 6.95 (d, *J* = 8.5 Hz, 2H), 3.87 (s, 3H), 2.62 (s, 3H). ^{13}C NMR (101 MHz, $CDCl_3$) δ 196.2, 160.3, 140.3, 136.3, 133.2, 133.0, 131.8, 128.9, 120.6, 114.6, 114.4, 100.0, 85.7, 55.4, 28.2. HRMS (ESI) *m/z* calcd for $C_{19}H_{15}ClO_2$, $[M+H]^+$: 311.0839, found: 311.0842.

(*E*)-3-benzylidenenon-4-yn-2-one 2k

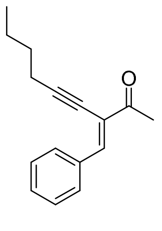

2k

Yellow oil, 1.24 g, 55% yield, ^{1}H NMR (400 MHz, $CDCl_3$) δ 8.06 (dd, *J* = 6.7, 3.0 Hz, 2H), 7.72 (s, 1H), 7.46 – 7.38 (m, 3H), 2.60 – 2.52 (m, 5H), 1.73 – 1.64 (m, 2H), 1.60 – 1.49 (m, 2H), 1.00 (t, *J* = 7.3 Hz, 3H). ^{13}C NMR (101 MHz, $CDCl_3$) δ 197.1, 141.7, 134.6, 130.4, 130.4, 128.4, 120.7, 101.2, 78.1, 30.5, 28.0, 22.2, 19.8, 13.6.

This is known compound[7]

Calcium(II) salt of (R)-3,3'-bis(9-anthracenyl)-1,1'-binaphthyl phosphate CaA*

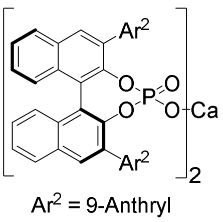

CaA*

Yellow solid, 683.8 mg, 95% yield, ^{1}H NMR (400 MHz, $CDCl_3$) δ 8.33 (s, 4H), 8.03 – 7.87 (m, 12H), 7.87 – 7.32 (m, 32H), 6.88 (s, 4H), 6.78 (s, 4H). ^{13}C NMR (101 MHz, $CDCl_3$) δ 148.1, 148.0, 133.3, 133.0, 132.8, 131.1, 131.0, 130.78,

130.8, 130.8, 130.3, 128.5, 128.3, 128.3, 127.3, 126.7, 126.7, 126.7, 126.0, 125.5, 125.1, 124.9, 123.0, 123.0. ^{31}P NMR (162 MHz, $CDCl_3$) δ 3.26.

This is known compound[9]

(*R*)-[*N*-(3-methoxybenzyl)-N-phenyldinaphtho[2,1-d:1',2'-f][1,3,2]dioxaphosphepin-4-amine](acetonitrile)gold(I) hexafluoroantimonate (*R*)-L_3[(NCMe)AuSbF$_6$]

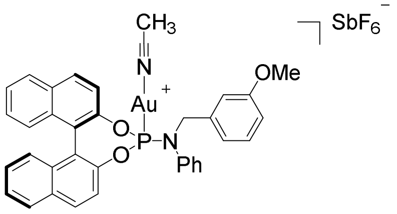

(*R*)-L_3[(NCMe)AuSbF$_6$]

Brown solid, 56.1 mg, 56% yield, ^{1}H NMR (400 MHz, $CDCl_3$) δ 8.23 (d, *J* = 8.9 Hz, 1H), 8.08 – 7.92 (m, 3H), 7.82 (d, *J* = 8.8 Hz, 1H), 7.59 – 7.22 (m, 12H), 7.09 (t, *J* = 7.9 Hz, 1H), 6.73 (dd, *J* = 8.2, 2.5 Hz, 1H), 6.62 (d, *J* = 7.6 Hz, 1H), 6.55 (s, 1H), 4.49 (dd, *J* = 14.8, 8.8 Hz, 1H), 4.27 (dd, *J* = 14.8, 6.9 Hz, 1H), 3.68 (s, 3H), 2.32 (s, 3H). ^{13}C NMR (101 MHz, $CDCl_3$) δ 159.6, 146.8 (d, *J* = 13.4 Hz), 146.0 (d, *J* = 6.6 Hz), 138.8 (d, *J* = 12.1 Hz), 136.5, 136.4, 132.7, 132.41 (d, *J* = 1.4 Hz), 132.33 (d, *J* = 1.7 Hz), 132.1 (d, *J* = 1.4 Hz), 131.82, 131.7 (d, *J* = 1.1 Hz), 130.19, 130.17, 129.74, 129.67, 129.0, 128.8 (d, *J* = 2.4 Hz), 128.7, 127.3, 127.2, 127.1, 126.9, 126.4, 122.5, 121.7, 120.9, 120.8 (d, *J* = 2.8 Hz), 119.8, 114.0, 113.8, 55.31, 55.21, 2.39. ^{31}P NMR (162 MHz, $CDCl_3$) δ 111.84. HRMS (ESI) *m/z* calcd for $C_{36}H_{29}AuF_6N_2O_3PSb$, [M-SbF$_6$]$^+$: 765.1576, found: 765.1568.

(*R*)-[5,5'-bis[di(3,5-di-t-butyl-4-methoxyphenyl)phosphino]-4,4'-bi-1,3-benzodioxole]di(acetonitrile)gold(I) hexafluoroantimonate (*R*)-L_6[(NCMe)AuSbF$_6$]$_2$

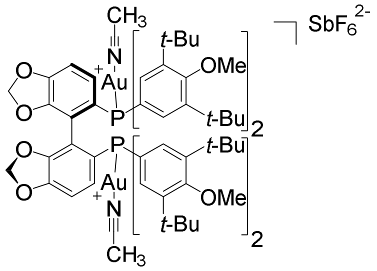

(*R*)-L_6[(NCMe)AuSbF$_6$]$_2$

Grey solid, 131.8 mg, 62% yield, ^{1}H NMR (400 MHz, Chloroform-*d*) δ 7.36 (d, *J* = 8.1 Hz, 2H), 7.28 (s, 2H), 7.20 (s, 2H), 7.05 (dd, *J* = 12.2, 8.2 Hz, 2H), 5.70 (s, 2H), 4.76 (s, 2H), 3.81 (s, 6H), 3.73 (s, 6H), 2.26 (s, 6H), 1.55 – 1.15 (m, 72H). ^{13}C NMR (101 MHz, $CDCl_3$) δ 163.8 (d, *J* = 18.1 Hz), 151.5, 149.1 (d, *J* = 15.2 Hz), 145.7 (d, *J* = 12.9 Hz), 145.6 (d, *J* = 12.9 Hz), 133.44 (d, *J* = 16.8 Hz), 132.75 (d, *J* = 16.3 Hz), 131.41 (d, *J* = 7.3 Hz), 120.6, 120.3, 119.8 (d, *J* = 24.4 Hz), 118.8, 118.1, 117.3 (d, *J* = 23.5 Hz), 110.68 (d, *J* = 12.4 Hz), 102.0, 65.0, 64.9, 36.1, 36.0, 31.7, 2.5. ^{31}P NMR (162 MHz, $CDCl_3$) δ 18.71. HRMS (ESI) *m/z* calcd for $C_{78}H_{106}Au_2F_{12}N_2O_8P_2Sb_2$, [M-SbF$_6$]$^+$: 1189.5698, found: 1189.5683.

6. Spectral Data of Products

2-((*R*)-((*R*)-(5-(4-methoxyphenyl)-2-methylfuran-3-yl)(phenyl)methoxy)(phenyl)methyl)phenol

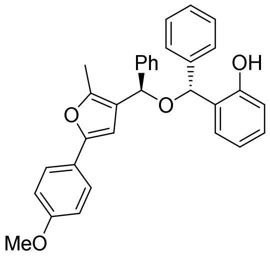

3a

White solid, 47.8 mg, 67% yield, 10:1 d.r., ^{1}H NMR (700 MHz, $CDCl_3$) δ 7.86 (s, 1H), 7.53 (d, *J* = 8.7 Hz, 2H), 7.44 7.28 (m, 10H), 7.23 (t, *J* = 8.2 Hz, 1H), 6.95 (d, *J* = 7.5 Hz, 1H), 6.92 (d, *J* = 7.9 Hz, 1H), 6.87 (m, 3H), 6.45 (s, 1H), 5.63 (s, 1H), 5.44 (s, 1H), 3.81 (s, 3H), 2.16 (s, 3H); ^{13}C NMR (176 MHz, $CDCl_3$) δ 158.9, 155.7, 152.4, 148.8, 140.5, 140.1, 129.6, 129.4, 128.7, 128.5, 128.0, 127.9, 127.2, 126.7, 124.9, 124.3, 123.8, 120.8, 119.9, 117.4, 114.0, 103.3, 80.9, 73.6, 55.3, 11.9.

Enantiomeric excess of the product was determined by chiral stationary phase HPLC analysis using Daicel IA column (*n*-Hexane/*i*-PrOH 97:3 at 0.6 ml/min, λ = 230 nm, t_{minor} = 19.6 min, t_{major} = 24.7 min, 90% *ee*, HRMS (ESI) *m/z* calcd for $C_{32}H_{28}O_4$, $[M+Na]^+$: 499.1880, found: 499.1879.

2-((*R*)-(4-methoxyphenyl)((*R*)-(5-(4-methoxyphenyl)-2-methylfuran-3-yl)(phenyl)methoxy)methyl)phenol

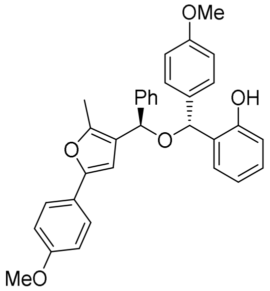

3b

White solid, 52.4 mg, 69% yield, 8:1 d.r., ^{1}H NMR (400 MHz, $CDCl_3$) δ 7.97 (s, 1H), 7.53 (d, *J* = 8.6 Hz, 2H), 7.43 – 7.20 (m, 8H), 7.00 – 6.80 (m, 7H), 6.44 (s, 1H), 5.58 (s, 1H), 5.42 (s, 1H), 3.80 (d, *J* = 13.5 Hz, 6H), 2.16 (s, 3H); ^{13}C NMR (101 MHz, $CDCl_3$) δ 159.4, 158.9, 155.7, 152.4, 148.7, 140.5, 132.2, 129.4, 129.3, 128.7, 128.7, 127.9, 126.7, 124.9, 124.5, 123.8, 121.0, 119.9, 117.4, 114.1, 113.9, 103.3, 80.7, 73.5, 55.3, 55.3, 12.0.

Enantiomeric excess of the product was determined by chiral stationary phase HPLC analysis using Daicel IA column (*n*-Hexane/*i*-PrOH 97:3 at 0.6 ml/min, λ = 230 nm, t_{minor} = 27.0 min, t_{major} = 36.5 min, 90% *ee*, HRMS (ESI) *m/z* calcd for $C_{33}H_{30}O_5$, $[M+H]^+$: 507.2166, found: 507.2167.

2-((*R*)-(4-ethylphenyl)((*R*)-(5-(4-methoxyphenyl)-2-methylfuran-3-yl)(phenyl)methoxy)methyl)phenol

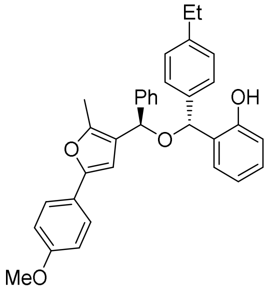

3c

White solid, 49.9 mg, 66% yield, 14:1 d.r., ^{1}H NMR (400 MHz, $CDCl_3$) δ 7.93 (s, 1H), 7.52 (d, *J* = 8.4 Hz, 2H), 7.45 – 7.20 (m, 8H), 7.20 – 7.12 (m, 2H), 7.00 – 6.80 (m, 5H), 6.45 (s, 1H), 5.60 (s, 1H), 5.44 (s, 1H), 3.82 (s, 3H), 2.62 (q, *J* = 7.5 Hz, 2H), 2.16 (s, 3H), 1.21 (t, *J* = 7.6 Hz, 3H); ^{13}C NMR (101 MHz, $CDCl_3$) δ 158.9, 155.7, 152.4,

148.7, 144.1, 140.6, 137.3, 129.5, 129.4, 128.7, 128.1, 127.8, 127.3, 126.7, 124.9, 124.5, 123.8, 120.9, 119.8, 117.4, 114.1, 103.4, 80.9, 73.5, 55.3, 29.7, 28.5, 15.5, 12.0.

Enantiomeric excess of the product was determined by chiral stationary phase HPLC analysis using Daicel IA column (*n*-Hexane/*i*-PrOH 97:3 at 0.6 ml/min, λ = 230 nm, t_{minor} = 19.5 min, t_{major} = 30.5 min, 86% *ee*, HRMS (ESI) *m/z* calcd for $C_{34}H_{32}O_4$, $[M+Na]^+$: 527.2193, found: 527.2181.

2-((*R*)-((*R*)-(5-(4-methoxyphenyl)-2-methylfuran-3-yl)(phenyl)methoxy)(*p*-tolyl)methyl)phenol

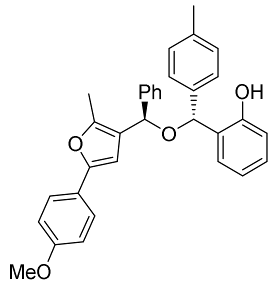

3d

White solid, 50.1 mg, 68% yield, 10:1 d.r., ^1H NMR (400 MHz, $CDCl_3$) δ 7.92 (s, 1H), 7.52 (d, *J* = 8.3 Hz, 2H), 7.45 – 7.05 (m, 10H), 6.96 – 6.75 (m, 5H), 6.44 (s, 1H), 5.60 (s, 1H), 5.43 (s, 1H), 3.81 (s, 3H), 2.32 (s, 3H), 2.16 (s, 3H); ^{13}C NMR (101 MHz, $CDCl_3$) δ 158.9, 155.7, 152.4, 148.7, 140.5, 137.8, 137.12, 129.4, 129.3, 129.3, 128.7, 127.9, 127.2, 126.7, 124.9, 124.6, 123.8, 121.0, 119.8, 117.4, 114.10, 103.4, 80.9, 73.5, 55.3, 21.2, 12.0

Enantiomeric excess of the product was determined by chiral stationary phase HPLC analysis using Daicel IA column (*n*-Hexane/*i*-PrOH 97:3 at 0.6 ml/min, λ = 230 nm, t_{minor} = 20.1 min, t_{major} = 28.7 min, 88% *ee*, HRMS (ESI) *m/z* calcd for $C_{33}H_{30}O_4$, $[M+H]^+$: 491.2217, found: 491.2204.

2-((*R*)-((*R*)-(5-(4-methoxyphenyl)-2-methylfuran-3-yl)(phenyl)methoxy)(*m*-tolyl)methyl)phenol

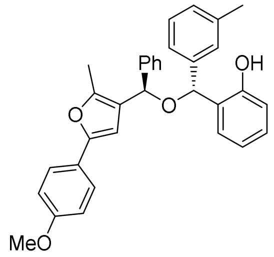

3e

White solid, 48.6 mg, 66% yield, 9:1 d.r., ^1H NMR (700 MHz, $CDCl_3$) δ 7.92 (s, 1H), 7.52 (d, *J* = 8.8 Hz, 2H), 7.43 – 7.39 (m, 2H), 7.38 – 7.34 (m, 2H), 7.29 (t, *J* = 7.3 Hz, 1H), 7.25 – 7.19 (m, 3H), 7.15 (s, 1H), 7.09 (d, *J* = 7.5 Hz, 1H), 6.94 – 6.83 (m, 5H), 6.44 (s, 1H), 5.59 (s, 1H), 5.43 (s, 1H), 3.81 (s, 3H), 2.31 (s, 3H), 2.16 (s, 3H); ^{13}C NMR (176 MHz, $CDCl_3$) δ 158.9, 155.7, 152.4, 148.7, 140.5, 140.1, 138.2, 129.4, 129.3, 128.8, 128.7, 128.4, 127.9, 127.8, 126.73, 124.9, 124.5, 124.3, 123.8, 120.9, 119.8, 117.4, 114.0, 103.4, 81.0, 73.6, 55.3, 21.5, 11.9.

Enantiomeric excess of the product was determined by chiral stationary phase HPLC analysis using Daicel IA column (*n*-Hexane/*i*-PrOH 98:2 at 0.6 ml/min, λ = 230 nm, t_{minor} = 27.7 min, t_{major} = 30.9 min, 90% *ee*, HRMS (ESI) *m/z* calcd for $C_{33}H_{30}O_4$, $[M+H]^+$: 491.2217, found: 491.2214.

2-((*R*)-((*R*)-(5-(4-methoxyphenyl)-2-methylfuran-3-yl)(phenyl)methoxy)(*o*-tolyl)methyl)phenol

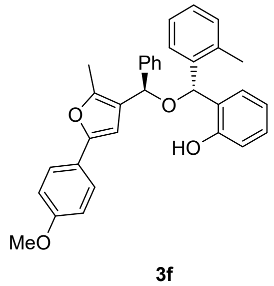

3f

White solid, 44.7 mg, 61% yield, >20:1 d.r., ^{1}H NMR (700 MHz, $CDCl_3$) δ 8.28 (s, 1H), 7.50 (d, *J* = 8.8 Hz, 2H), 7.40 – 7.35 (m, 5H), 7.32 – 7.28 (m, 1H), 7.21 – 7.16 (m, 4H), 6.91 (d, *J* = 7.6 Hz, 1H), 6.87 (d, *J* = 8.8 Hz, 2H), 6.77 – 6.74 (m, 1H), 6.69 – 6.67 (m, 1H), 6.40 (s, 1H), 5.89 (s, 1H), 5.35 (s, 1H), 3.81 (s, 3H), 2.19 (s, 3H), 2.07 (s, 3H); ^{13}C NMR (176 MHz, $CDCl_3$) δ 158.8, 155.9, 152.1, 148.3, 140.2, 137.3, 137.1, 131.0, 129.2, 128.8, 128.7, 128.6, 128.4, 128.1, 127.0, 126.4, 124.9, 124.3, 123.8, 121.2, 119.9, 117.2, 114.0, 103.7, 78.4, 73.8, 55.3, 19.6, 11.9.

Enantiomeric excess of the product was determined by chiral stationary phase HPLC analysis using Daicel IA column (*n*-Hexane/*i*-PrOH 97:3 at 0.6 ml/min, λ = 230 nm, t_{minor} = 20.7 min, t_{major} = 26.4 min, 61% *ee*, [α]20 D = -9.28 (*c* = 0.27, in $CHCl_3$), HRMS (ESI) *m/z* calcd for $C_{33}H_{30}O_4$, $[M+Na]^+$: 513.2036, found: 513.2047.

2-((*R*)-(4-chlorophenyl)((*R*)-(5-(4-methoxyphenyl)-2-methylfuran-3-yl)(phenyl)methoxy)methyl)phenol

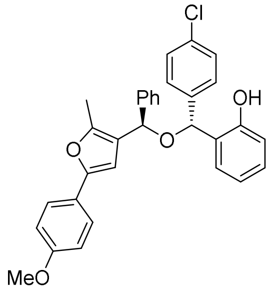

3g

White solid, 51.3 mg, 67% yield, 13:1 d.r., ^{1}H NMR (700 MHz, $CDCl_3$) δ 7.65 (s, 1H), 7.53 (d, *J* = 8.8 Hz, 2H), 7.40 (d, *J* = 7.4 Hz, 2H), 7.36 (t, *J* = 7.7 Hz, 2H), 7.33 – 7.27 (m, 3H), 7.27 – 7.24 (m, 3H), 6.96 (dd, *J* = 7.5, 1.4 Hz, 1H), 6.92 (d, *J* = 7.7 Hz, 1H),6.96 – 6.86 (m, 3H), 6.44 (s, 1H), 5.60 (s, 1H), 5.43 (s, 1H), 5.41 (s, 1H), 3.82 (s, 3H), 2.18 (s, 3H); ^{13}C NMR (176 MHz, $CDCl_3$) δ 159.0, 155.6, 152.6, 148.9, 140.4, 138.7, 133.8, 129.8, 129.3, 128.7, 128.7, 128.5, 128.0, 126.6, 124.9, 123.9, 123.7, 120.6, 120.1, 117.5, 114.1, 103.1, 79.9, 73.7, 55.3, 12.0.

Enantiomeric excess of the product was determined by chiral stationary phase HPLC analysis using Daicel IA column (*n*-Hexane/*i*-PrOH 98:2 at 0.4 ml/min, λ = 230 nm, t_{minor} = 26.2 min, t_{major} = 29.3 min, 87% *ee*, HRMS (ESI) *m/z* calcd for $C_{32}H_{27}ClO_4$, $[M+H]^+$: 511.1671, found: 511.1661.

2-((*R*)-(3-chlorophenyl)((*R*)-(5-(4-methoxyphenyl)-2-methylfuran-3-yl)(phenyl)methoxy)methyl)phenol

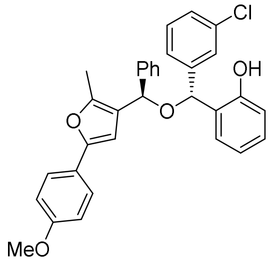

3h

White solid, 50.6 mg, 66% yield, 8:1 d.r., ^{1}H NMR (700 MHz, $CDCl_3$) δ 7.58 (s, 1H), 7.53 (d, *J* = 8.8 Hz, 2H), 7.43 – 7.17 (m, 10H), 6.98 (d, *J* = 7.0 Hz, 1H), 6.95 – 6.81 (m, 4H), 6.45 (s, 1H), 5.60 (s, 1H), 5.44 (s, 1H), 3.81 (d, *J* = 3.1 Hz, 3H), 2.18 (s, 3H); ^{13}C NMR (176 MHz, $CDCl_3$) δ 159.0, 155.6, 152.6, 148.9, 142.4, 140.3, 134.4, 129.9, 129.8, 129.4, 128.7, 128.1, 127.9, 127.2, 126.6, 125.2, 125.0, 123.7, 123.7, 120.5, 120.1, 117.6, 114.1, 103.1, 79.9, 73.8, 55.3, 11.9.

Enantiomeric excess of the product was determined by chiral stationary phase HPLC analysis using Daicel IA column (*n*-Hexane/*i*-PrOH 97:3 at 0.62 ml/min, λ = 230 nm, t_{minor} = 21.6 min, t_{major} = 25.8 min, 89% *ee*, HRMS (ESI) *m/z* calcd for $C_{32}H_{27}ClO_4$, $[M+H]^+$: 511.1671, found: 511.1685.

2-((*R*)-(4-fluorophenyl)((*R*)-(5-(4-methoxyphenyl)-2-methylfuran-3-yl)(phenyl)methoxy)methyl)phenol

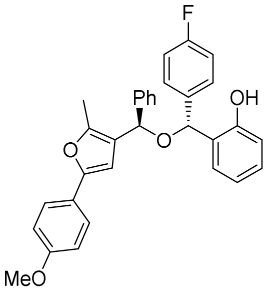

3i

White solid, 43.1 mg, 58% yield, 13:1 d.r., ^{1}H NMR (700 MHz, $CDCl_3$) δ 7.74 (s, 1H), 7.53 (d, *J* = 8.7 Hz, 2H), 7.40 (d, *J* = 7.6 Hz, 2H), 7.36 (t, *J* = 7.6 Hz, 2H), 7.32 – 7.27 (m, 3H), 7.27 – 7.24 (m, 1H), 7.00 (t, *J* = 8.6 Hz, 2H), 6.98 – 6.92 (m, 2H), 6.90 – 6.85 (m, 3H), 6.45 (s, 1H), 5.60 (s, 1H), 5.43 (s, 1H), 3.82 (s, 3H), 2.17 (s, 3H); ^{13}C NMR (176 MHz, $CDCl_3$) δ 162.4 (d, $J_{C\text{-}F}$ = 246.6 Hz), 159.0, 155.7, 152.6, 148.9, 140.4, 136.0, 129.8, 129.3, 129.0 (d, $J_{C\text{-}F}$ = 8.1 Hz), 128.7, 127.9, 126.6, 125.0, 124.1, 123.7, 120.7, 120.0, 117.5, 115.4 (d, $J_{C\text{-}F}$ = 21.4 Hz), 114.1, 103.1, 80.1, 73.6, 55.3, 11.9.

Enantiomeric excess of the product was determined by chiral stationary phase HPLC analysis using Daicel IA column (*n*-Hexane/*i*-PrOH 97:3 at 0.6 ml/min, λ = 230 nm, t_{minor} =22.3 min, t_{major} = 31.3 min, 84% *ee*, HRMS (ESI) *m/z* calcd for $C_{32}H_{27}FO_4$, $[M+Na]^+$: 517.1786, found: 517.1785.

2-((*R*)-[1,1'-biphenyl]-4-yl((*R*)-(5-(4-methoxyphenyl)-2-methylfuran-3-yl)(phenyl)methoxy)methyl)phenol

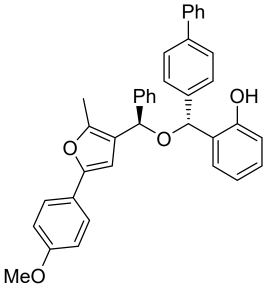

3j

White solid, 58.6 mg, 71% yield, 5:1 d.r., ^{1}H NMR (400 MHz, $CDCl_3$) δ7.87 (s, 1H), 7.61 – 7.50 (m, 6H), 7.49 – 7.28 (m, 10H), 7.00 (d, *J* = 7.3 Hz, 1H), 6.94 (d, *J* = 8.1 Hz, 1H), 6.92 – 6.85 (m, 3H), 6.48 (s, 1H), 5.68 (s, 1H), 5.49 (s, 1H), 3.81 (s, 3H), 2.19 (s, 3H); ^{13}C NMR (101 MHz, $CDCl_3$) δ 158.9, 155.7, 152.5, 148.8, 140.9, 140.7, 140.5, 139.2, 129.6, 129.4, 128.8, 128.7, 127.9, 127.6, 127.4, 127.3, 127.1, 126.7, 125.0, 124.3, 123.8, 120.8, 120.0, 117.5, 114.1, 103.3, 80.7, 73.7, 55.3, 12.0.

Enantiomeric excess of the product was determined by chiral stationary phase HPLC analysis using Daicel IA column (*n*-Hexane/*i*-PrOH 97:3 at 0.6 ml/min, λ = 230 nm, t_{minor} = 25.3 min, t_{major} = 30.7 min, 91% *ee*, HRMS (ESI) *m/z* calcd for $C_{38}H_{32}O_4$, $[M+H]^+$: 553.2373, found: 553.2388.

2-((*R*)-((*R*)-(5-(4-methoxyphenyl)-2-methylfuran-3-yl)(phenyl)methoxy)(naphthalen-2-yl)methyl)phenol

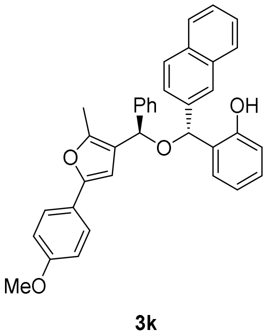

3k

White solid, 55.1 mg, 70% yield, 6:1 d.r., ^{1}H NMR (700 MHz, $CDCl_3$) δ 8.00 (s, 1H), 7.86 – 7.75 (m, 3H), 7.73 (s, 1H), 7.58 – 7.28 (m, 10H), 7.25 – 7.22 (m, 1H), 6.98 (dd, *J* = 7.5, 1.1 Hz, 1H), 6.94 (d, *J* = 8.1 Hz, 1H), 6.91 – 6.82 (m, 3H), 6.47 (s, 1H), 5.84 (s, 1H), 5.80 (s, 1H), 5.49 (s, 1H), 3.81 (s, 3H), 2.15 (s, 3H); ^{13}C NMR (176 MHz, $CDCl_3$) δ 158.9, 155.7, 152.4, 148.7, 140.4, 137.5, 133.1, 129.6, 129.4, 128.7, 128.6, 128.2, 127.9, 127.7, 126.8, 126.2, 126.1, 125.2, 124.9, 124.3, 123.8, 120.9, 119.9, 117.5, 114.0, 103.3, 81.1, 73.8, 55.3, 12.0.

Enantiomeric excess of the product was determined by chiral stationary phase HPLC analysis using Daicel IA column (*n*-Hexane/*i*-PrOH 97:3 at 0.6 ml/min, λ = 230 nm, t_{minor} = 36.1 min, t_{major} = 37.9 min, 90% *ee*,; HRMS (ESI) *m/z* calcd for $C_{36}H_{30}O_4$, $[M+Na]^+$: 549.2036, found: 549.2045.

2-((*R*)-((*R*)-(5-(4-methoxyphenyl)-2-methylfuran-3-yl)(phenyl)methoxy)(naphthalen-1-yl)methyl)phenol

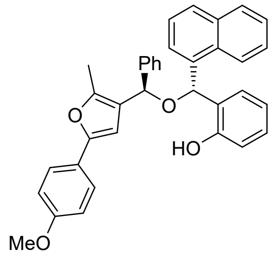

3l

White solid, 56.2 mg, 71% yield, 10:1 d.r., ^{1}H NMR (400 MHz, $CDCl_3$) δ 8.32 (s, 1H), 8.08 (d, *J* = 8.5 Hz, 1H), 7.86 (t, *J* = 8.4 Hz, 2H), 7.56 – 7.26 (m, 11H), 7.21 (d, *J* = 6.8 Hz, 1H), 6.97 (d, *J* = 8.1 Hz, 1H), 6.89 (d, *J* = 8.3 Hz, 2H), 6.81 – 6.72 (m, 2H), 6.45 (s, 1H), 6.38 (s, 1H), 5.41 (s, 1H), 3.82 (s, 3H), 2.00 (s, 3H); ^{13}C NMR (101 MHz, $CDCl_3$) δ 158.9, 156.1, 152.2, 148.9, 140.3, 134.6, 134.1, 131.5, 129.5, 129.4, 129.2, 128.8, 128.7, 128.0, 127.3, 126.8, 126.2, 125.8, 125.3, 124.9, 124.8, 124.0, 123.8, 120.9, 120.0, 117.3, 114.1, 103.9, 79.0, 74.0, 55.3, 11.9.

Enantiomeric excess of the product was determined by chiral stationary phase HPLC analysis using Daicel AD-H column (*n*-Hexane/*i*-PrOH 98.5:1.5 at 0.4 ml/min, λ = 230 nm, t_{major} = 39.4 min, t_{minor} = 43.3 min, 78% *ee*, HRMS (ESI) *m/z* calcd for $C_{36}H_{30}O_4$, $[M+H]^+$: 527.2216, found: 527.2217.

4-methoxy-2-((*R*)-((*R*)-(5-(4-methoxyphenyl)-2-methylfuran-3-yl)(phenyl)methoxy)(phenyl)methyl)phenol

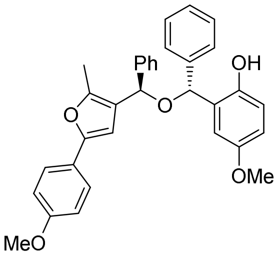

3m

White solid, 51.7 mg, 68% yield, 5:1 d.r., ^{1}H NMR (700 MHz, $CDCl_3$) δ 7.55 – 7.50 (m, 2H), 7.43 – 7.27 (m, 11H), 6.90 – 6.83 (m, 3H), 6.80 (dd, *J* = 8.8, 2.9 Hz, 1H), 6.53 (d, *J* = 2.9 Hz, 1H), 6.45 (s, 1H), 5.57 (s, 1H), 5.45 (s, 1H), 3.81 (s, 3H), 3.72 (s, 3H), 2.18 (s, 3H); ^{13}C NMR (176 MHz, $CDCl_3$) δ 158.9, 153.0, 152.4, 149.5, 148.7, 140.5, 139.9, 128.7, 128.5, 128.0, 127.9, 127.2, 126.7, 125.0, 124.9, 123.8, 120.9, 118.0, 114.7, 114.6, 114.1, 103.3, 80.7, 73.6, 55.7, 55.3, 12.0.

Enantiomeric excess of the product was determined by chiral stationary phase HPLC analysis using Daicel IA column (*n*-Hexane/*i*-PrOH 98:2 at 0.4 ml/min, λ = 230 nm, t_{minor} = 54.6 min, t_{major} = 73.3 min, 90% *ee*, HRMS (ESI) *m/z* calcd for $C_{33}H_{30}O_5$, $[M+H]^+$: 507.2166, found: 507.2163.

2-((*R*)-((*R*)-(5-(4-methoxyphenyl)-2-methylfuran-3-yl)(phenyl)methoxy)(phenyl)methyl)-4-methylphenol

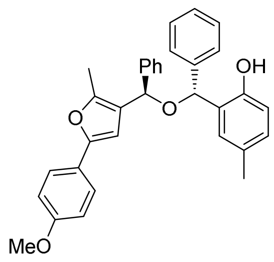

3n

White solid, 50.1 mg, 68% yield, 6:1 d.r., ^{1}H NMR (400 MHz, $CDCl_3$) δ 7.67 (s, 1H), 7.52 (d, J = 8.5 Hz, 2H), 7.45 – 7.26 (m, 11H), 7.03 (d, J = 8.1 Hz, 1H), 6.90 – 6.75 (dd, J = 24.1, 8.3 Hz, 3H), 6.75 (s, 1H), 6.44 (s, 1H), 5.57 (s, 1H), 5.43 (s, 1H), 3.81 (s, 3H), 2.24 (s, 3H), 2.16 (s, 3H); ^{13}C NMR (101 MHz, $CDCl_3$) δ 158.9, 153.4, 152.4, 148.7, 140.6, 140.3, 130.04, 129.8, 129.0, 128.7, 128.6, 128.0, 128.0, 127.3, 126.7, 124.9, 124.0, 123.8, 120.9, 117.2, 114.1, 103.4, 81.0, 73.5, 55.3, 20.5, 11.9.

Enantiomeric excess of the product was determined by chiral stationary phase HPLC analysis using Daicel IA column (*n*-Hexane/*i*-PrOH 97:3 at 0.6 ml/min, λ = 230 nm, t_{minor} = 18.3 min, t_{major} = 25.2 min, 86% *ee*, HRMS (ESI) *m/z* calcd for $C_{33}H_{30}O_4$, $[M+H]^+$: 491.2217, found: 491.2224.

6-((*R*)-cyclohexyl((*R*)-(5-(4-methoxyphenyl)-2-methylfuran-3-yl)(phenyl)methoxy)methyl)benzo[d][1,3]dioxol-5-ol

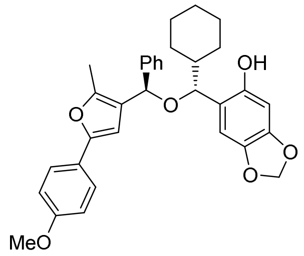

3o

White solid, 29.8 mg, 38% yield, 20:1 d.r., ^{1}H NMR (700 MHz, $CDCl_3$) δ 7.68 (s, 1H), 7.48 (d, J = 8.5 Hz, 2H), 7.41 – 7.35 (m, 4H), 7.34 – 7.29 (m, 1H), 6.85 (d, J = 8.5 Hz, 2H), 6.46 (s, 1H), 6.40 (s, 1H), 6.22 (s, 1H), 5.89 (d, J = 11.9 Hz, 2H), 5.32 (s, 1H), 3.87 (d, J = 8.6 Hz, 1H), 3.80 (s, 3H), 2.27 (d, J = 12.5 Hz, 1H), 2.19 (s, 3H), 2.05 – 1.99 (m, 1H), 1.90 – 1.80 (m, 1H), 1.80 – 1.71 (m, 1H), 1.70 – 1.60 (m, 2H), 1.17 – 1.03 (m, 2H), 0.98 – 0.77 (m, 4H); ^{13}C NMR (176 MHz, $CDCl_3$) δ 158.7, 151.8, 150.9, 147.9, 147.7, 140.4, 140.3, 128.7, 127.9, 127.2, 124.8, 123.9, 122.2, 115.2, 113.94, 109.0, 103.3, 101.0, 99.2, 85.5, 74.1, 55.3, 42.2, 30.2, 29.9, 26.3, 25.9, 25.9, 12.0.

Enantiomeric excess of the product was determined by chiral stationary phase HPLC analysis using Daicel IA column (*n*-Hexane/*i*-PrOH 97:3 at 0.68 ml/min, λ = 230 nm, t_{minor} = 18.5 min, t_{major} = 23.7 min, 74% *ee*, [α]20 D = -23.20 (*c* = 0.6, in $CHCl_3$), HRMS (ESI) *m/z* calcd for $C_{33}H_{34}O_6$, $[M+H]^+$: 527.2434, found: 527.2436.

2-((*R*)-(4-methoxyphenyl)((*S*)-(5-(4-methoxyphenyl)-2-methylfuran-3-yl)(phenyl)methoxy)methyl)phenol

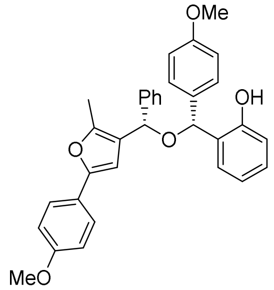

4a

White solid, 59.0 mg, 78% yield, 3:1 d.r., ^{1}H NMR (700 MHz, $CDCl_3$) δ 8.23 (s, 0.75×1H), 7.98 (s, 0.25×1H), 7.52 (d, *J* = 8.7, 2H), 7.42 – 7.38 (m, 2H), 7.36 (t, *J* = 7.6 Hz, 2H), 7.34 – 7.26 (m, 2H), 7.25 – 7.18 (m, 2H), 6.94 – 6.74 (m, 7H), 6.44 (s, 1H), 5.62 (s, 0.75×1H), 5.58 (s, 0.25×1H), 5.42 (s, 0.25×1H), 5.41 (s, 0.75×1H), 3.84 – 3.77 (m, 6H), 2.18 (s, 0.75×3H), 2.16 (s, 0.25×3H); ^{13}C NMR (176 MHz, $CDCl_3$) δ 159.9, 158.9, 155.6, 152.6, 148.9, 141.4, 140.6, 129.7, 129.4, 129.1, 128.7, 127.9, 126.6, 125.1, 125.0, 123.8, 120.6, 119.9, 117.3, 114.1, 113.6, 103.3, 81.1, 73.5, 55.3, 55.3, 12.0.

Enantiomeric excess of the product was determined by chiral stationary phase HPLC analysis using Daicel IA column (*n*-Hexane/*i*-PrOH 97:3 at 0.4 ml/min, λ = 230 nm, t_{minor} = 35.4 min, t_{major} = 38.8 min, 91% *ee*, HRMS (ESI) *m/z* calcd for $C_{33}H_{30}O_5$, $[M+H]^+$: 507.2166, found: 507.2153.

2-((*R*)-(3-methoxyphenyl)((*S*)-(5-(4-methoxyphenyl)-2-methylfuran-3-yl)(phenyl)methoxy)methyl)phenol

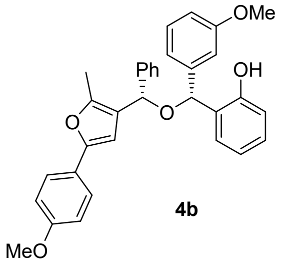

4b

White solid, 59.1 mg, 78% yield, 4:1 d.r., ^{1}H NMR (400 MHz, $CDCl_3$) δ 8.09 (s, 0.8×1H), 7.82 (s, 0.2×1H), 7.52 (d, *J* = 8.4 Hz, 2H), 7.34 – 7.14 (m, 7H), 7.01 – 6.74 (m, 8H), 6.44 (s, 1H), 5.62 (s, 1H), 5.44 (s, 1H), 3.86 – 3.70 (m, 6H), 2.17 (s, 3H); ^{13}C NMR (101 MHz, $CDCl_3$) δ 159.9, 158.9, 155.6, 152.6, 148.9, 141.4, 140.6, 129.7, 129.4, 129.1, 128.7, 127.9, 126.6, 125.2, 125.0, 123.8, 120.6, 120.0, 119.8, 117.3, 114.1, 113.6, 113.2, 103.3, 81.1, 73.6, 55.3, 55.2, 12.0.

Enantiomeric excess of the product was determined by chiral stationary phase HPLC analysis using Daicel IA column (*n*-Hexane/*i*-PrOH 98:2 at 0.4 ml/min, λ = 230 nm, t_{minor} = 46.0 min, t_{major} = 48.5 min, 94% *ee*, HRMS (ESI) *m/z* calcd for $C_{33}H_{30}O_5$, $[M+H]^+$: 507.2166, found: 507.2171.

2-((*R*)-(3-chlorophenyl)((*S*)-(5-(4-methoxyphenyl)-2-methylfuran-3-yl)(phenyl)methoxy)methyl)phenol

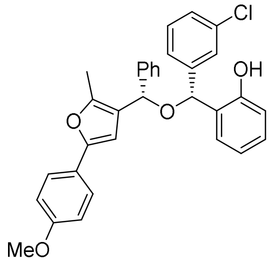

4c

White solid, 61.2 mg, 80% yield, 5:1 d.r., ^{1}H NMR (400 MHz, $CDCl_3$) δ 7.85 (s, 0.83×1H), 7.57 (s, 0.17×1H), 7.52 (d, *J* = 8.5 Hz, 2H), 7.45 – 7.20 (m, 10H), 7.00 – 6.79 (m, 5H), 6.44 (s, 1H), 5.59 (s, 1H), 5.44 (s, 0.17×1H), 5.41 (s, 0.83×1H), 3.81 (s, 3H), 2.17 (s, 3H); ^{13}C NMR (101 MHz, $CDCl_3$) δ 159.0, 155.4, 152.6, 148.8, 142.1, 140.2, 134.6, 130.0, 129.7, 129.1, 128.8, 128.4, 128.1, 127.6, 126.7, 125.5, 125.0, 124.5, 123.7, 120.6, 120.0, 117.5, 114.1, 103.2, 80.5, 73.9, 55.3, 11.9.

Enantiomeric excess of the product was determined by chiral stationary phase HPLC analysis using Daicel IA column (*n*-Hexane/*i*-PrOH 97:3 at 0.6 ml/min, λ = 230 nm, t_{minor} = 23.6 min, t_{major} = 26.1 min, 94% *ee*, HRMS (ESI) *m/z* calcd for $C_{32}H_{27}ClO_4$, $[M+H]^+$: 511.1671, found: 511.1674.

2-((*R*)-((*S*)-(5-(4-methoxyphenyl)-2-methylfuran-3-yl)(phenyl)methoxy)(naphthalen-2-yl)methyl)phenol

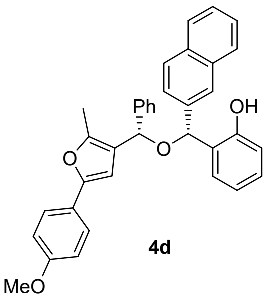

4d

White solid, 69.4 mg, 88% yield, 4:1 d.r., ^{1}H NMR (400 MHz, $CDCl_3$) δ 8.09 (s, 0.8×1H), 7.82 (s, 0.2×1H), 7.52 (d, J = 8.4 Hz, 2H), 7.34 – 7.14 (m, 7H), 7.01 – 6.74 (m, 8H), 6.44 (s, 1H), 5.62 (s, 1H), 5.44 (s, 1H), 3.78 (s, 3H), 2.17 (s, 3H); ^{13}C NMR (176 MHz, $CDCl_3$) δ 158.9, 155.6, 152.6, 148.9, 140.5, 137.1, 133.2, 133.1, 129.3, 129.1, 128.9, 128.7, 128.1, 127.9, 127.7, 126.8, 126.6, 126.4, 126.4, 125.3, 125.1, 125.0, 123.7, 120.5, 119.8, 117.35, 114.0, 103.3, 81.5, 73.5, 55.3, 12.0.

Enantiomeric excess of the product was determined by chiral stationary phase HPLC analysis using Daicel IA column (*n*-Hexane/*i*-PrOH 97:3 at 0.6 ml/min, λ = 230 nm, t_{minor} = 38.4 min, t_{major} = 48.0 min, 92% *ee*, HRMS (ESI) *m/z* calcd for $C_{36}H_{30}O_4$, $[M+H]^+$: 527.2217, found: 527.2217.

2-((*R*)-[1,1'-biphenyl]-4-yl((*S*)-(5-(4-methoxyphenyl)-2-methylfuran-3-yl)(phenyl)methoxy)methyl)phenol

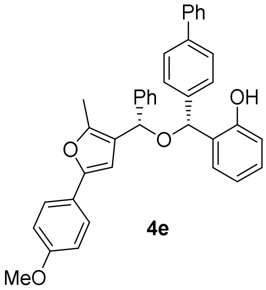

4e

White solid, 58,5 mg, 71% yield, 4:1 d.r.,^{1}H NMR (700 MHz, $CDCl_3$) δ 8.13 (s, 0.8×1H), 7.88 (s, 0.2×1H), 7.60 – 7.51 (m, 6H), 7.47 – 7.27 (m, 10H), 7.26 – 7.24 (t, J = 7.5 Hz, 0.2×1H), 7.23 – 7.18 (t, J = 7.5 Hz, 0.8×1H), 7.01 – 6.78 (m, 5H), 6.47 (s, 1H), 5.70 (s, 0.8×1H), 5.68 (s, 0.2×1H), 5.48 (s, 1H), 3.80 (s, 3H), 2.19 (s, 3H); ^{13}C NMR (176 MHz, $CDCl_3$) δ 158.9, 158.9, 155.7, 155.6, 152.5, 152.5, 148.8, 141.1, 140.9, 140.7, 140.6, 140.5, 139.1, 138.8, 129.61, 129.4, 129.4, 129.1, 128.8, 128.8 128.7, 128.7 128.0, 127.9, 127.6, 127.4, 127.4, 127.4, 127.3, 127.1, 126.7, 126.7, 125.1, 124.9, 124.3, 123.7, 120.8, 120.7, 120.0, 119.8, 117.5, 117.4, 114.1, 114.0, 103.3, 103.3, 81.0, 80.6, 73.7, 73.6, 55.3, 12.0, 12.0.

Enantiomeric excess of the product was determined by chiral stationary phase HPLC analysis using Daicel IA column (*n*-Hexane/*i*-PrOH 97:3 at 0.6 ml/min, λ = 230 nm, t_{minor} = 28.0 min, t_{major} = 32.9 min, 95% *ee*, HRMS (ESI) *m/z* calcd for $C_{38}H_{32}O_4$, $[M+Na]^+$: 575.2193, found: 575.2200.

4-methoxy-2-((*R*)-((*S*)-(5-(4-methoxyphenyl)-2-methylfuran-3-yl)(phenyl)methoxy)(phenyl)methyl)phenol

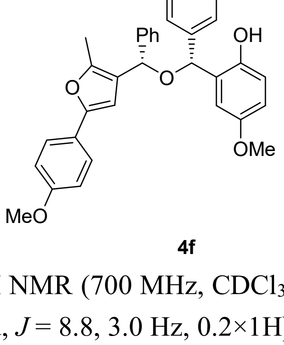

4f

White solid, 64.3 mg, 85% yield, 4:1 d.r., ^{1}H NMR (700 MHz, $CDCl_3$) δ 7.66 (s, 1H), 7.54 – 7.49 (m, 2H), 7.42 – 7.27 (m, 10H), 6.90 – 6.82 (m, 3H), 6.80 (dd, J = 8.8, 3.0 Hz, 0.2×1H), 6.75 (dd, J = 8.8, 3.0 Hz, 0.8×1H), 6.53 (d, J = 3.0 Hz, 0.2×1H), 6.45 (s, 0.2×1H), 6.43 (s, 0.8×1H), 6.38 (d, J = 3.0 Hz, 0.8×1H), 5.59 (s, 0.8×1H), 5.57 (s,

0.2×1H), 5.45 (s, 0.2×1H), 5.42 (s, 0.8×1H), 3.81 (s, 3H), 3.72 (s, 0.2×3H), 3.67 (s, 0.8×3H), 2.18 (s, 0.2×3H), 2.17 (s, 0.8×3H); ^{13}C NMR (176 MHz, $CDCl_3$) δ 158.9, 153.0, 152.8, 152.5, 149.5, 149.3, 148.8, 140.5, 139.9, 139.6, 128.7, 128.7, 128.7, 128.5, 128.3, 128.0, 127.9, 127.9, 127.5, 127.2, 126.7, 126.7, 125.9, 124.9, 124.9, 123.8, 120.9, 120.65, 118.0, 117.8, 114.7, 114.6, 114.2, 114.0, 114.0, 103.3, 81.1, 80.7, 73.6, 73.6, 55.7, 55.7, 55.3, 12.0, 12.0.

Enantiomeric excess of the product was determined by chiral stationary phase HPLC analysis using Daicel IA column (*n*-Hexane/*i*-PrOH 98:2 at 0.4 ml/min, λ = 230 nm, t_{major} = 51.5 min, t_{minor} = 60.9 min, 88% *ee*, HRMS (ESI) *m/z* calcd for $C_{33}H_{30}O_5$, $[M+H]^+$: 507.2166, found: 507.2168.

2-((*R*)-((*S*)-(5-(4-methoxyphenyl)-2-methylfuran-3-yl)(phenyl)methoxy)(phenyl)methyl)-4-methylphenol

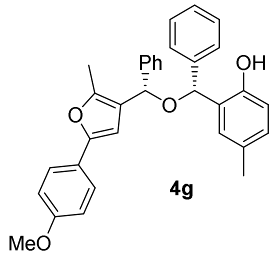

4g

White solid, 48.5 mg, 66% yield, 7:1 d.r., ^{1}H NMR (700 MHz, $CDCl_3$) δ 7.95 (s, 1H), 7.52 (t, *J* = 7.2 Hz, 2H), 7.43 – 7.26 (m, 10H), 6.99 (d, *J* = 7.9 Hz, 1H), 6.87 (d, *J* = 8.6 Hz, 2H), 6.81 (d, *J* = 8.2 Hz, 1H), 6.61 (s, 1H), 6.43 (s, 1H), 5.59 (s, 1H), 5.42 (s, 1H), 3.81 (s, 3H), 2.25 – 2.14 (s, 6H); ^{13}C NMR (176 MHz, $CDCl_3$) δ 158.9, 153.2, 152.4, 148.7, 140.6, 140.0, 129.8, 129.4, 128.9, 128.7, 128.7, 128.2, 127.9, 127.5, 126.6, 124.9, 124.8, 123.8, 120.7, 117.1, 114.0, 103.3, 81.4, 73.5, 55.3, 20.5, 12.0.

Enantiomeric excess of the product was determined by chiral stationary phase HPLC analysis using Daicel IA column (*n*-Hexane/*i*-PrOH 97:3 at 0.6 ml/min, λ = 230 nm, t_{major} = 19.2 min, t_{minor} = 20.4 min, 89% *ee*, HRMS (ESI) *m/z* calcd for $C_{33}H_{30}O_4$, $[M+Na]^+$: 513.2036, found: 513.2046.

4-bromo-2-((*R*)-((*S*)-(5-(4-methoxyphenyl)-2-methylfuran-3-yl)(phenyl)methoxy)(phenyl)methyl)phenol

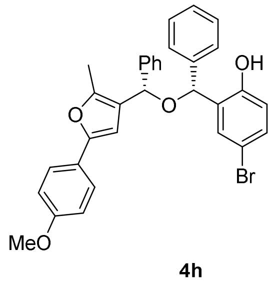

4h

White solid, 63.2 mg, 76% yield, 5:1 d.r., ^{1}H NMR (700 MHz, $CDCl_3$) δ 8.20 (s, 0.83×1H), 7.96 (s, 0.17×1H), 7.53 (d, *J* = 8.7 Hz, 2H), 7.43 – 7.26 (m, 11H), 7.05 (d, *J* = 2.2 Hz, 0.17×1H), 6.91 (d, *J* = 2.2 Hz, 0.83×1H), 6.88 (d, *J* = 8.5 Hz, 2H), 6.80 (d, *J* = 8.7 Hz, 1H), 6.42 (s, 1H), 5.60 (s, 0.83×1H), 5.56 (s, 0.17×1H), 5.40 (s, 1H), 3.81 (s, 3H), 2.17 (s, 3H); ^{13}C NMR (176 MHz, $CDCl_3$) δ 159.0, 154.7, 152.7, 148.9, 140.2, 139.0, 132.1, 131.4, 128.9, 128.8, 128.6, 128.0, 127.6, 127.4, 126.6, 125.0, 123.7, 120.3, 119.2, 114.1, 111.7, 103.1, 80.7, 73.7, 55.3, 12.0.

Enantiomeric excess of the product was determined by chiral stationary phase HPLC analysis using Daicel AD-H column (*n*-Hexane/*i*-PrOH 97:3 at 0.6 ml/min, λ = 230 nm, t_{major} = 27.8 min, t_{minor} = 31.3 min, 89% *ee*, HRMS (ESI) *m/z* calcd for $C_{32}H_{27}BrO_4$, $[M+Na]^+$:577.0985, found: 577.0995.

2-((*R*)-((*R*)-(2-methyl-5-phenylfuran-3-yl)(phenyl)methoxy)(phenyl)methyl)phenol

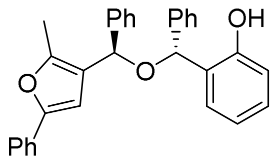

3p

White solid, 40.6 mg, 61% yield, 8:1 d.r., ^{1}H NMR (400 MHz, $CDCl_3$) δ 7.83 (s, 1H), 7.60 (d, *J* = 7.7 Hz, 2H), 7.46 – 7.15 (m, 14H), 6.98 – 6.88 (m, 2H), 6.86 (t, *J* = 7.3 Hz, 1H), 6.59 (s, 1H), 5.63 (s, 1H), 5.46 (s, 1H), 2.18 (s, 3H); ^{13}C NMR (101 MHz, $CDCl_3$) δ 155.7, 152.4, 149.5, 140.4, 140.1, 130.6, 129.6, 129.4, 128.7, 128.6, 128.6, 128.1, 127.9, 127.3, 127.2, 126.7, 124.3, 123.5, 121.1, 119.9, 117.4, 105.0, 80.9, 73.6, 12.0.

Enantiomeric excess of the product was determined by chiral stationary phase HPLC analysis using Daicel IA column (*n*-Hexane/*i*-PrOH 97:3 at 0.6 ml/min, λ = 230 nm, t_{minor} = 14.6 min, t_{major} = 16.9 min, 88% *ee*, HRMS (ESI) *m/z* calcd for $C_{31}H_{26}O_3$, $[M+H]^+$: 447.1955, found: 447.1968.

2-((*R*)-((*R*)-(2-methyl-5-(*p*-tolyl)furan-3-yl)(phenyl)methoxy)(phenyl)methyl)phenol

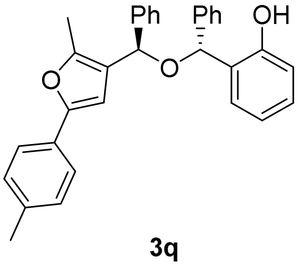

3q

White solid, 43.5 mg, 63% yield, 18:1 d.r.,^{1}H NMR (400 MHz, $CDCl_3$) δ 7.85 (s, 1H), 7.49 (d, *J* = 8.0 Hz, 2H), 7.44 – 7.21 (m, 11H), 7.14 (d, *J* = 7.9 Hz, 2H), 6.97 – 6.87 (m, 2H), 6.86 (t, *J* = 7.4 Hz, 1H), 6.53 (s, 1H), 5.63 (s, 1H), 5.45 (s, 1H), 2.33 (s, 3H), 2.17 (s, 3H); ^{13}C NMR (101 MHz, $CDCl_3$) δ 155.7, 152.6, 149.1, 140.5, 140.1, 137.0, 129.5, 129.4, 129.3, 128.7, 128.5, 128.0, 128.0, 127.9, 127.2, 126.7, 124.3, 123.4, 120.9, 119.9, 117.4, 104.2, 80.9, 73.6, 21.2, 12.0.

Enantiomeric excess of the product was determined by chiral stationary phase HPLC analysis using Daicel IA column (*n*-Hexane/*i*-PrOH 99:1 at 0.3 ml/min, λ = 230 nm, t_{minor} = 29.4 min, t_{major} = 31.6 min, 88% *ee*, HRMS (ESI) *m/z* calcd for $C_{32}H_{28}O_3$, $[M+Na]^+$: 483.1931, found: 483.1929.

2-((*R*)-((*R*)-(2-methyl-5-(*m*-tolyl)furan-3-yl)(phenyl)methoxy)(phenyl)methyl)phenol

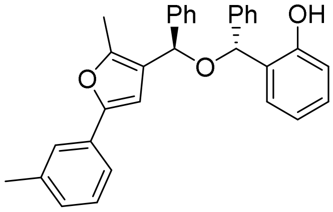

3r

White solid, 35.6 mg, 52% yield, 13:1 d.r., ^{1}H NMR (700 MHz, $CDCl_3$) δ 7.85 (s, 1H), 7.45 – 7.26 (m, 12H), 7.25 – 7.21 (m, 2H), 7.04 (d, J = 7.4 Hz, 1H), 6.96 – 6.91 (m, 2H), 6.86 (t, *J* = 7.4 Hz, 1H), 6.57 (s, 1H), 5.65 (s, 1H), 5.63 (s, 1H), 5.45 (s, 1H), 2.36 (s, 3H), 2.17 (s, 3H); ^{13}C NMR (176 MHz, $CDCl_3$) δ 155.7, 152.6, 149.4, 140.5, 140.1, 138.2, 130.5, 129.6, 129.4, 128.7, 128.6, 128.5, 128.0, 127.9, 127.3, 126.7, 124.3, 124.1, 121.0, 120.7, 119.9, 117.4, 104.8, 80.9, 73.6, 21.5, 12.0.

Enantiomeric excess of the product was determined by chiral stationary phase HPLC analysis using Daicel AD-H column (*n*-Hexane/*i*-PrOH 97:3 at 0.6 ml/min, λ = 230 nm, t_{minor} = 13.8 min, t_{major} = 15.3 min, 93% *ee*, HRMS (ESI) *m/z* calcd for $C_{32}H_{28}O_3$, $[M+H]^+$: 461.2111, found: 461.2120.

2-((*R*)-((*R*)-(5-(4-bromophenyl)-2-methylfuran-3-yl)(phenyl)methoxy)(phenyl)methyl)phenol

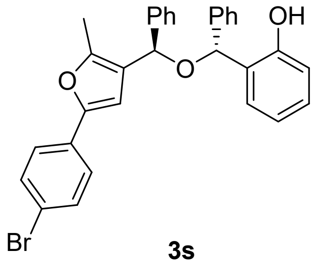

3s

White solid, 31.4 mg, 40% yield, 20:1 d.r., ^1H NMR (400 MHz, $CDCl_3$) δ 7.81 (s, 1H), 7.48 – 7.20 (m, 16H), 6.93 (t, *J* = 8.1 Hz, 2H), 6.85 (t, *J* = 7.3 Hz, 1H), 6.58 (s, 1H), 5.62 (s, 1H), 5.44 (s, 1H), 2.16 (s, 3H); ^{13}C NMR (101 MHz, $CDCl_3$) δ 155.6, 151.3, 149.8, 140.3, 140.0, 131.7, 129.6, 129.3, 128.8, 128.6, 128.1, 128.0, 127.2, 126.7, 125.0, 124.3, 121.4, 120.9, 119.9, 117.4, 105.5, 81.0, 73.5, 12.0.

Enantiomeric excess of the product was determined by chiral stationary phase HPLC analysis using Daicel IA column (*n*-Hexane/*i*-PrOH 97:3 at 0.6 ml/min, λ = 230 nm, t_{minor} = 18.2 min, t_{major} = 19.4 min, 81% *ee*, [α]20 D = -82.70 (*c* = 0.4, in $CHCl_3$), HRMS (ESI) *m/z* calcd for $C_{31}H_{25}BrO_3$, $[M+Na]^+$: 547.0879, found: 547.0894.

2-((*R*)-((*R*)-(2-methyl-5-(naphthalen-2-yl)furan-3-yl)(phenyl)methoxy)(phenyl)methyl)phenol

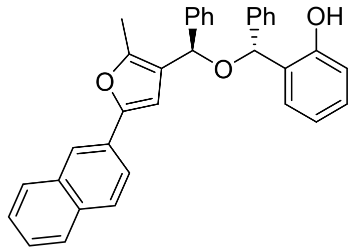

3t

White solid, 40.1 mg, 54% yield, 20:1 d.r., ^1H NMR (400 MHz, $CDCl_3$) δ 8.07 (s, 1H), 7.88 – 7.75 (m, 4H), 7.69 (d, *J* = 8.6 Hz, 1H), 7.51 – 7.28 (m, 13H), 7.00 – 6.83 (m, 3H), 6.71 (s, 1H), 5.66 (s, 1H), 5.48 (s, 1H), 2.22 (s, 3H); ^{13}C NMR (101 MHz, $CDCl_3$) δ 155.7, 152.5, 149.8, 140.4, 140.1, 133.5, 132.6, 129.6, 129.4, 128.7, 128.6, 128.3, 128.1, 128.1, 128.0, 127.7, 127.3, 126.7, 126.4, 125.8, 124.4, 122.1, 121.7, 121.3, 119.9, 117.4, 105.6, 81.0, 73.6, 12.1.

Enantiomeric excess of the product was determined by chiral stationary phase HPLC analysis using Daicel IA column (*n*-Hexane/*i*-PrOH 97:3 at 0.6 ml/min, λ = 230 nm, t_{minor} = 20.5 min, t_{major} = 21.4 min, 88% *ee*, [α]20 D = -33.20 (*c* = 0.2, in $CHCl_3$), HRMS (ESI) *m/z* calcd for $C_{35}H_{28}O_3$, $[M+H]^+$: 497.2111, found: 497.2087.

2-((*R*)-((*R*)-(2-methyl-5-phenylfuran-3-yl)(*p*-tolyl)methoxy)(phenyl)methyl)phenol

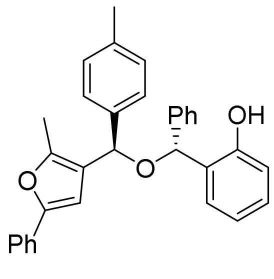

3u

White solid, 36.6 mg, 53% yield, 18:1 d.r., ^{1}H NMR (400 MHz, $CDCl_3$) δ 7.88 (s, 1H), 7.60 (d, *J* = 7.8 Hz, 2H), 7.39 – 7.15 (m, 12H), 7.10 (d, *J* = 6.3 Hz, 1H), 6.98 – 6.88 (m, 2H), 6.85 (t, *J* = 7.3 Hz, 1H), 6.60 (s, 1H), 5.62 (s, 1H), 5.41 (s, 1H), 2.34 (s, 3H), 2.18 (s, 3H); ^{13}C NMR (101 MHz, $CDCl_3$) δ 155.8, 152.4, 149.4, 140.4, 140.1, 138.4, 130.67, 129.5, 129.4, 128.7, 128.6, 128.6, 128.0, 127.3, 127.2, 124.4, 123.7, 123.5, 121.2, 119.9, 117.4, 105.0, 80.9, 73.6, 21.6, 12.0.

Enantiomeric excess of the product was determined by chiral stationary phase HPLC analysis using Daicel AD-H column (*n*-Hexane/*i*-PrOH 98:2 at 0.4 ml/min, λ = 230 nm, t_{minor} = 17.4 min, t_{major} = 20.8 min, 90% *ee*, HRMS (ESI) *m/z* calcd for $C_{32}H_{28}O_3$, $[M+Na]^+$: 483.1931, found: 483.1934.

2-((*R*)-((*R*)-(4-bromophenyl)(2-methyl-5-phenylfuran-3-yl)methoxy)(phenyl)methyl)phenol

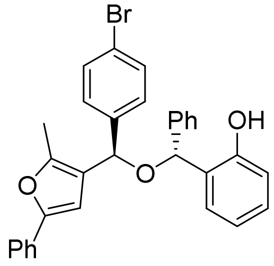

3v

White solid, 33.8 mg, 43% yield, 9:1 d.r., ^{1}H NMR (400 MHz, $CDCl_3$) δ 7.65 – 7.55 (m, 3H), 7.48 (d, *J* = 8.1 Hz, 2H), 7.39 – 7.20 (m, 11H), 6.98 (d, *J* = 7.3 Hz, 1H), 6.94 – 6.85 (m, 2H), 6.54 (s, 1H), 5.61 (s, 1H), 5.41 (s, 1H), 2.18 (s, 3H); ^{13}C NMR (101 MHz, $CDCl_3$) δ 155.6, 152.7, 149.7, 139.9, 139.6, 131.8, 130.5, 129.72, 129.4, 128.7, 128.6, 128.4, 128.1, 127.3, 127.2, 124.0, 123.5, 121.8, 120.6, 120.1, 117.5, 104.6, 80.9, 72.9, 12.0.

Enantiomeric excess of the product was determined by chiral stationary phase HPLC analysis using Daicel ASH column (*n*-Hexane/*i*-PrOH 97:3 at 0.6 ml/min, λ = 230 nm, t_{minor} = 18.2 min, t_{major} = 24.2 min, 75% *ee*, HRMS (ESI) *m/z* calcd for $C_{31}H_{25}BrO_3$, $[M+H]^+$: 525.1060, found: 525.1071.

4-bromo-2-((*R*)-((*R*)-(5-(4-bromophenyl)-2-methylfuran-3-yl)(phenyl)methoxy)(phenyl)methyl)phenol

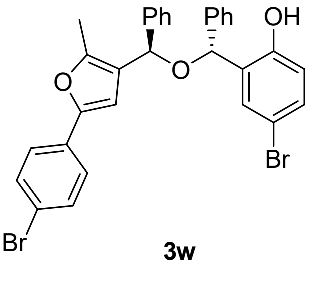

3w

White solid, 36.1 mg, 40% yield, 8:1 d.r., ^{1}H NMR (700 MHz, $CDCl_3$) δ 7.91 (s, 1H), 7.47 – 7.43 (m, 4H), 7.41 – 7.29 (m, 12H), 7.04 (d, *J* = 2.2 Hz, 1H), 6.80 (d, *J* = 8.6 Hz, 1H), 6.55 (s, 1H), 5.54 (s, 1H), 5.40 (s, 1H), 2.17 (s, 3H); ^{13}C NMR (176 MHz, $CDCl_3$) δ 154.7, 151.4, 149.8, 139.8, 139.3, 132.3, 131.7, 131.6, 129.4, 128.9, 128.8, 128.5, 128.2, 127.2, 126.7, 126.6, 125.0, 121.2, 120.9, 119.4, 111.8, 105.4, 80.6, 73.8, 12.0.

Enantiomeric excess of the product was determined by chiral stationary phase HPLC analysis using Daicel IA column (*n*-Hexane/*i*-PrOH 95:5 at 0.9 ml/min, λ = 230 nm, t_{minor} = 13.8 min, t_{major} = 17.9 min, 67% *ee*, HRMS (ESI) *m/z* calcd for $C_{31}H_{24}Br_2O_3$, $[M+H]^+$: 603.0170, found:603.0175.

2-((*R*)-((*S*)-(2-methyl-5-phenylfuran-3-yl)(phenyl)methoxy)(phenyl)methyl)phenol

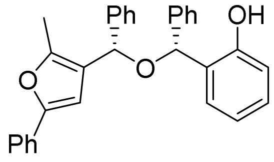

4j

White solid, 38.6 mg, 58% yield, 3:1 d.r., ^{1}H NMR (700 MHz, $CDCl_3$) δ 8.09 (s, 0.75×1H), 7.84 (s, 0.25×1H), 7.59 (d, J = 7.8 Hz, 2H), 7.45 – 7.30 (m, 11H), 7.25 – 7.18 (m, 2H), 6.98 – 6.77 (m, 4H), 6.59 (s, 1H), 5.65 (s, 0.75×1H), 5.63 (s, 0.25×1H), 5.46 (s, 0.25×1H), 5.44 (s, 0.75×1H), 2.18 (s, 3H). ^{13}C NMR (176 MHz, $CDCl_3$) δ 155.7, 155.5, 152.5, 152.4, 149.5, 149.5, 140.4, 140.1, 139.7, 130.6, 130.6, 129.6, 129.4, 129.1, 128.7, 128.7, 128.6, 128.6, 128.6, 128.3, 128.0, 127.9, 127.9, 127.5, 127.2, 127.2, 127.2, 126.7, 126.7, 125.1, 124.3, 123.5, 123.5, 121.1, 120.8, 119.9, 119.8, 117.4, 117.3, 104.9, 81.3, 80.9, 73.5, 73.5, 12.0, 12.0.

Enantiomeric excess of the product was determined by chiral stationary phase HPLC analysis using Daicel IA column (*n*-Hexane/*i*-PrOH 97:3 at 0.6 ml/min, λ = 230 nm, t_{major} = 14.4 min, t_{minor} = 15.2 min, 73% *ee*, HRMS (ESI) *m/z* calcd for $C_{31}H_{26}O_3$, $[M+Na]^+$: 447.1955, found: 447.1948.

2-((*R*)-((*S*)-(5-(4-methoxyphenyl)-2-methylfuran-3-yl)(phenyl)methoxy)(phenyl)methyl)phenol

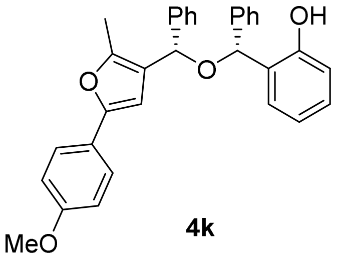

4k

White solid, 46.5 mg, 65% yield, 4:1 d.r., ^{1}H NMR (700 MHz, $CDCl_3$) δ 8.12 (s, 0.8×1H), 7.86 (s, 0.2×1H), 7.52 (d, J = 8.8 Hz, 2H), 7.44 – 7.27 (m, 10H), 7.24 – 7.16 (m, 1H), 6.96 – 6.76 (m, 5H), 6.45 (s, 1H), 5.66 (s, 0.8×1H), 5.63 (s, 0.2×1H), 5.44 (s, 0.2×1H), 5.43 (s, 0.8×1H), 3.81 (s, 3H), 2.16 (s, 3H); ^{13}C NMR (176 MHz, $CDCl_3$) δ 158.9, 155.6, 152.6, 148.8, 140.5, 139.8, 129.4, 129.1, 128.7, 128.7, 128.6, 128.3, 127.9, 127.6, 127.2, 126.7, 126.7, 125.2, 125.0, 125.0, 123.8, 120.6, 119.8, 117.3, 114.1, 114.0, 103.3, 81.3, 73.5, 55.3, 12.0.

Enantiomeric excess of the product was determined by chiral stationary phase HPLC analysis using Daicel IA column (*n*-Hexane/*i*-PrOH 97:3 at 0.6 ml/min, λ = 230 nm, t_{minor} = 21.7 min, t_{major} = 22.4 min, 93% *ee*, HRMS (ESI) *m/z* calcd for $C_{32}H_{28}O_4$, $[M+Na]^+$: 499.1880, found: 499.1870.

2-((*R*)-((*S*)-(4-bromophenyl)(5-(4-methoxyphenyl)-2-methylfuran-3-yl)methoxy)(phenyl)methyl)phenol

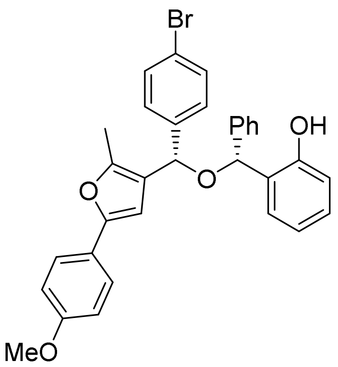

4l

White solid, 34.7 mg, 42% yield, 4:1 d.r., ^{1}H NMR (700 MHz, $CDCl_3$) δ 7.90 (s, 0.8×1H), 7.63 (s, 0.2×1H), 7.52 (d, J = 8.8 Hz, 2H), 7.49 (d, J = 8.4 Hz, 2H), 7.40 – 7.36 (m, 3H), 7.35 – 7.26 (m, 4H), 7.25 – 7.18 (m, 1H), 6.98 (d, J = 7.5 Hz, 0.2×1H), 6.93 – 6.85 (m, 0.2×1H+3H), 6.81 – 6.76 (m, 0.8×2H), 6.40 (s, 0.2×1H), 6.38 (s, 0.8×1H), 5.64 (s, 0.8×1H), 5.61 (s, 0.2×1H), 5.40 (s, 0.2×1H), 5.38 (s, 0.8×1H), 3.82 (s, 3H), 2.16 (s, 3H). ^{13}C NMR (176 MHz, $CDCl_3$) δ 159.0, 155.4, 152.8, 148.9, 139.6, 139.4, 131.8, 129.5, 129.0, 128.8, 128.6, 128.4, 128.3, 127.5, 127.1, 125.1, 125.0, 125.0, 123.6, 121.8, 120.1, 119.9, 117.3, 114., 114., 103.0, 81.2, 72.9, 55.3, 12.0.

Enantiomeric excess of the product was determined by chiral stationary phase HPLC analysis using Daicel AD-H column (*n*-Hexane/*i*-PrOH 97:3 at 0.6 ml/min, λ = 230 nm, t_{minor} = 25.0 min, t_{major} = 26.2 min, 84% *ee*, HRMS (ESI) *m/z* calcd for $C_{32}H_{27}BrO_4$, $[M-H]^+$: 553.1020, found: 553.1028.

2-((*R*)-((*S*)-(4-chlorophenyl)(5-(4-methoxyphenyl)-2-methylfuran-3-yl)methoxy)(phenyl)methyl)phenol

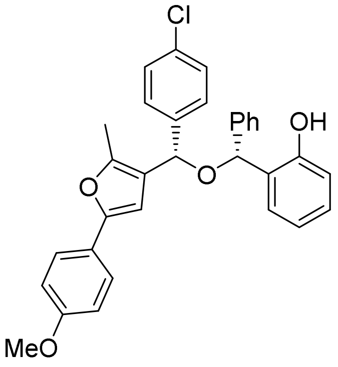

4m

White solid, 32.0 mg, 42% yield, 5:1 d.r., ^{1}H NMR (700 MHz, $CDCl_3$) δ 7.92 (s, 0.83×1H), 7.64 (s, 0.17×1H), 7.52 (d, J = 8.8 Hz, 2H), 7.41 – 7.21 (m, 9H), 7.21 – 7.16 (m, 1H), 6.98 (d, J = 6.4 Hz, 0.17×1H), 6.93 – 6.85 (m, 0.17×1H+3H), 6.81 – 6.76 (m, 0.83×2H), 6.40 (s, 1H), 5.64 (s, 0.83×1H), 5.61 (s, 0.17×1H), 5.42 (s, 0.17×1H), 5.39 (s, 0.83×1H), 3.81 (s, 3H), 2.16 (s, 3H); ^{13}C NMR (176 MHz, $CDCl_3$) δ 159.0, 155.5, 152.8, 148.9, 139.5, 139.1, 133.7, 129.5, 129.1, 128.9, 128.8, 128.6, 128.4, 128.0, 127.6, 127.2, 125.1, 125.0, 125.0, 123.6, 120.2, 119.9, 117.3, 114.10, 114.1, 103.0, 81.2, 72.9, 55.3, 12.0.

Enantiomeric excess of the product was determined by chiral stationary phase HPLC analysis using Daicel IA column (*n*-Hexane/*i*-PrOH 97:3 at 0.6 ml/min, λ = 230 nm, t_{minor} = 24.2 min, t_{major} = 25.2 min, 88% *ee*, HRMS (ESI) *m/z* calcd for $C_{32}H_{27}ClO_4$, $[M+Na]^+$: 533.1490, found: 533.1477.

2-((*R*)-((*S*)-(5-butyl-2-methylfuran-3-yl)(phenyl)methoxy)(4-methoxyphenyl)methyl)phenol

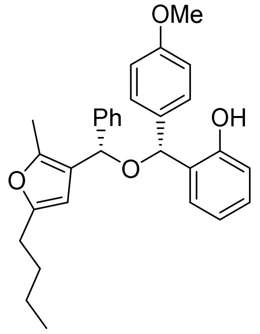

4n

Yellow oil, 39.5 mg, 58% yield, 4:1 d.r., ^{1}H NMR (400 MHz, $CDCl_3$) δ 8.25 (s, 0.8×1H), 8.13 (s, 0.2×1H), 7.42 – 7.20 (m, 9H), 6.96 – 6.86 (m, 3H), 6.82 – 6.73 (m, 1H), 5.94 (s, 0.2×1H), 5.92 (s, 0.8×1H), 5.60 (s, 0.8×1H), 5.56 (s, 0.2×1H), 5.37 (s, 1H), 3.83 (s, 3H), 2.59 – 2.50 (m, 2H), 2.11 (s, 3H), 1.65 – 1.55 (m, 2H), 1.42 – 1.30 (m, 2H), 0.93 (t, J = 7.4 Hz, 3H).; ^{13}C NMR (101 MHz, $CDCl_3$) δ 159.5, 155.7, 155.2, 147.8, 140.9, 131.8, 129.2,

129.1, 129.0, 128.6, 127.7, 126.6, 125.6, 119.7, 118.8, 117.2, 114.0, 104.4, 80.7, 73.4, 55.3, 30.0, 27.8, 22.4, 13.9, 11.8.

Enantiomeric excess of the product was determined by chiral stationary phase HPLC analysis using Daicel IC column (*n*-Hexane/*i*-PrOH 93:7 at 1.0 ml/min, λ = 230 nm, t_{major} = 8.5 min, t_{minor} = 14.9 min, 86% *ee*,; HRMS (ESI) *m/z* calcd for $C_{30}H_{32}O_4$, $[M+H]^+$: 457.2379, found: 457.2373.

2-((*R*)-(((*S*,*Z*)-2-acetyl-4-(4-methoxyphenyl)-4-oxo-1-phenylbut-2-en-1-yl)oxy)(phenyl)methyl)phenyl 4-bromobenzoate

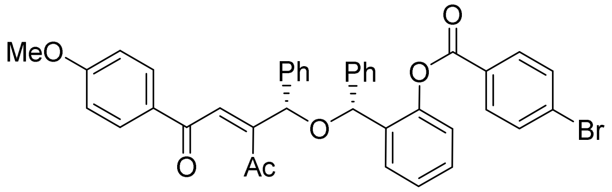

6a

White solid, 36.8 mg, 55% yield, ^{1}H NMR (700 MHz, $CDCl_3$) δ 7.81 (d, *J* = 8.8 Hz, 2H), 7.61 (d, *J* = 8.4 Hz, 2H), 7.52 (d, *J* = 6.5 Hz, 1H), 7.45 (d, *J* = 8.4 Hz, 2H), 7.42 (td, *J* = 7.9, 1.5 Hz, 1H), 7.38 – 7.28 (m, 6H), 7.26 – 7.20 (m, 5H), 7.19 (d, *J* = 8.0 Hz, 1H), 6.96 (d, *J* = 1.5 Hz, 1H), 6.92 (d, *J* = 8.8 Hz, 2H), 5.61 (s, 1H), 5.19 (d, *J* = 1.4 Hz, 1H), 3.88 (s, 3H), 1.98 (s, 3H). ^{13}C NMR (176 MHz, $CDCl_3$) δ 205.2, 187.9, 164.0, 163.9, 159.4, 148.8, 141.0, 136.8, 132.5, 131.8, 131.6, 131.0, 130.0, 129.4, 129.3, 128.9, 128.9, 128.8, 128.3, 128.2, 127.9, 127.5, 126.8, 126.5, 123.2, 120.0, 114.0, 79.3, 75.2, 55.6, 30.6.

Enantiomeric excess of the product was determined by chiral stationary phase HPLC analysis using Daicel IA column (*n*-Hexane/*i*-PrOH 90:10 at 1.0 ml/min, λ = 230 nm, t_{minor} = 19.1 min, t_{major} = 30.9 min, 93% *ee*, [α]20 D = -72.50 (*c* = 0.40, in $CHCl_3$), HRMS (ESI) *m/z* calcd for $C_{39}H_{31}BrO_6$, $[M+H]^+$: 675.1377, found: 675.1357.

4-((2-((*R*)-((*S*)-(5-(4-methoxyphenyl)-2-methylfuran-3-yl)(phenyl)methoxy)(phenyl)methyl)phenoxy)methyl)-1-tosyl-1H-1,2,3-triazole

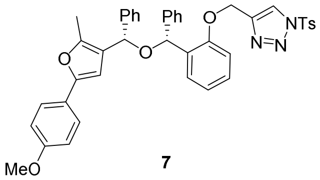

7

White solid, 108.0 mg, 76% yield, ^{1}H NMR (400 MHz, $CDCl_3$) δ 7.91 (d, *J* = 8.2 Hz, 2H), 7.74 (dd, *J* = 7.7, 1.7 Hz, 1H), 7.59 – 7.52 (m, 3H), 7.48 – 7.43 (m, 2H), 7.38 – 7.26 (m, 11H), 7.09 (t, *J* = 7.5 Hz, 1H), 6.95 – 6.87 (m, 3H), 6.42 (s, 1H), 5.86 (s, 1H), 5.41 (s, 1H), 5.16 – 5.06 (m, 2H), 3.84 (s, 3H), 2.44 (s, 3H), 2.15 (s, 3H).; ^{13}C NMR (101 MHz, $CDCl_3$) δ 158.8, 154.9, 151.9, 148.4, 147.4, 144.2, 142.3, 142.0, 133.0, 130.6, 130.5, 128.7, 128.6, 128.4, 128.2, 128.0, 127.5, 127.4, 127.3, 126.7, 124.9, 124.1, 122.3, 122.3, 121.7, 114.1, 111.8, 104.1, 74.3, 72.8, 61.6, 55.3, 21.9, 11.9.

Enantiomeric excess of the product was determined by chiral stationary phase HPLC analysis using Daicel IA column (*n*-Hexane/*i*-PrOH 85:15 at 1.0 ml/min, λ = 230 nm, t_{major} = 3.9 min, t_{minor} = 13.7 min, 93% *ee*, [α]20 D = +30.20 (*c* = 0.20, in $CHCl_3$), HRMS (ESI) *m/z* calcd for $C_{42}H_{37}N_3O_6S$, $[M+H]^+$: 712.2481, found: 712.2478.

3-((*S*)-((*R*)-[1,1'-biphenyl]-2-yl(4-methoxyphenyl)methoxy)(phenyl)methyl)-5-(4-methoxyphenyl)-2-methylfuran

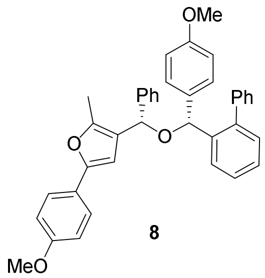

8

White solid, 57.8 mg, 51% yield, ^{1}H NMR (400 MHz, $CDCl_3$) δ 7.90 (d, J = 7.8, 1H), 7.53 – 7.30 (m, 9H), 7.26 – 7.16 (m, 4H), 7.00 – 6.93 (m, 4H), 6.89 (d, J = 8.8 Hz, 2H), 6.76 (d, J = 8.7 Hz, 2H), 6.33 (s, 1H), 5.61 (s, 1H), 5.25 (s, 1H), 3.84 (s, 3H), 3.77 (s, 3H), 2.05 (s, 3H).; ^{13}C NMR (101 MHz, $CDCl_3$) δ 158.7, 151.7, 148.0, 142.1, 141.5, 140.6, 139.2, 134.7, 129.9 129.2, 129.0, 128.2, 127.9, 127.8, 127.4, 127.2, 127.2, 127.0, 126.8, 126.6, 124.8, 124.1, 122.2, 114.0, 113.4, 104.1, 76.1, 72.9, 55.3, 55.2, 12.0.

Enantiomeric excess of the product was determined by chiral stationary phase HPLC analysis using Daicel IA column (*n*-Hexane/*i*-PrOH 95:5 at 1.0 ml/min, λ = 230 nm, t_{major} = 3.6 min, t_{minor} = 18.4 min, 90% *ee*, [α]20 D = +67.60 (c = 0.40, in $CHCl_3$), HRMS (ESI) *m/z* calcd for $C_{39}H_{34}O_4$, $[M+H]^+$: 567.2535, found: 567.2541.

2-((*S*)-(4-methoxyphenyl)((*S*)-(5-(4-methoxyphenyl)-2-methylfuran-3-yl)(phenyl)methoxy)methyl)phenol

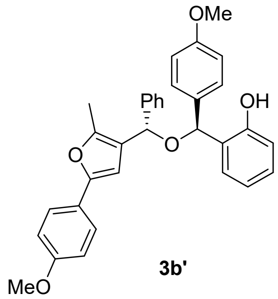

3b'

White solid, 49.4 mg, 65% yield, 8:1 d.r., ^{1}H NMR (400 MHz, $CDCl_3$) δ 8.05 (s, 1H), 7.61 – 7.55 (m, 2H), 7.48 – 7.28 (m, 8H), 6.99 – 6.86 (m, 7H), 6.50 (s, 1H), 5.64 (s, 1H), 5.47 (s, 1H), 3.87 – 3.80 (m, 6H), 2.21 (s, 3H).; ^{13}C NMR (101 MHz, $CDCl_3$) δ 159.4, 158.9, 155.7, 152.4, 148.7, 140.5, 132.2, 129.5, 129.3, 128.7, 128.7, 127.9, 126.8, 125.0, 124.6, 123.8, 121.0, 119.9, 117.4, 114.1, 113.9, 103.3, 80.7, 73.5, 55.3, 55.3, 12.0.

Enantiomeric excess of the product was determined by chiral stationary phase HPLC analysis using Daicel IA column (*n*-Hexane/*i*-PrOH 97:3 at 0.4 ml/min, λ = 230 nm, t_{major} = 32.9 min, t_{minor} = 45.2 min, 87% *ee*,; HRMS (ESI) *m/z* calcd for $C_{33}H_{30}O_5$, $[M+H]^+$: 507.2166, found: 507.2171.

2-((*S*)-(4-methoxyphenyl)((*R*)-(5-(4-methoxyphenyl)-2-methylfuran-3-yl)(phenyl)methoxy)methyl)phenol

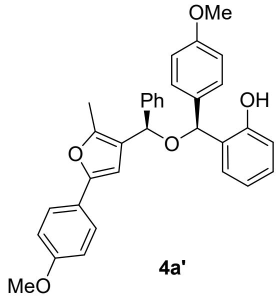

4a'

White solid, 30.3 mg, 40% yield, 4:1 d.r., ^{1}H NMR (400 MHz, $CDCl_3$) δ 8.29 (s, 0.8×1H), 8.03 (s, 0.2×1H), 7.60 – 7.57 (m, 2H), 7.48 – 7.28 (m, 8H), 6.99 – 6.85 (m, 6H), 6.82 – 6.78 (m, 1H), 6.48 (s, 1H), 5.68 (s, 0.8×1H), 5.62 (s, 0.2×1H), 5.46 (s, 1H), 3.86 – 3.81 (m, 6H), 2.22 (s, 3H).; ^{13}C NMR (101 MHz, $CDCl_3$) δ 159.6, 159.0, 155.6, 152.6, 148.8, 140.8, 131.9, 129.3, 129.1, 128.8, 128.8, 127.9, 126.7, 125.6, 125.0, 123.8, 120.7, 119.9, 117.3, 114.2, 114.1, 103.4, 81.0, 73.4, 55.3 (2C), 12.1.

Enantiomeric excess of the product was determined by chiral stationary phase HPLC analysis using Daicel IA column (*n*-Hexane/*i*-PrOH 97:3 at 0.6 ml/min, λ = 230 nm, t_{major} = 27.8 min, t_{minor} = 31.3 min, 89% *ee*,; HRMS (ESI) *m/z* calcd for $C_{33}H_{30}O_5$, $[M+H]^+$: 507.2166, found: 507.2173.

6. X-ray crystallographic data of 6b (Relative configuration)

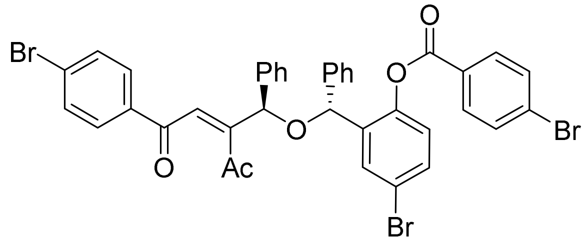

6b

Compound **6b** was synthesized from **3w**. The experiment was conducted as the synthesis of **6a** from **3a**.

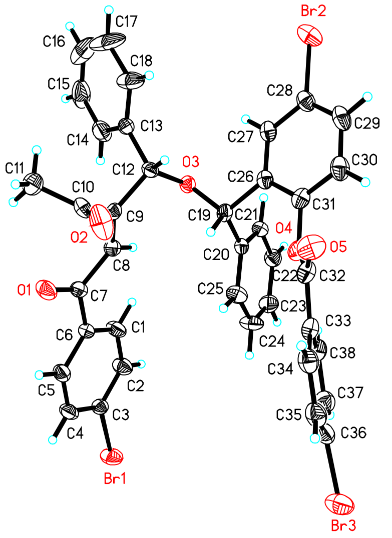

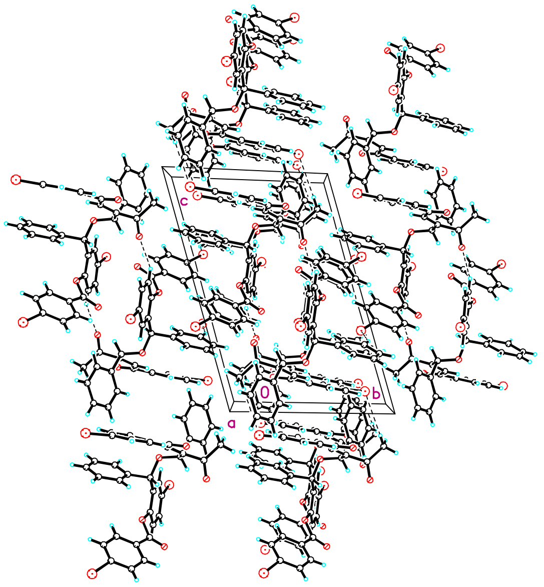

Supplementary Table 2 Crystal data and structure refinement for mo_d8v21786_0m.

Identification code	mo_d8v21786_0m	
Empirical formula	C38 H27 Br3 O5	
Formula weight	803.32	
Temperature	293(2) K	
Wavelength	0.71073 Å	
Crystal system	Triclinic	
Space group	P -1	
Unit cell dimensions	a = 10.1417(8) Å	α= 106.483(3)°.
	b = 10.7945(9) Å	β= 91.706(2)°.
	c = 16.0881(13) Å	γ = 101.766(2)°.
Volume	1646.2(2) $Å^3$	
Z	2	
Density (calculated)	1.621 Mg/m^3	
Absorption coefficient	3.720 mm^{-1}	
F(000)	800	

Crystal size	0.190 x 0.150 x 0.110 mm^3
Theta range for data collection	2.552 to 25.995°.
Index ranges	-12<=h<=12, -13<=k<=13, -19<=l<=19
Reflections collected	26494
Independent reflections	6456 [R(int) = 0.0629]
Completeness to theta = 25.242°	99.8 %
Absorption correction	Semi-empirical from equivalents
Max. and min. transmission	0.7456 and 0.6105
Refinement method	Full-matrix least-squares on F^2
Data / restraints / parameters	6456 / 0 / 416
Goodness-of-fit on F^2	1.026
Final R indices [I>2sigma(I)]	R1 = 0.0475, wR2 = 0.0957
R indices (all data)	R1 = 0.1028, wR2 = 0.1174
Extinction coefficient	n/a
Largest diff. peak and hole	0.492 and -0.506 e.$Å^{-3}$

Supplementary Table 3 Atomic coordinates (x 10^4) and equivalent isotropic displacement parameters ($Å^2$x 10^3) for mo_d8v21786_0m. U(eq) is defined as one third of the trace of the orthogonalized U^{ij} tensor.

	x	y	z	U(eq)
Br(1)	11778(1)	1414(1)	9145(1)	61(1)
Br(2)	67(1)	4367(1)	6306(1)	70(1)
Br(3)	9930(1)	-657(1)	3449(1)	82(1)
O(1)	9864(3)	6831(3)	8559(3)	62(1)
O(2)	8014(4)	7366(4)	7121(3)	85(1)
O(3)	4949(3)	4623(3)	7787(2)	36(1)
O(4)	5161(3)	2580(3)	5199(2)	51(1)
O(5)	5562(4)	3504(4)	4116(2)	69(1)
C(1)	9006(5)	3623(4)	8918(3)	51(1)
C(2)	9599(5)	2649(5)	9056(3)	53(1)
C(3)	10976(4)	2800(4)	9027(3)	39(1)
C(4)	11773(5)	3911(5)	8899(3)	50(1)
C(5)	11152(4)	4878(4)	8778(3)	49(1)
C(6)	9758(4)	4739(4)	8771(3)	40(1)
C(7)	9153(4)	5796(4)	8587(3)	44(1)
C(8)	7656(4)	5526(4)	8399(3)	42(1)
C(9)	7051(4)	6305(4)	8094(3)	35(1)
C(10)	7784(4)	7504(5)	7871(3)	44(1)
C(11)	8100(5)	8801(4)	8541(4)	63(2)
C(12)	5519(4)	5983(4)	7878(3)	36(1)
C(13)	4838(4)	6805(4)	8592(3)	43(1)
C(14)	5032(5)	6809(6)	9439(4)	69(2)
C(15)	4430(7)	7555(8)	10083(5)	107(3)
C(16)	3615(9)	8276(7)	9892(8)	123(4)
C(17)	3375(8)	8297(7)	9053(8)	120(4)
C(18)	3979(5)	7523(5)	8385(5)	71(2)
C(19)	5109(4)	3754(4)	6948(3)	36(1)
C(20)	5253(4)	2461(4)	7100(3)	35(1)
C(21)	4231(4)	1728(4)	7418(3)	38(1)
C(22)	4376(5)	573(4)	7577(3)	46(1)
C(23)	5540(5)	131(5)	7402(3)	54(1)
C(24)	6550(5)	828(5)	7075(4)	62(2)
C(25)	6426(5)	2020(5)	6929(3)	55(1)
C(26)	3914(4)	3601(4)	6301(3)	37(1)

C(27)	2739(4)	3978(4)	6564(3)	42(1)
C(28)	1679(5)	3839(5)	5947(3)	47(1)
C(29)	1787(5)	3336(5)	5074(4)	58(1)
C(30)	2950(5)	2925(5)	4802(3)	55(1)
C(31)	3998(5)	3061(4)	5420(3)	44(1)
C(32)	5832(5)	2793(5)	4520(3)	49(1)
C(33)	6888(5)	2011(4)	4329(3)	47(1)
C(34)	7836(5)	2312(5)	3772(3)	54(1)
C(35)	8769(5)	1542(5)	3520(3)	58(1)
C(36)	8738(5)	475(5)	3839(3)	54(1)
C(37)	7841(5)	192(5)	4410(3)	54(1)
C(38)	6902(5)	965(4)	4664(3)	49(1)

Supplementary Table 4 Bond lengths [Å] and angles [°] for mo_d8v21786_0m.

Br(1)-C(3)	1.893(4)
Br(2)-C(28)	1.892(5)
Br(3)-C(36)	1.888(5)
O(1)-C(7)	1.213(5)
O(2)-C(10)	1.209(6)
O(3)-C(12)	1.428(5)
O(3)-C(19)	1.444(5)
O(4)-C(32)	1.354(6)
O(4)-C(31)	1.399(5)
O(5)-C(32)	1.202(5)
C(1)-C(6)	1.373(6)
C(1)-C(2)	1.378(6)
C(1)-H(1)	0.9300
C(2)-C(3)	1.376(6)
C(2)-H(2)	0.9300
C(3)-C(4)	1.374(6)
C(4)-C(5)	1.379(6)
C(4)-H(4)	0.9300
C(5)-C(6)	1.390(6)
C(5)-H(5)	0.9300
C(6)-C(7)	1.494(6)
C(7)-C(8)	1.491(6)
C(8)-C(9)	1.323(6)
C(8)-H(8)	0.9300
C(9)-C(10)	1.498(6)
C(9)-C(12)	1.528(6)
C(10)-C(11)	1.471(7)
C(11)-H(11A)	0.9600
C(11)-H(11B)	0.9600
C(11)-H(11C)	0.9600
C(12)-C(13)	1.517(6)
C(12)-H(12)	0.9800
C(13)-C(18)	1.370(6)
C(13)-C(14)	1.370(7)
C(14)-C(15)	1.363(8)
C(14)-H(14)	0.9300
C(15)-C(16)	1.329(12)

C(15)-H(15)	0.9300
C(16)-C(17)	1.371(12)
C(16)-H(16)	0.9300
C(17)-C(18)	1.403(10)
C(17)-H(17)	0.9300
C(18)-H(18)	0.9300
C(19)-C(20)	1.517(6)
C(19)-C(26)	1.525(6)
C(19)-H(19)	0.9800
C(20)-C(21)	1.375(6)
C(20)-C(25)	1.376(6)
C(21)-C(22)	1.376(6)
C(21)-H(21)	0.9300
C(22)-C(23)	1.369(6)
C(22)-H(22)	0.9300
C(23)-C(24)	1.354(7)
C(23)-H(23)	0.9300
C(24)-C(25)	1.401(7)
C(24)-H(24)	0.9300
C(25)-H(25)	0.9300
C(26)-C(27)	1.377(6)
C(26)-C(31)	1.383(6)
C(27)-C(28)	1.396(6)
C(27)-H(27)	0.9300
C(28)-C(29)	1.370(7)
C(29)-C(30)	1.384(7)
C(29)-H(29)	0.9300
C(30)-C(31)	1.389(7)
C(30)-H(30)	0.9300
C(32)-C(33)	1.481(7)
C(33)-C(38)	1.385(6)
C(33)-C(34)	1.386(7)
C(34)-C(35)	1.379(7)
C(34)-H(34)	0.9300
C(35)-C(36)	1.383(7)
C(35)-H(35)	0.9300
C(36)-C(37)	1.363(7)
C(37)-C(38)	1.388(7)
C(37)-H(37)	0.9300

C(38)-H(38)	0.9300
C(12)-O(3)-C(19)	112.7(3)
C(32)-O(4)-C(31)	121.8(4)
C(6)-C(1)-C(2)	121.6(4)
C(6)-C(1)-H(1)	119.2
C(2)-C(1)-H(1)	119.2
C(1)-C(2)-C(3)	118.5(4)
C(1)-C(2)-H(2)	120.8
C(3)-C(2)-H(2)	120.8
C(4)-C(3)-C(2)	121.9(4)
C(4)-C(3)-Br(1)	119.5(3)
C(2)-C(3)-Br(1)	118.6(3)
C(3)-C(4)-C(5)	118.1(4)
C(3)-C(4)-H(4)	120.9
C(5)-C(4)-H(4)	120.9
C(4)-C(5)-C(6)	121.6(4)
C(4)-C(5)-H(5)	119.2
C(6)-C(5)-H(5)	119.2
C(1)-C(6)-C(5)	118.2(4)
C(1)-C(6)-C(7)	123.4(4)
C(5)-C(6)-C(7)	118.4(4)
O(1)-C(7)-C(8)	120.6(4)
O(1)-C(7)-C(6)	120.9(4)
C(8)-C(7)-C(6)	118.4(4)
C(9)-C(8)-C(7)	122.6(4)
C(9)-C(8)-H(8)	118.7
C(7)-C(8)-H(8)	118.7
C(8)-C(9)-C(10)	124.1(4)
C(8)-C(9)-C(12)	121.6(4)
C(10)-C(9)-C(12)	114.1(4)
O(2)-C(10)-C(11)	122.7(5)
O(2)-C(10)-C(9)	117.4(4)
C(11)-C(10)-C(9)	119.8(4)
C(10)-C(11)-H(11A)	109.5
C(10)-C(11)-H(11B)	109.5
H(11A)-C(11)-H(11B)	109.5
C(10)-C(11)-H(11C)	109.5
H(11A)-C(11)-H(11C)	109.5

H(11B)-C(11)-H(11C)	109.5
O(3)-C(12)-C(13)	107.2(3)
O(3)-C(12)-C(9)	111.0(3)
C(13)-C(12)-C(9)	111.4(3)
O(3)-C(12)-H(12)	109.1
C(13)-C(12)-H(12)	109.1
C(9)-C(12)-H(12)	109.1
C(18)-C(13)-C(14)	119.1(5)
C(18)-C(13)-C(12)	119.7(5)
C(14)-C(13)-C(12)	121.2(4)
C(15)-C(14)-C(13)	121.3(6)
C(15)-C(14)-H(14)	119.4
C(13)-C(14)-H(14)	119.4
C(16)-C(15)-C(14)	120.1(9)
C(16)-C(15)-H(15)	120.0
C(14)-C(15)-H(15)	120.0
C(15)-C(16)-C(17)	121.0(8)
C(15)-C(16)-H(16)	119.5
C(17)-C(16)-H(16)	119.5
C(16)-C(17)-C(18)	119.3(7)
C(16)-C(17)-H(17)	120.3
C(18)-C(17)-H(17)	120.3
C(13)-C(18)-C(17)	119.1(7)
C(13)-C(18)-H(18)	120.5
C(17)-C(18)-H(18)	120.5
O(3)-C(19)-C(20)	106.2(3)
O(3)-C(19)-C(26)	110.0(3)
C(20)-C(19)-C(26)	113.6(3)
O(3)-C(19)-H(19)	108.9
C(20)-C(19)-H(19)	108.9
C(26)-C(19)-H(19)	108.9
C(21)-C(20)-C(25)	119.2(4)
C(21)-C(20)-C(19)	120.9(4)
C(25)-C(20)-C(19)	119.9(4)
C(20)-C(21)-C(22)	120.9(4)
C(20)-C(21)-H(21)	119.6
C(22)-C(21)-H(21)	119.6
C(23)-C(22)-C(21)	119.9(5)
C(23)-C(22)-H(22)	120.1

C(21)-C(22)-H(22)	120.1
C(24)-C(23)-C(22)	120.1(5)
C(24)-C(23)-H(23)	119.9
C(22)-C(23)-H(23)	119.9
C(23)-C(24)-C(25)	120.5(4)
C(23)-C(24)-H(24)	119.8
C(25)-C(24)-H(24)	119.8
C(20)-C(25)-C(24)	119.4(5)
C(20)-C(25)-H(25)	120.3
C(24)-C(25)-H(25)	120.3
C(27)-C(26)-C(31)	118.3(4)
C(27)-C(26)-C(19)	122.1(4)
C(31)-C(26)-C(19)	119.6(4)
C(26)-C(27)-C(28)	120.1(4)
C(26)-C(27)-H(27)	120.0
C(28)-C(27)-H(27)	120.0
C(29)-C(28)-C(27)	121.1(5)
C(29)-C(28)-Br(2)	118.8(4)
C(27)-C(28)-Br(2)	120.1(4)
C(28)-C(29)-C(30)	119.4(5)
C(28)-C(29)-H(29)	120.3
C(30)-C(29)-H(29)	120.3
C(29)-C(30)-C(31)	119.1(5)
C(29)-C(30)-H(30)	120.4
C(31)-C(30)-H(30)	120.4
C(26)-C(31)-C(30)	121.9(5)
C(26)-C(31)-O(4)	115.7(4)
C(30)-C(31)-O(4)	122.2(4)
O(5)-C(32)-O(4)	123.2(5)
O(5)-C(32)-C(33)	125.2(5)
O(4)-C(32)-C(33)	111.6(4)
C(38)-C(33)-C(34)	120.3(5)
C(38)-C(33)-C(32)	121.2(5)
C(34)-C(33)-C(32)	118.4(4)
C(35)-C(34)-C(33)	120.4(5)
C(35)-C(34)-H(34)	119.8
C(33)-C(34)-H(34)	119.8
C(34)-C(35)-C(36)	118.4(5)
C(34)-C(35)-H(35)	120.8

C(36)-C(35)-H(35)	120.8
C(37)-C(36)-C(35)	121.9(5)
C(37)-C(36)-Br(3)	118.8(4)
C(35)-C(36)-Br(3)	119.3(4)
C(36)-C(37)-C(38)	119.7(5)
C(36)-C(37)-H(37)	120.1
C(38)-C(37)-H(37)	120.1
C(33)-C(38)-C(37)	119.2(5)
C(33)-C(38)-H(38)	120.4
C(37)-C(38)-H(38)	120.4

Symmetry transformations used to generate equivalent atoms:

Supplementary Table 5 Anisotropic displacement parameters (Å^{2}x 10^3) for mo_d8v21786_0m. The anisotropic displacement factor exponent takes the form: $-2\pi^2[h^2 a^{*2}U^{11} + ... + 2 h k a^* b^* U^{12}]$

	U^{11}	U^{22}	U^{33}	U^{23}	U^{13}	U^{12}
Br(1)	53(1)	62(1)	83(1)	33(1)	8(1)	28(1)
Br(2)	44(1)	89(1)	86(1)	40(1)	1(1)	14(1)
Br(3)	52(1)	95(1)	107(1)	33(1)	17(1)	26(1)
O(1)	36(2)	45(2)	108(3)	31(2)	1(2)	2(2)
O(2)	80(3)	112(3)	51(3)	38(2)	-3(2)	-27(2)
O(3)	35(2)	32(2)	38(2)	9(1)	4(1)	4(1)
O(4)	68(2)	54(2)	37(2)	15(2)	10(2)	22(2)
O(5)	87(3)	61(2)	75(3)	40(2)	20(2)	24(2)
C(1)	29(2)	50(3)	81(4)	28(3)	7(2)	8(2)
C(2)	40(3)	49(3)	76(4)	29(3)	8(3)	10(2)
C(3)	39(3)	42(3)	38(3)	12(2)	1(2)	15(2)
C(4)	31(2)	51(3)	70(4)	19(3)	1(2)	12(2)
C(5)	36(3)	41(3)	67(4)	15(2)	0(2)	5(2)
C(6)	29(2)	41(3)	45(3)	6(2)	2(2)	10(2)
C(7)	32(2)	38(3)	59(3)	12(2)	4(2)	9(2)
C(8)	36(2)	33(2)	55(3)	12(2)	5(2)	6(2)
C(9)	36(2)	34(2)	30(3)	4(2)	5(2)	6(2)
C(10)	35(2)	52(3)	45(3)	20(3)	-7(2)	2(2)
C(11)	61(3)	39(3)	88(4)	21(3)	1(3)	6(3)
C(12)	30(2)	35(2)	47(3)	15(2)	0(2)	7(2)
C(13)	27(2)	35(2)	59(4)	6(2)	5(2)	2(2)
C(14)	45(3)	92(4)	52(4)	-4(3)	0(3)	13(3)
C(15)	59(4)	122(6)	80(5)	-41(5)	20(4)	-11(4)
C(16)	88(6)	53(5)	195(11)	-11(6)	88(7)	-5(4)
C(17)	85(5)	55(4)	256(12)	74(7)	90(7)	43(4)
C(18)	54(3)	53(3)	127(6)	46(4)	31(3)	24(3)
C(19)	37(2)	37(2)	29(3)	6(2)	8(2)	2(2)
C(20)	33(2)	35(2)	31(3)	3(2)	-2(2)	6(2)
C(21)	32(2)	41(2)	42(3)	11(2)	4(2)	9(2)
C(22)	46(3)	40(3)	51(3)	15(2)	2(2)	6(2)
C(23)	58(3)	49(3)	60(4)	17(3)	1(3)	20(3)
C(24)	47(3)	70(4)	83(4)	27(3)	13(3)	35(3)
C(25)	39(3)	65(3)	62(4)	22(3)	12(3)	13(2)
C(26)	42(3)	31(2)	38(3)	14(2)	-1(2)	2(2)

C(27)	44(3)	39(3)	39(3)	16(2)	0(2)	-2(2)
C(28)	42(3)	47(3)	53(4)	22(3)	-3(2)	1(2)
C(29)	56(3)	58(3)	56(4)	24(3)	-17(3)	-2(3)
C(30)	66(4)	56(3)	40(3)	17(2)	-7(3)	5(3)
C(31)	58(3)	36(3)	39(3)	14(2)	5(2)	8(2)
C(32)	63(3)	37(3)	43(3)	9(2)	4(3)	7(2)
C(33)	55(3)	36(3)	41(3)	6(2)	0(2)	3(2)
C(34)	50(3)	52(3)	55(4)	20(3)	4(3)	-6(3)
C(35)	46(3)	67(4)	53(4)	19(3)	8(3)	-7(3)
C(36)	38(3)	59(3)	57(4)	14(3)	-1(3)	1(2)
C(37)	54(3)	49(3)	59(4)	17(3)	-1(3)	8(3)
C(38)	56(3)	46(3)	41(3)	11(2)	4(2)	6(2)

Supplementary Table 6 Hydrogen coordinates (x 10^4) and isotropic displacement parameters ($Å^2$x 10^3) for mo_d8v21786_0m.

	x	y	z	U(eq)
H(1)	8074	3522	8924	62
H(2)	9081	1906	9167	63
H(4)	12705	4008	8895	60
H(5)	11678	5642	8700	59
H(8)	7127	4780	8499	50
H(11A)	8574	9460	8296	94
H(11B)	8658	8758	9020	94
H(11C)	7276	9032	8742	94
H(12)	5320	6167	7331	44
H(14)	5585	6293	9578	82
H(15)	4590	7560	10656	128
H(16)	3200	8774	10334	148
H(17)	2817	8819	8929	144
H(18)	3799	7499	7810	86
H(19)	5943	4141	6736	43
H(21)	3432	2015	7527	46
H(22)	3685	94	7803	55
H(23)	5638	-651	7508	65
H(24)	7331	514	6948	75
H(25)	7129	2508	6717	65
H(27)	2650	4326	7153	50
H(29)	1084	3271	4669	69
H(30)	3030	2562	4213	66
H(34)	7843	3037	3567	65
H(35)	9405	1736	3144	69
H(37)	7859	-517	4627	65
H(38)	6289	784	5056	59

Supplementary Table 7 Torsion angles [°] for mo_d8v21786_0m.

C(6)-C(1)-C(2)-C(3)	-1.3(8)
C(1)-C(2)-C(3)-C(4)	2.6(8)
C(1)-C(2)-C(3)-Br(1)	-176.1(4)
C(2)-C(3)-C(4)-C(5)	-1.5(7)
Br(1)-C(3)-C(4)-C(5)	177.2(4)
C(3)-C(4)-C(5)-C(6)	-0.9(8)
C(2)-C(1)-C(6)-C(5)	-1.0(8)
C(2)-C(1)-C(6)-C(7)	177.8(5)
C(4)-C(5)-C(6)-C(1)	2.2(7)
C(4)-C(5)-C(6)-C(7)	-176.7(5)
C(1)-C(6)-C(7)-O(1)	171.6(5)
C(5)-C(6)-C(7)-O(1)	-9.5(7)
C(1)-C(6)-C(7)-C(8)	-11.2(7)
C(5)-C(6)-C(7)-C(8)	167.6(4)
O(1)-C(7)-C(8)-C(9)	8.3(7)
C(6)-C(7)-C(8)-C(9)	-168.9(4)
C(7)-C(8)-C(9)-C(10)	2.5(7)
C(7)-C(8)-C(9)-C(12)	178.0(4)
C(8)-C(9)-C(10)-O(2)	94.9(6)
C(12)-C(9)-C(10)-O(2)	-80.9(5)
C(8)-C(9)-C(10)-C(11)	-89.7(6)
C(12)-C(9)-C(10)-C(11)	94.5(5)
C(19)-O(3)-C(12)-C(13)	158.3(3)
C(19)-O(3)-C(12)-C(9)	-79.9(4)
C(8)-C(9)-C(12)-O(3)	-18.0(6)
C(10)-C(9)-C(12)-O(3)	157.9(4)
C(8)-C(9)-C(12)-C(13)	101.4(5)
C(10)-C(9)-C(12)-C(13)	-82.8(5)
O(3)-C(12)-C(13)-C(18)	-111.2(4)
C(9)-C(12)-C(13)-C(18)	127.2(4)
O(3)-C(12)-C(13)-C(14)	66.8(5)
C(9)-C(12)-C(13)-C(14)	-54.8(5)
C(18)-C(13)-C(14)-C(15)	-2.6(8)
C(12)-C(13)-C(14)-C(15)	179.4(5)
C(13)-C(14)-C(15)-C(16)	1.4(10)
C(14)-C(15)-C(16)-C(17)	-0.7(12)
C(15)-C(16)-C(17)-C(18)	1.3(12)

C(14)-C(13)-C(18)-C(17)	3.2(8)
C(12)-C(13)-C(18)-C(17)	-178.9(5)
C(16)-C(17)-C(18)-C(13)	-2.5(10)
C(12)-O(3)-C(19)-C(20)	147.7(3)
C(12)-O(3)-C(19)-C(26)	-88.9(4)
O(3)-C(19)-C(20)-C(21)	61.4(5)
C(26)-C(19)-C(20)-C(21)	-59.7(5)
O(3)-C(19)-C(20)-C(25)	-117.8(4)
C(26)-C(19)-C(20)-C(25)	121.1(5)
C(25)-C(20)-C(21)-C(22)	0.9(7)
C(19)-C(20)-C(21)-C(22)	-178.3(4)
C(20)-C(21)-C(22)-C(23)	-1.3(7)
C(21)-C(22)-C(23)-C(24)	0.2(8)
C(22)-C(23)-C(24)-C(25)	1.2(8)
C(21)-C(20)-C(25)-C(24)	0.5(7)
C(19)-C(20)-C(25)-C(24)	179.7(4)
C(23)-C(24)-C(25)-C(20)	-1.5(8)
O(3)-C(19)-C(26)-C(27)	-14.7(5)
C(20)-C(19)-C(26)-C(27)	104.3(5)
O(3)-C(19)-C(26)-C(31)	165.6(3)
C(20)-C(19)-C(26)-C(31)	-75.4(5)
C(31)-C(26)-C(27)-C(28)	-1.3(6)
C(19)-C(26)-C(27)-C(28)	178.9(4)
C(26)-C(27)-C(28)-C(29)	-0.3(7)
C(26)-C(27)-C(28)-Br(2)	-179.7(3)
C(27)-C(28)-C(29)-C(30)	1.8(7)
Br(2)-C(28)-C(29)-C(30)	-178.9(4)
C(28)-C(29)-C(30)-C(31)	-1.5(7)
C(27)-C(26)-C(31)-C(30)	1.6(6)
C(19)-C(26)-C(31)-C(30)	-178.6(4)
C(27)-C(26)-C(31)-O(4)	-174.1(4)
C(19)-C(26)-C(31)-O(4)	5.6(6)
C(29)-C(30)-C(31)-C(26)	-0.2(7)
C(29)-C(30)-C(31)-O(4)	175.2(4)
C(32)-O(4)-C(31)-C(26)	-137.9(4)
C(32)-O(4)-C(31)-C(30)	46.4(6)
C(31)-O(4)-C(32)-O(5)	7.3(7)
C(31)-O(4)-C(32)-C(33)	-170.8(4)
O(5)-C(32)-C(33)-C(38)	-162.7(5)

O(4)-C(32)-C(33)-C(38)	15.4(6)
O(5)-C(32)-C(33)-C(34)	14.6(7)
O(4)-C(32)-C(33)-C(34)	-167.4(4)
C(38)-C(33)-C(34)-C(35)	2.5(7)
C(32)-C(33)-C(34)-C(35)	-174.7(4)
C(33)-C(34)-C(35)-C(36)	-0.3(7)
C(34)-C(35)-C(36)-C(37)	-1.9(8)
C(34)-C(35)-C(36)-Br(3)	176.1(4)
C(35)-C(36)-C(37)-C(38)	1.8(8)
Br(3)-C(36)-C(37)-C(38)	-176.2(4)
C(34)-C(33)-C(38)-C(37)	-2.5(7)
C(32)-C(33)-C(38)-C(37)	174.6(4)
C(36)-C(37)-C(38)-C(33)	0.4(7)

Symmetry transformations used to generate equivalent atoms:

Supplementary Table 8 Hydrogen bonds for mo_d8v21786_0m [Å and °].

D-H...A	d(D-H)	d(H...A)	d(D...A)	<(DHA)
C(11)-H(11B)...Br(1)#1	0.96	3.05	3.796(6)	135.6
C(11)-H(11B)...O(1)	0.96	2.57	3.051(6)	111.4
C(12)-H(12)...O(5)#2	0.98	2.61	3.584(6)	171.9
C(30)-H(30)...O(2)#2	0.93	2.39	3.126(6)	135.6

Symmetry transformations used to generate equivalent atoms:

#1 -x+2,-y+1,-z+2 #2 -x+1,-y+1,-z+1

7. CD experimental and theory computational spectra of 3a and 4j

Computational details

All the structural optimizations and calculations were performed using the Gaussian 16 package.[11] Geometries of molecules were computed using DFT with the B3LYP/6-311++G(d,p)[12-14] functional and basis set, and the polarizable continuum model (PCM) solvation model[15] in dichloromethane. Structures were confirmed to be minima on the potential energy surface via the inclusion of a frequency calculation revealing no imaginary frequencies. ECD spectra were predicted via single points using TD-DFT[16] B3LYP/6-311++G(2d,p) (nstates=30) with the PCM solvation model in dichloromethane. All predicted ECD spectra were compared to experimentally obtained solvent blank corrected ECD spectra on **3a** and **4j**, respectively.

In our calculated ECD spectra of **3a**, there is a strong peak at 310 nm compared with the experimental spectra. We think it that our calculation overestimates the intensity of signal at 310 nm because of the absence of explicit dichloromethane molecule (such as the interaction between solvent and solute).

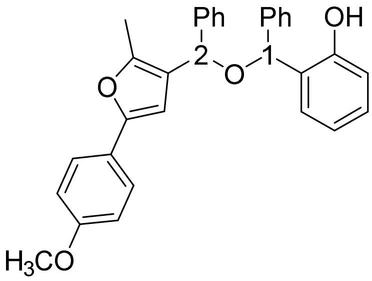

Supplementary Table 9 The configuration of **3a**, enantiomer of **3a**, **4j** and enantiomer of **4j**.

product	1	2
**3a**	*R*	*R*
enantiomer of **3a**	*S*	*S*
**4j**	*R*	*S*
enantiomer of **4j**	*S*	*R*

3a

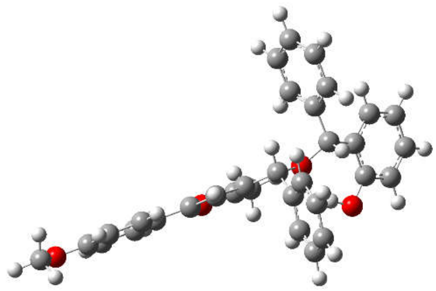

No imaginary frequencies found.

Zero-point correction=	0.516856 (Hartree/Particle)
Thermal correction to Energy=	0.548272
Thermal correction to Enthalpy=	0.549216
Thermal correction to Gibbs Free Energy=	0.447200
Sum of electronic and zero-point Energies=	-1537.036955

Sum of electronic and thermal Energies=	-1537.005538
Sum of electronic and thermal Enthalpies=	-1537.004594
Sum of electronic and thermal Free Energies=	-1537.106611

Supplementary Table 10 Forces acting on atoms in compound **3a**

Center Number	Atomic Number	Forces (Hartrees/Bohr)		
		X	Y	Z
1	6	0.000001676	0.000004518	0.000001209
2	6	-0.000003453	-0.000001437	0.000000588
3	6	0.000001004	-0.000000879	0.000000461
4	6	-0.000001379	-0.000000213	0.000001302
5	6	-0.000000335	-0.000000398	0.000002794
6	6	-0.000001009	-0.000006152	-0.000001202
7	8	0.000001958	0.000004750	-0.000004244
8	6	0.000009633	0.000002329	-0.000005688
9	8	-0.000012762	-0.000010011	0.000003487
10	6	-0.000004277	-0.000006176	0.000001769
11	6	0.000011674	0.000014716	0.000004428
12	6	-0.000004169	0.000001146	-0.000008326
13	6	-0.000011652	0.000000719	0.000009254
14	8	0.000010103	-0.000002591	-0.000002373
15	6	-0.000002205	0.000005378	0.000001101
16	6	0.000008159	-0.000006169	0.000001394
17	6	0.000006833	-0.000001156	-0.000005291
18	6	-0.000001370	-0.000002378	-0.000005701
19	6	0.000003126	0.000001251	0.000005258
20	6	-0.000002621	-0.000000518	-0.000001259
21	6	0.000007947	-0.000002637	-0.000007720
22	6	-0.000007386	0.000000162	0.000000261
23	6	0.000000167	0.000002574	0.000007803
24	6	-0.000000691	-0.000003405	0.000004207
25	6	-0.000004473	0.000005976	0.000004518
26	6	-0.000000177	-0.000000077	0.000000836
27	6	0.000000598	-0.000000104	-0.000002884
28	6	-0.000000477	-0.000002095	-0.000000272
29	6	-0.000001327	-0.000005553	0.000004559
30	6	0.000000457	0.000005524	-0.000003841
31	6	0.000003421	-0.000001646	-0.000012056
32	6	-0.000002728	-0.000000805	0.000008804
33	6	0.000000437	0.000007481	0.000001528
34	6	0.000000563	-0.000007022	-0.000005210
35	8	-0.000002839	-0.000000011	0.000006110
36	6	0.000002731	-0.000001129	-0.000004830
37	1	0.000000090	0.000000770	-0.000001005
38	1	0.000000830	0.000000863	-0.000000369

39	1	0.000000209	-0.000000316	0.000000689
40	1	0.000000208	0.000001779	-0.000002456
41	1	0.000001816	0.000002906	0.000006712
42	1	-0.000002082	0.000001352	-0.000001090
43	1	0.000000733	0.000000794	-0.000003679
44	1	-0.000001956	-0.000000298	-0.000000502
45	1	-0.000002460	-0.000001882	0.000000904
46	1	-0.000000233	-0.000001939	0.000002598
47	1	-0.000000589	0.000002730	0.000000432
48	1	-0.000001704	-0.000001780	-0.000004136
49	1	-0.000001706	0.000000577	0.000001423
50	1	0.000003175	0.000001679	0.000004164
51	1	0.000002061	-0.000001923	-0.000003922
52	1	-0.000000616	0.000003303	0.000001070
53	1	0.000002774	-0.000000416	-0.000005952
54	1	-0.000000120	-0.000000982	0.000001698
55	1	0.000000123	-0.000000319	-0.000000665
56	1	-0.000000200	-0.000000170	-0.000001327
57	1	-0.000001556	-0.000000634	0.000001751
58	1	-0.000001000	-0.000002955	0.000004121
59	1	0.000000570	0.000002442	-0.000000489
60	1	0.000000188	-0.000000883	-0.000000483
61	1	0.000000134	0.000001715	-0.000001323
62	1	-0.000003789	0.000000367	0.000001040
63	1	-0.000000087	0.000000196	0.000000120
64	1	0.000000030	-0.000000935	-0.000000098

enantiomer of 3a

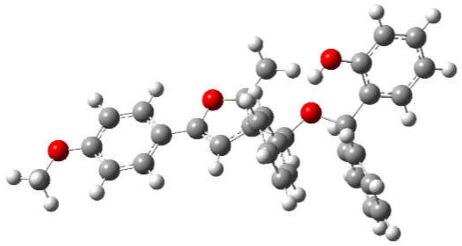

No imaginary frequencies found.

Zero-point correction=	0.516856 (Hartree/Particle)
Thermal correction to Energy=	0.548272
Thermal correction to Enthalpy=	0.549216
Thermal correction to Gibbs Free Energy=	0.447237
Sum of electronic and zero-point Energies=	-1537.036955
Sum of electronic and thermal Energies=	-1537.005539
Sum of electronic and thermal Enthalpies=	-1537.004595
Sum of electronic and thermal Free Energies=	-1537.106573

Supplementary Table 11 Forces acting on atoms in enantiomer of **3a**

Center Number	Atomic Number	Forces (Hartrees/Bohr)		
		X	Y	Z
1	6	0.000004565	-0.000005504	0.000002649
2	6	-0.000005036	0.000004021	-0.000002944
3	6	0.000001048	0.000002790	-0.000001365
4	6	0.000002700	-0.000004885	-0.000001181
5	6	-0.000003932	0.000004505	-0.000002554
6	6	0.000002674	0.000002184	0.000002730
7	8	-0.000002314	-0.000000014	0.000000637
8	6	-0.000004515	0.000000838	-0.000001996
9	8	0.000002893	-0.000000798	0.000001789
10	6	-0.000000192	-0.000000081	-0.000003337
11	6	-0.000002287	0.000002938	0.000001014
12	6	0.000004716	0.000008733	-0.000000159
13	6	-0.000001970	-0.000008619	0.000002570
14	8	-0.000002272	0.000000648	-0.000002763
15	6	0.000002837	0.000001146	-0.000000382
16	6	0.000000375	-0.000006283	-0.000004731
17	6	-0.000004202	-0.000000997	-0.000000874
18	6	-0.000001546	0.000000084	-0.000000111
19	6	-0.000000889	-0.000001566	-0.000000849
20	6	0.000005711	-0.000000156	0.000001994
21	6	0.000002361	0.000001618	-0.000001005
22	6	-0.000000157	-0.000001540	-0.000004254
23	6	0.000001866	-0.000001905	-0.000003135
24	6	-0.000000095	0.000003512	0.000001807
25	6	0.000000122	-0.000000034	-0.000001875
26	6	-0.000000556	0.000000366	-0.000001192
27	6	-0.000000583	0.000000051	-0.000000727
28	6	-0.000002274	-0.000002181	-0.000005995
29	6	-0.000000102	-0.000002790	0.000004726
30	6	-0.000001144	-0.000001765	-0.000004422
31	6	-0.000000821	0.000001865	-0.000004544
32	6	0.000001261	-0.000002952	0.000002763
33	6	-0.000001164	0.000002542	-0.000004935
34	6	0.000000246	0.000001047	-0.000002060
35	8	-0.000002881	-0.000000645	0.000001162
36	6	-0.000004344	-0.000001405	-0.000001413
37	1	0.000000364	-0.000000757	0.000001797
38	1	0.000001212	-0.000001004	0.000000671
39	1	-0.000001851	0.000000558	0.000002168
40	1	0.000000837	-0.000000925	0.000000635
41	1	0.000000123	-0.000000303	-0.000001204
42	1	0.000000979	0.000000566	0.000000013

43	1	0.000000495	-0.000000283	-0.000000918
44	1	-0.000001470	0.000002841	0.000006066
45	1	-0.000000020	0.000000162	-0.000000078
46	1	0.000004046	0.000000439	0.000001649
47	1	-0.000001049	0.000001732	-0.000000002
48	1	-0.000001865	0.000001041	0.000002097
49	1	-0.000001750	-0.000001224	-0.000002288
50	1	-0.000001796	0.000000984	0.000003230
51	1	0.000001257	0.000000735	0.000002328
52	1	-0.000000278	-0.000000270	0.000000054
53	1	0.000001551	0.000000696	0.000003093
54	1	0.000000140	-0.000001666	0.000001672
55	1	0.000000317	0.000000910	0.000000242
56	1	0.000002238	0.000000903	0.000006471
57	1	-0.000000220	0.000001404	-0.000001011
58	1	0.000000292	0.000000677	0.000002736
59	1	-0.000000488	0.000001611	0.000000422
60	1	0.000000437	-0.000002355	0.000003515
61	1	0.000000297	-0.000000961	0.000000844
62	1	0.000006260	0.000000598	0.000001093
63	1	-0.000000015	-0.000001328	0.000000466
64	1	-0.000000140	0.000000448	-0.000000801

4j

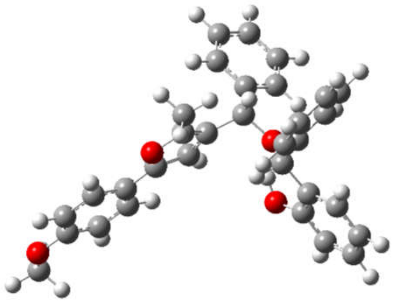

No imaginary frequencies found.

Zero-point correction=	0.516948 (Hartree/Particle)
Thermal correction to Energy=	0.548284
Thermal correction to Enthalpy=	0.549228
Thermal correction to Gibbs Free Energy=	0.448510
Sum of electronic and zero-point Energies=	-1537.039536
Sum of electronic and thermal Energies=	-1537.008200
Sum of electronic and thermal Enthalpies=	-1537.007256
Sum of electronic and thermal Free Energies=	-1537.107974

Supplementary Table 12 Forces acting on atoms in compound **4j**

Center Number	Atomic Number	Forces (Hartrees/Bohr)		
		X	Y	Z
1	6	-0.000004479	0.000000833	-0.000001488
2	6	0.000003893	-0.000002783	0.000003178
3	6	0.000002263	0.000003967	0.000006303
4	6	-0.000003503	-0.000001694	-0.000003173
5	6	0.000000761	-0.000003226	-0.000000729
6	6	0.000006457	0.000000647	0.000006333
7	8	-0.000001518	0.000002727	0.000000184
8	6	0.000000629	-0.000002631	-0.000000305
9	8	-0.000001320	0.000002160	0.000000857
10	6	0.000001270	0.000002187	-0.000003958
11	6	0.000002317	-0.000006364	-0.000003090
12	6	0.000003031	-0.000005175	0.000003335
13	6	-0.000001784	0.000004091	0.000001190
14	8	0.000000508	0.000002426	-0.000003312
15	6	0.000000803	-0.000008690	0.000001159
16	6	-0.000003038	0.000008983	-0.000005973
17	6	0.000002723	0.000000395	-0.000001397
18	6	-0.000002836	-0.000000609	0.000000984
19	6	0.000001826	0.000001444	-0.000002141
20	6	-0.000000484	0.000000176	-0.000004251
21	6	0.000004690	-0.000000353	-0.000002146
22	6	0.000000072	0.000000130	0.000000104
23	6	-0.000004216	-0.000000880	0.000001526
24	6	-0.000001885	-0.000001161	0.000003329
25	6	-0.000002480	-0.000000024	0.000005058
26	6	0.000001204	0.000001130	-0.000000088
27	6	-0.000001462	-0.000000941	0.000000958
28	6	-0.000001551	-0.000001037	0.000000878
29	6	-0.000000291	0.000001729	-0.000001466
30	6	-0.000000533	0.000000116	-0.000000326
31	6	-0.000001042	-0.000000219	0.000001112
32	6	0.000000645	0.000000749	-0.000003403
33	6	0.000000284	0.000000076	0.000001996
34	6	-0.000002039	0.000001352	0.000001222
35	8	-0.000002959	0.000000376	-0.000002428
36	6	0.000001626	-0.000000659	0.000002457
37	1	-0.000002781	-0.000000181	-0.000001842
38	1	-0.000000096	-0.000000307	0.000001287
39	1	0.000000827	-0.000000737	0.000001691
40	1	0.000000494	0.000000656	0.000001964
41	1	-0.000000449	-0.000000639	0.000000218
42	1	-0.000000331	0.000000138	-0.000000152

43	1	-0.000000038	0.000000922	0.000001337
44	1	0.000000428	0.000001012	0.000002813
45	1	-0.000000583	-0.000000036	0.000000848
46	1	-0.000001540	0.000003216	0.000000552
47	1	-0.000000104	0.000000437	-0.000000911
48	1	0.000000105	0.000000364	-0.000000072
49	1	-0.000002413	0.000000232	0.000002418
50	1	0.000000657	0.000000128	0.000000800
51	1	0.000001905	-0.000000239	-0.000003381
52	1	0.000001001	0.000001189	-0.000000524
53	1	-0.000000105	0.000001095	-0.000001538
54	1	0.000000059	0.000000620	0.000001277
55	1	0.000000895	0.000001237	-0.000000751
56	1	0.000000048	0.000000009	0.000000638
57	1	0.000000277	-0.000000211	-0.000000943
58	1	0.000000818	-0.000001056	-0.000001024
59	1	0.000000374	-0.000002249	0.000000629
60	1	0.000001783	-0.000001503	-0.000002504
61	1	0.000001938	-0.000003319	-0.000001024
62	1	-0.000001161	-0.000000647	-0.000000825
63	1	0.000000133	0.000001217	-0.000002501
64	1	0.000000271	-0.000000599	-0.000000969

enantiomer of 4j

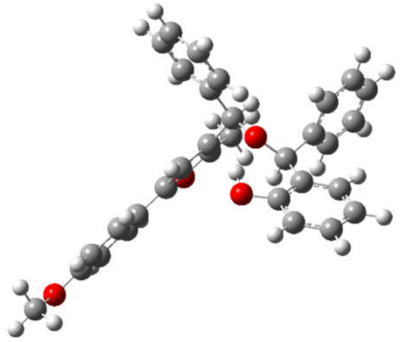

No imaginary frequencies found.

Zero-point correction=	0.516949 (Hartree/Particle)
Thermal correction to Energy=	0.548284
Thermal correction to Enthalpy=	0.549228
Thermal correction to Gibbs Free Energy=	0.448510
Sum of electronic and zero-point Energies=	-1537.039535
Sum of electronic and thermal Energies=	-1537.008200
Sum of electronic and thermal Enthalpies=	-1537.007256
Sum of electronic and thermal Free Energies=	-1537.107975

Supplementary Table 13 Forces acting on atoms in enantiomer of **4j**

Center Number	Atomic Number	Forces (Hartrees/Bohr)		
		X	Y	Z
1	6	0.000002764	0.000000161	-0.000001805
2	6	0.000000347	0.000000392	0.000000046
3	6	0.000002889	0.000001149	0.000001987
4	6	-0.000000203	0.000002942	0.000001115
5	6	-0.000002960	-0.000001566	0.000001182
6	6	0.000002452	-0.000001426	0.000004031
7	8	-0.000000828	-0.000003582	-0.000003982
8	6	0.000002371	-0.000000058	0.000003833
9	8	-0.000003693	0.000003150	0.000000624
10	6	0.000001623	-0.000000408	0.000002784
11	6	0.000001318	-0.000000835	0.000001859
12	6	-0.000003523	-0.000009074	-0.000000661
13	6	0.000007381	0.000006781	-0.000001908
14	8	-0.000004360	0.000001861	0.000002671
15	6	0.000000040	-0.000008481	-0.000006825
16	6	0.000002994	0.000010019	0.000000109
17	6	-0.000001307	0.000003650	0.000000730
18	6	-0.000002845	0.000001165	0.000002583
19	6	-0.000002419	0.000002832	0.000002737
20	6	0.000003082	0.000001373	-0.000001010
21	6	-0.000000177	-0.000000645	0.000000527
22	6	-0.000002946	0.000000210	-0.000001214
23	6	0.000001618	-0.000000883	-0.000001426
24	6	-0.000001536	0.000002352	-0.000003541
25	6	-0.000002067	0.000002025	-0.000002779
26	6	-0.000000507	-0.000000462	0.000003764
27	6	0.000000183	0.000001649	0.000000374
28	6	-0.000000487	-0.000001281	0.000003612
29	6	0.000003097	0.000002703	-0.000001347
30	6	0.000001354	0.000002788	-0.000002158
31	6	-0.000002724	-0.000004551	0.000002902
32	6	0.000002690	-0.000000461	0.000002839
33	6	-0.000003824	-0.000001353	-0.000005439
34	6	-0.000003911	-0.000004894	-0.000002387
35	8	-0.000000533	-0.000002023	-0.000001004
36	6	0.000002719	0.000003284	-0.000000768
37	1	-0.000001019	0.000000309	0.000001798
38	1	-0.000001727	-0.000002127	0.000001630
39	1	-0.000000572	-0.000001916	0.000000693
40	1	-0.000000192	-0.000000461	0.000001164
41	1	-0.000000334	0.000000764	0.000001962
42	1	-0.000003404	0.000000019	-0.000002094

43	1	-0.000000739	0.000000114	0.000000311
44	1	0.000000075	0.000000594	0.000001901
45	1	0.000002357	0.000000952	0.000000106
46	1	0.000000000	-0.000000784	0.000000745
47	1	-0.000000357	-0.000001606	-0.000001135
48	1	0.000001453	-0.000000378	-0.000003879
49	1	0.000000085	0.000000574	-0.000001193
50	1	0.000000201	-0.000000434	-0.000001168
51	1	-0.000000639	-0.000000785	-0.000000607
52	1	0.000001776	-0.000000335	0.000000874
53	1	0.000001253	0.000000316	0.000002813
54	1	0.000002901	-0.000000210	-0.000001560
55	1	0.000000411	-0.000000274	0.000001177
56	1	0.000000449	0.000000227	-0.000003350
57	1	-0.000001783	-0.000002098	-0.000000219
58	1	-0.000000250	-0.000000671	-0.000000382
59	1	-0.000000729	0.000000132	-0.000002259
60	1	0.000001666	0.000001613	0.000000795
61	1	0.000002000	0.000000768	0.000002156
62	1	-0.000000054	0.000000427	-0.000001412
63	1	-0.000000703	-0.000001624	-0.000001042
64	1	-0.000000199	-0.000001610	0.000000120

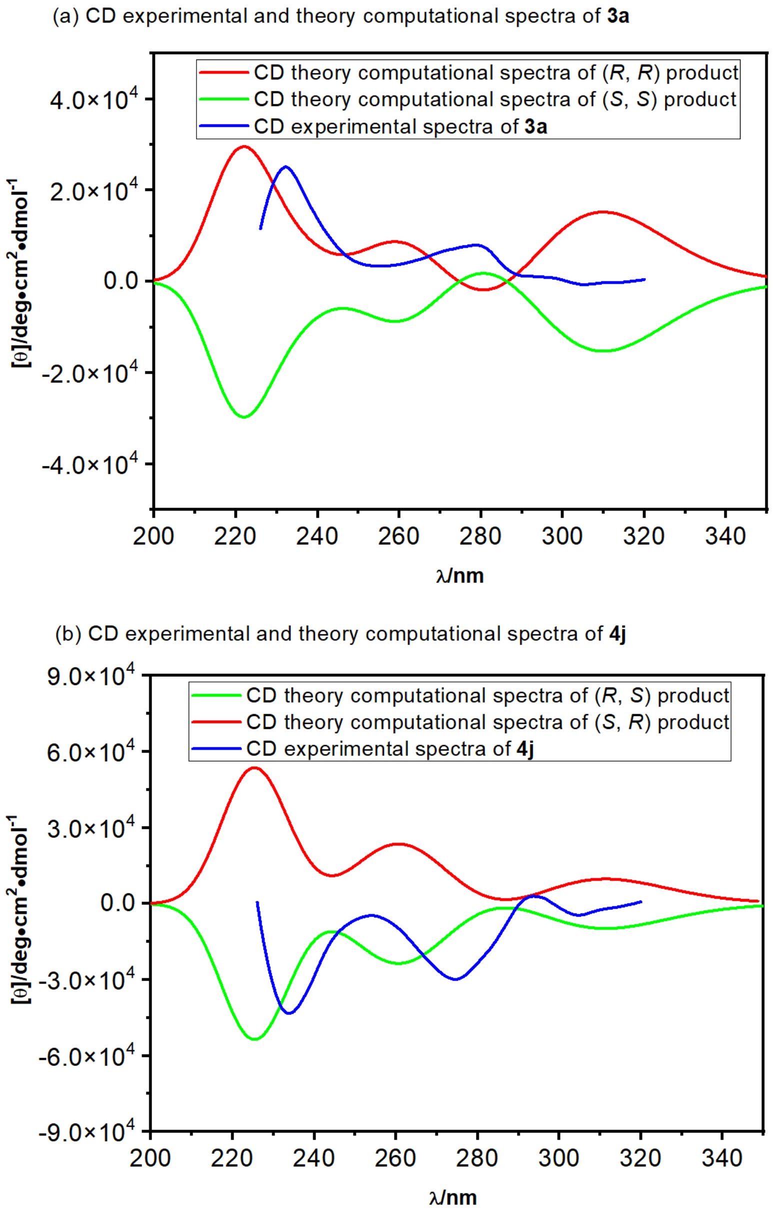

Supplementary Fig. 2 CD experimental and theory computational spectra. (a) CD experimental and theory computational spectra of **3a**. (b) CD experimental and theory computational spectra of **4j**.

8. ^{1}H NMR and ^{13}C NMR spectra

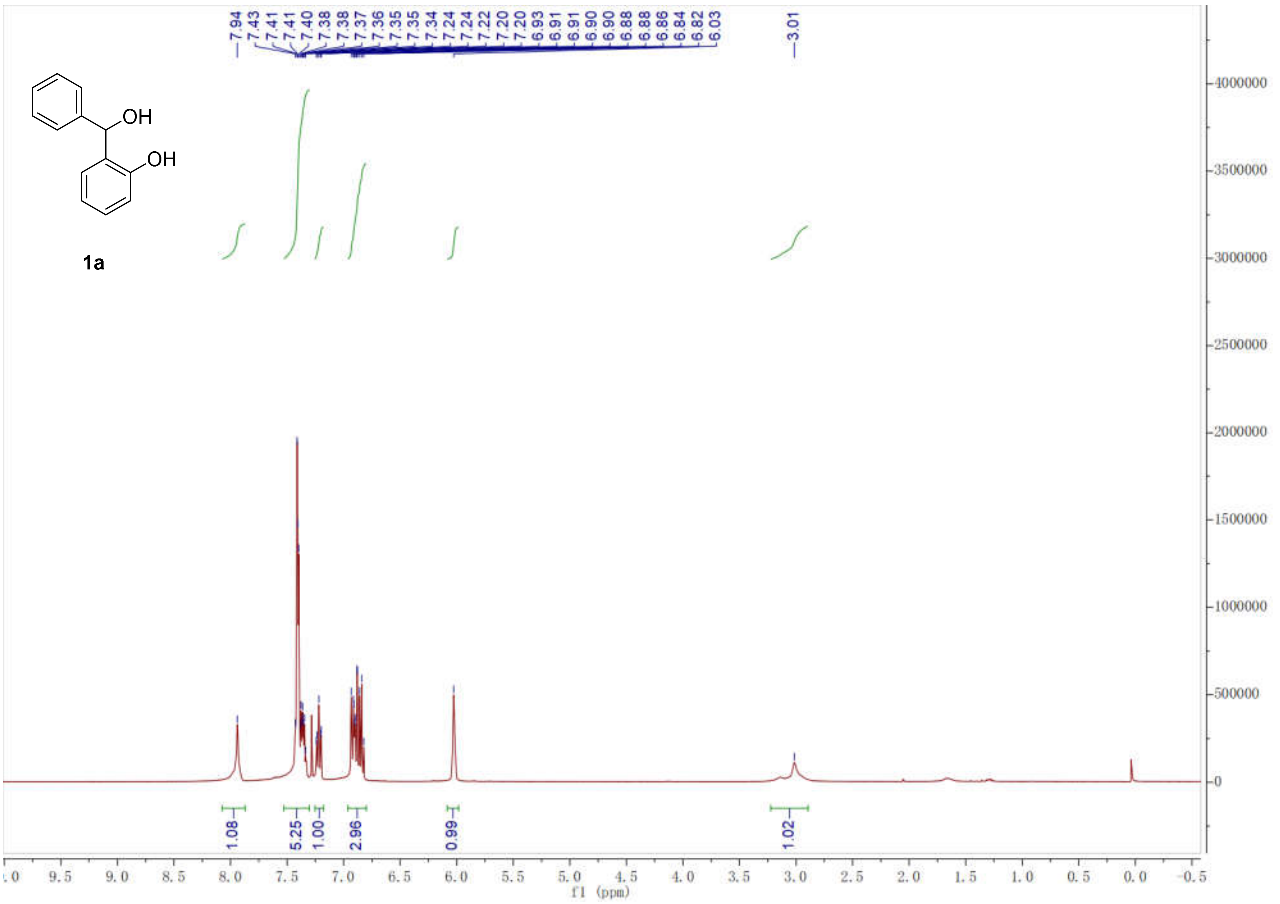

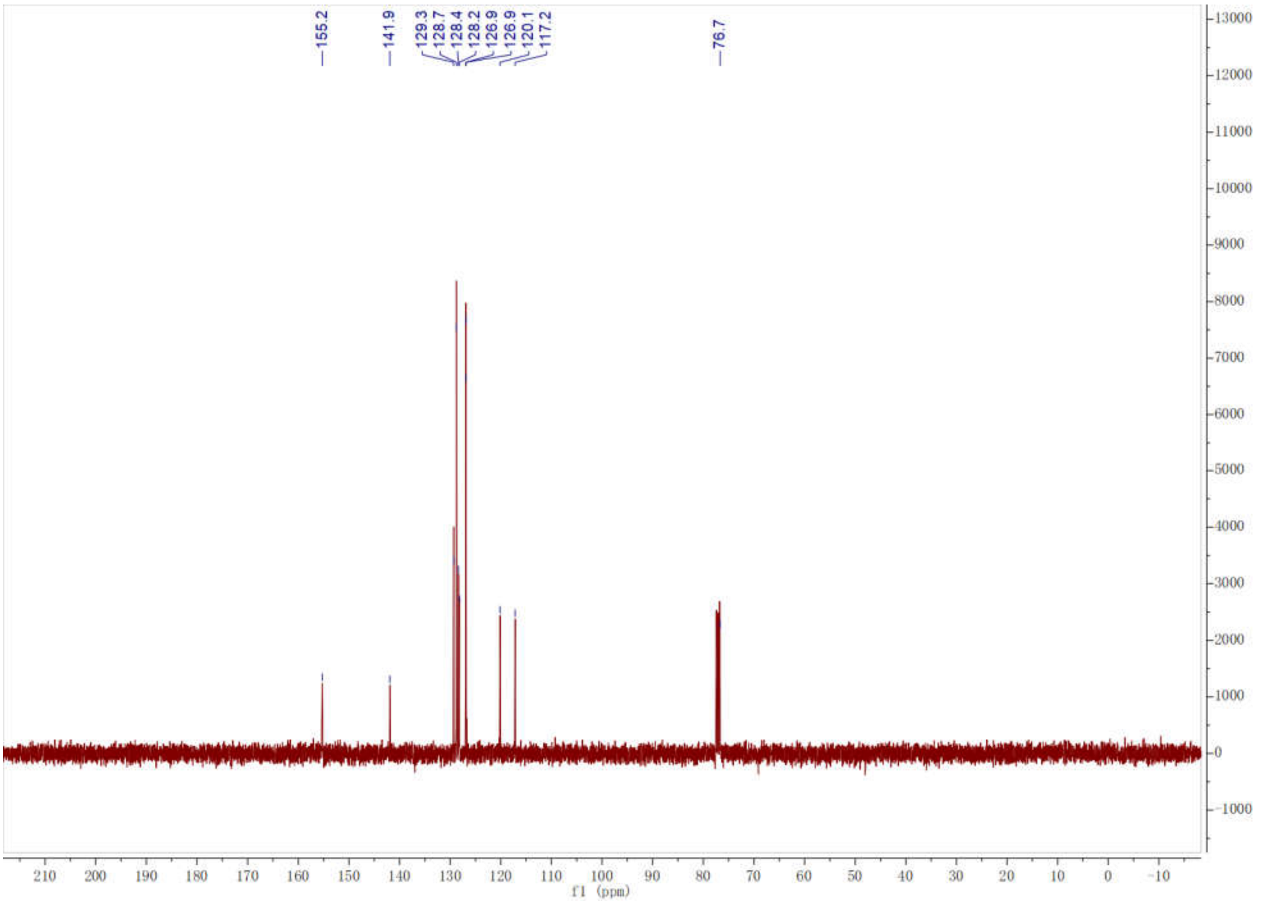

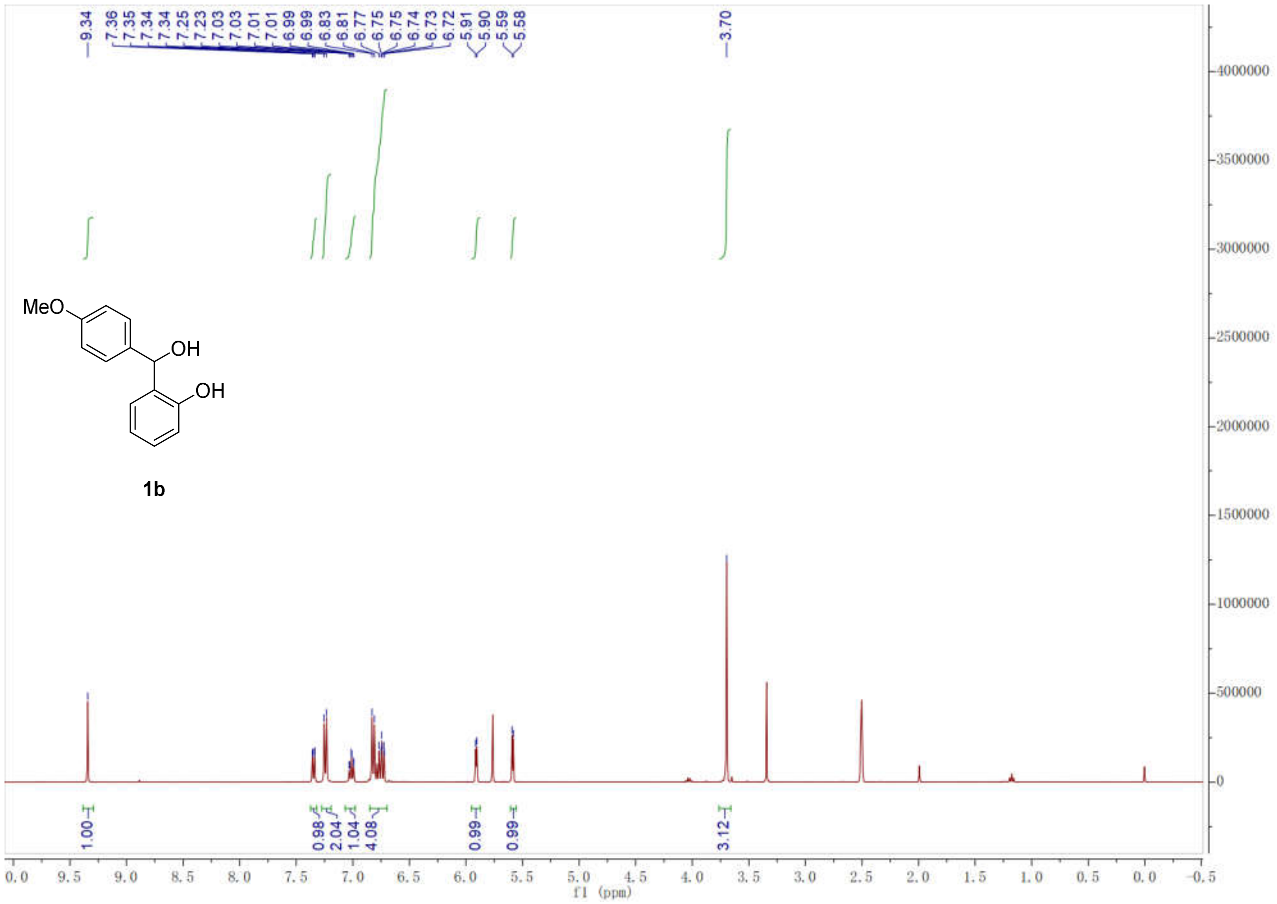
9.34
7.36
7.35
7.34
7.34
7.25
7.23
7.03
7.03
7.01
6.99
6.99
6.83
6.81
6.77
6.75
6.75
6.74
6.73
6.72
5.91
5.90
5.59
5.58
3.70
MeO
OH
OH
1b
1.00
0.98
2.04
1.04
4.08
0.99
0.99
3.12
f1 (ppm)

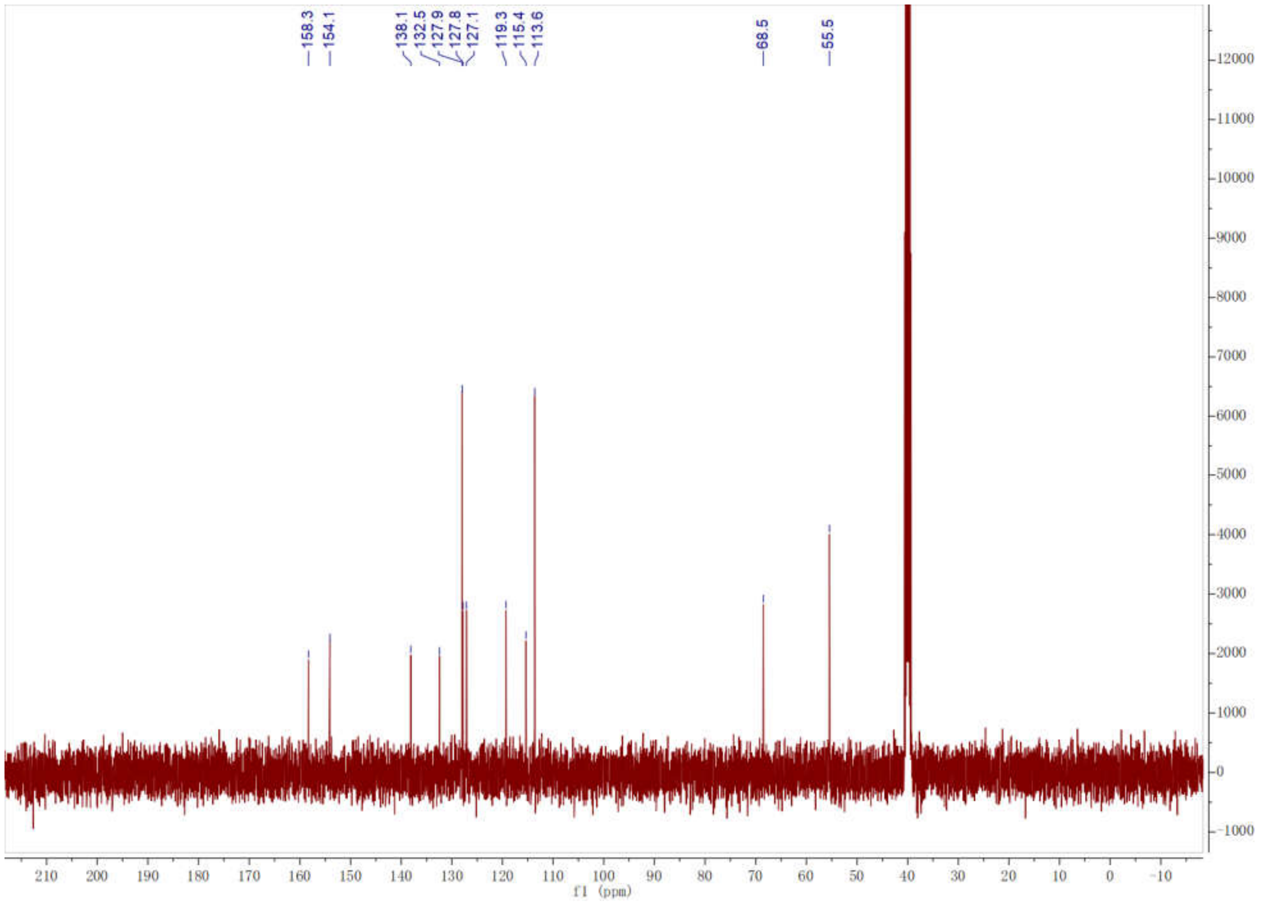
158.3
154.1
138.1
132.5
127.9
127.8
127.1
119.3
115.4
113.6
68.5
55.5
f1 (ppm)

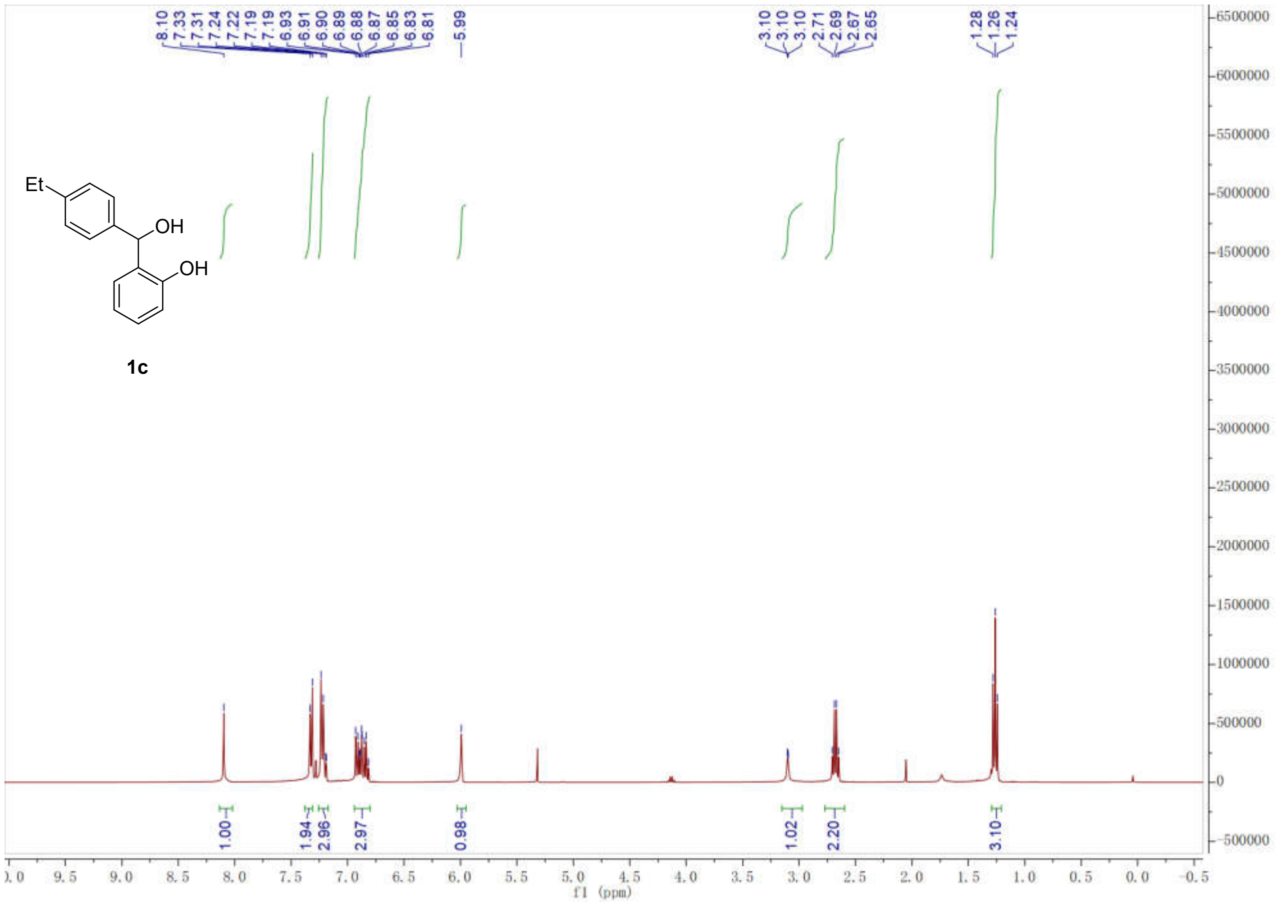
Et
OH
OH
1c
8.10
7.33
7.31
7.24
7.22
7.19
7.19
6.93
6.91
6.90
6.89
6.88
6.87
6.85
6.83
6.81
5.99
3.10
3.10
3.10
2.71
2.69
2.67
2.65
1.28
1.26
1.24
1.00
1.94
2.96
2.97
0.98
1.02
2.20
3.10
f1 (ppm)

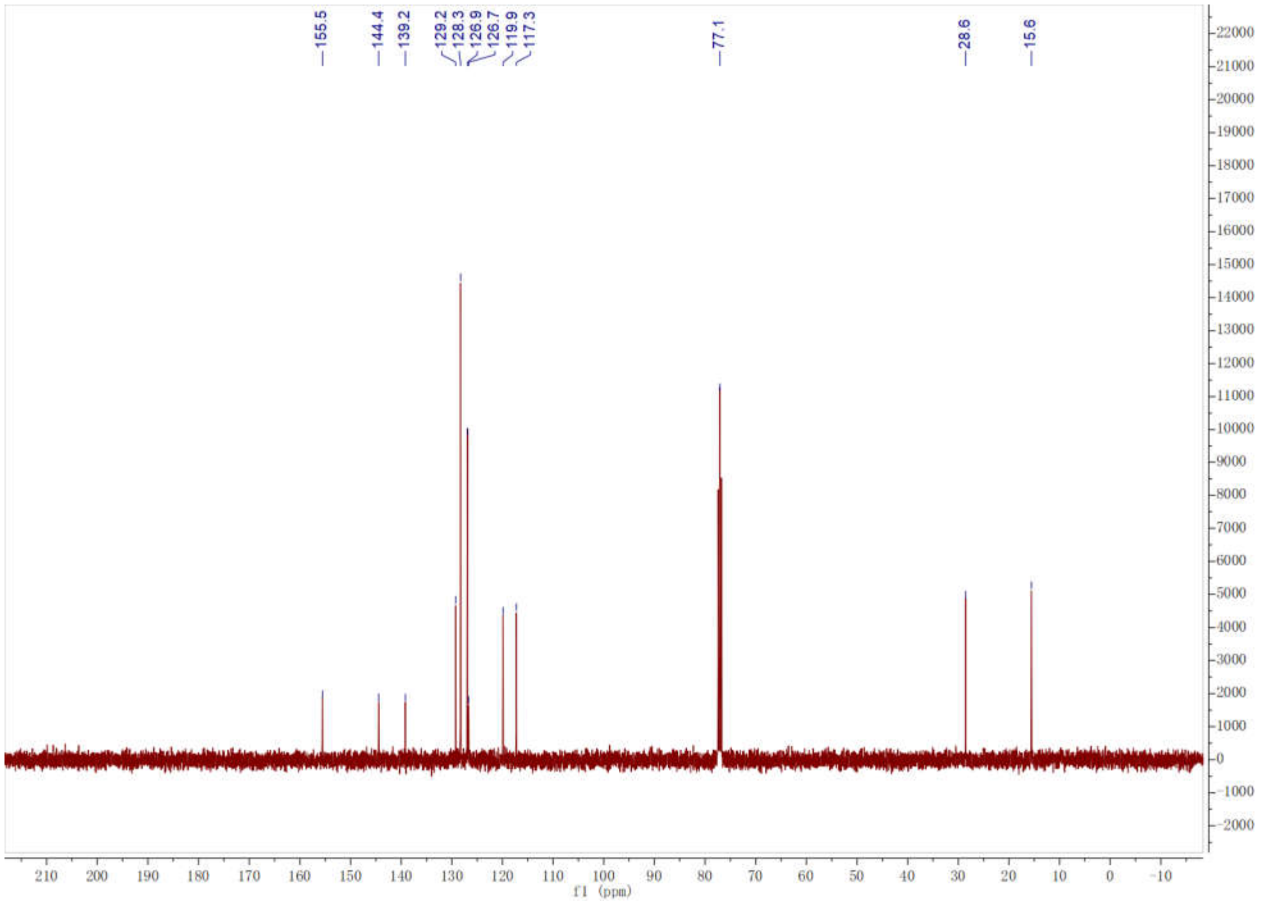
155.5
144.4
139.2
129.2
128.3
126.9
126.7
119.9
117.3
77.1
28.6
15.6
f1 (ppm)

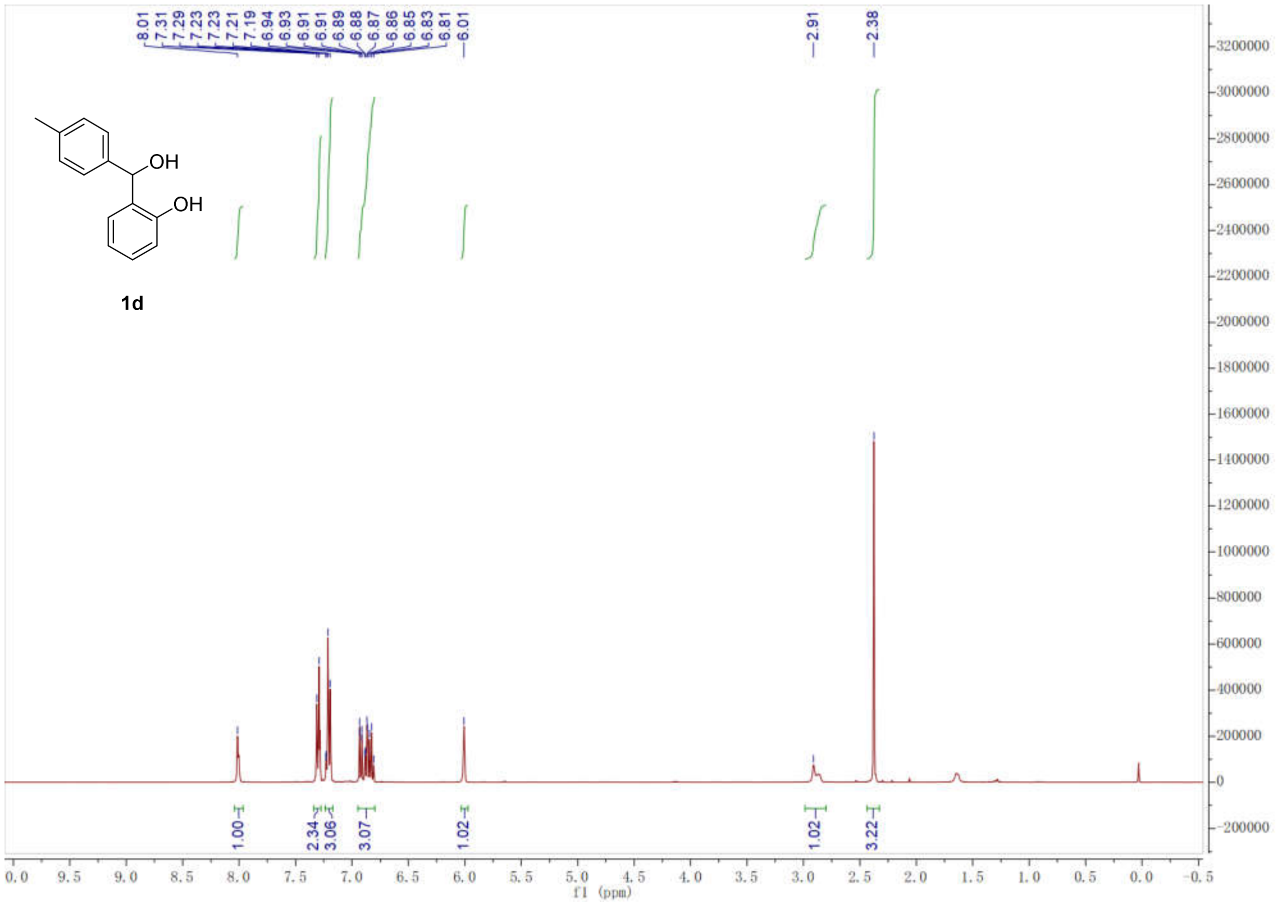
8.01
7.31
7.29
7.23
7.23
7.21
7.19
6.94
6.93
6.91
6.91
6.89
6.88
6.87
6.86
6.85
6.83
6.81
6.01
2.91
2.38
OH
OH
1d
1.00
2.34
3.06
3.07
1.02
1.02
3.22
f1 (ppm)

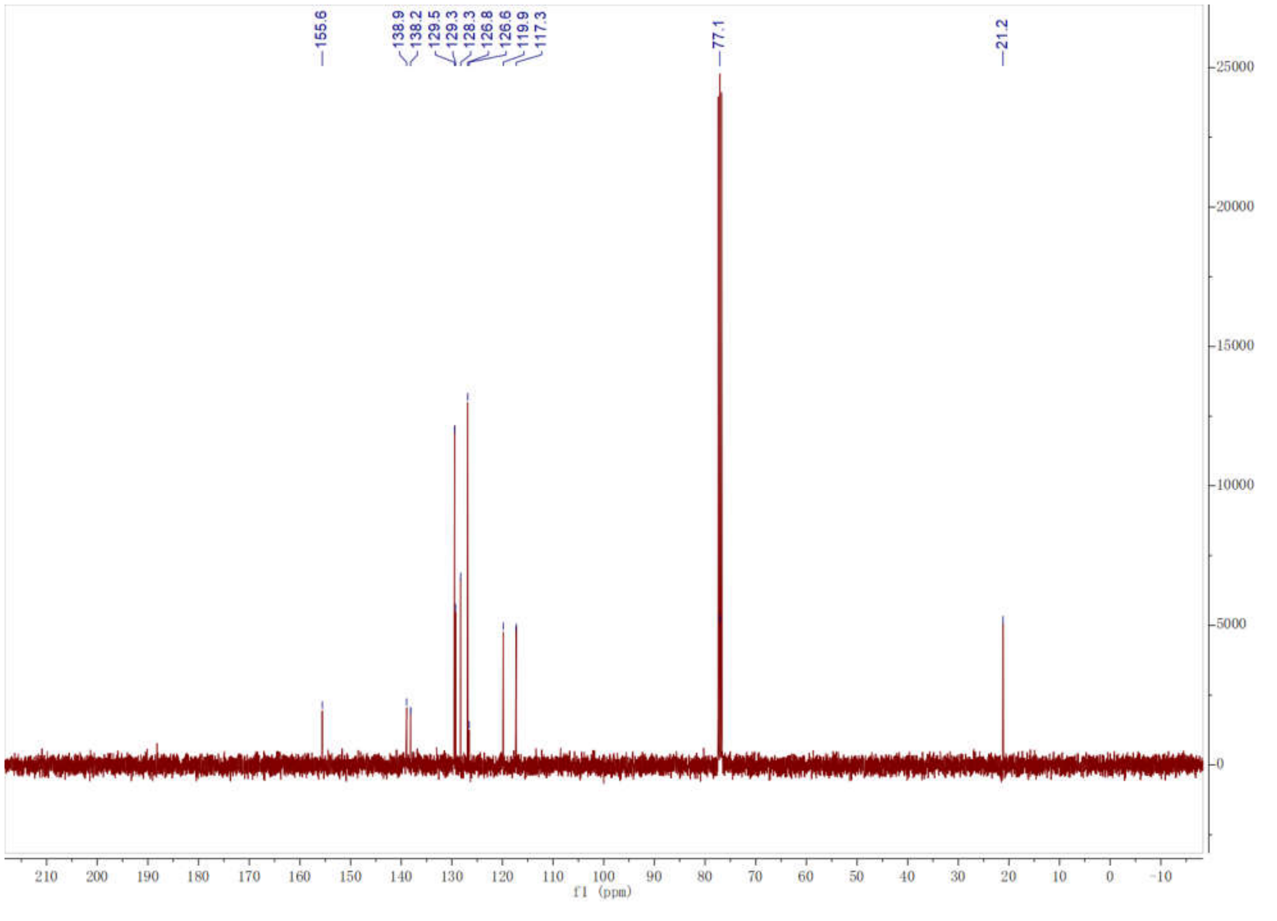
155.6
138.9
138.2
129.5
129.3
128.3
126.8
126.6
119.9
117.3
77.1
21.2
f1 (ppm)

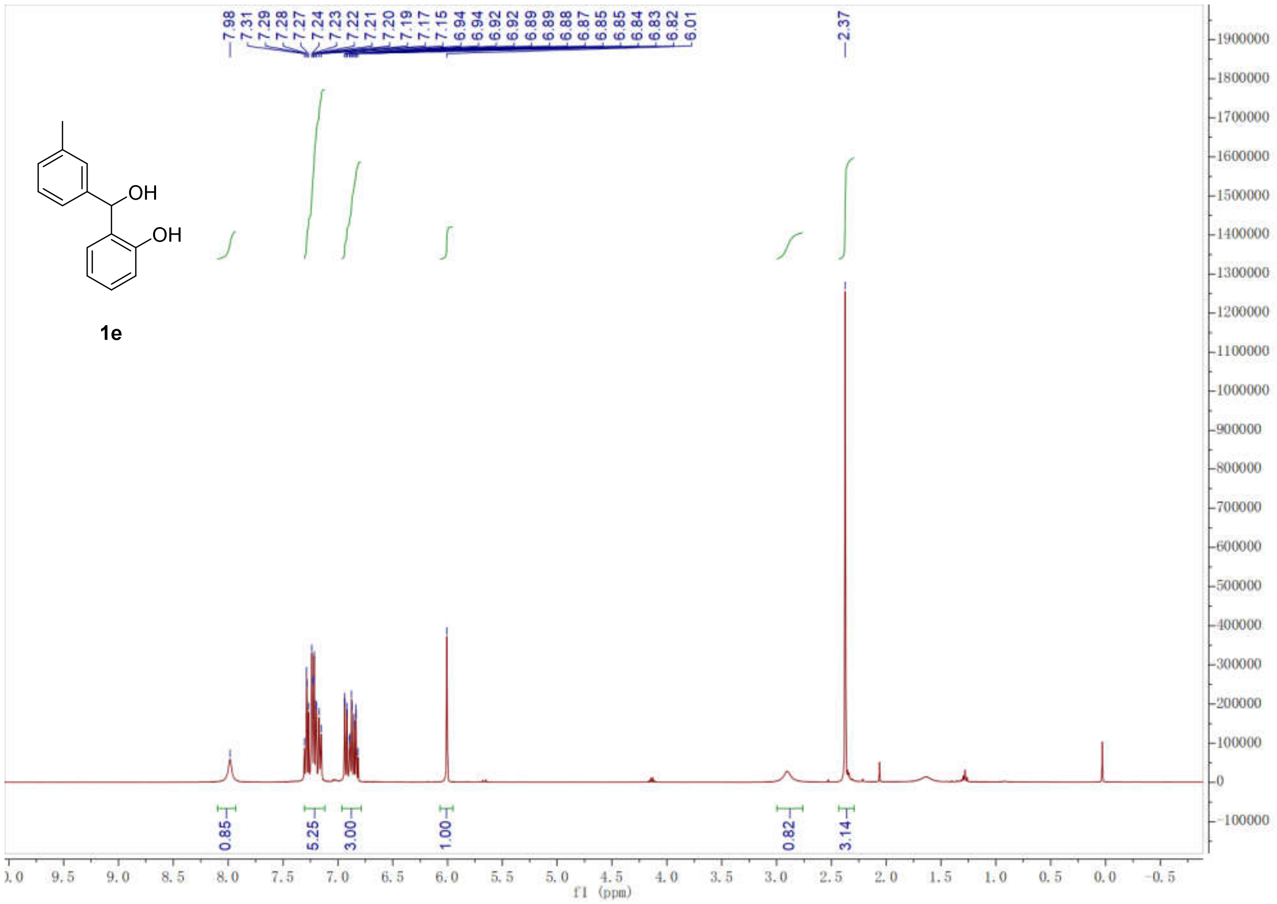
7.98
7.31 7.29 7.28 7.27 7.24 7.23 7.22 7.21 7.20 7.19 7.17 7.15 6.94 6.94 6.92 6.92 6.89 6.89 6.88 6.87 6.85 6.85 6.84 6.83 6.82 6.01
2.37
OH
OH
1e
0.85
5.25
3.00
1.00
0.82
3.14
f1 (ppm)

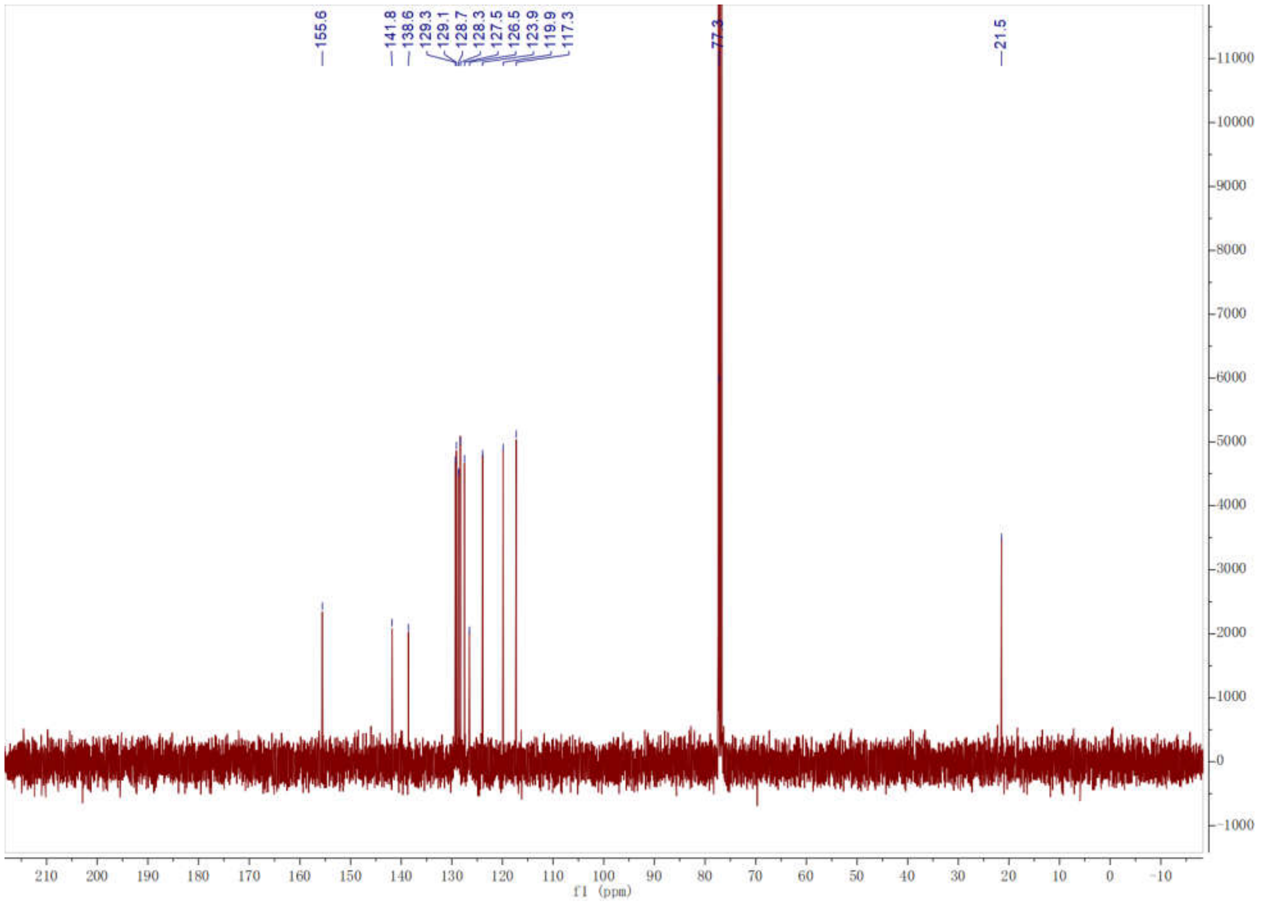
155.6
141.8 138.6 129.3 129.1 128.7 128.3 127.5 126.5 123.9 119.9 117.3
77.3
21.5
f1 (ppm)

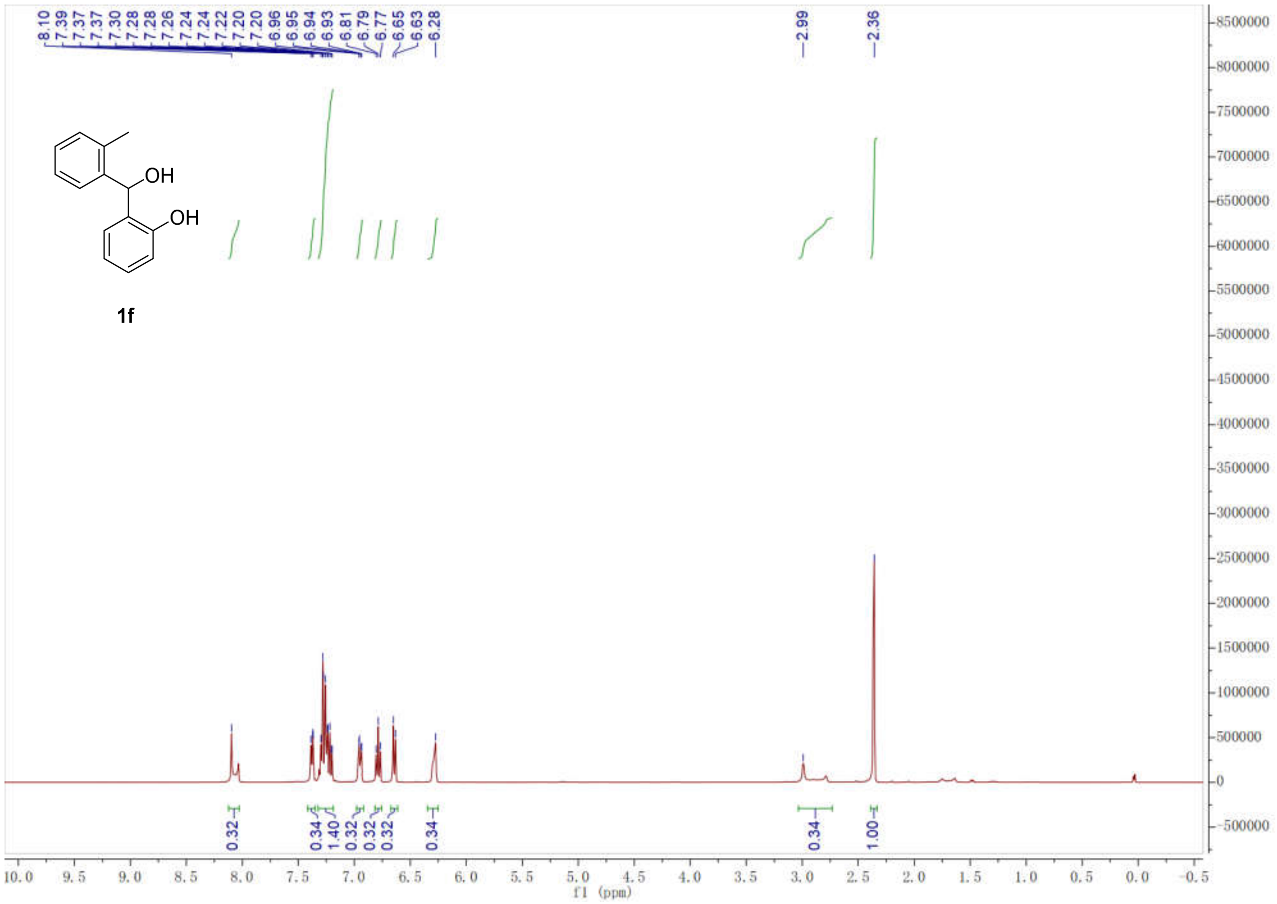
8.10 7.39 7.37 7.37 7.30 7.28 7.28 7.26 7.24 7.24 7.22 7.20 7.20 6.96 6.95 6.94 6.93 6.81 6.79 6.77 6.65 6.63 6.28
2.99
2.36
OH
OH
1f
0.32 0.34 1.40 0.32 0.32 0.32 0.34 0.34 1.00
f1 (ppm)

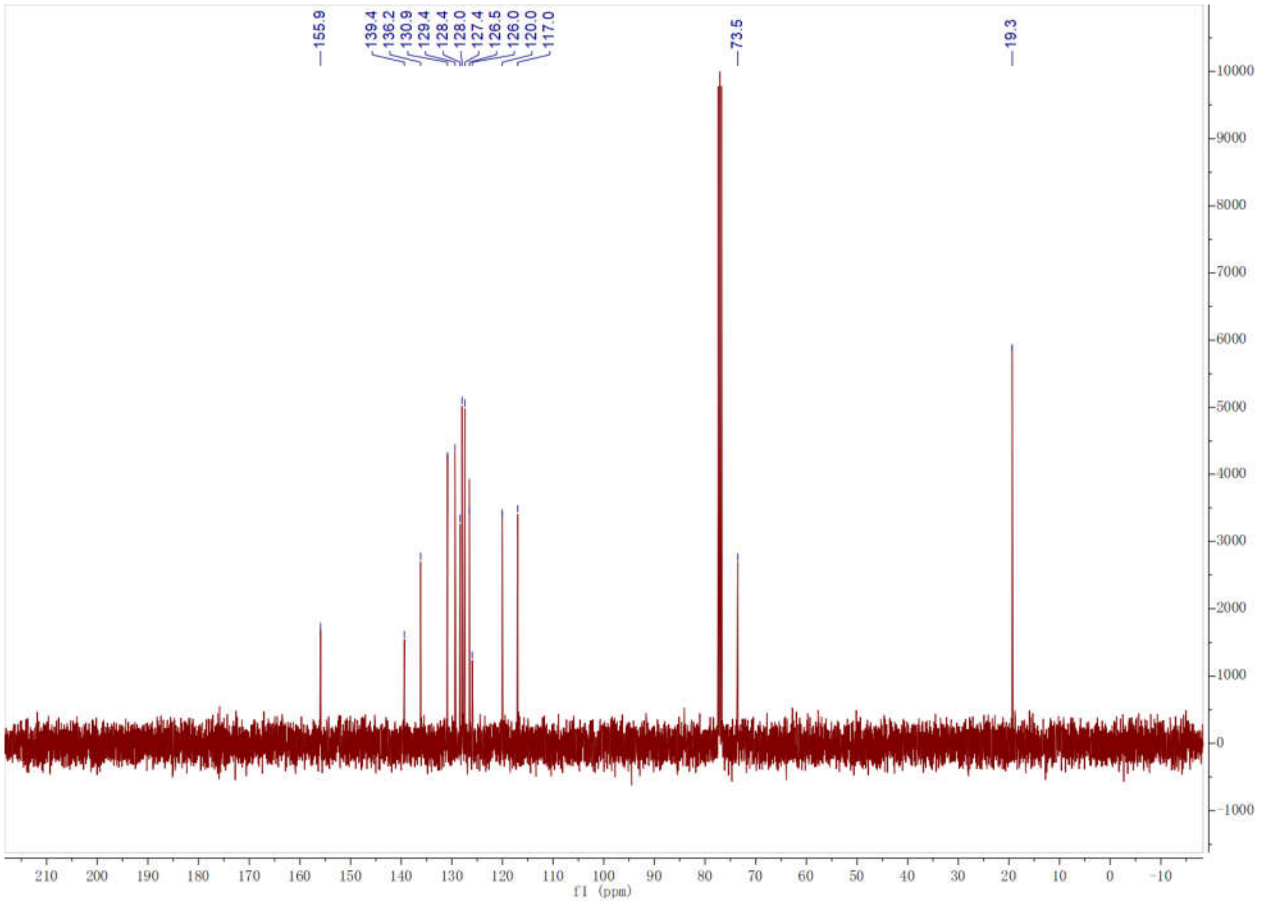
155.9
139.4 136.2 130.9 129.4 128.4 128.0 127.4 126.5 126.0 120.0 117.0
73.5
19.3
f1 (ppm)

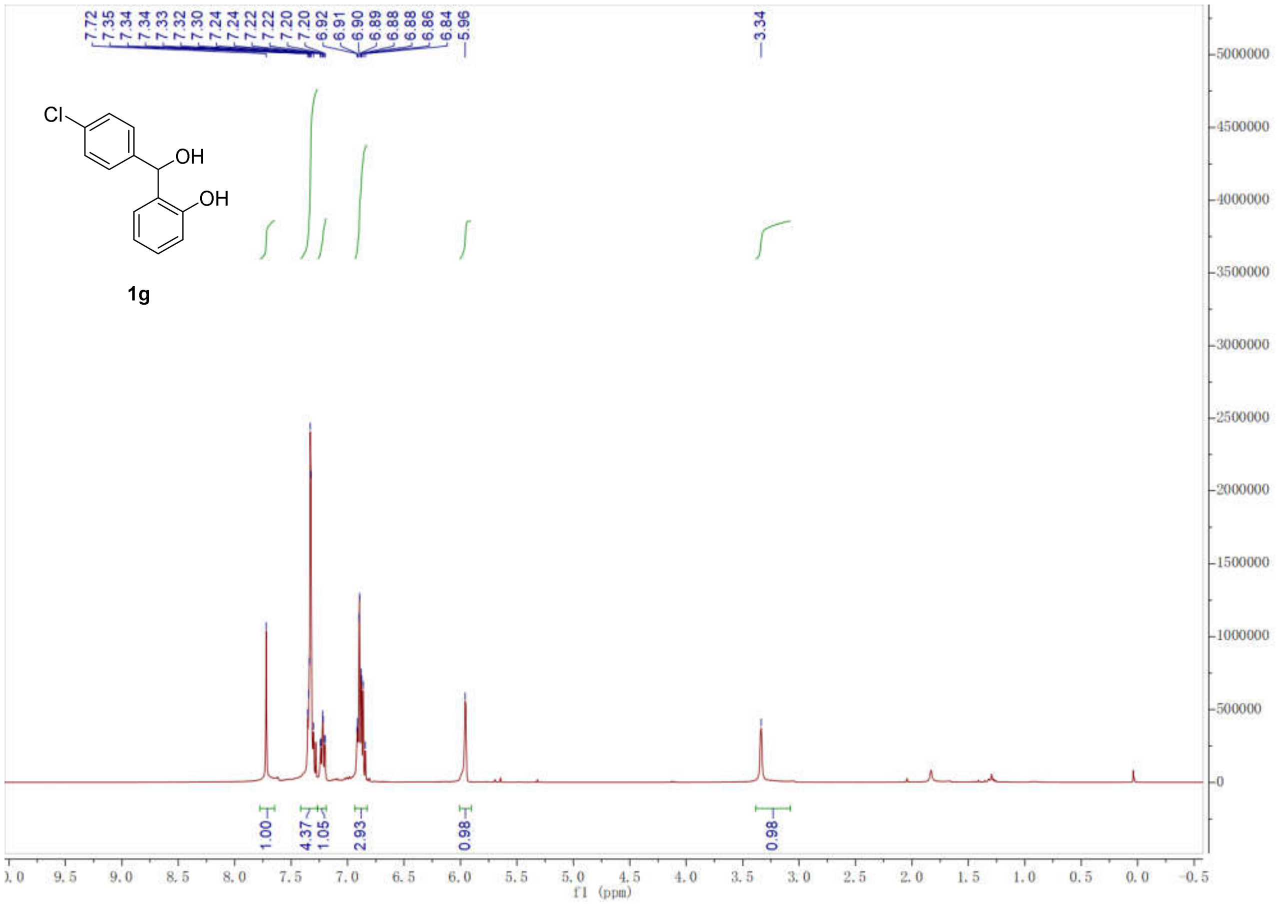
1g
7.72
7.35
7.34
7.34
7.33
7.32
7.30
7.24
7.24
7.22
7.22
7.20
7.20
6.92
6.91
6.90
6.89
6.88
6.88
6.86
6.84
5.96
3.34
1.00
4.37
1.05
2.93
0.98
0.98
f1 (ppm)

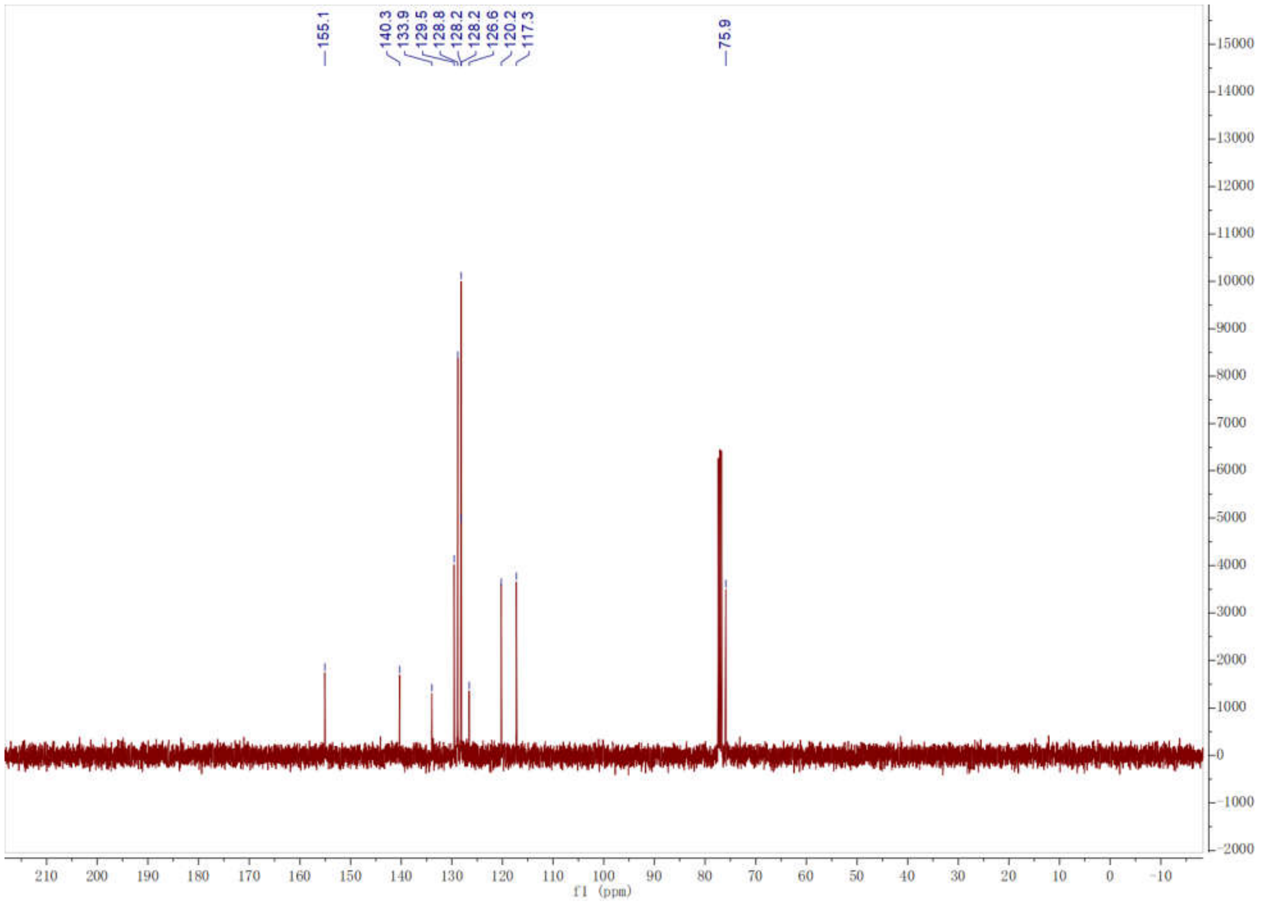
155.1
140.3
133.9
129.5
128.8
128.2
128.2
126.6
120.2
117.3
75.9
f1 (ppm)

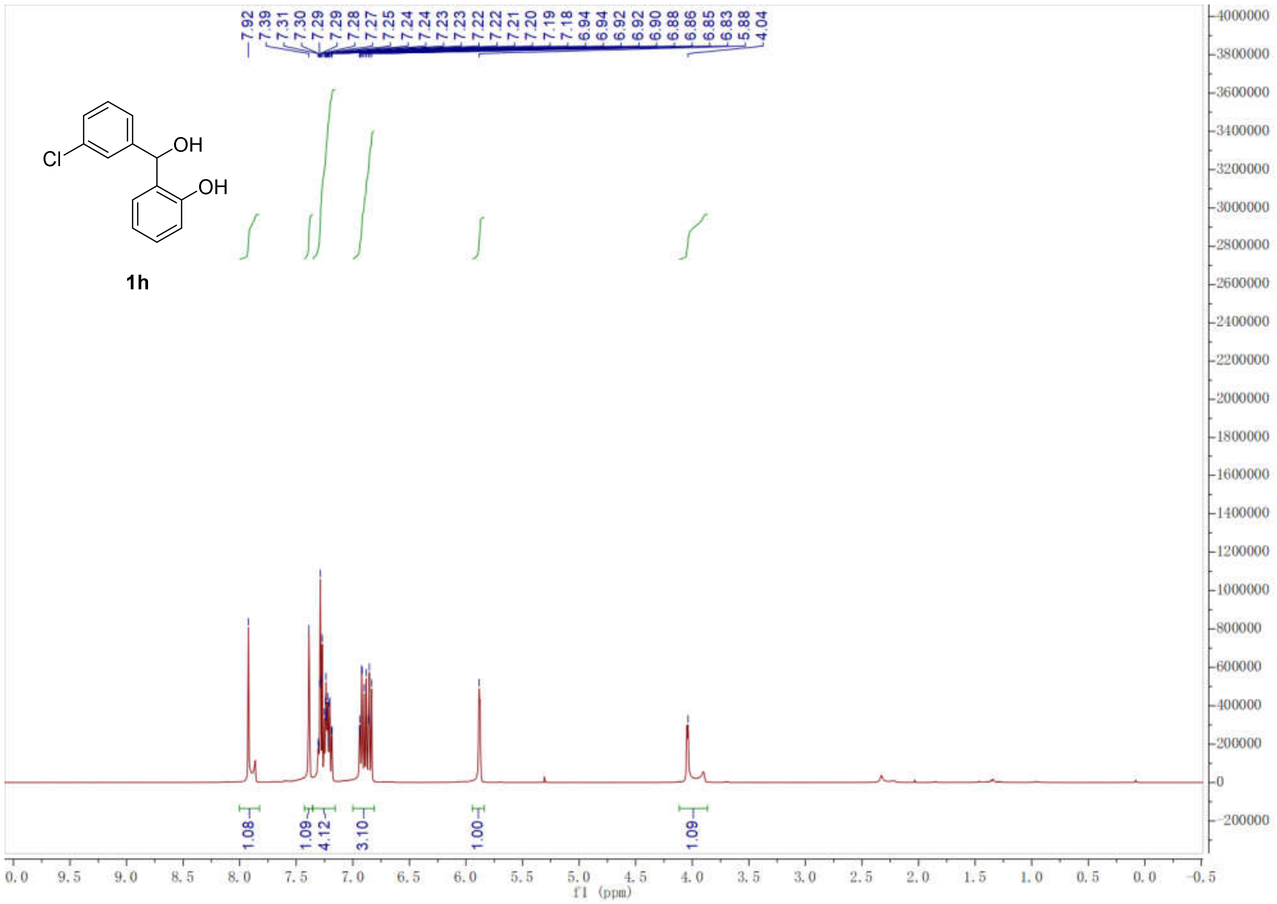

Cl
OH
OH
1h
7.92
7.39
7.31
7.30
7.29
7.29
7.28
7.27
7.25
7.24
7.24
7.23
7.23
7.22
7.22
7.21
7.20
7.19
7.18
6.94
6.94
6.92
6.92
6.90
6.88
6.86
6.85
6.83
5.88
4.04
1.08
1.09
4.12
3.10
1.00
1.09
f1 (ppm)

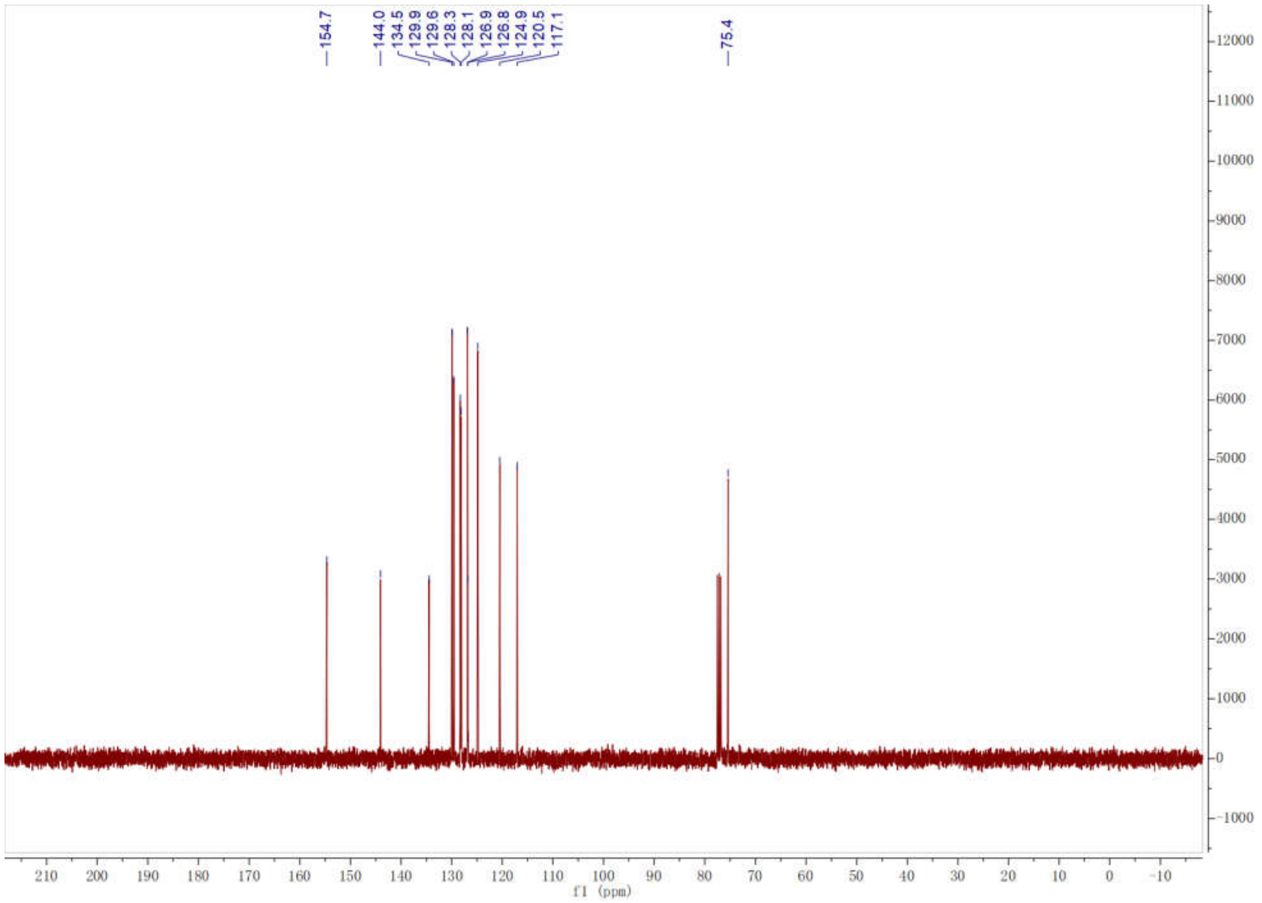

154.7
144.0
134.5
129.9
129.6
128.3
128.1
126.9
126.8
124.9
120.5
117.1
75.4
f1 (ppm)

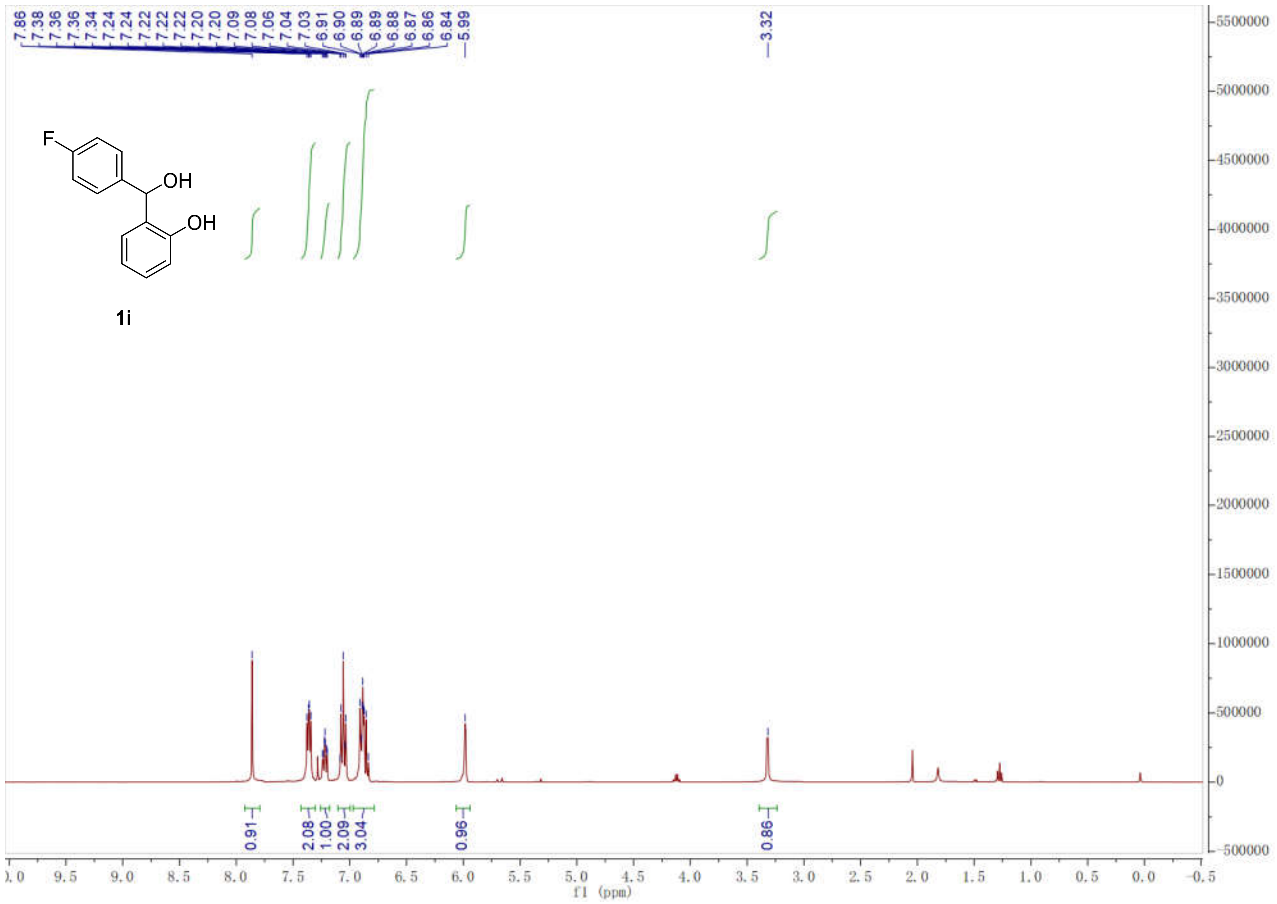

7.86
7.38
7.36
7.36
7.34
7.24
7.24
7.22
7.22
7.22
7.20
7.09
7.08
7.06
7.04
7.03
6.91
6.90
6.89
6.89
6.88
6.87
6.86
6.84
5.99
3.32
F
OH
OH
1i
0.91
2.08
1.00
2.09
3.04
0.96
0.86
f1 (ppm)

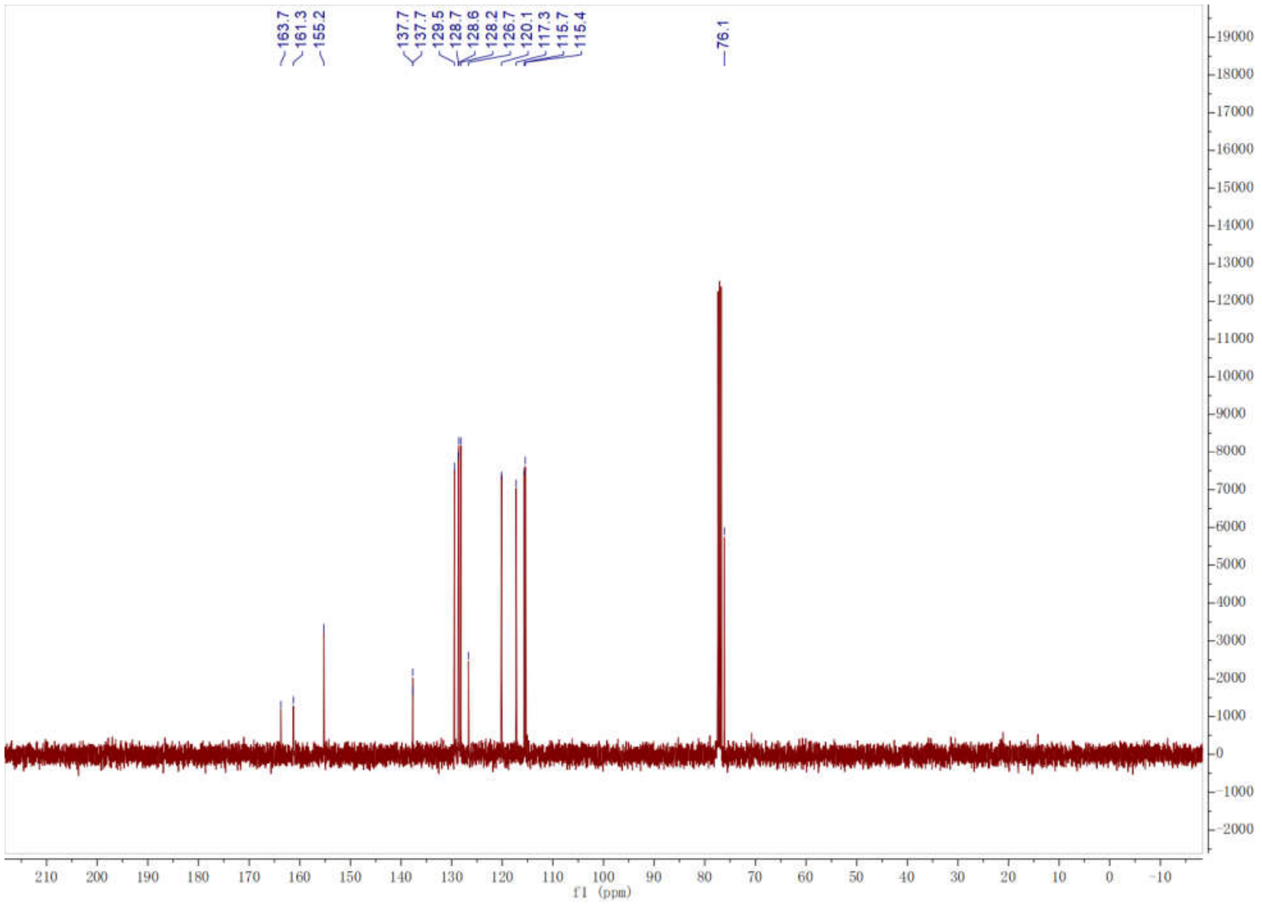
163.7
161.3
155.2
137.7
137.7
129.5
128.7
128.6
128.2
126.7
120.1
117.3
115.7
115.4
76.1
f1 (ppm)

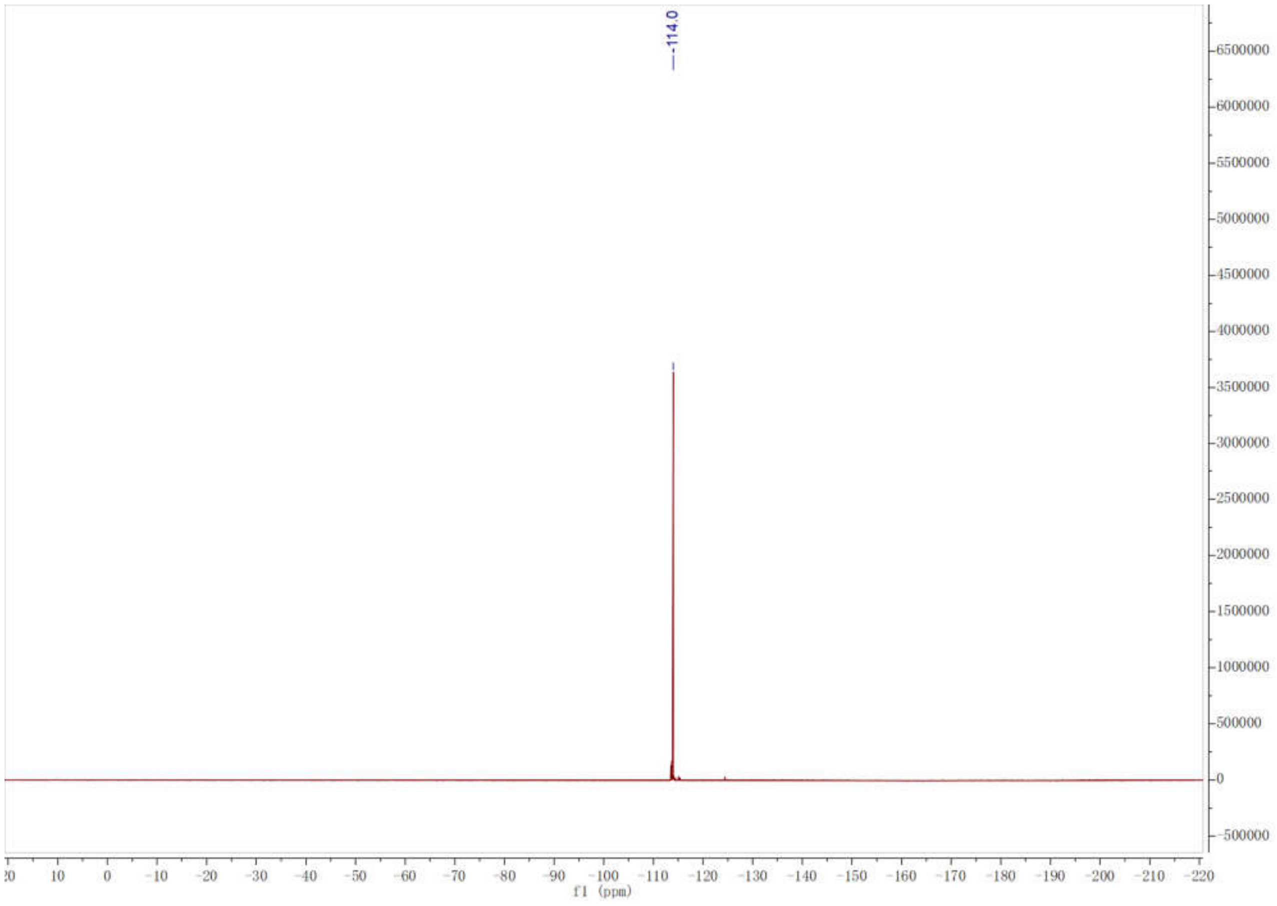
-114.0
f1 (ppm)

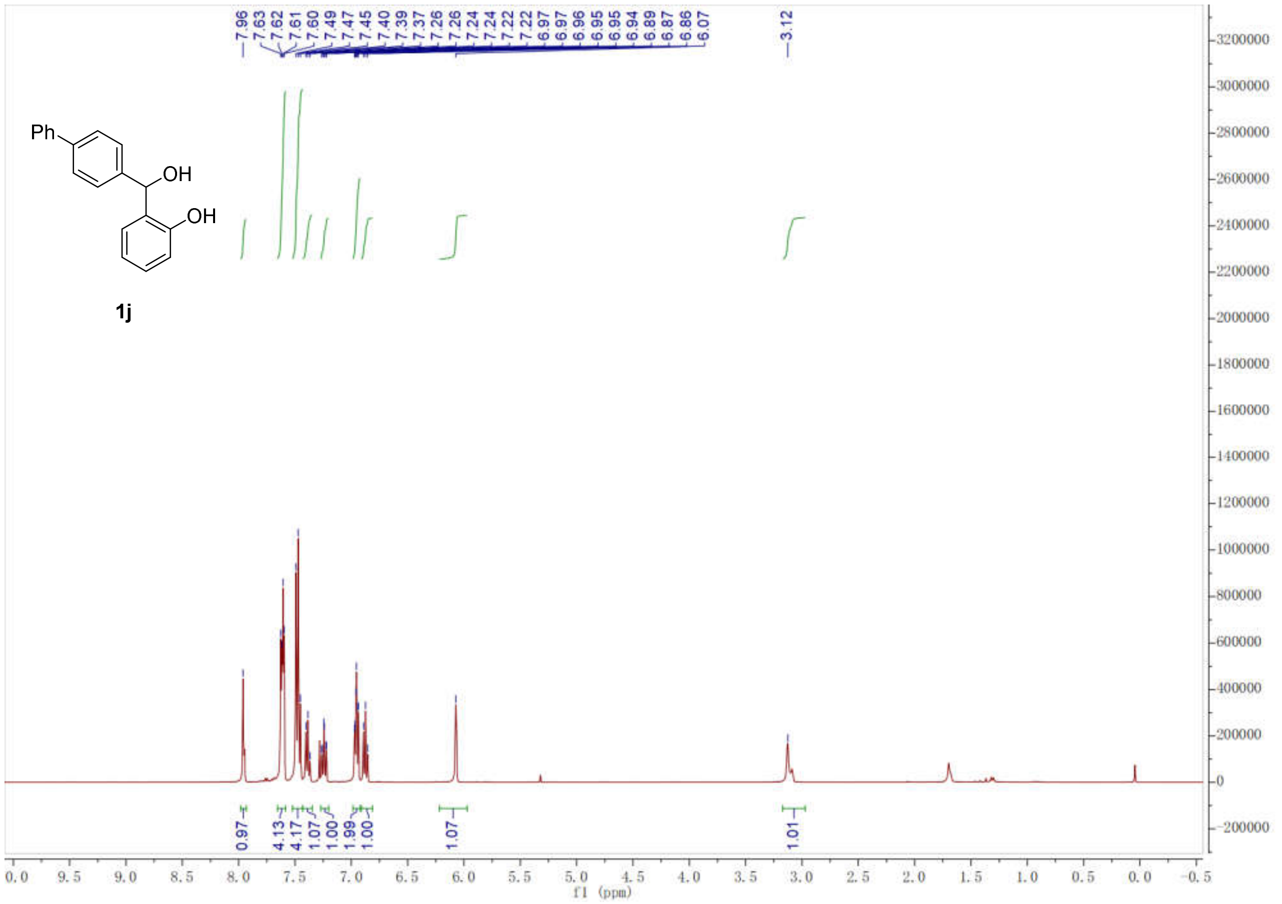
Ph
OH
OH
1j
f1 (ppm)

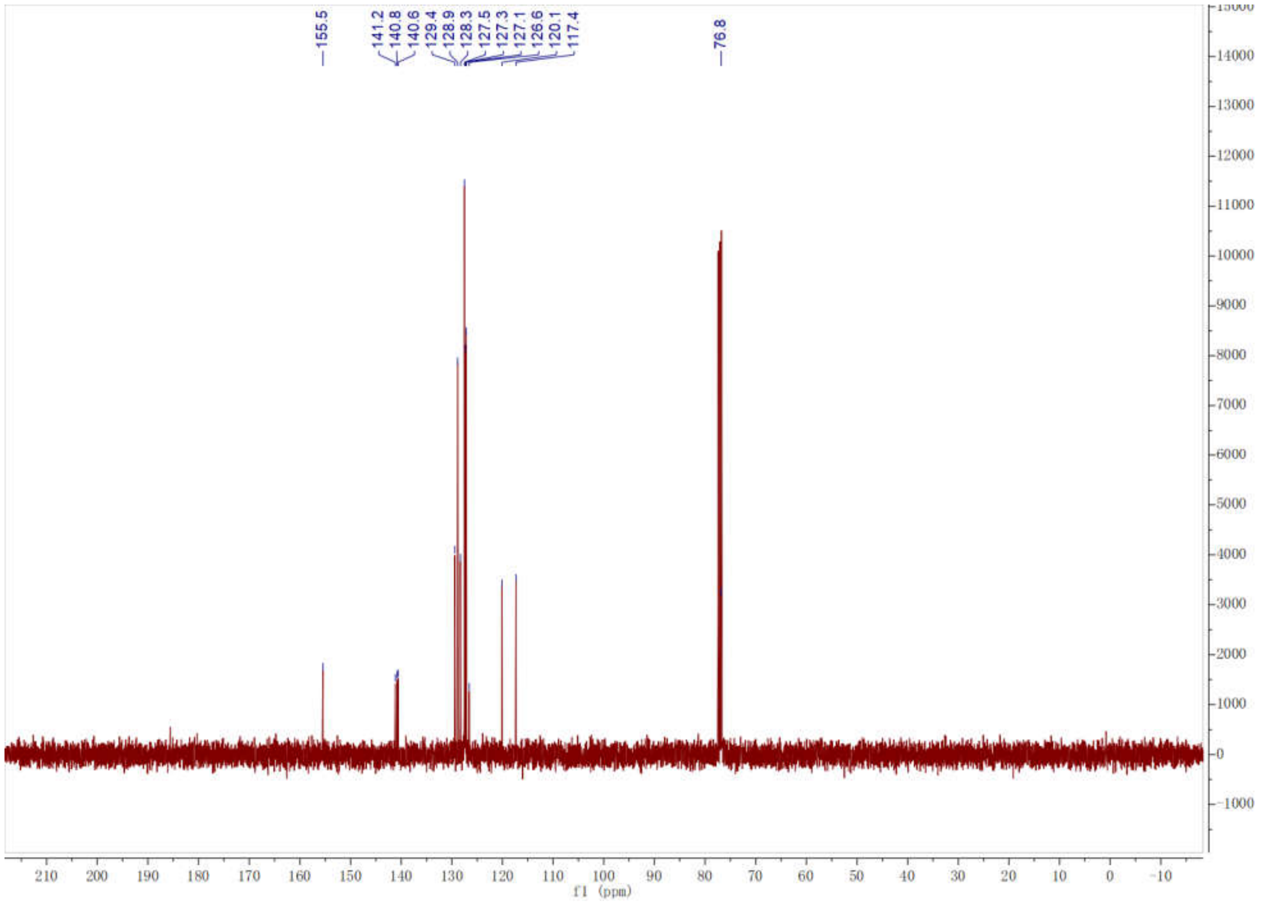
f1 (ppm)

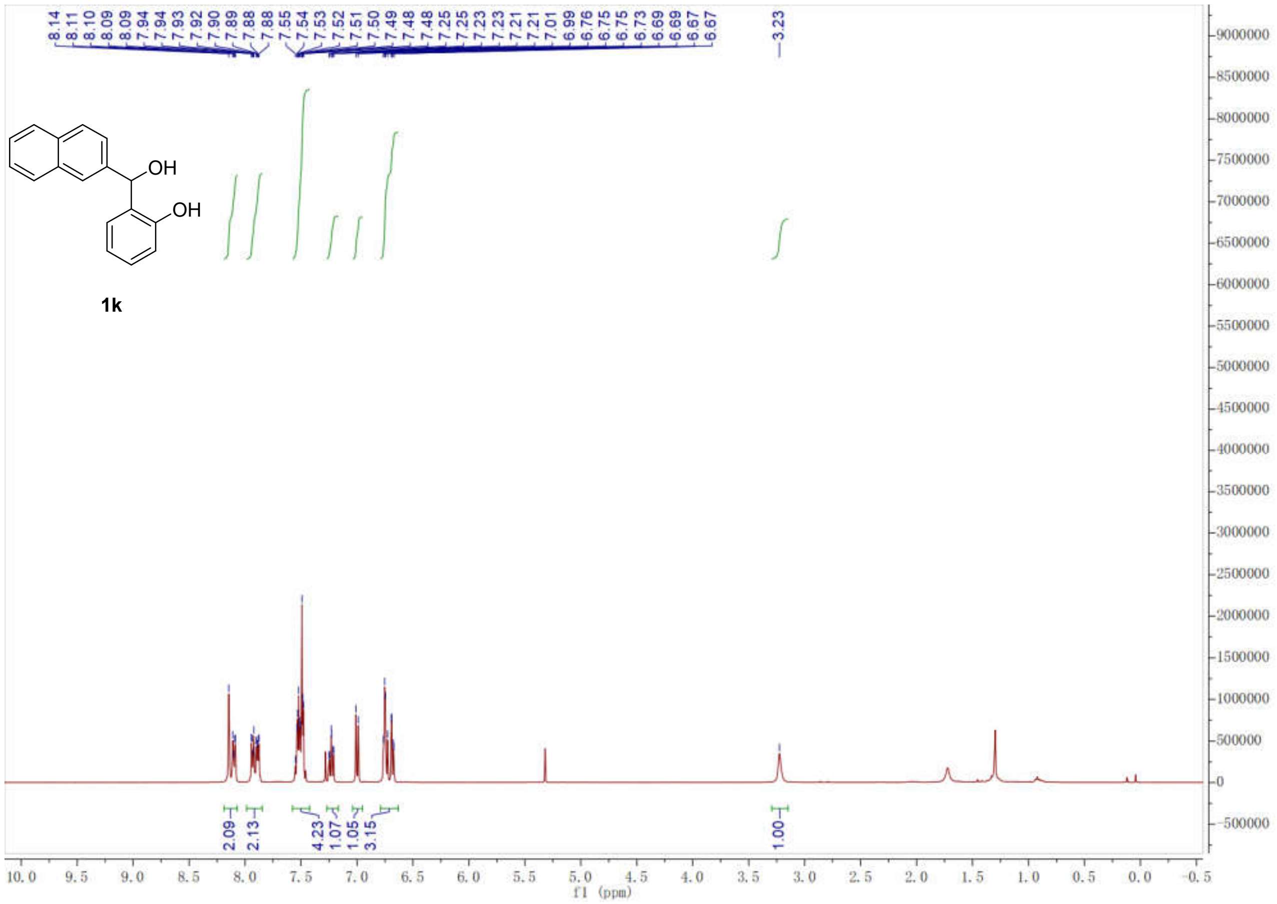

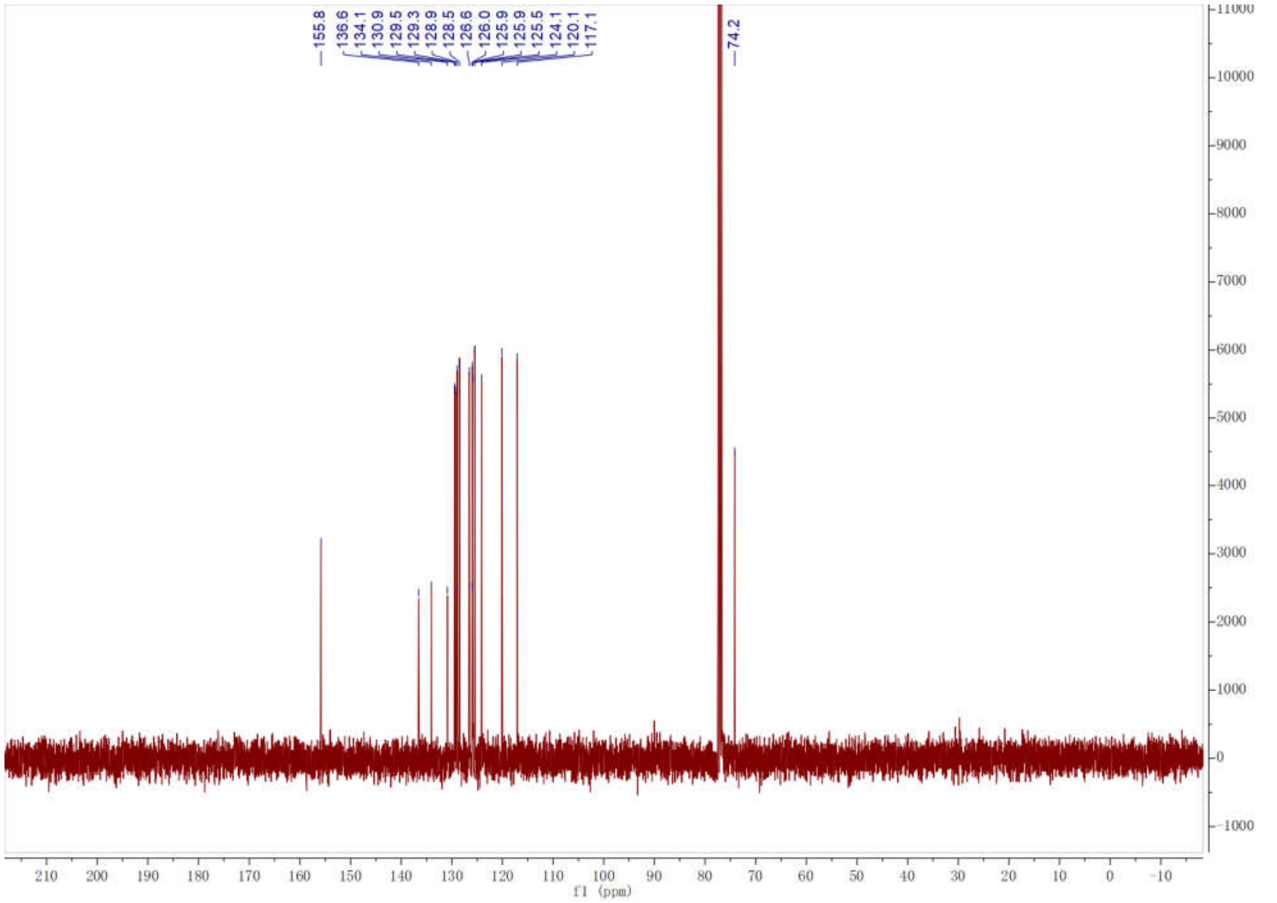

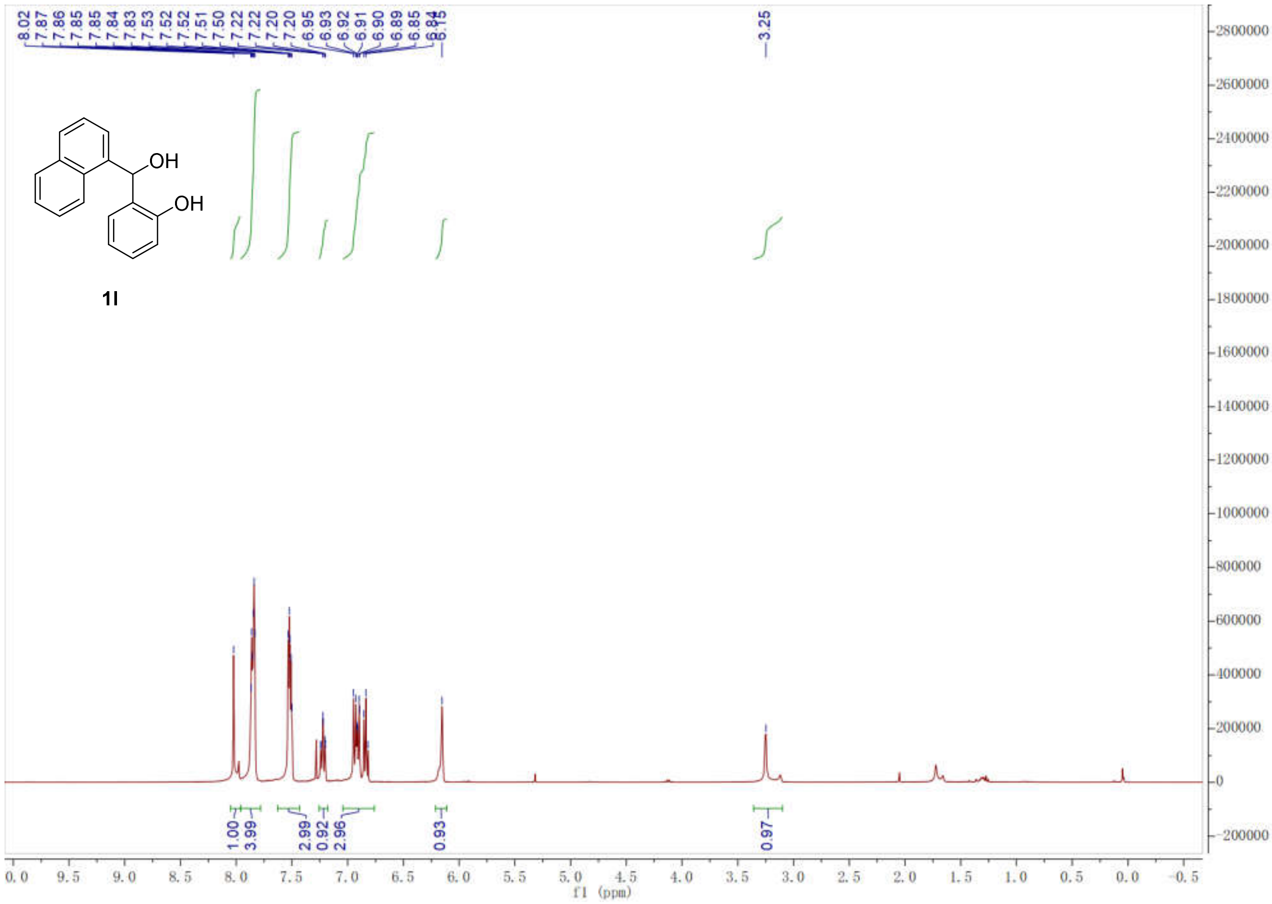
8.02
7.87
7.86
7.85
7.84
7.83
7.53
7.52
7.52
7.51
7.50
7.22
7.22
7.20
7.20
6.95
6.93
6.92
6.91
6.90
6.89
6.85
6.84
6.15
3.25
OH
OH
1l
1.00
3.99
2.99
0.92
2.96
0.93
0.97
f1 (ppm)

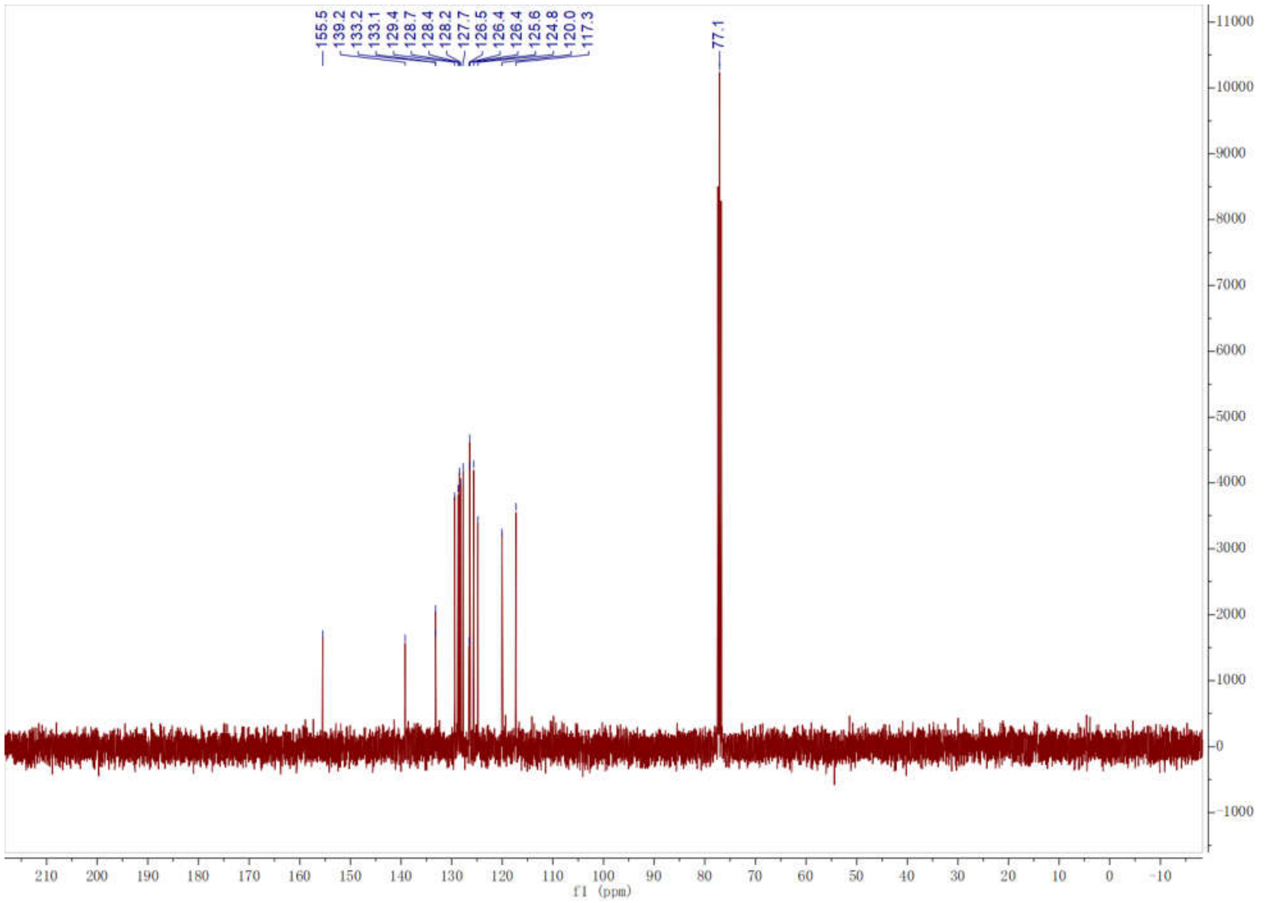
155.5
139.2
133.2
133.1
129.4
128.7
128.4
128.2
127.7
126.5
126.4
126.4
125.6
124.8
120.0
117.3
77.1
f1 (ppm)

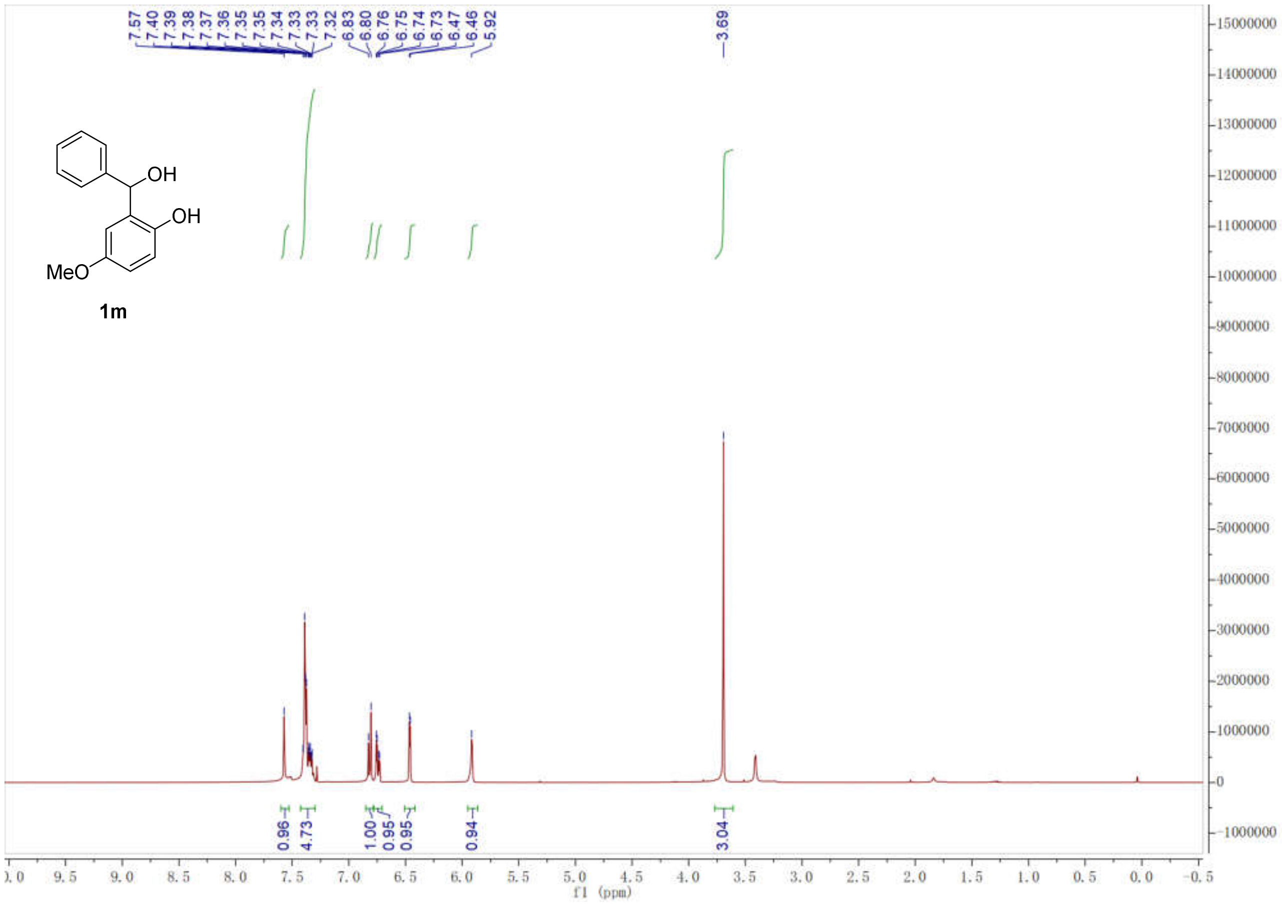

OH
OH
MeO
1m
7.57
7.40
7.39
7.38
7.37
7.36
7.35
7.35
7.34
7.33
7.33
7.32
6.83
6.80
6.76
6.75
6.74
6.73
6.47
6.46
5.92
3.69
0.96
4.73
1.00
0.95
0.95
0.94
3.04
f1 (ppm)

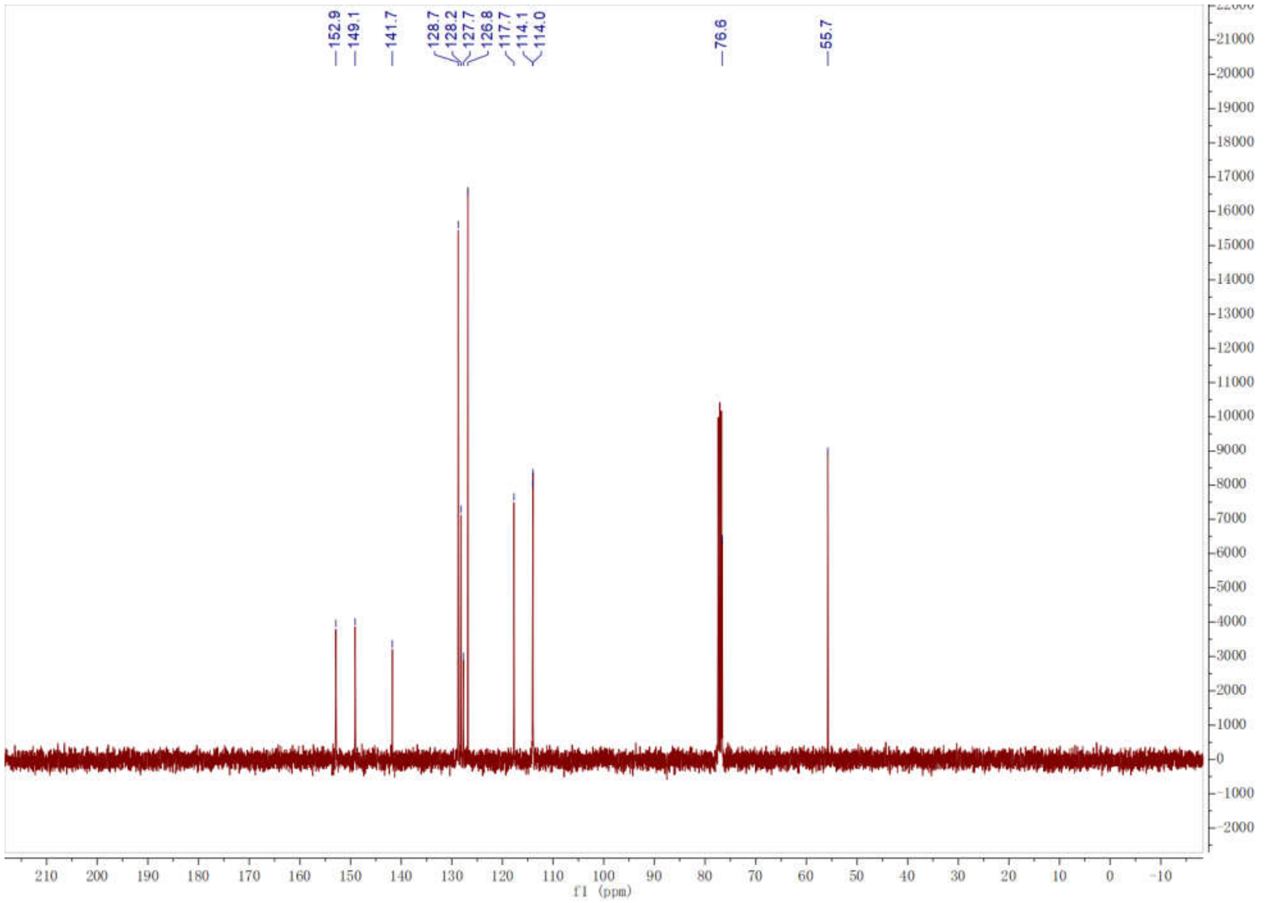

152.9
149.1
141.7
128.7
128.2
127.7
126.8
117.7
114.1
114.0
76.6
55.7
f1 (ppm)

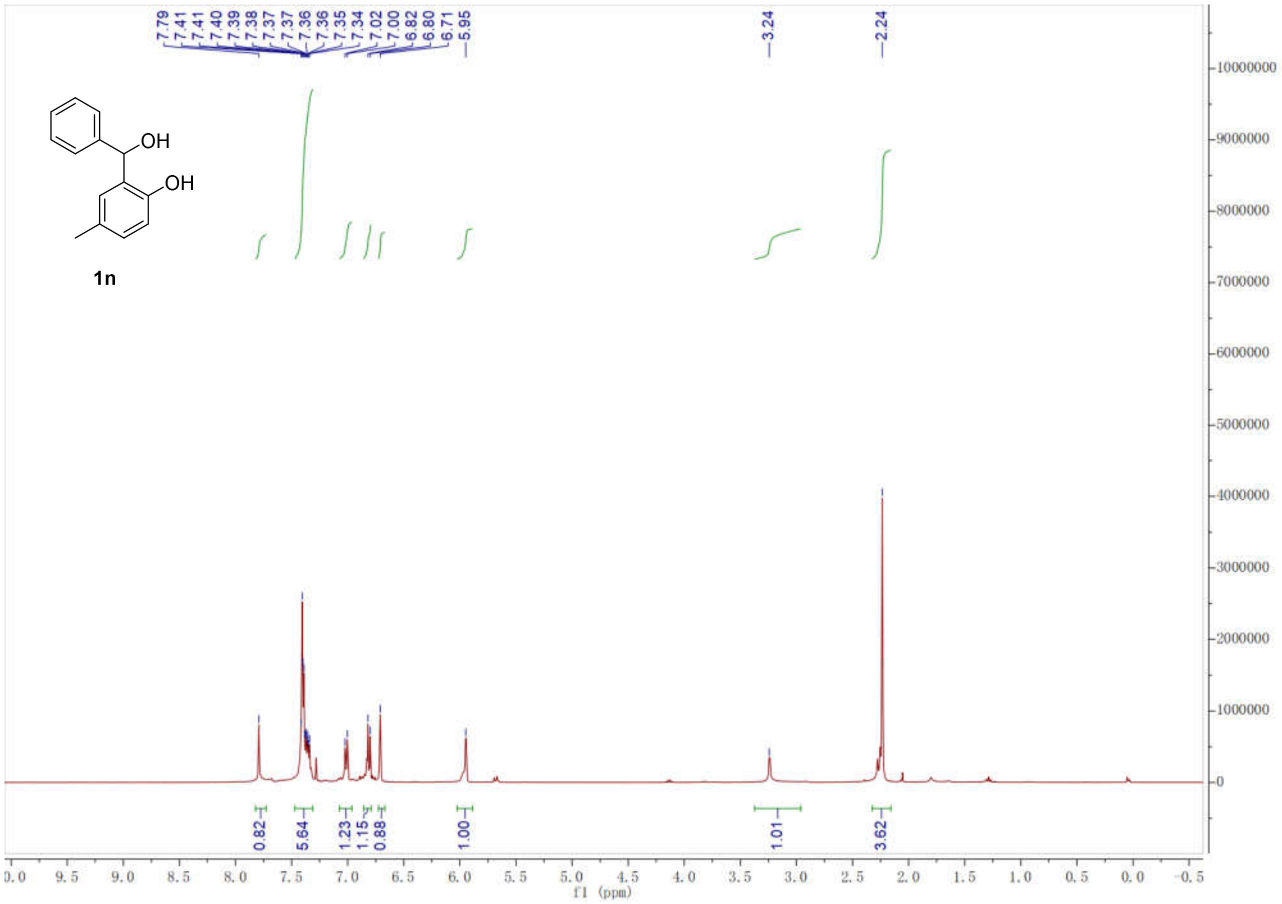
7.79 7.41 7.41 7.40 7.39 7.38 7.37 7.37 7.36 7.36 7.35 7.34 7.02 7.00 6.82 6.80 6.71 5.95
3.24
2.24
OH
OH
1n
0.82 5.64 1.23 1.15 0.88 1.00 1.01 3.62
f1 (ppm)

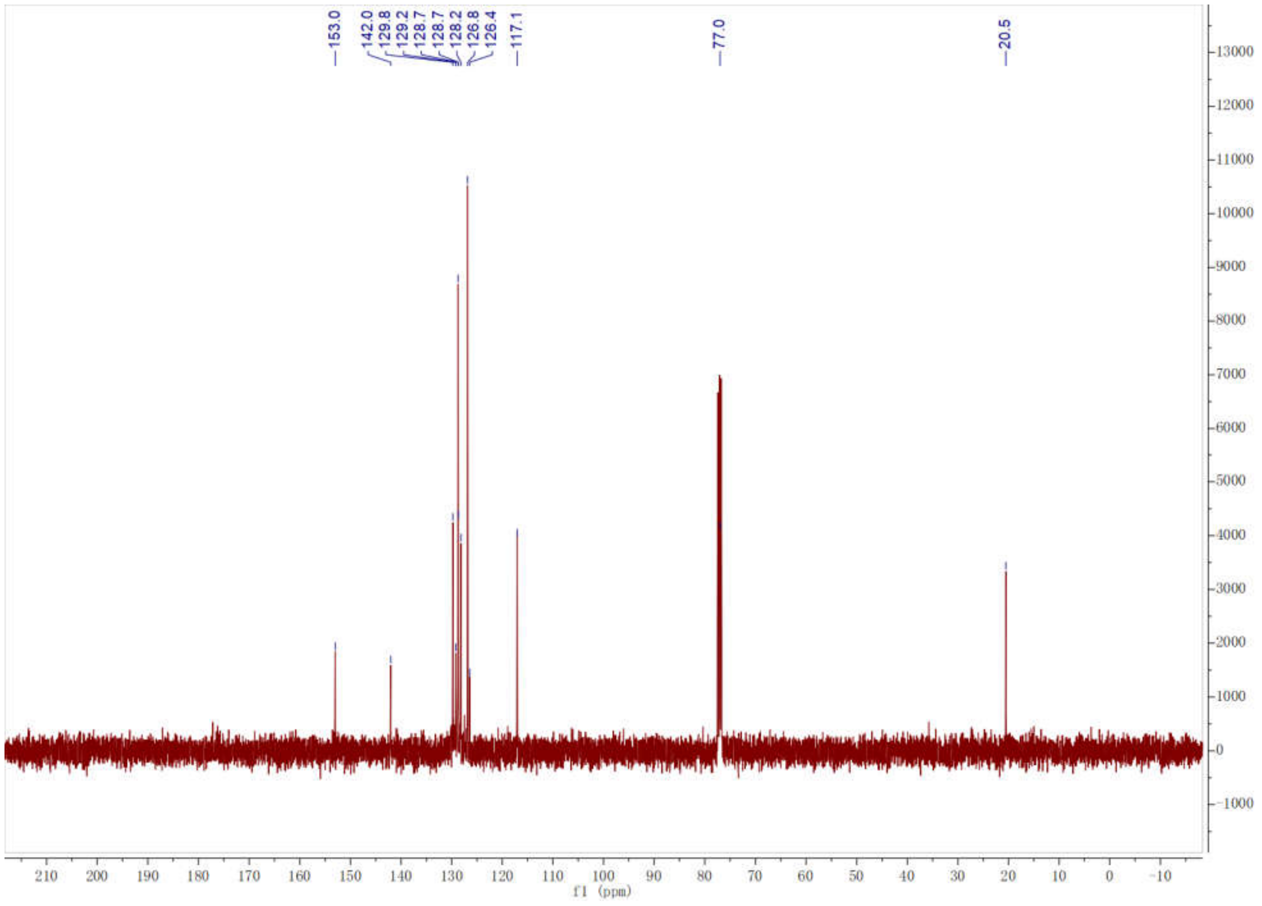
153.0
142.0 129.8 129.2 128.7 128.7 128.2 126.8 126.4
117.1
77.0
20.5
f1 (ppm)

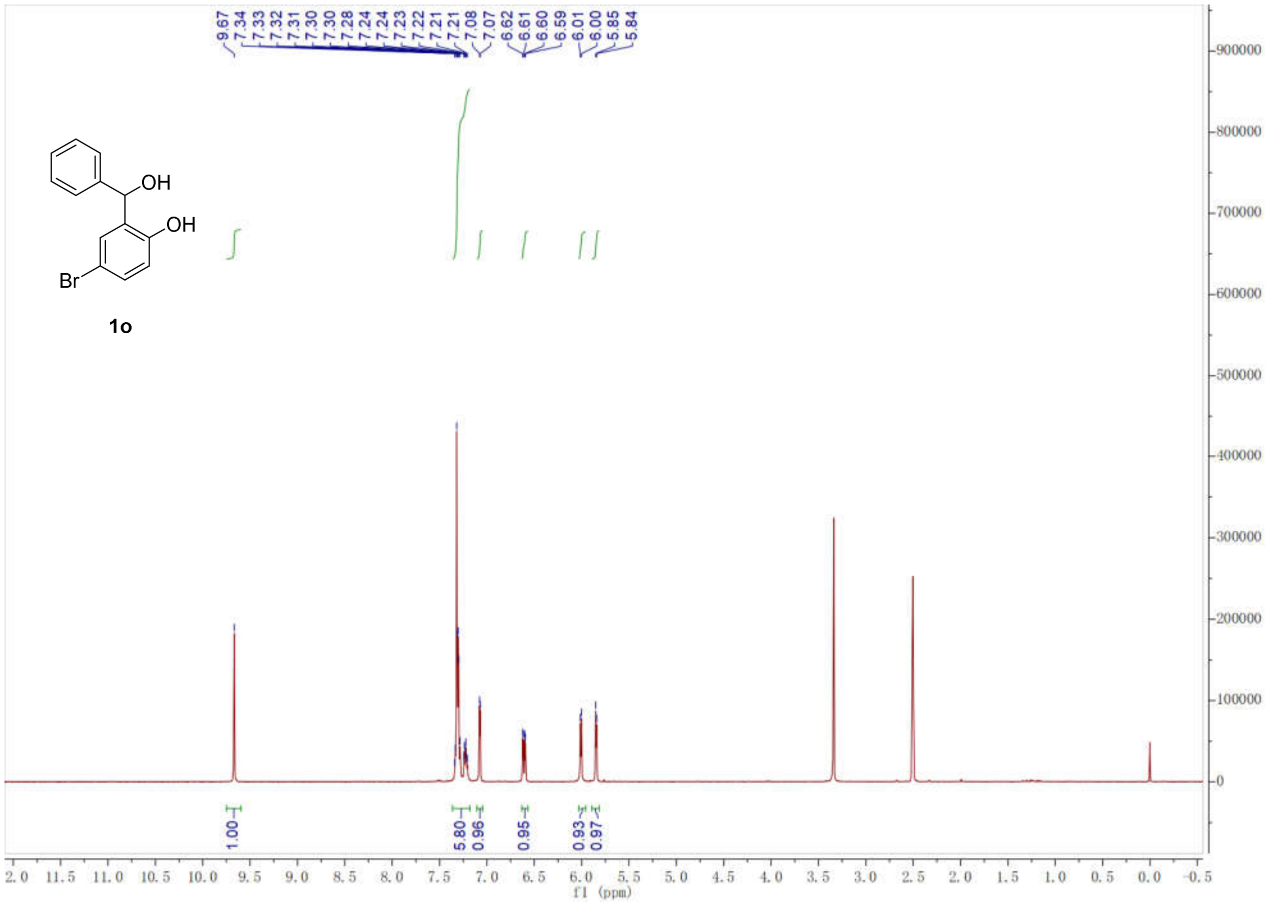
OH
OH
Br
1o

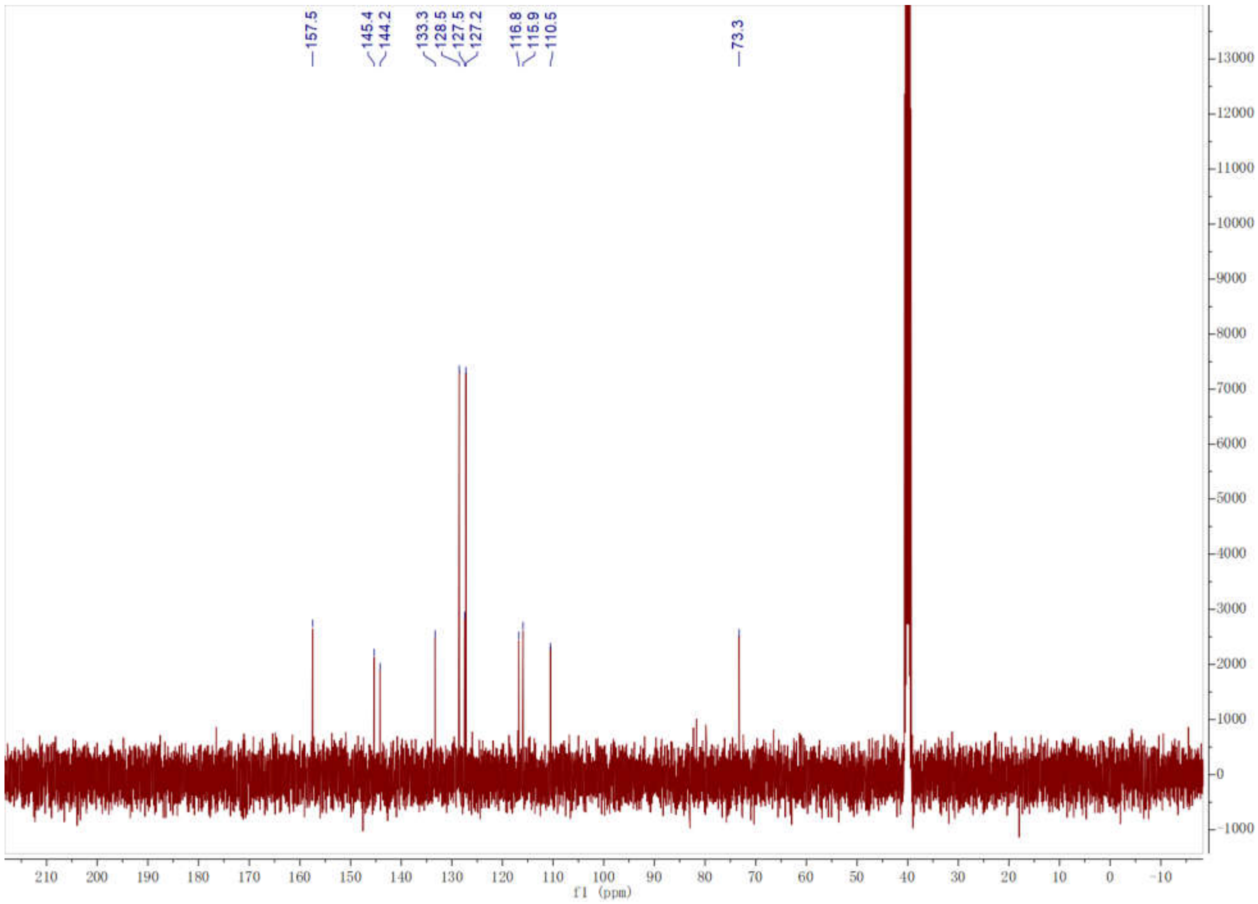

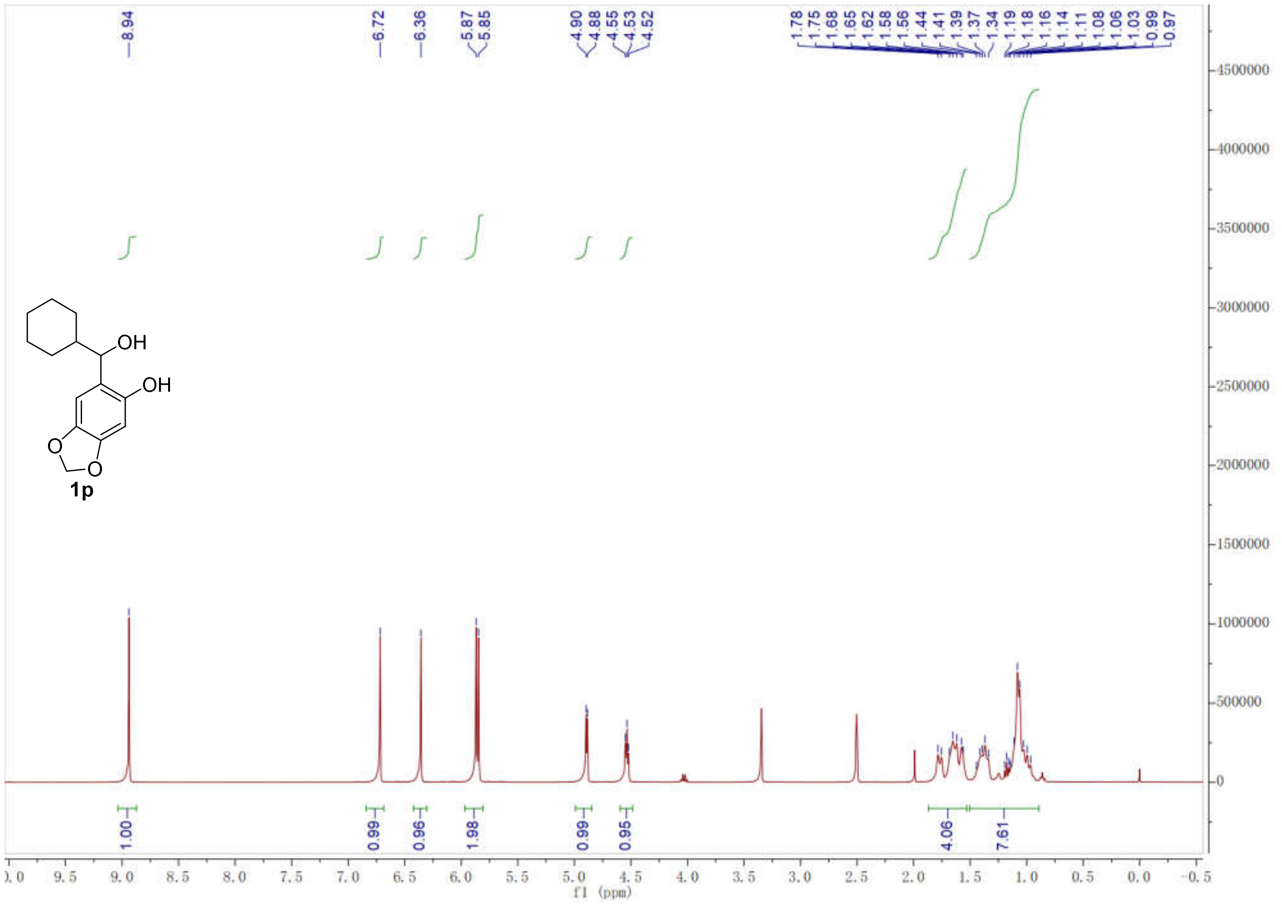
8.94
6.72
6.36
5.87
5.85
4.90
4.88
4.55
4.53
4.52
1.78 1.75 1.68 1.65 1.62 1.58 1.56 1.44 1.41 1.39 1.37 1.34 1.19 1.18 1.16 1.14 1.11 1.08 1.06 1.03 0.99 0.97
OH
OH
O
O
1p
1.00
0.99
0.96
1.98
0.99
0.95
4.06
7.61
f1 (ppm)

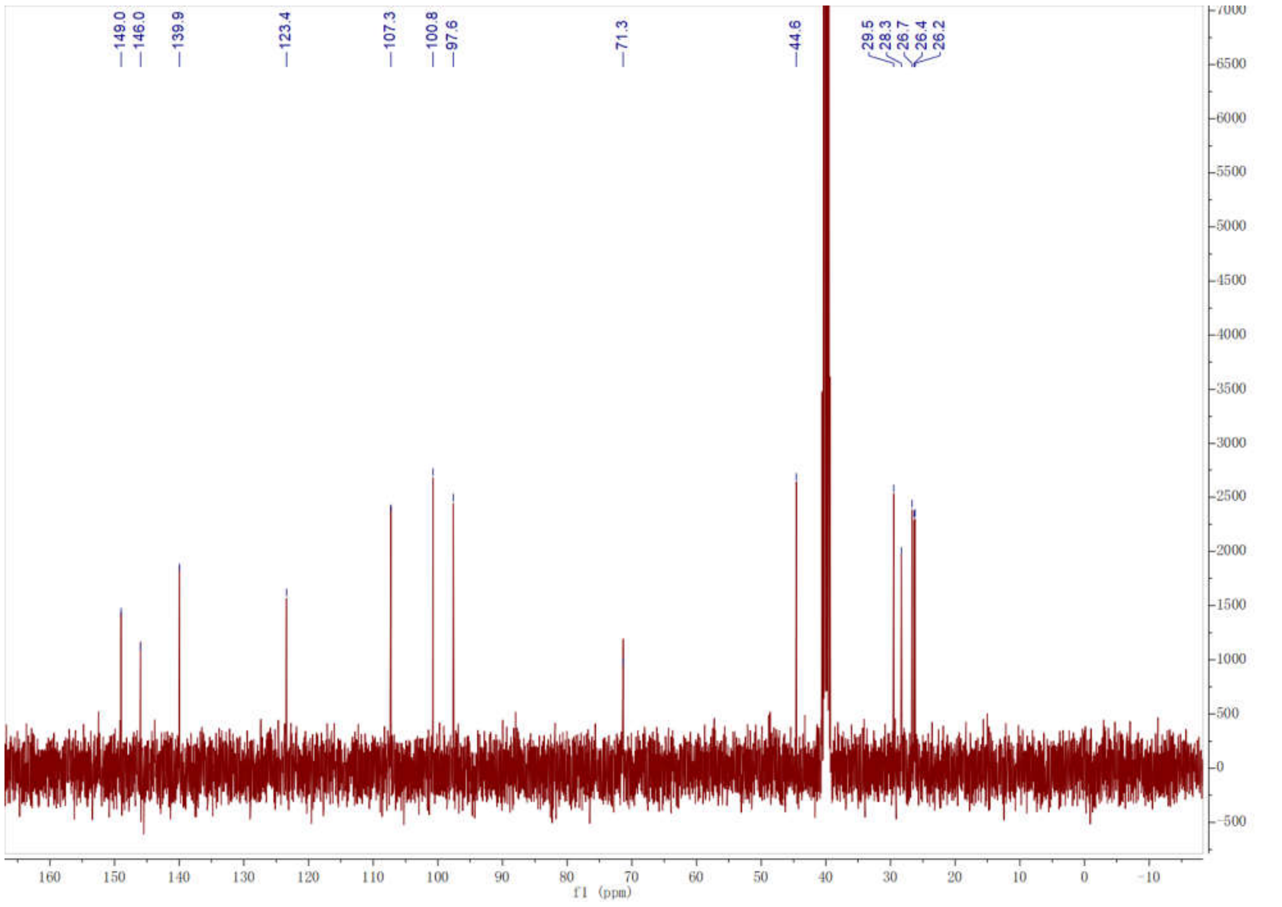
149.0
146.0
139.9
123.4
107.3
100.8
97.6
71.3
44.6
29.5
28.3
26.7
26.4
26.2
f1 (ppm)

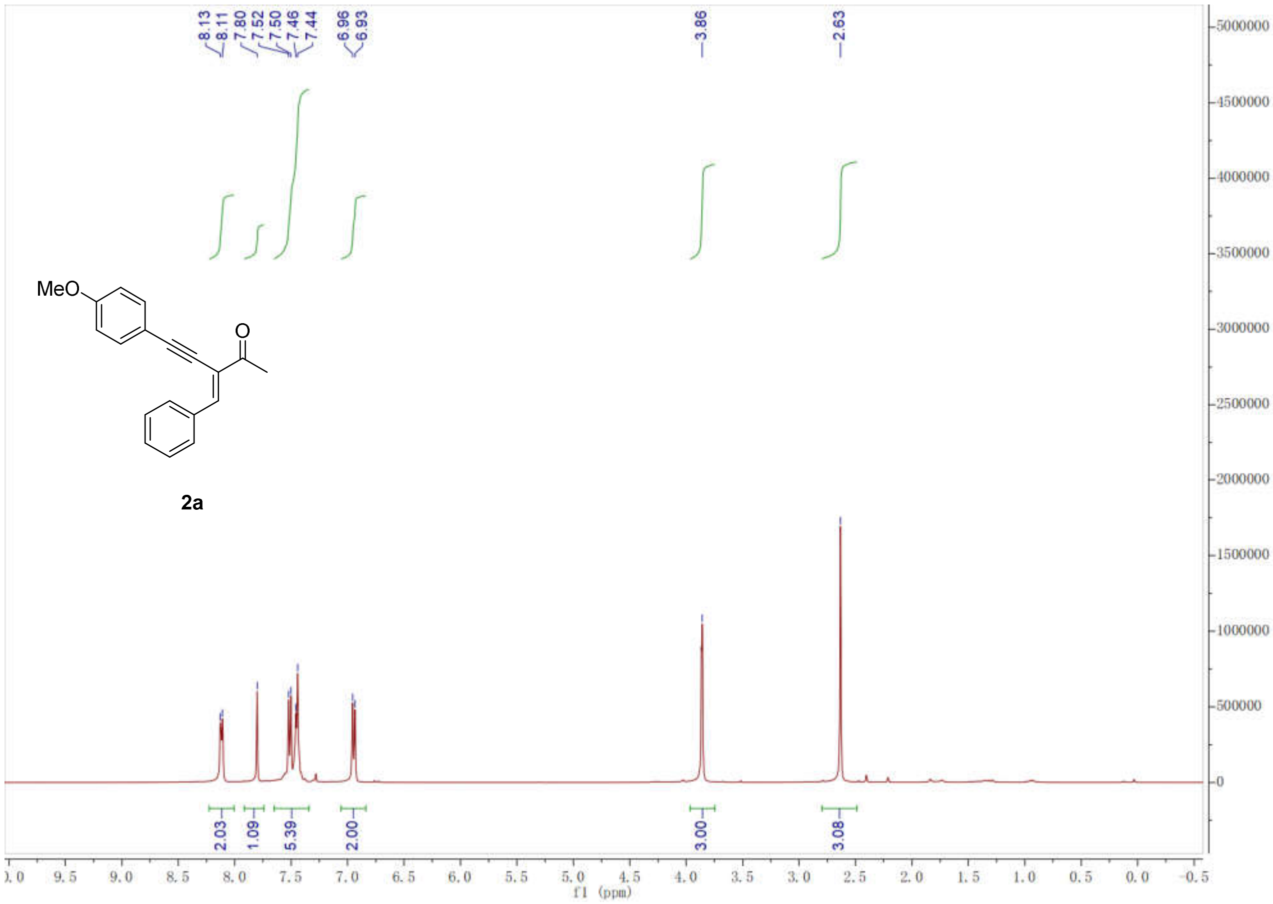
8.13
8.11
7.80
7.52
7.50
7.46
7.44
6.96
6.93
3.86
2.63
MeO
O
2a
2.03
1.09
5.39
2.00
3.00
3.08
f1 (ppm)

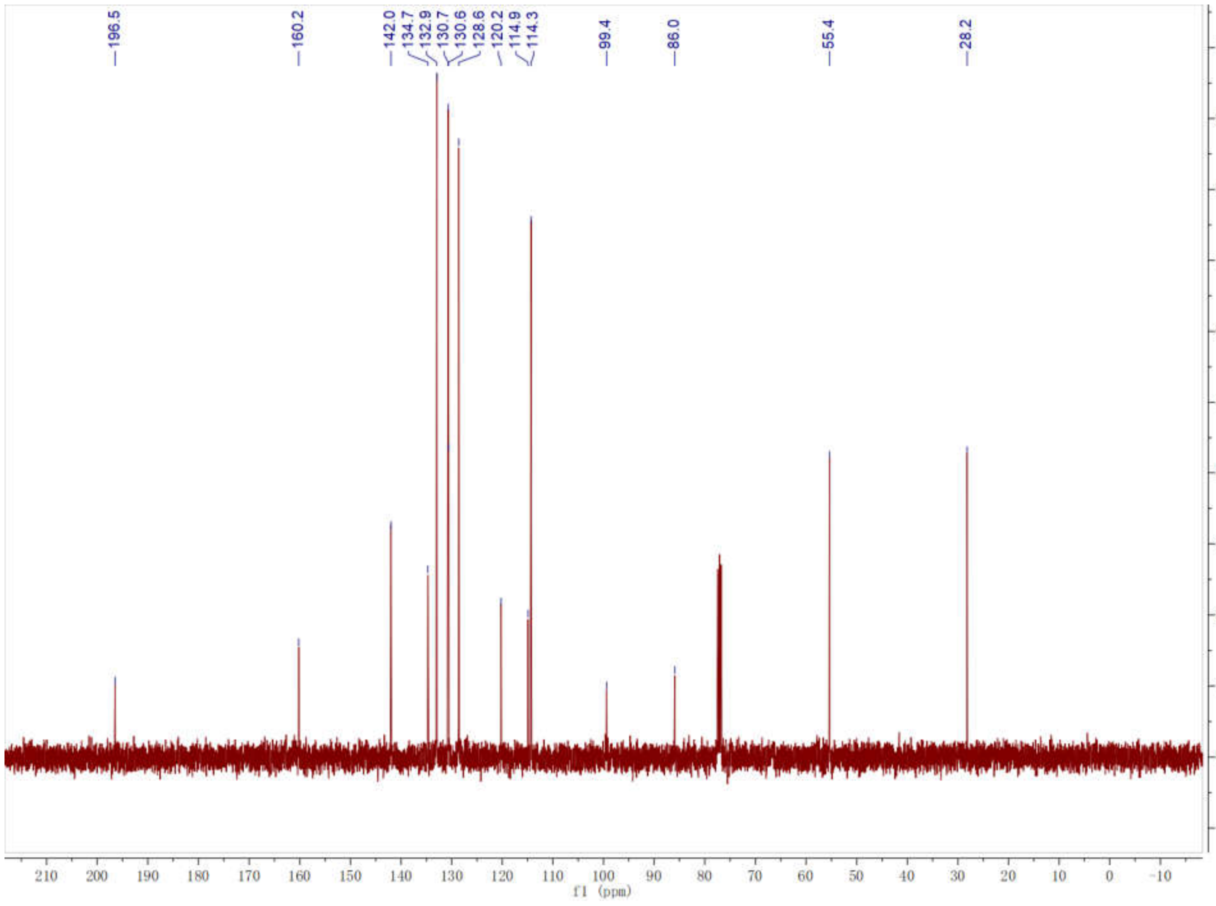
196.5
160.2
142.0
134.7
132.9
130.7
130.6
128.6
120.2
114.9
114.3
99.4
86.0
55.4
28.2
f1 (ppm)

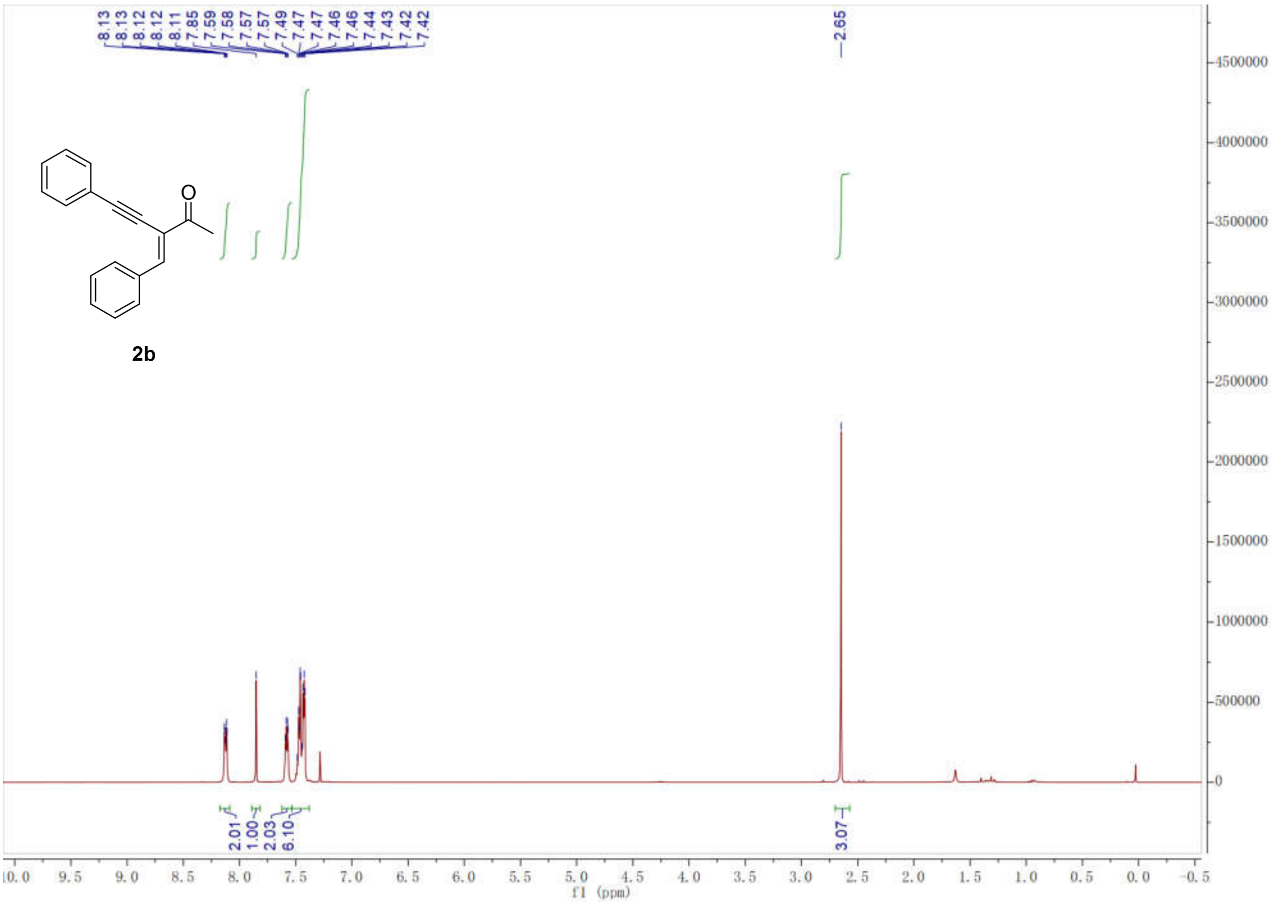
8.13
8.13
8.12
8.12
8.11
7.85
7.59
7.58
7.57
7.57
7.49
7.47
7.47
7.46
7.46
7.44
7.43
7.42
7.42
2.65
2b
2.01
1.00
2.03
6.10
3.07
f1 (ppm)

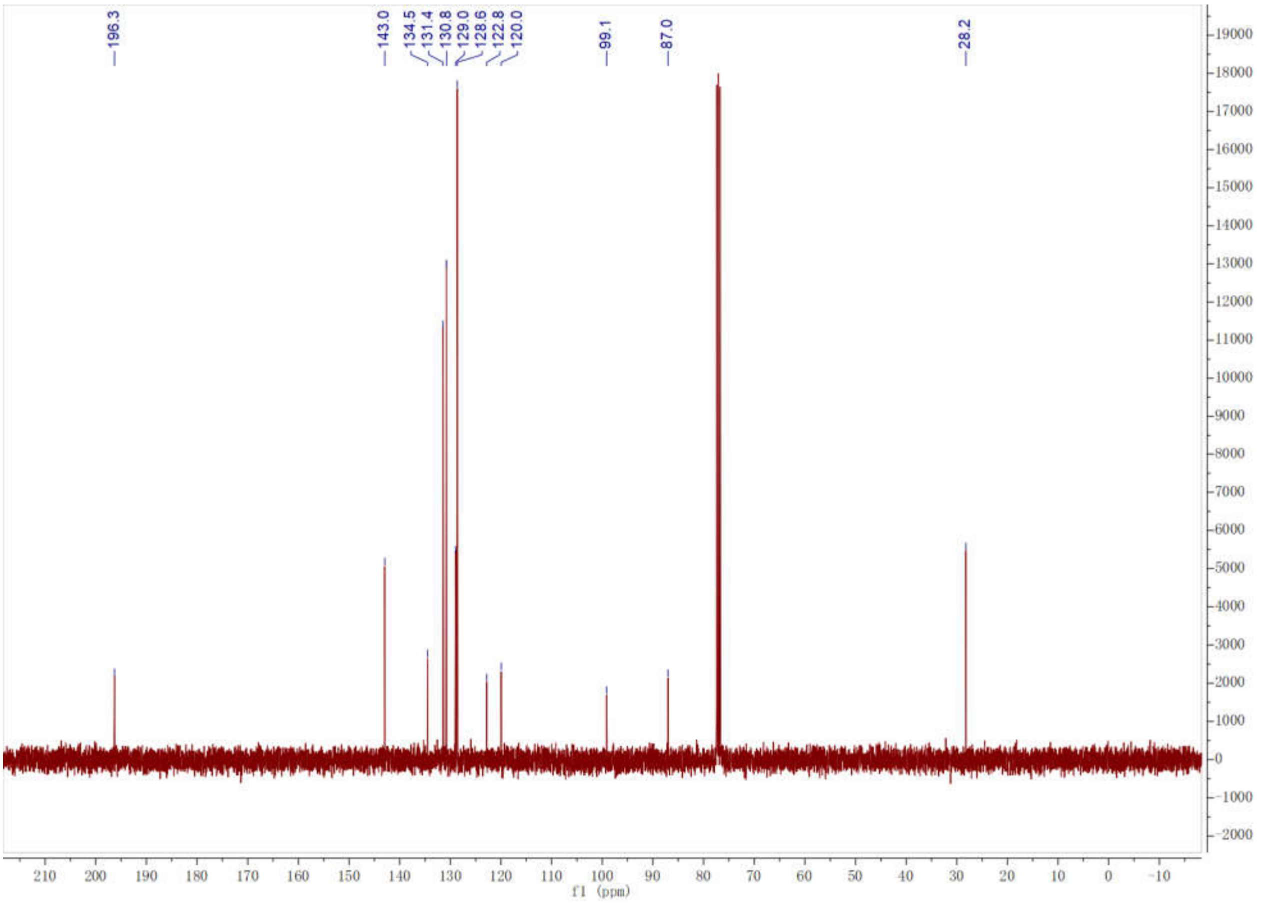
196.3
143.0
134.5
131.4
130.8
129.0
128.6
122.8
120.0
99.1
87.0
28.2
f1 (ppm)

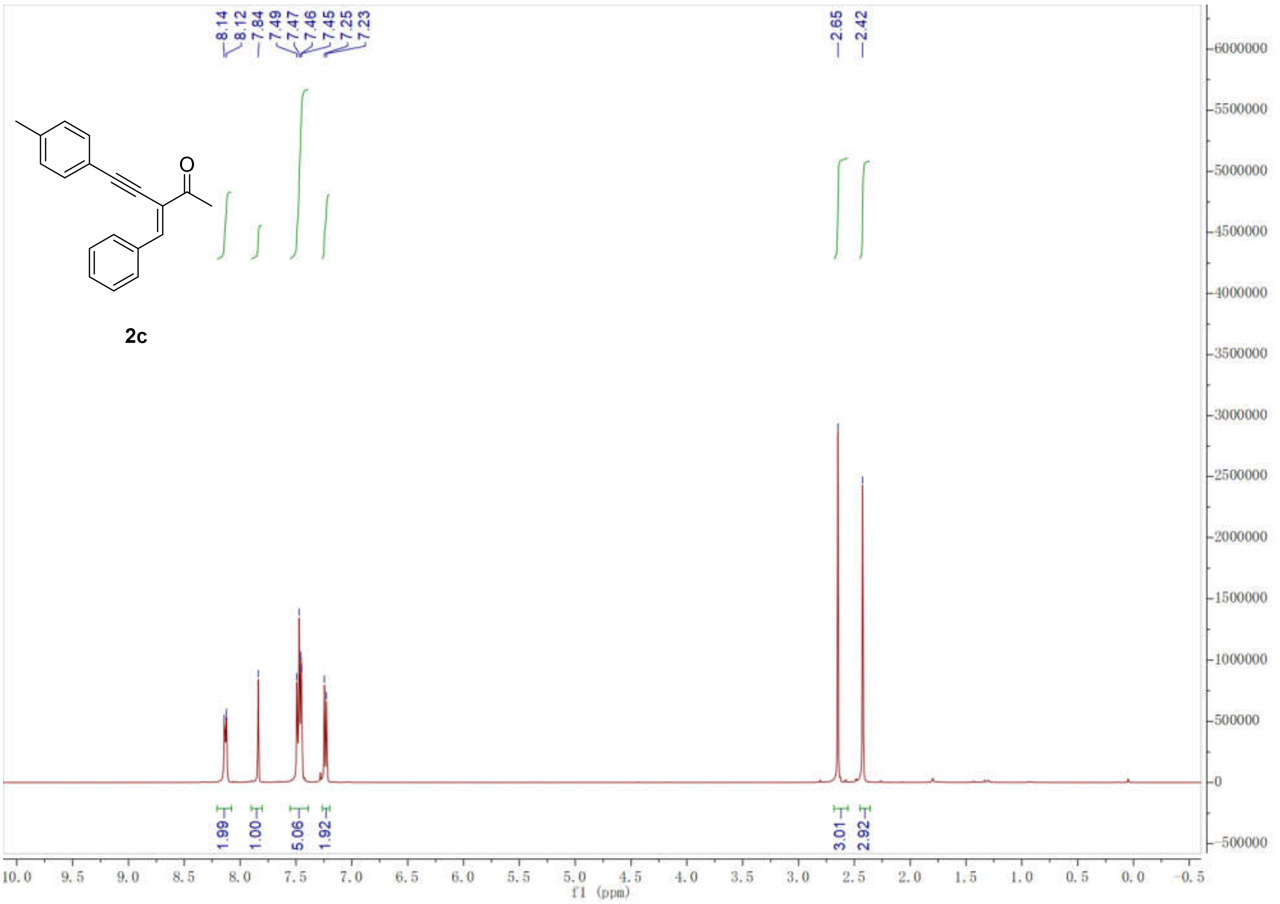
8.14
8.12
7.84
7.49
7.47
7.46
7.45
7.25
7.23
2.65
2.42
2c
1.99
1.00
5.06
1.92
3.01
2.92
f1 (ppm)

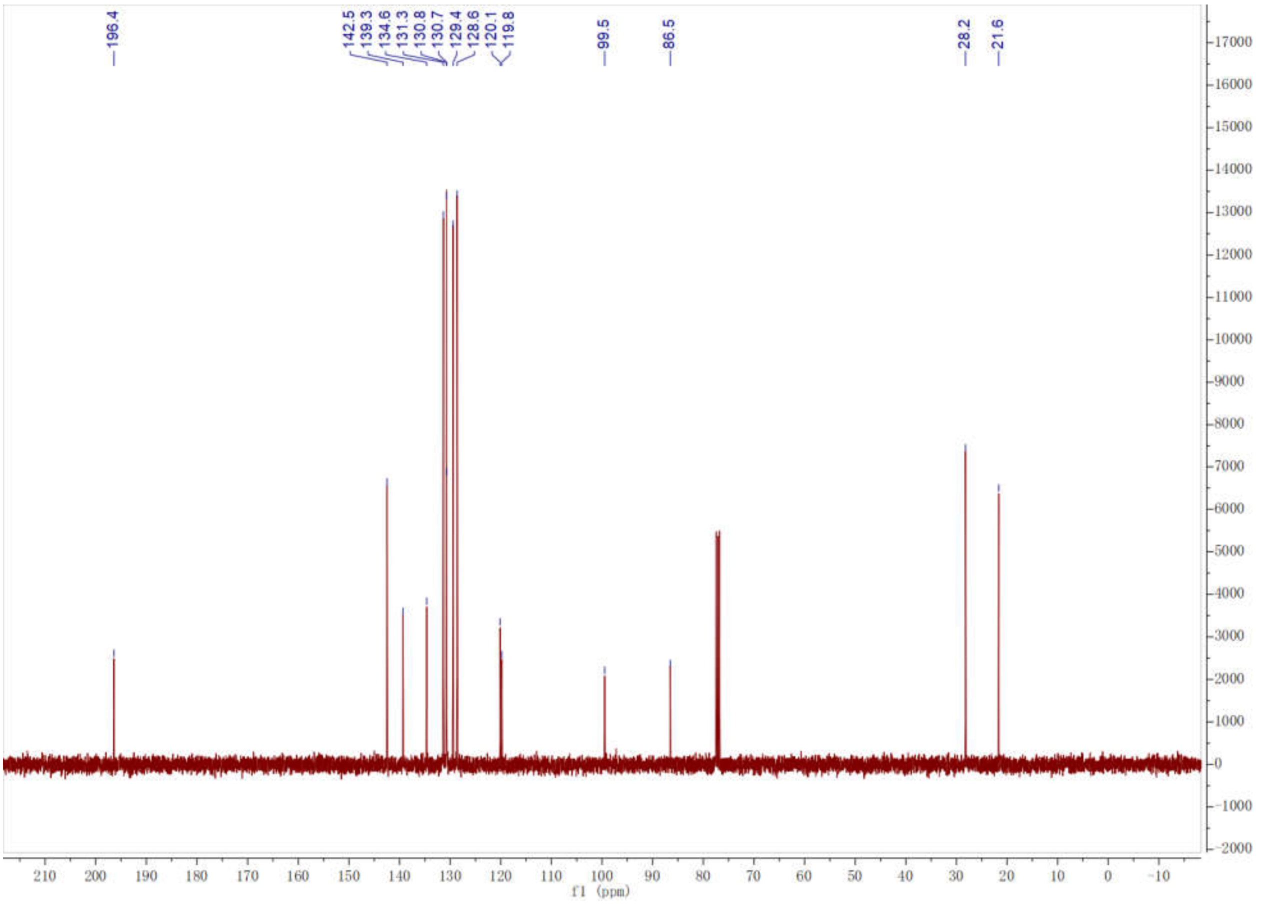
196.4
142.5
139.3
134.6
131.3
130.8
130.7
129.4
128.6
120.1
119.8
99.5
86.5
28.2
21.6
f1 (ppm)

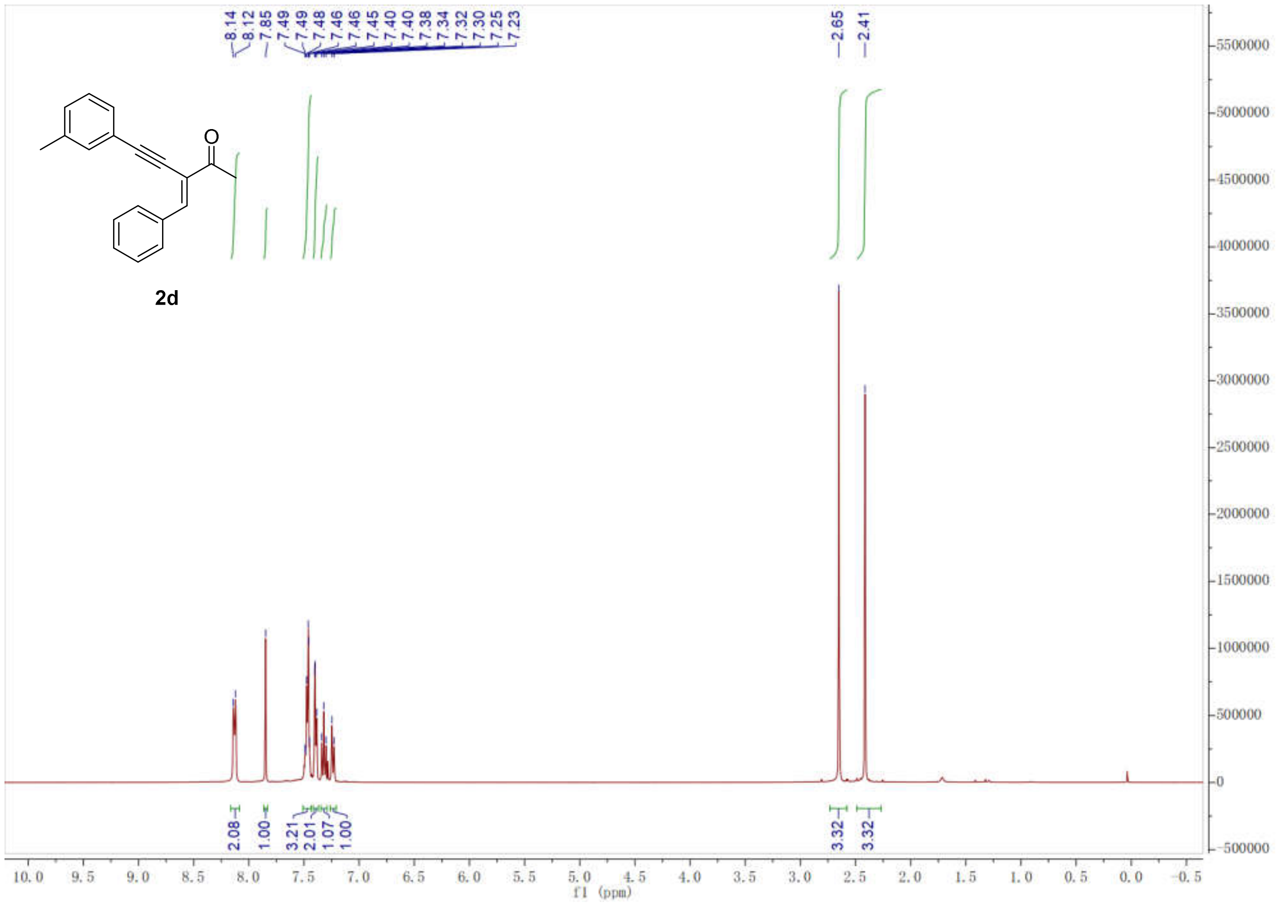
2d
8.14
8.12
7.85
7.49
7.48
7.46
7.45
7.40
7.40
7.38
7.34
7.32
7.30
7.25
7.23
2.65
2.41
2.08
1.00
3.21
2.01
1.07
1.00
3.32
3.32
f1 (ppm)

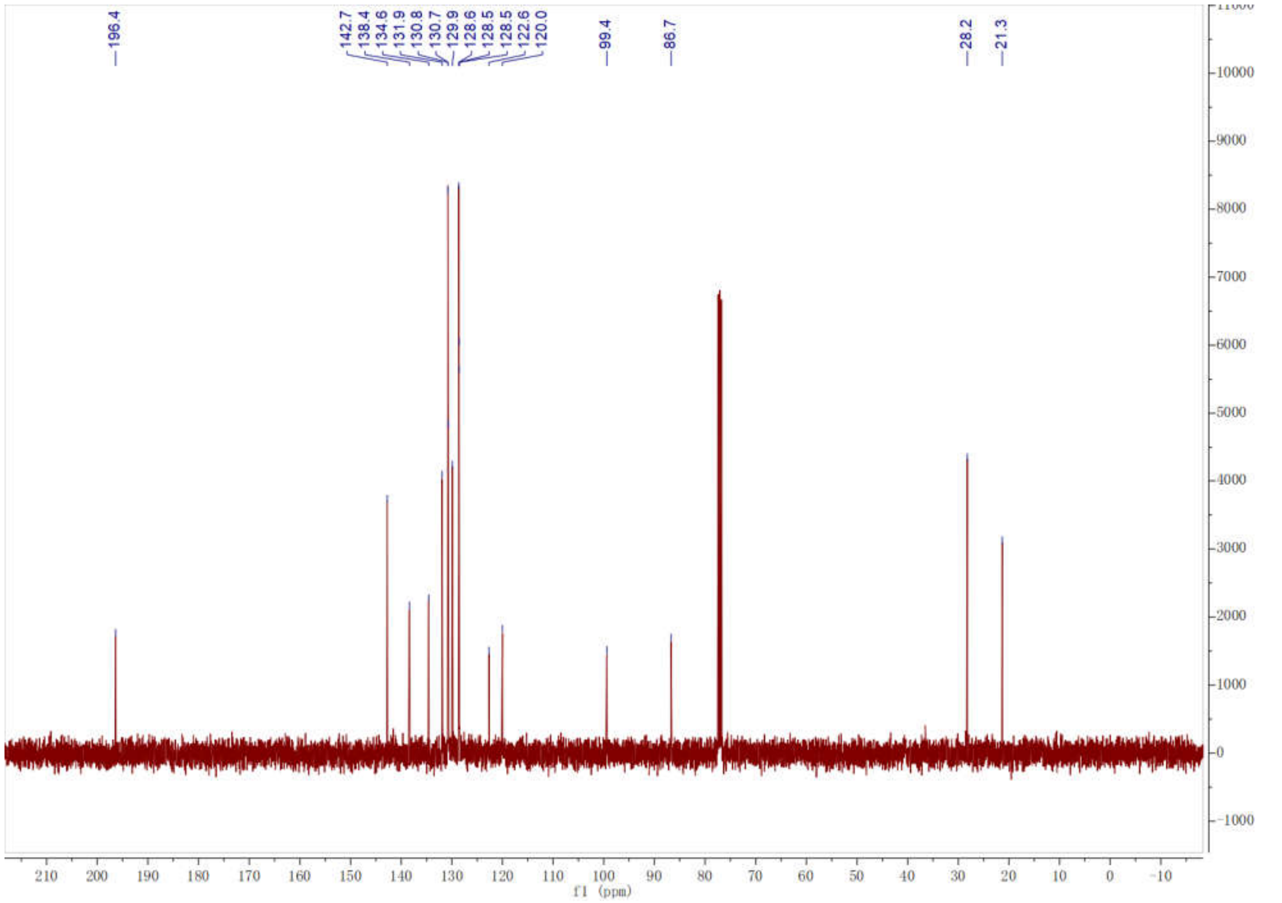
196.4
142.7
138.4
134.6
131.9
130.8
130.7
129.9
128.6
128.5
122.6
120.0
99.4
86.7
28.2
21.3
f1 (ppm)

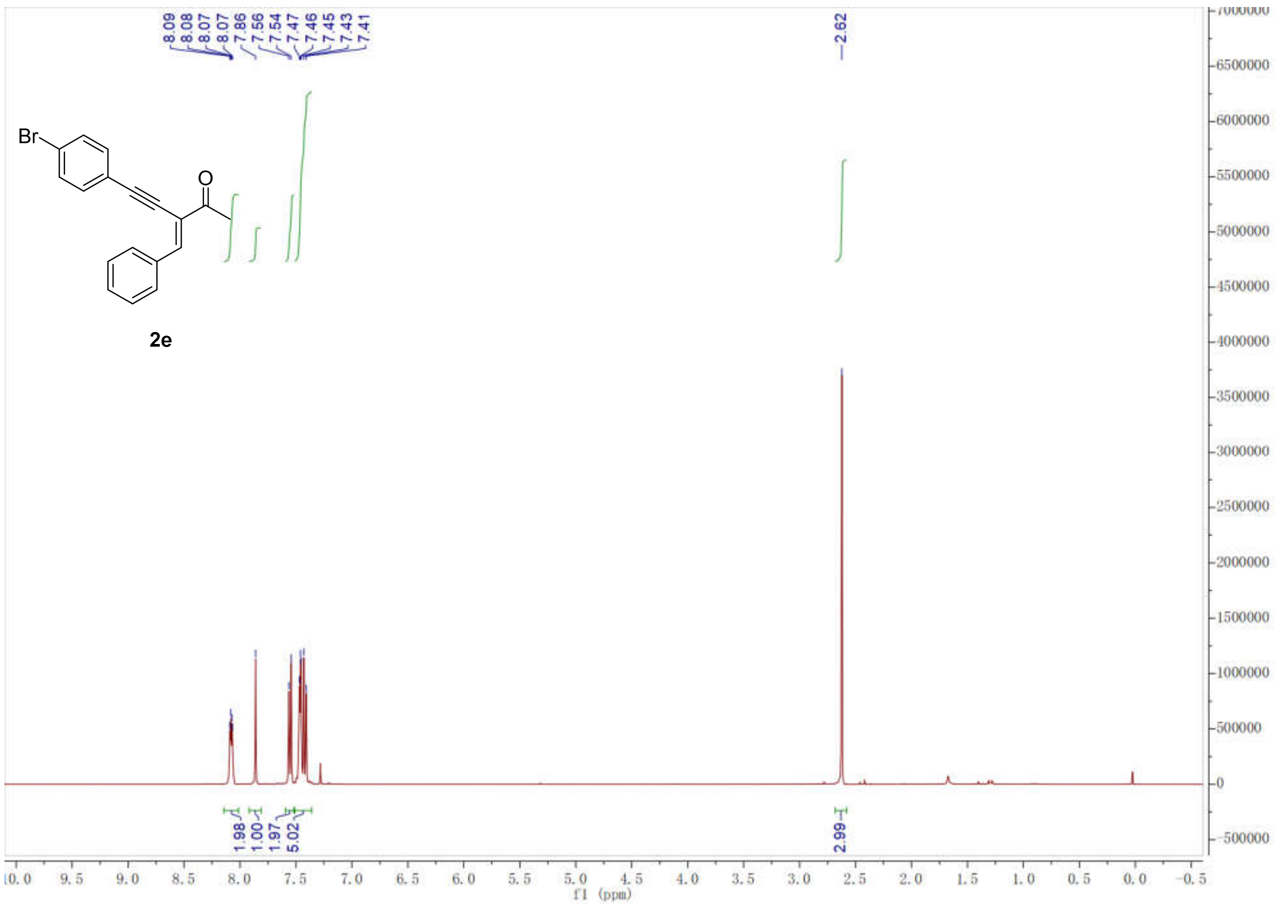
8.09
8.08
8.07
8.07
7.86
7.56
7.54
7.47
7.46
7.45
7.43
7.41
2.62
Br
O
2e
1.98
1.00
1.97
5.02
2.99
f1 (ppm)

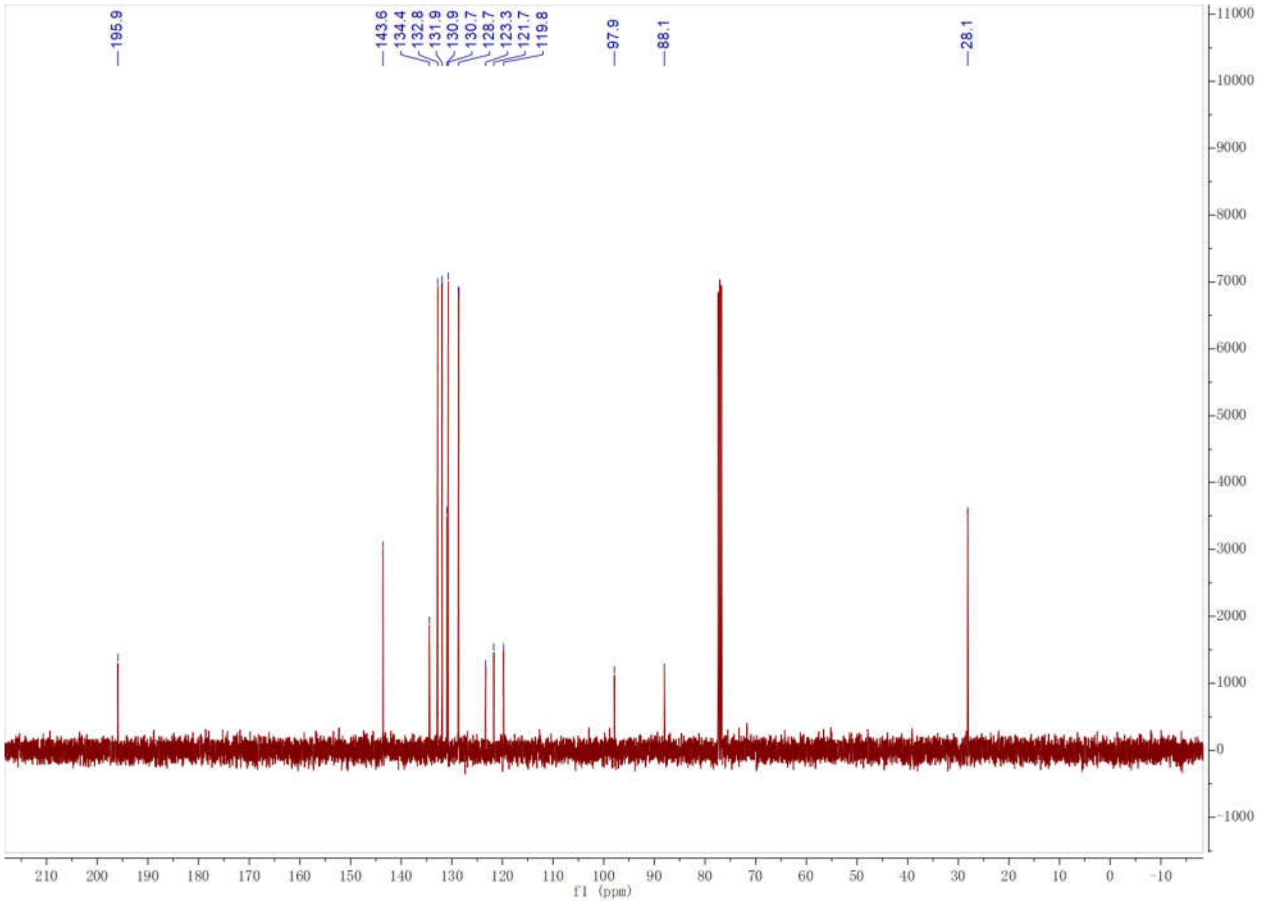
195.9
143.6
134.4
132.8
131.9
130.9
130.7
128.7
123.3
121.7
119.8
97.9
88.1
28.1
f1 (ppm)

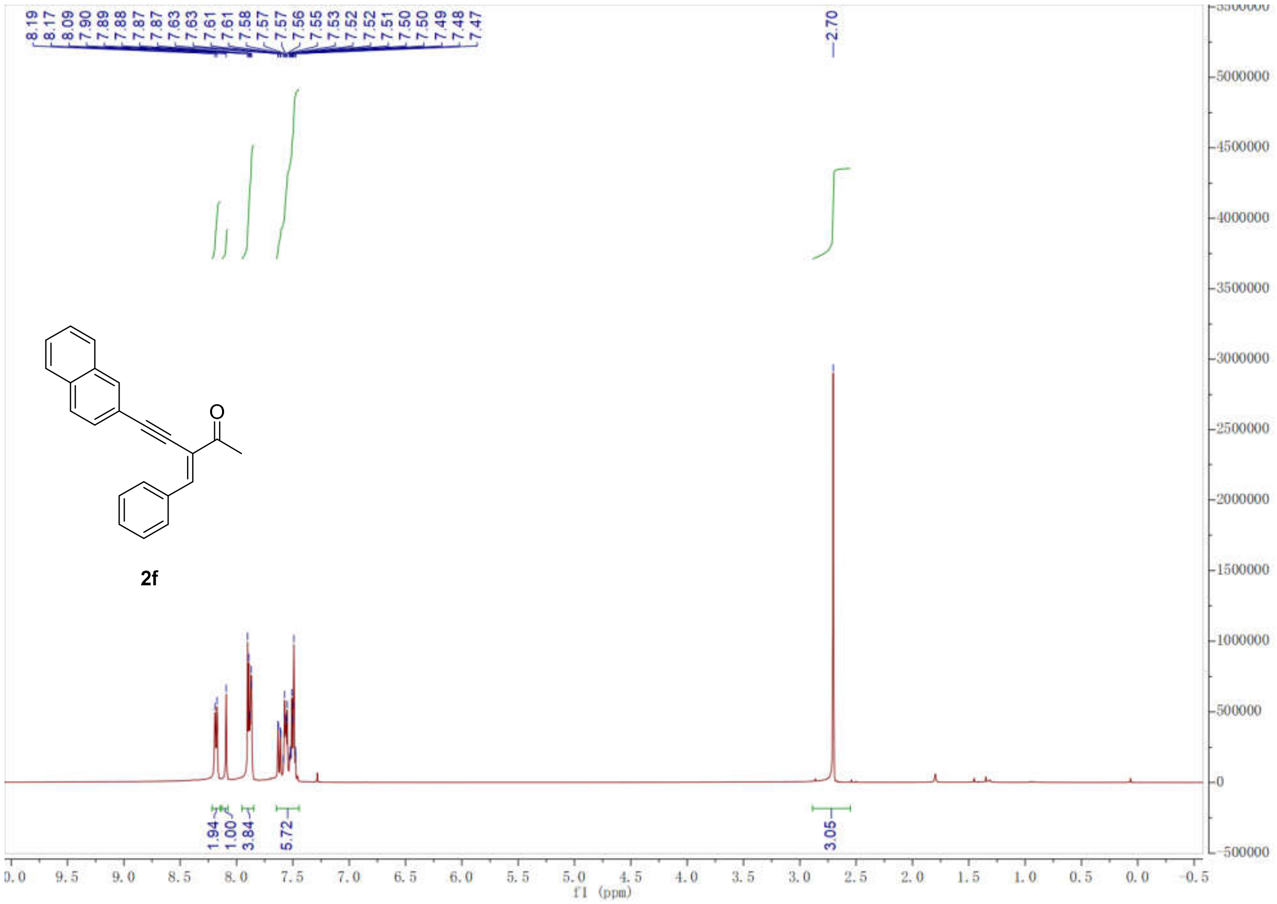
8.19
8.17
8.09
7.90
7.89
7.88
7.87
7.87
7.63
7.63
7.61
7.61
7.58
7.57
7.57
7.56
7.55
7.53
7.52
7.52
7.51
7.50
7.50
7.49
7.48
7.47
2.70
O
2f
1.94
1.00
3.84
5.72
3.05
f1 (ppm)

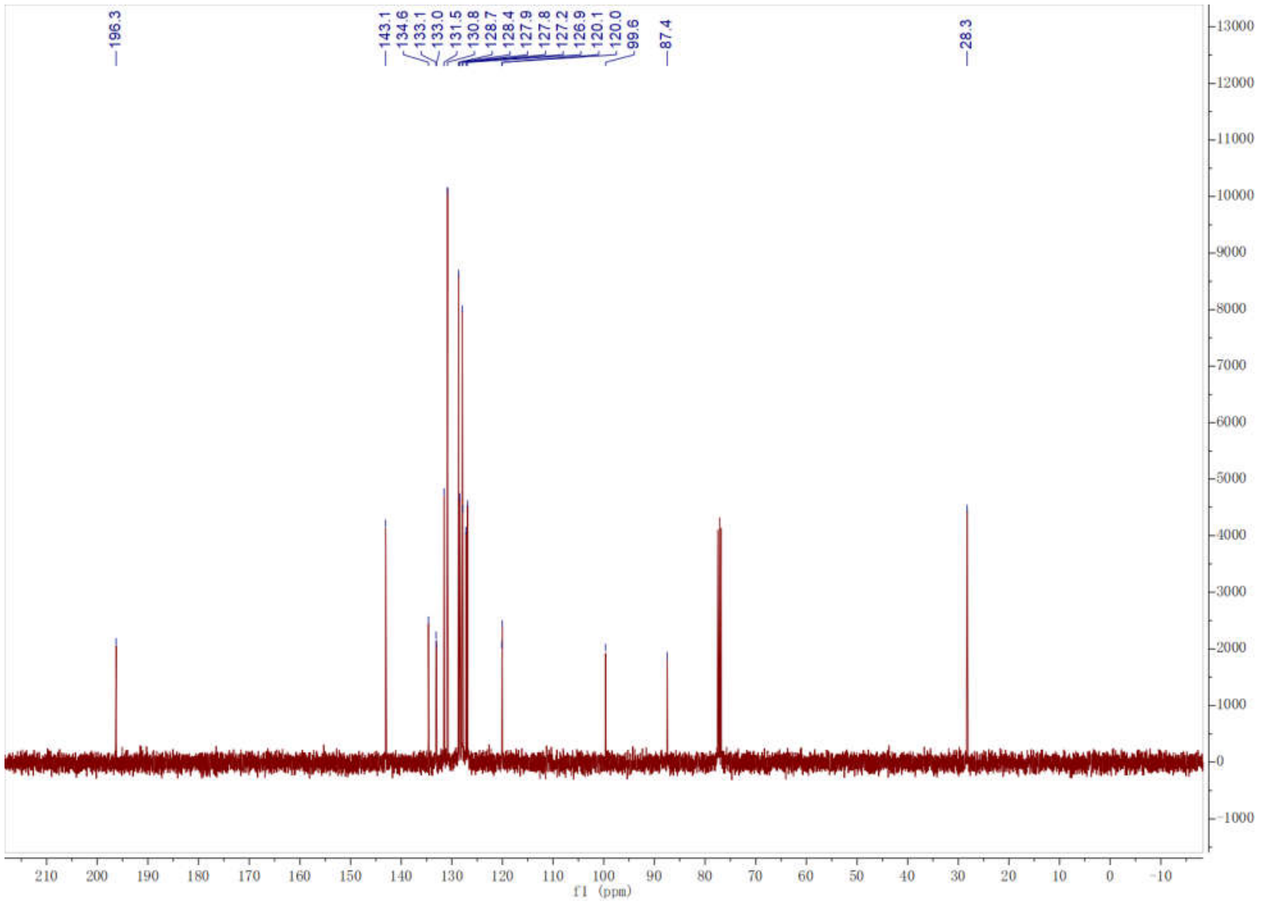
196.3
143.1
134.6
133.1
133.0
131.5
130.8
128.7
128.4
127.9
127.8
127.2
126.9
120.1
120.0
99.6
87.4
28.3
f1 (ppm)

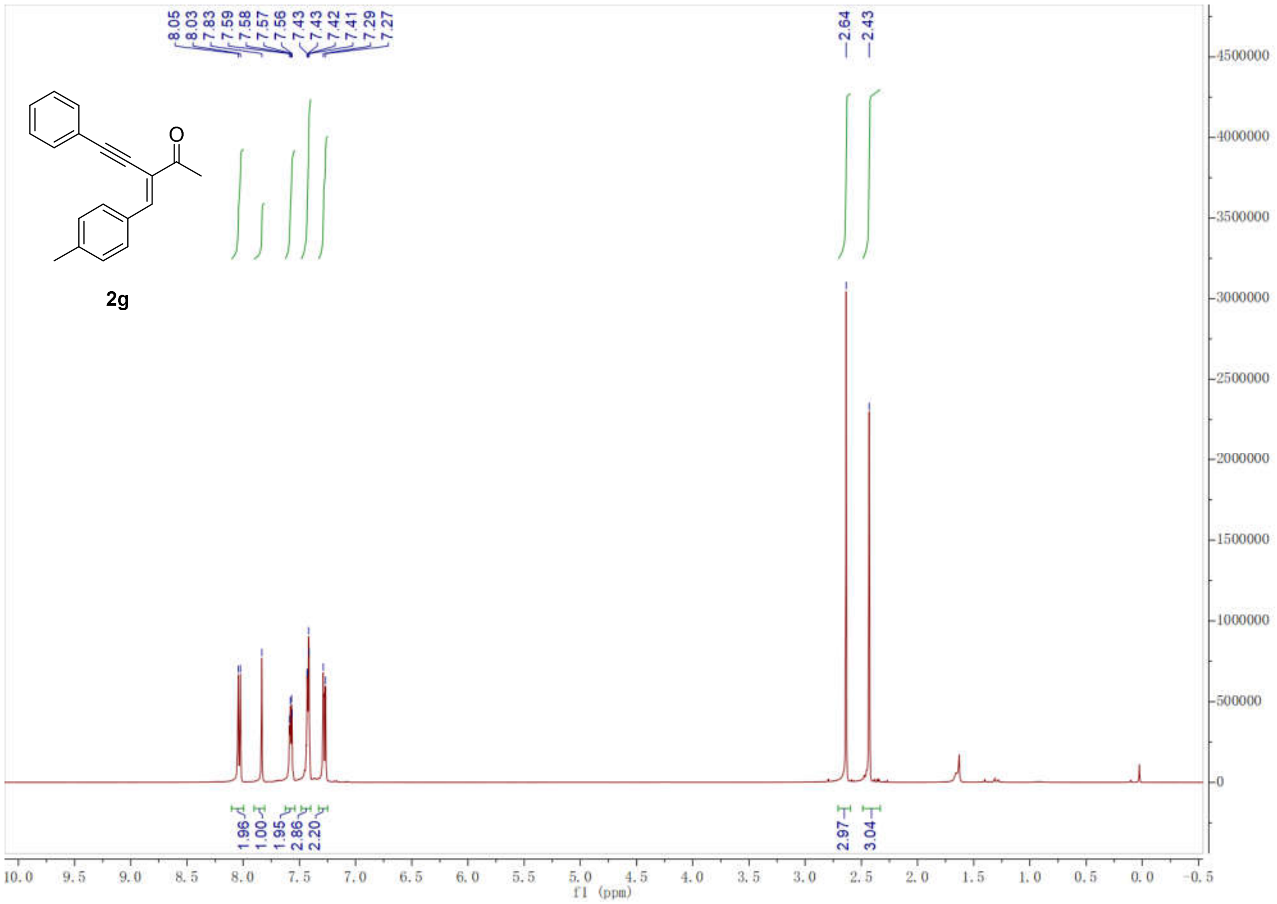

8.05
8.03
7.83
7.59
7.58
7.57
7.56
7.43
7.43
7.42
7.41
7.29
7.27
2.64
2.43
O
2g
1.96
1.00
1.95
2.86
2.20
2.97
3.04
f1 (ppm)

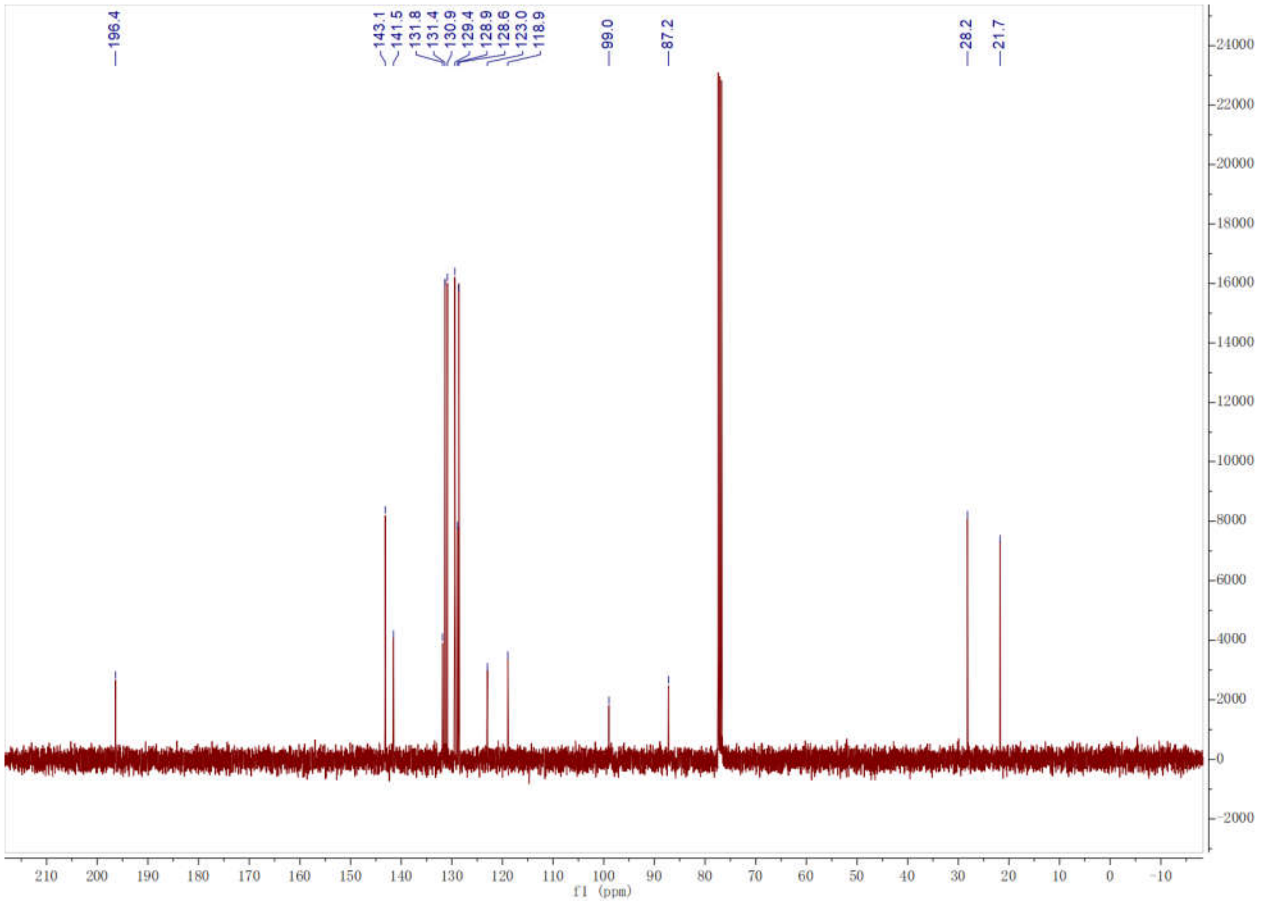

196.4
143.1
141.5
131.8
131.4
130.9
129.4
128.9
128.6
123.0
118.9
99.0
87.2
28.2
21.7
f1 (ppm)

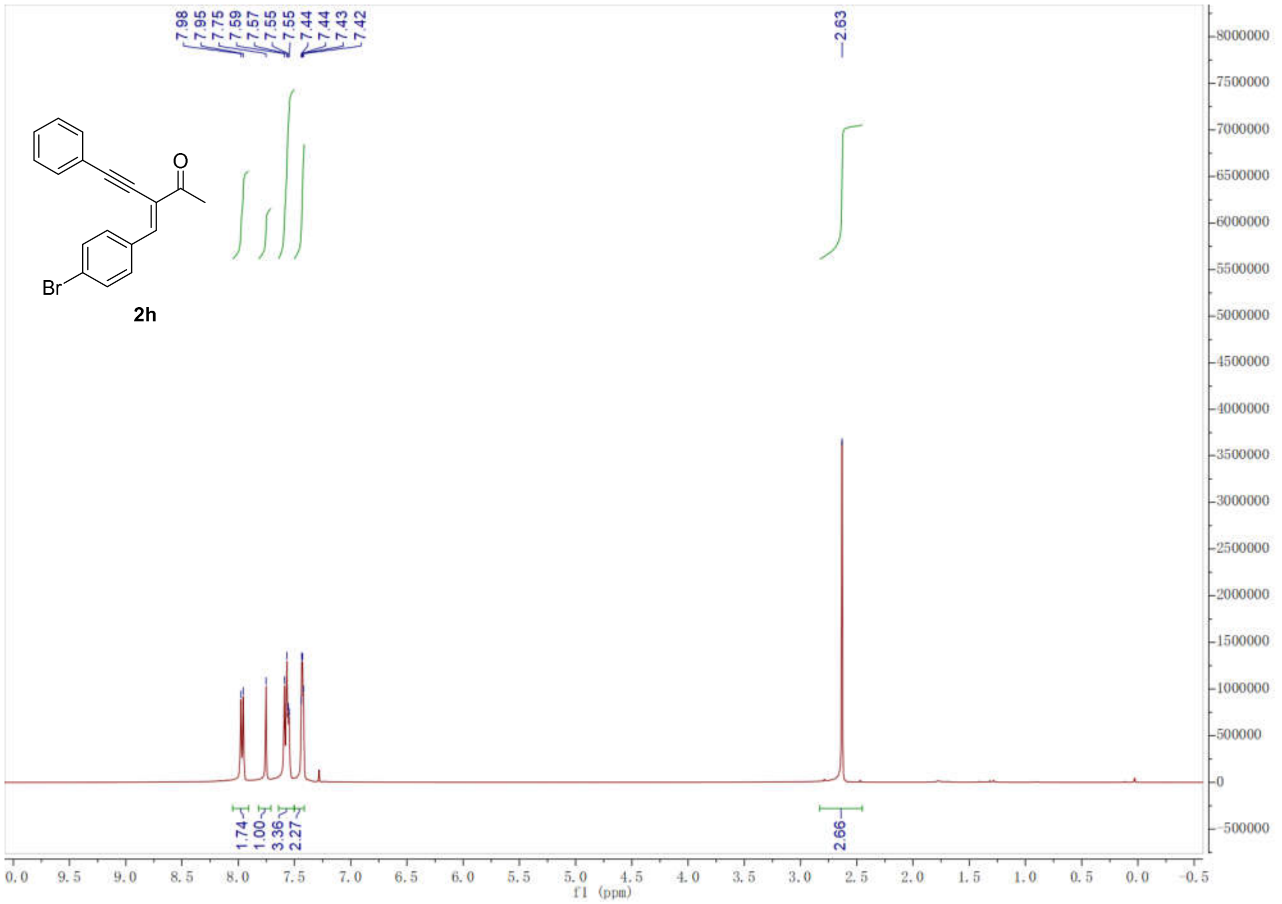
7.98
7.95
7.75
7.59
7.57
7.55
7.55
7.44
7.44
7.43
7.42
2.63
O
Br
2h
1.74
1.00
3.36
2.27
2.66
f1 (ppm)

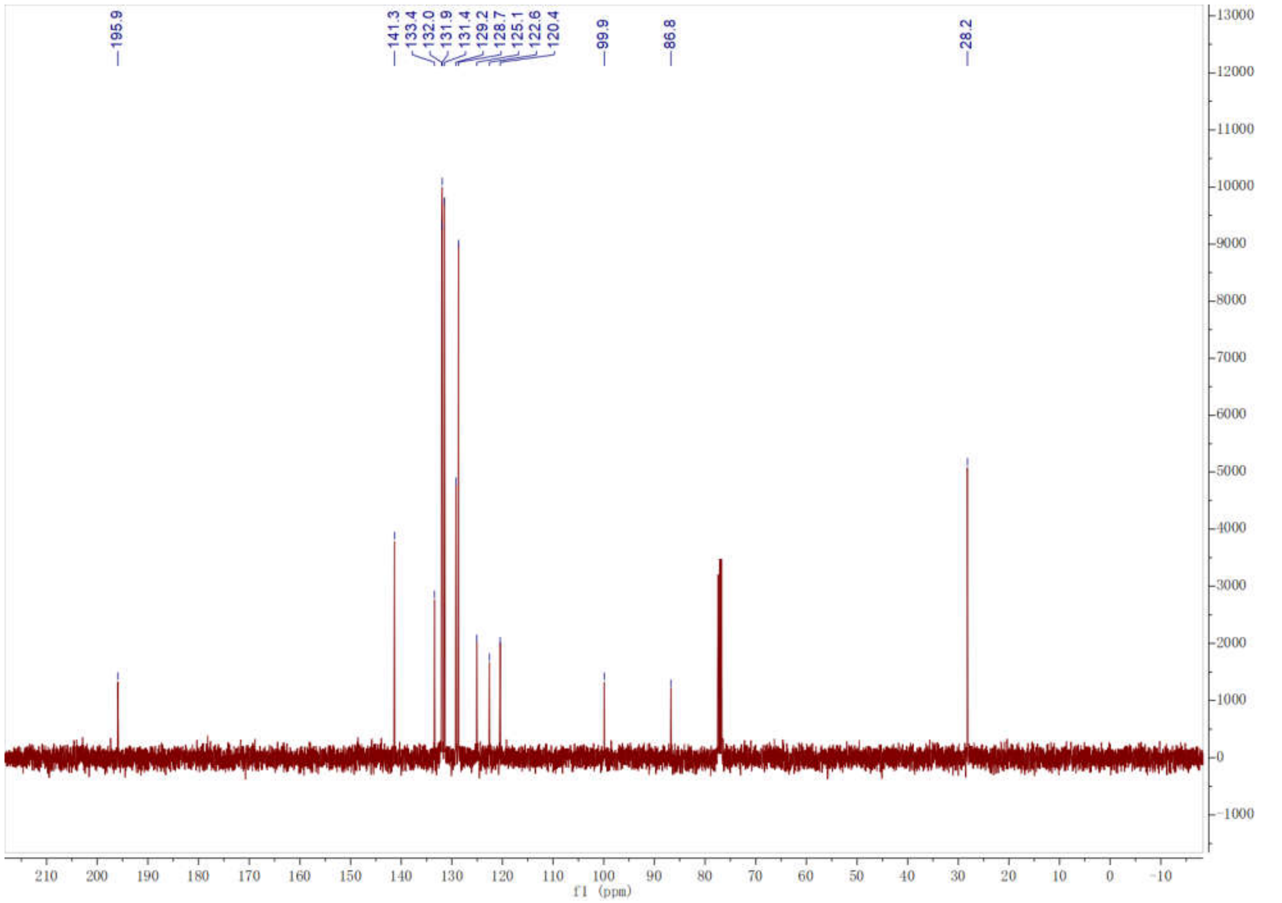
195.9
141.3
133.4
132.0
131.9
131.4
129.2
128.7
125.1
122.6
120.4
99.9
86.8
28.2
f1 (ppm)

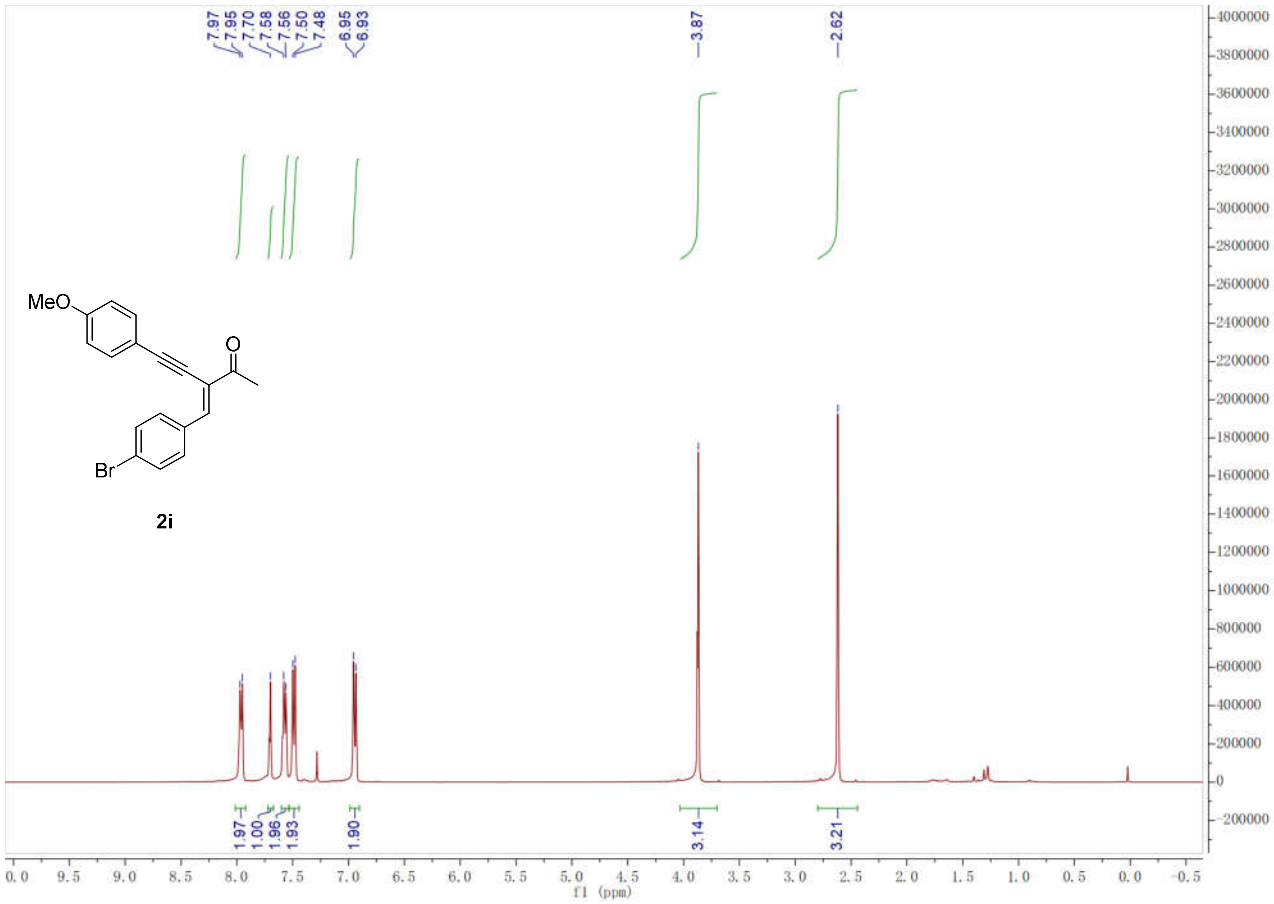
7.97
7.95
7.70
7.58
7.56
7.50
7.48
6.95
6.93
3.87
2.62
MeO
O
Br
2i
1.97
1.00
1.96
1.93
1.90
3.14
3.21
f1 (ppm)

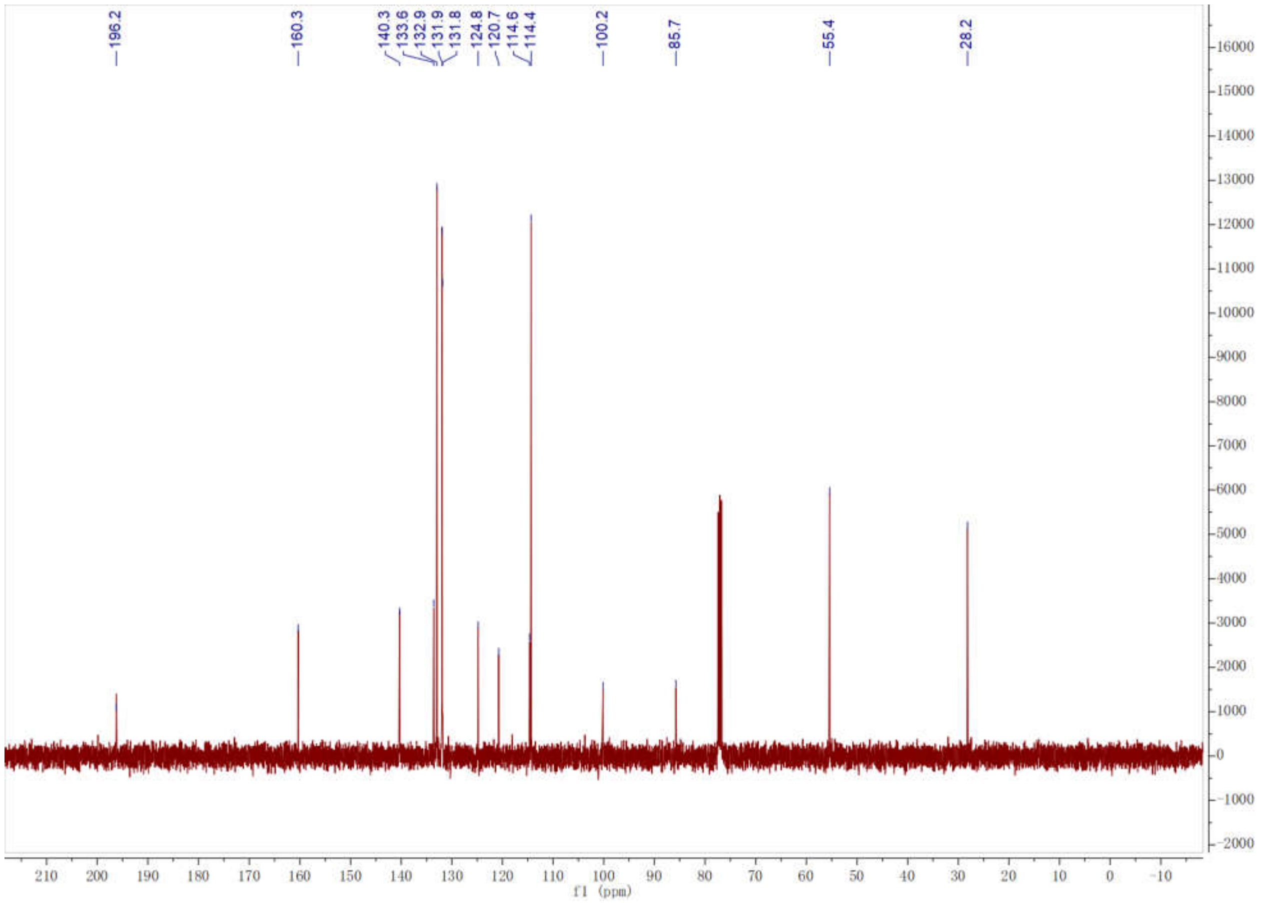
196.2
160.3
140.3
133.6
132.9
131.9
131.8
124.8
120.7
114.6
114.4
100.2
85.7
55.4
28.2
f1 (ppm)

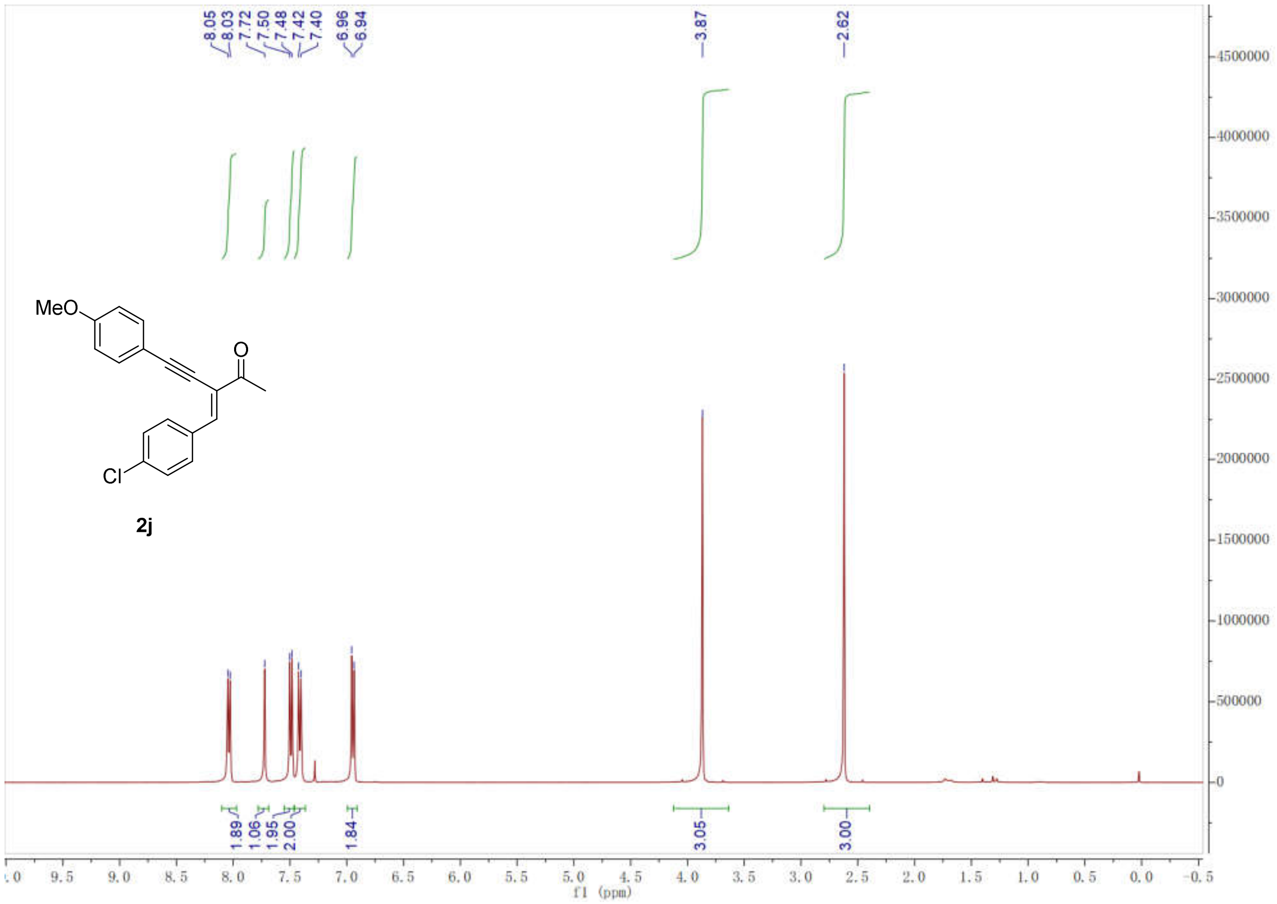
8.05
8.03
7.72
7.50
7.48
7.42
7.40
6.96
6.94
3.87
2.62
MeO
O
Cl
2j
1.89
1.06
1.95
2.00
1.84
3.05
3.00
f1 (ppm)

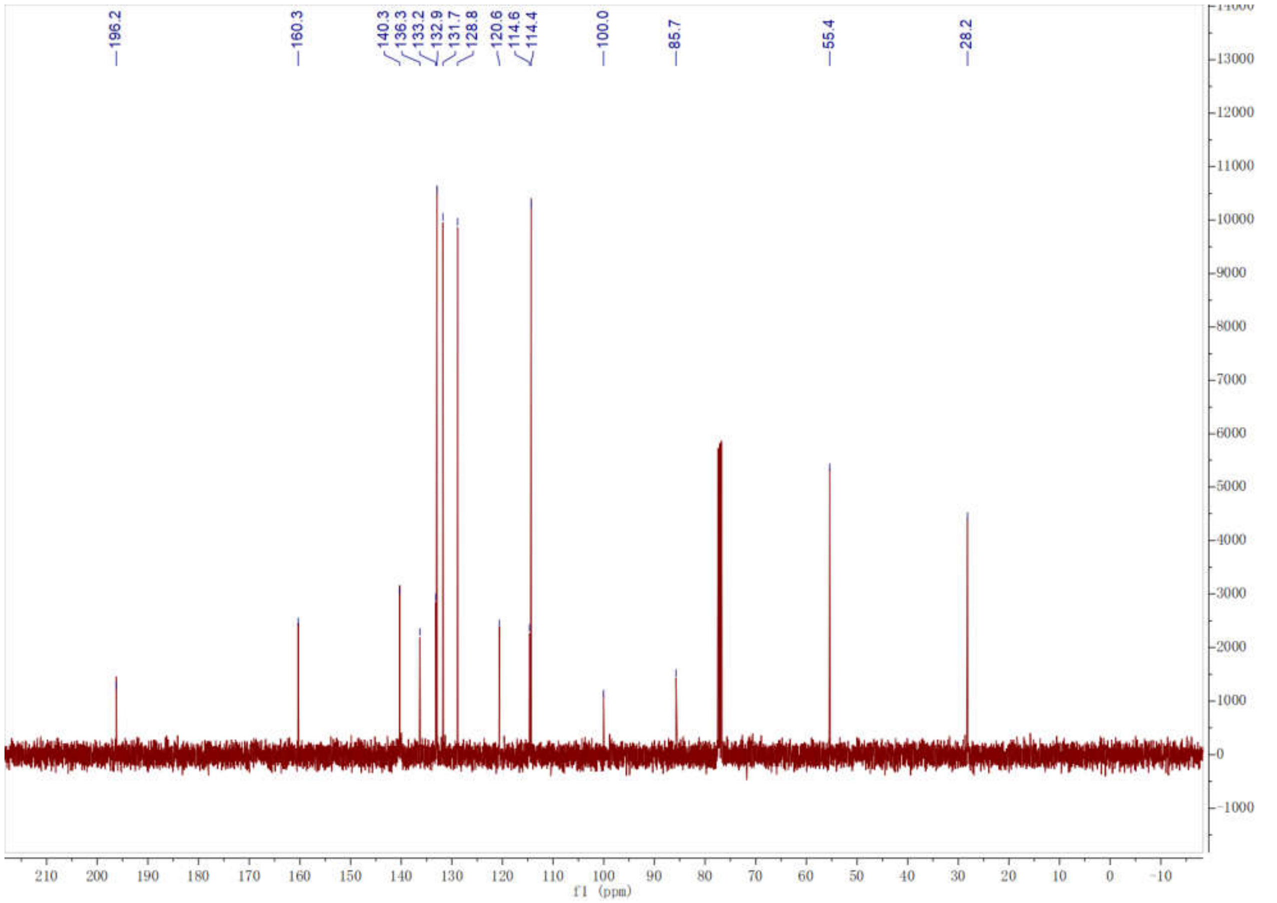
196.2
160.3
140.3
136.3
133.2
132.9
131.7
128.8
120.6
114.6
114.4
100.0
85.7
55.4
28.2
f1 (ppm)

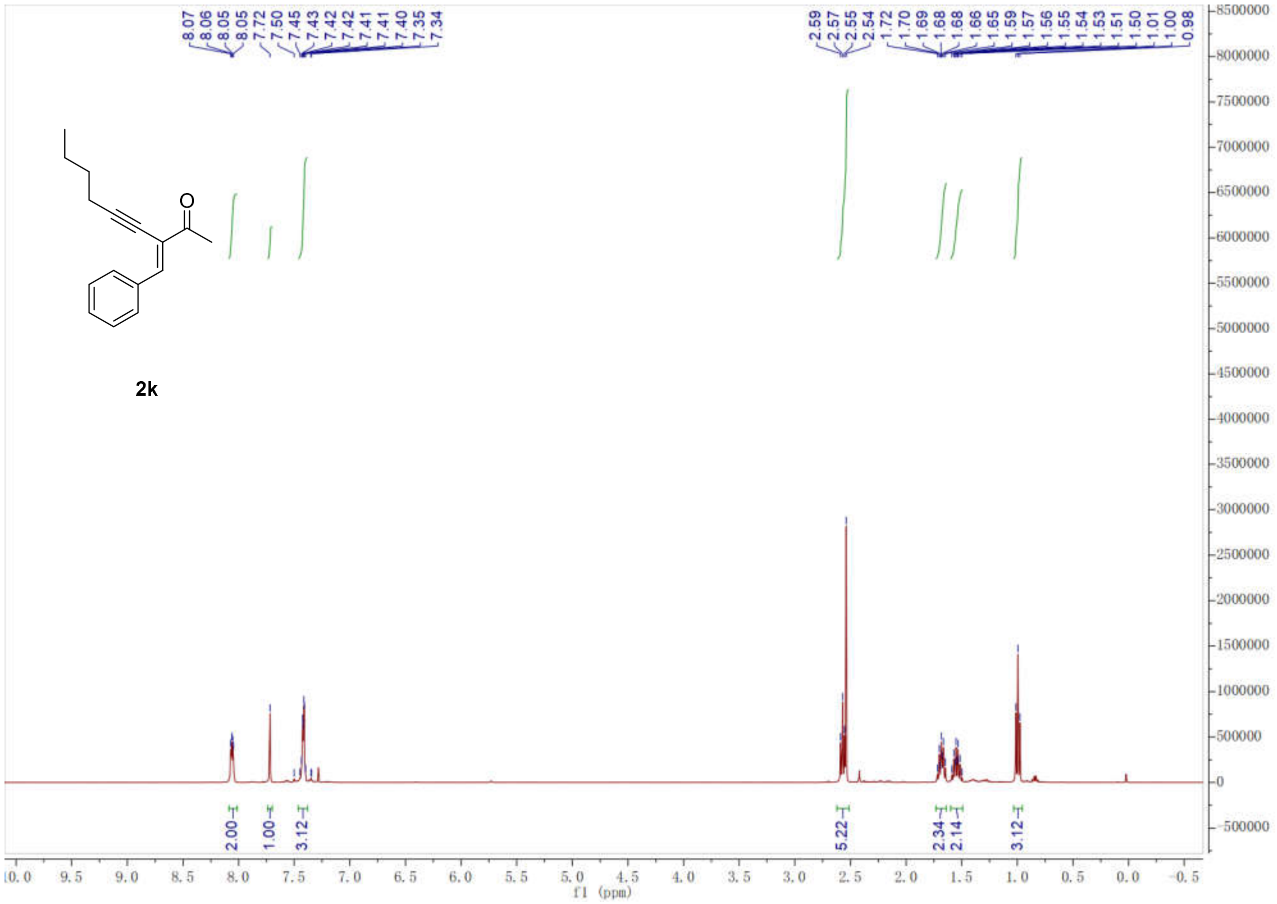
8.07
8.06
8.05
8.05
7.72
7.50
7.45
7.43
7.42
7.42
7.41
7.41
7.40
7.35
7.34
2.59
2.57
2.55
2.54
1.72
1.70
1.69
1.68
1.68
1.66
1.65
1.59
1.57
1.56
1.55
1.54
1.53
1.51
1.50
1.01
1.00
0.98
O
2k
2.00
1.00
3.12
5.22
2.34
2.14
3.12
f1 (ppm)

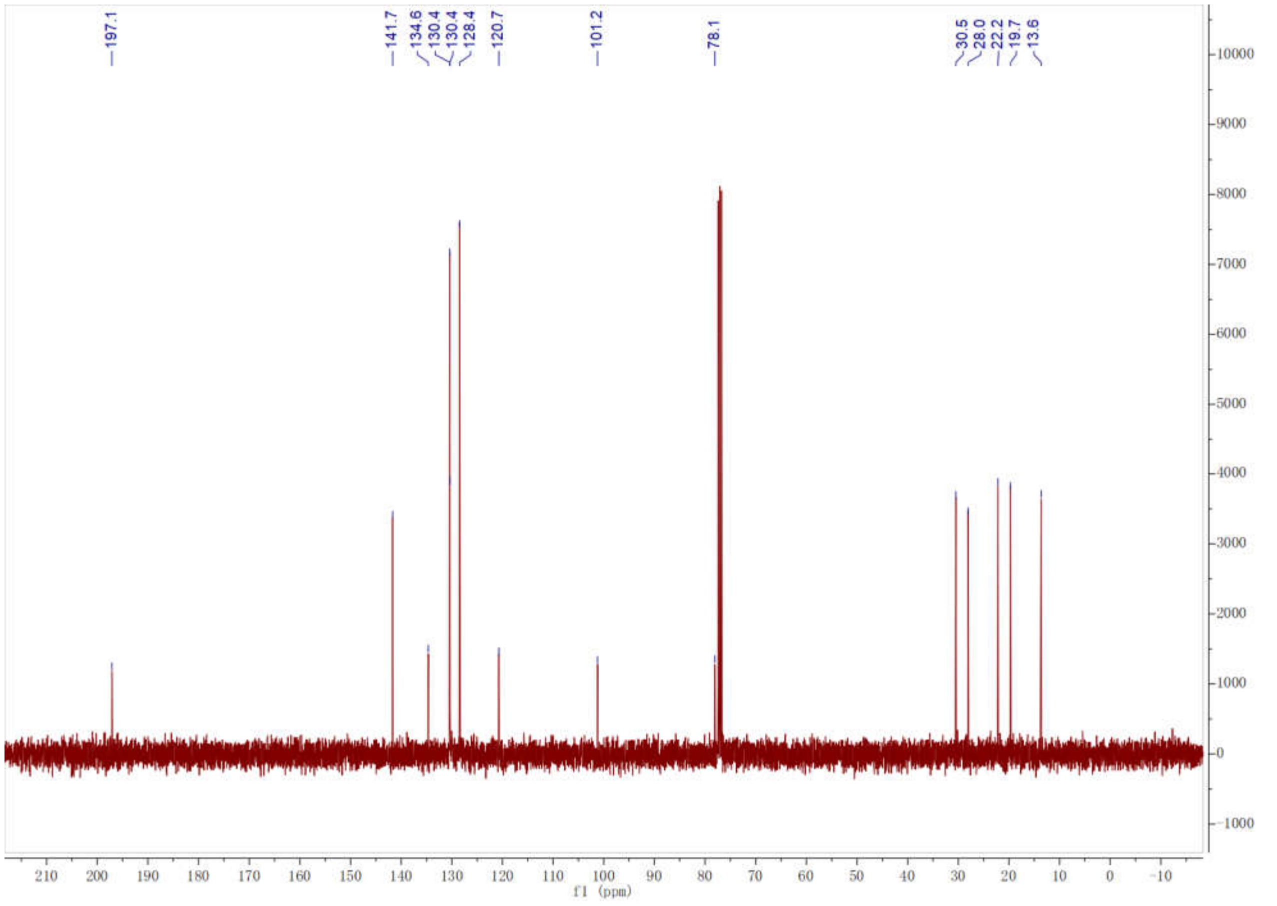
197.1
141.7
134.6
130.4
130.4
128.4
120.7
101.2
78.1
30.5
28.0
22.2
19.7
13.6
f1 (ppm)

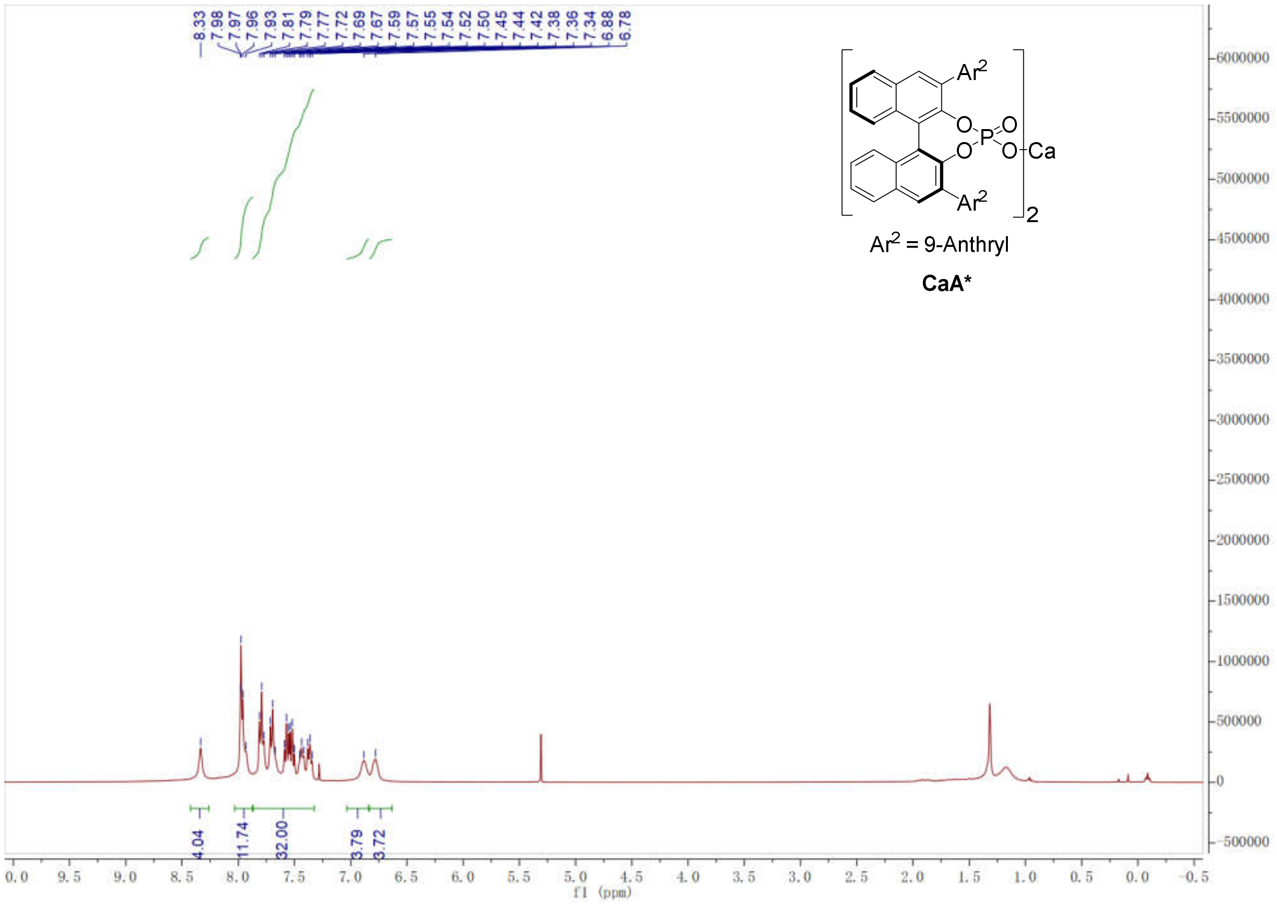
8.33 7.98 7.97 7.96 7.93 7.81 7.79 7.77 7.72 7.69 7.67 7.59 7.57 7.55 7.54 7.52 7.50 7.45 7.44 7.42 7.38 7.36 7.34 6.88 6.78
Ar2 = 9-Anthryl
CaA*
4.04 11.74 32.00 3.79 3.72
f1 (ppm)

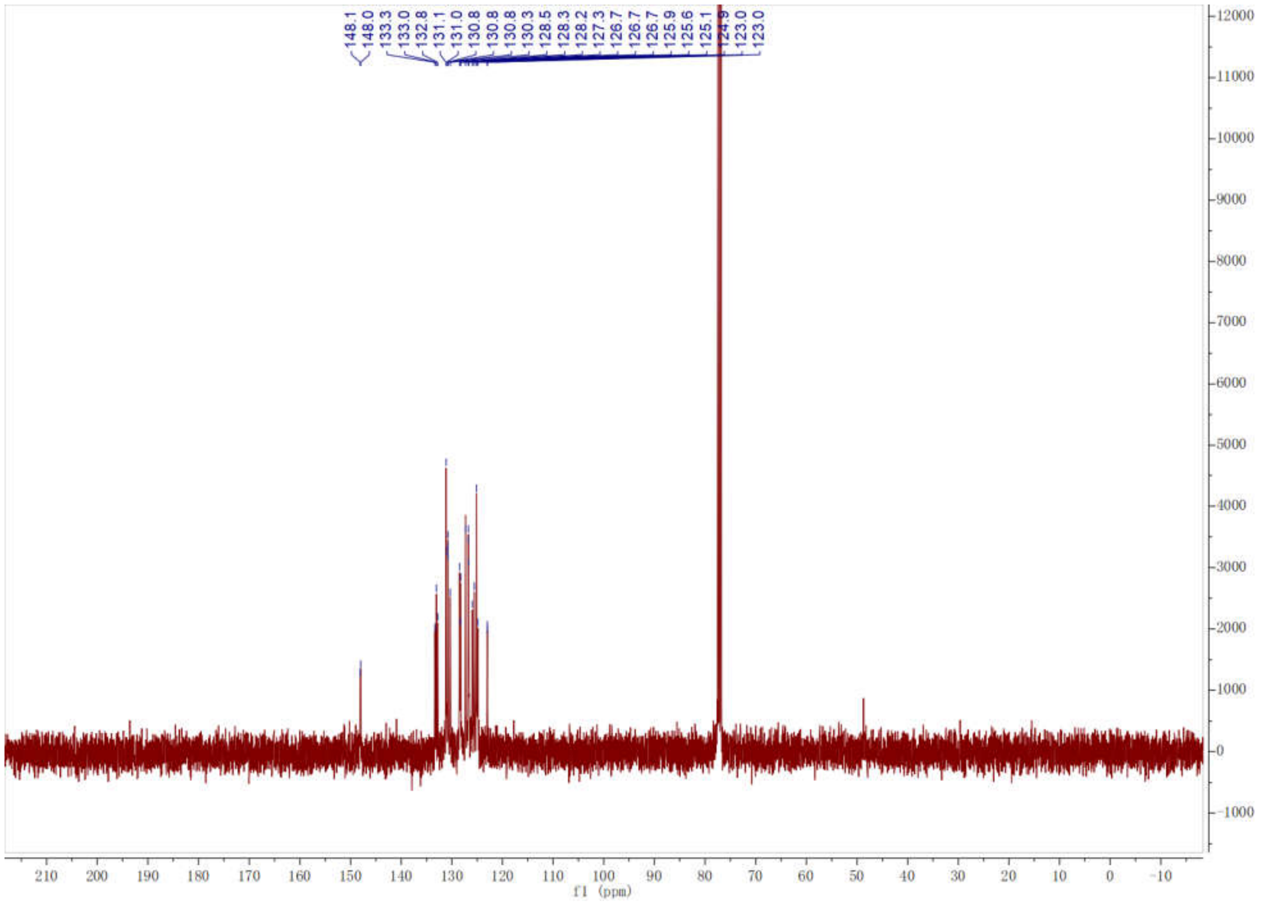
148.1 148.0 133.3 133.0 132.8 131.1 131.0 130.8 130.8 130.8 130.3 128.5 128.3 128.2 127.3 126.7 126.7 125.9 125.6 125.1 124.9 123.0 123.0
f1 (ppm)

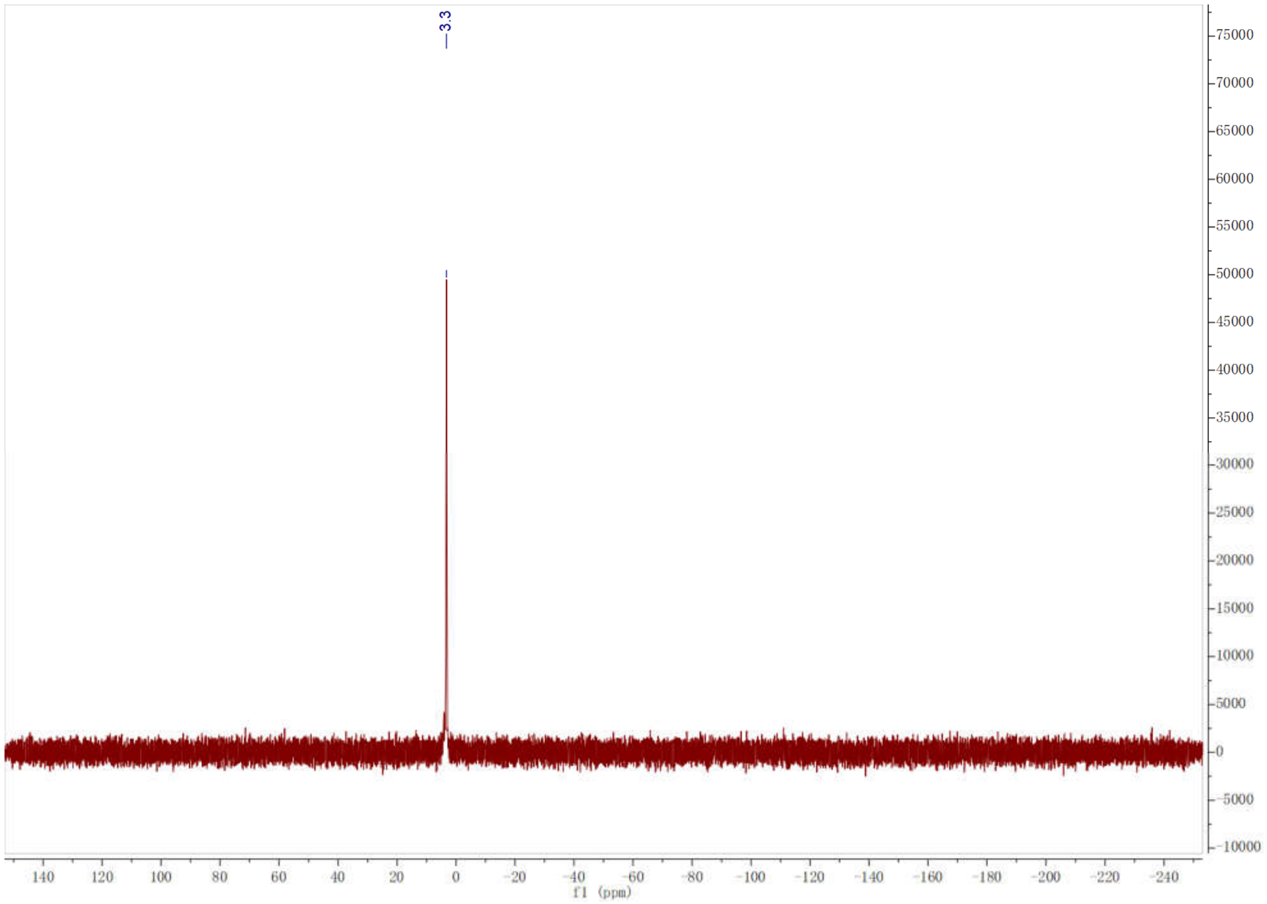
3.3
f1 (ppm)

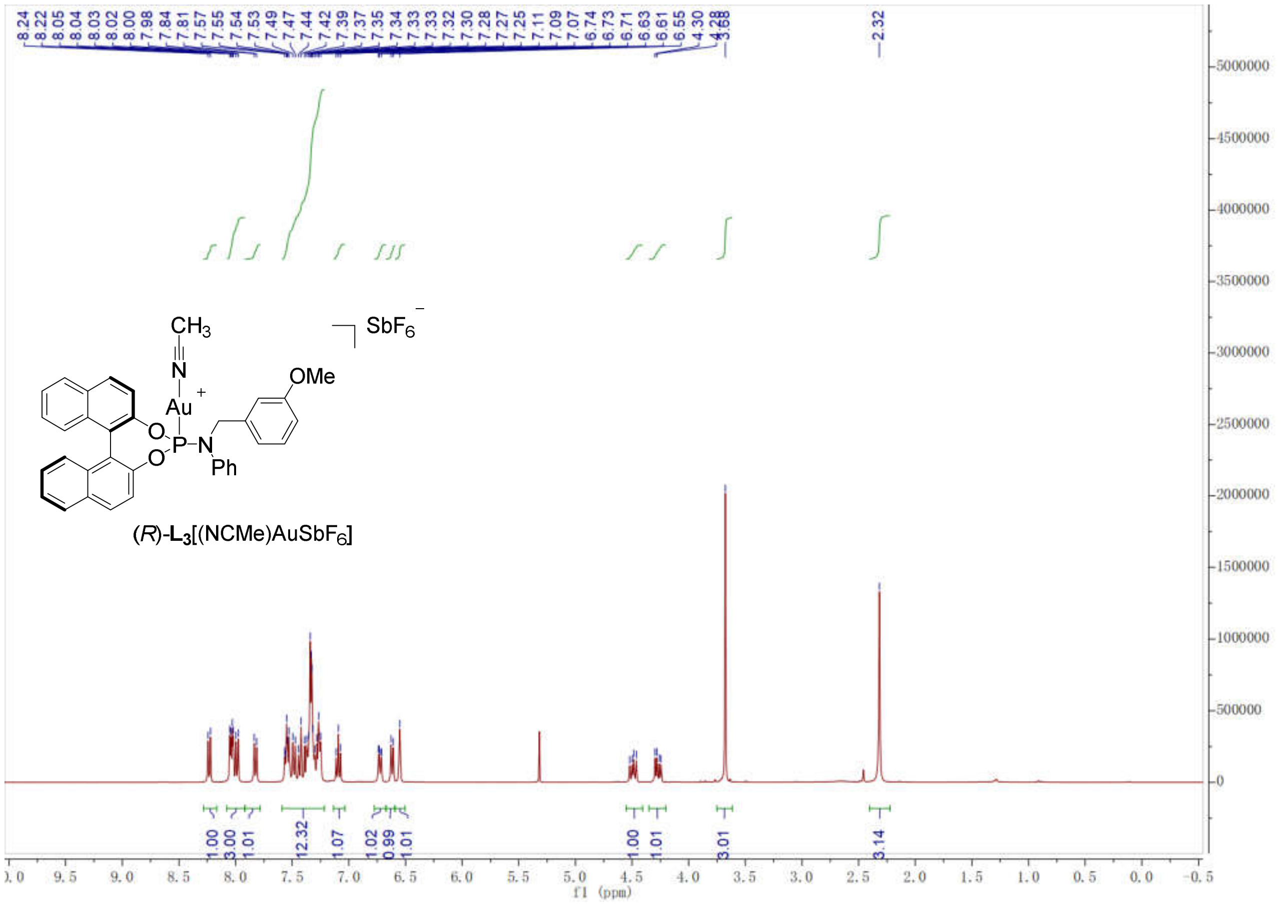
8.24 8.22 8.05 8.04 8.03 8.02 8.00 7.98 7.84 7.81 7.57 7.55 7.54 7.53 7.49 7.47 7.44 7.42 7.39 7.37 7.35 7.34 7.33 7.32 7.30 7.28 7.27 7.25 7.11 7.09 7.07 6.74 6.73 6.71 6.63 6.61 6.55 4.30 4.28 3.68
2.32
CH3
N
Au +
O
P–N
O
Ph
OMe
SbF6 −
(R)-L3[(NCMe)AuSbF6]
1.00 3.00 1.01 12.32 1.07 1.02 0.99 1.01 1.00 1.01 3.01 3.14
f1 (ppm)

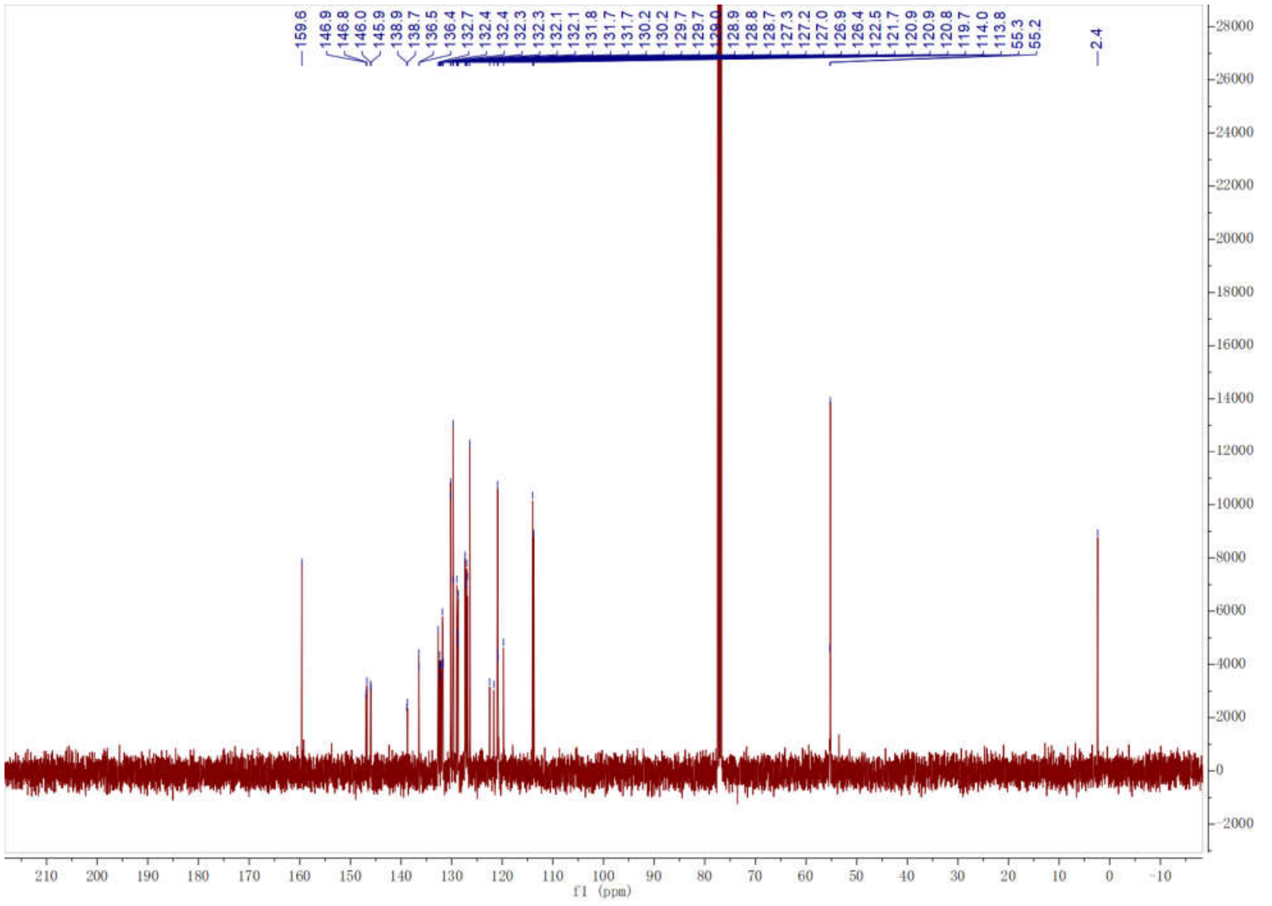
159.6 146.9 146.8 146.0 145.9 138.9 138.7 136.5 136.4 132.7 132.4 132.3 132.3 132.1 132.1 131.8 131.7 131.7 130.2 130.2 129.7 129.7 129.0 128.9 128.8 128.7 127.3 127.2 127.0 126.9 126.4 122.5 121.7 120.9 120.9 120.8 119.7 114.0 113.8 55.3 55.2
2.4
f1 (ppm)

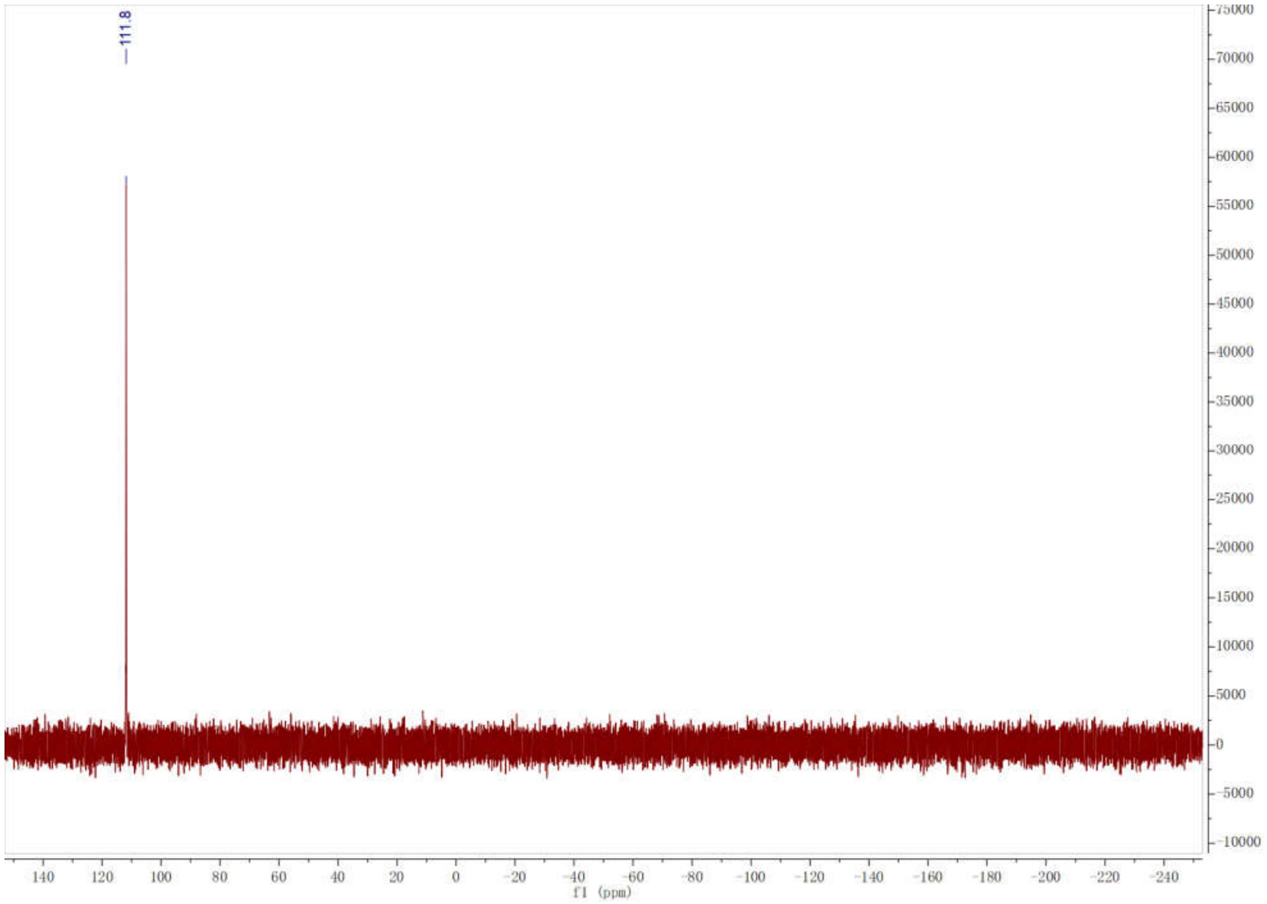

111.8
75000
70000
65000
60000
55000
50000
45000
40000
35000
30000
25000
20000
15000
10000
5000
0
-5000
-10000
140 120 100 80 60 40 20 0 -20 -40 -60 -80 -100 -120 -140 -160 -180 -200 -220 -240
f1 (ppm)

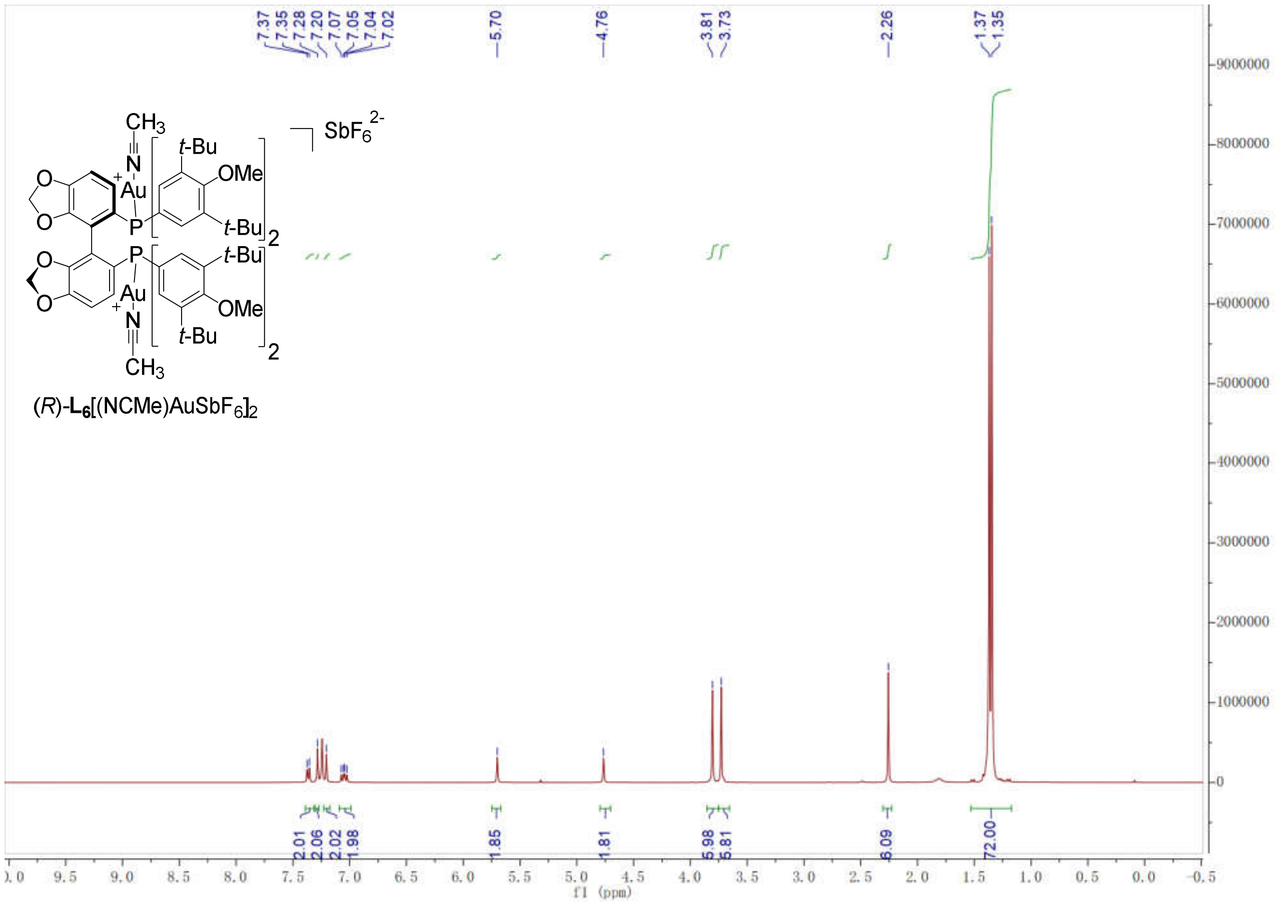
(R)-L6[(NCMe)AuSbF6]2
SbF6 2-
f1 (ppm)

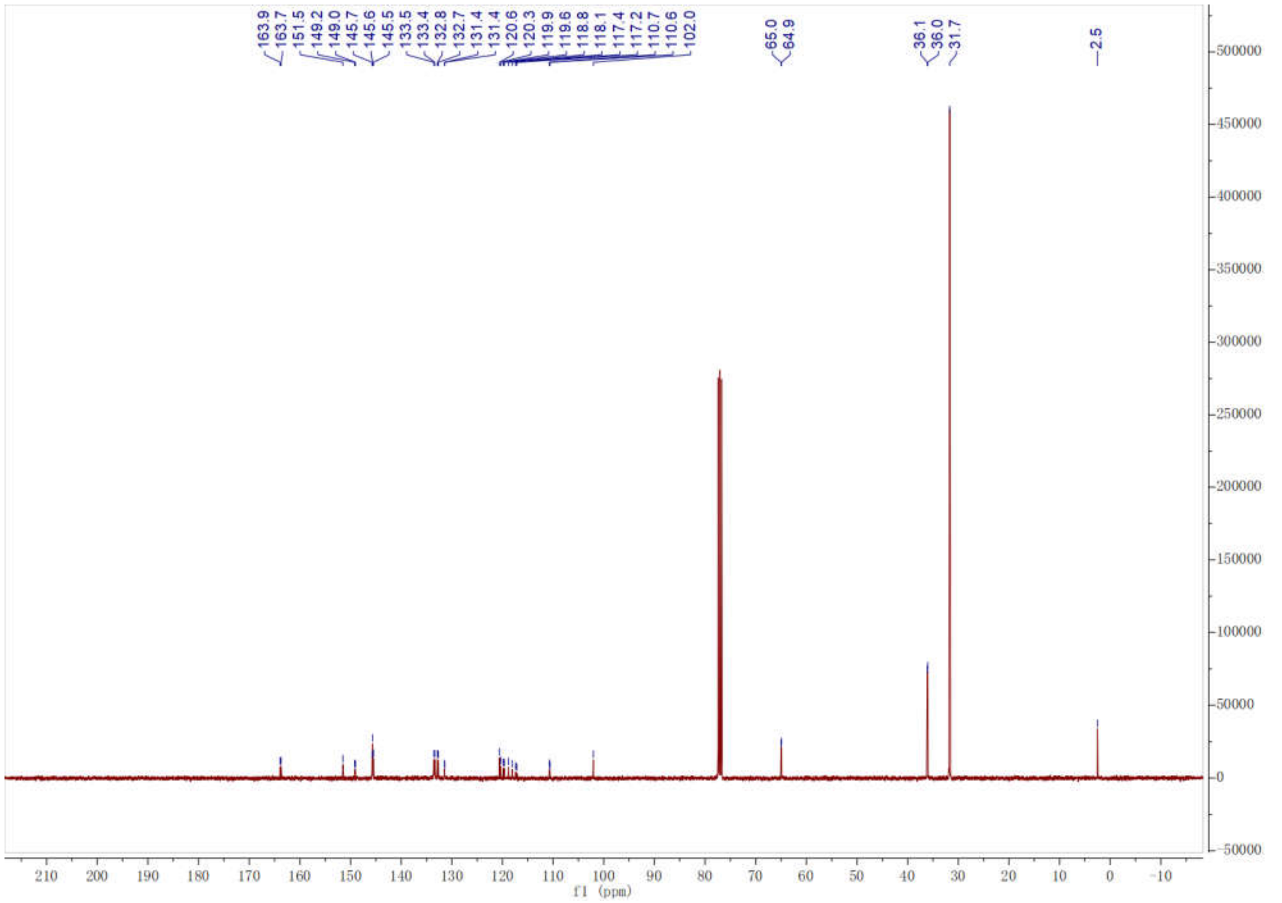
f1 (ppm)

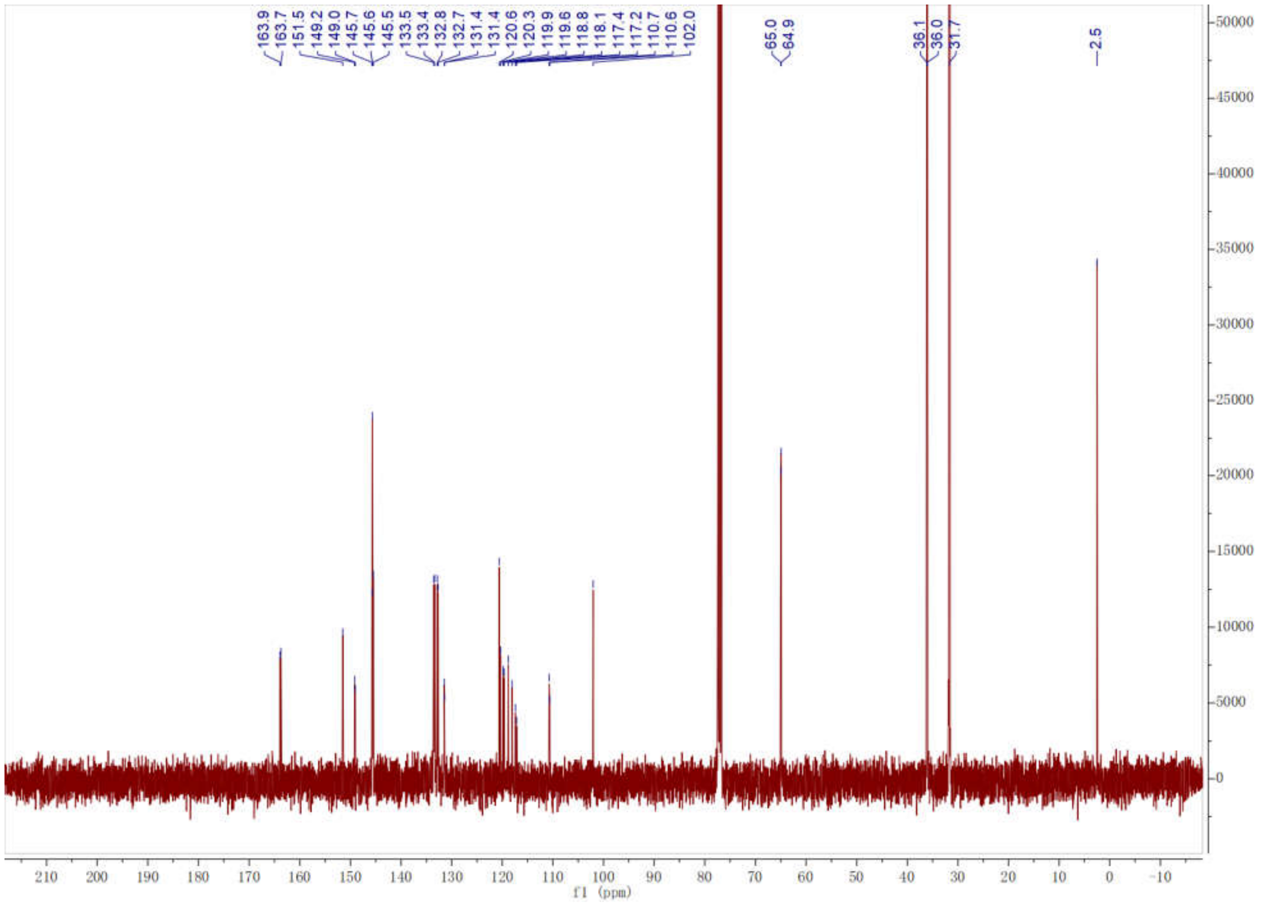
163.9
163.7
151.5
149.2
149.0
145.7
145.6
145.5
133.5
133.4
132.8
132.7
131.4
131.4
120.6
120.3
119.9
119.6
118.8
118.1
117.4
117.2
110.7
110.6
102.0
65.0
64.9
36.1
36.0
31.7
2.5
f1 (ppm)

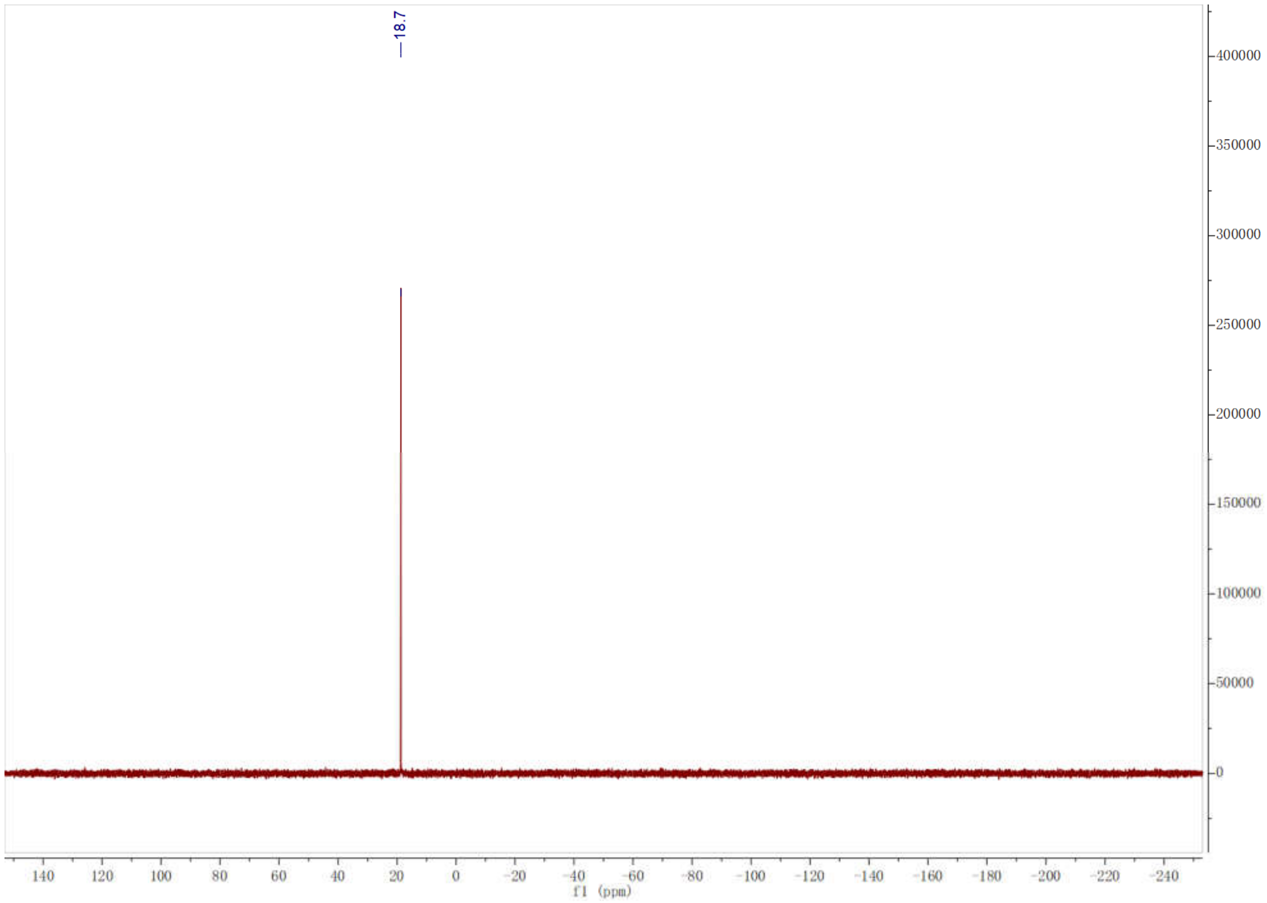
18.7
f1 (ppm)

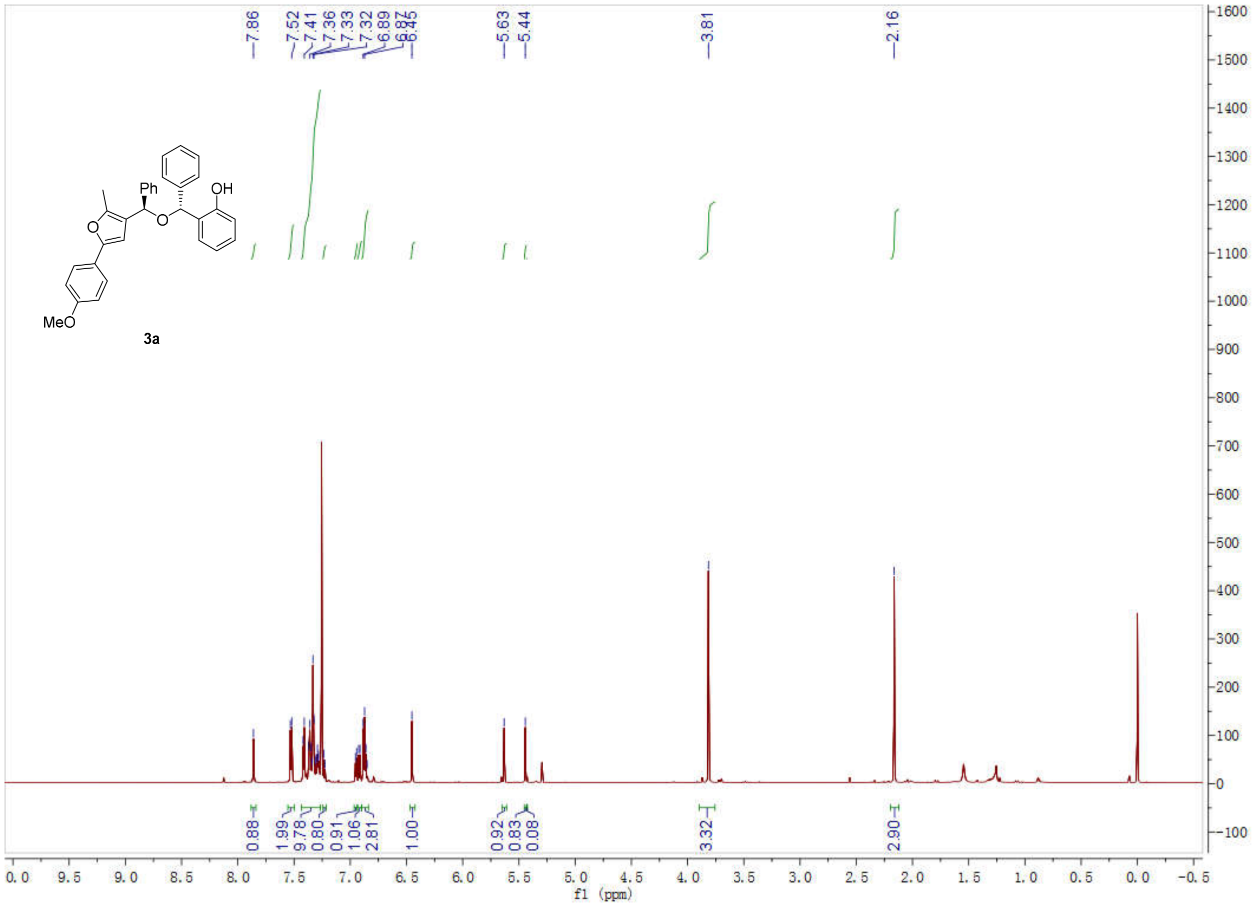
7.86
7.52
7.41
7.36
7.33
7.32
6.89
6.87
6.45
5.63
5.44
3.81
2.16
Ph
OH
O
MeO
3a
0.88
1.99
9.78
0.80
0.91
1.06
2.81
1.00
0.92
0.83
0.08
3.32
2.90
f1 (ppm)

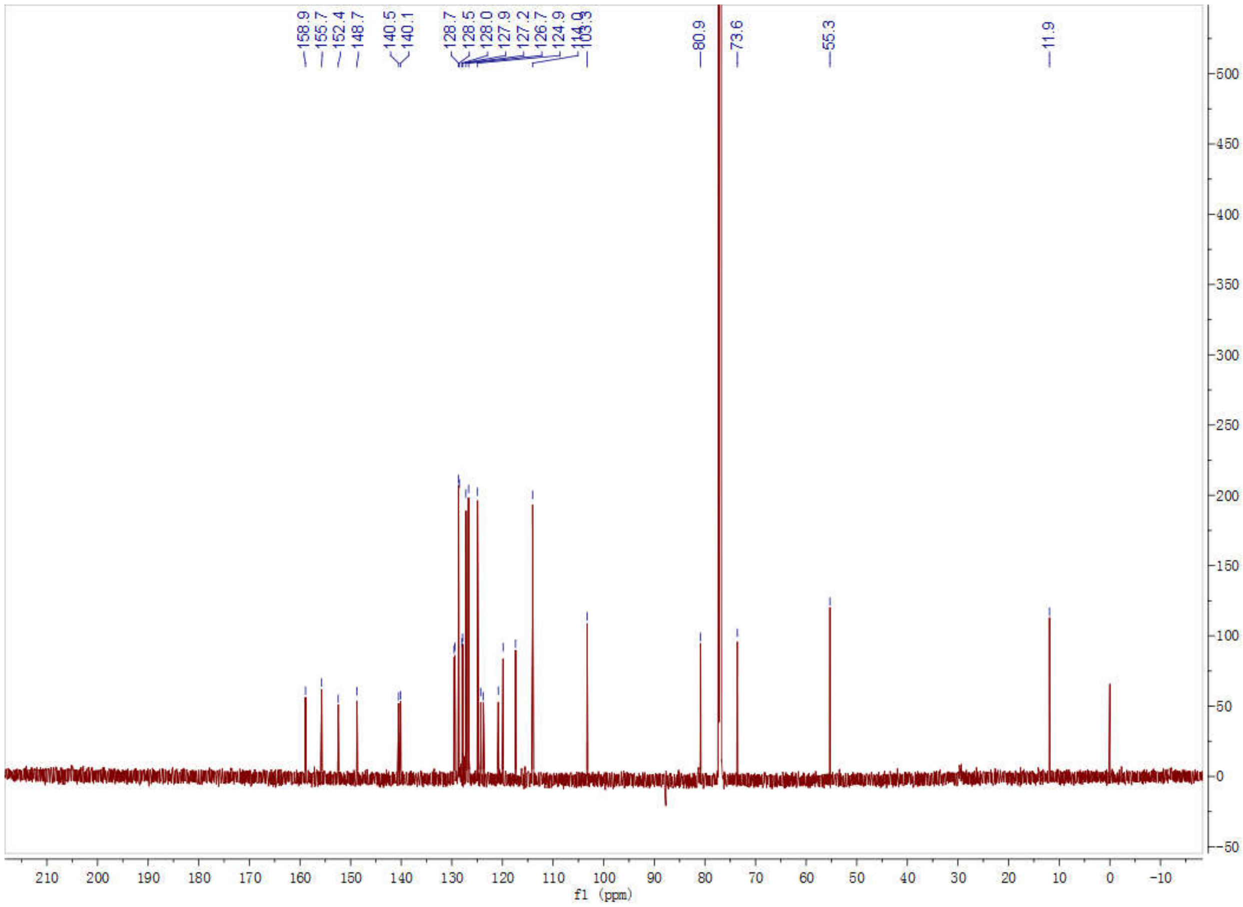
158.9
155.7
152.4
148.7
140.5
140.1
128.7
128.5
128.0
127.9
127.2
126.7
124.9
103.3
80.9
73.6
55.3
11.9
f1 (ppm)

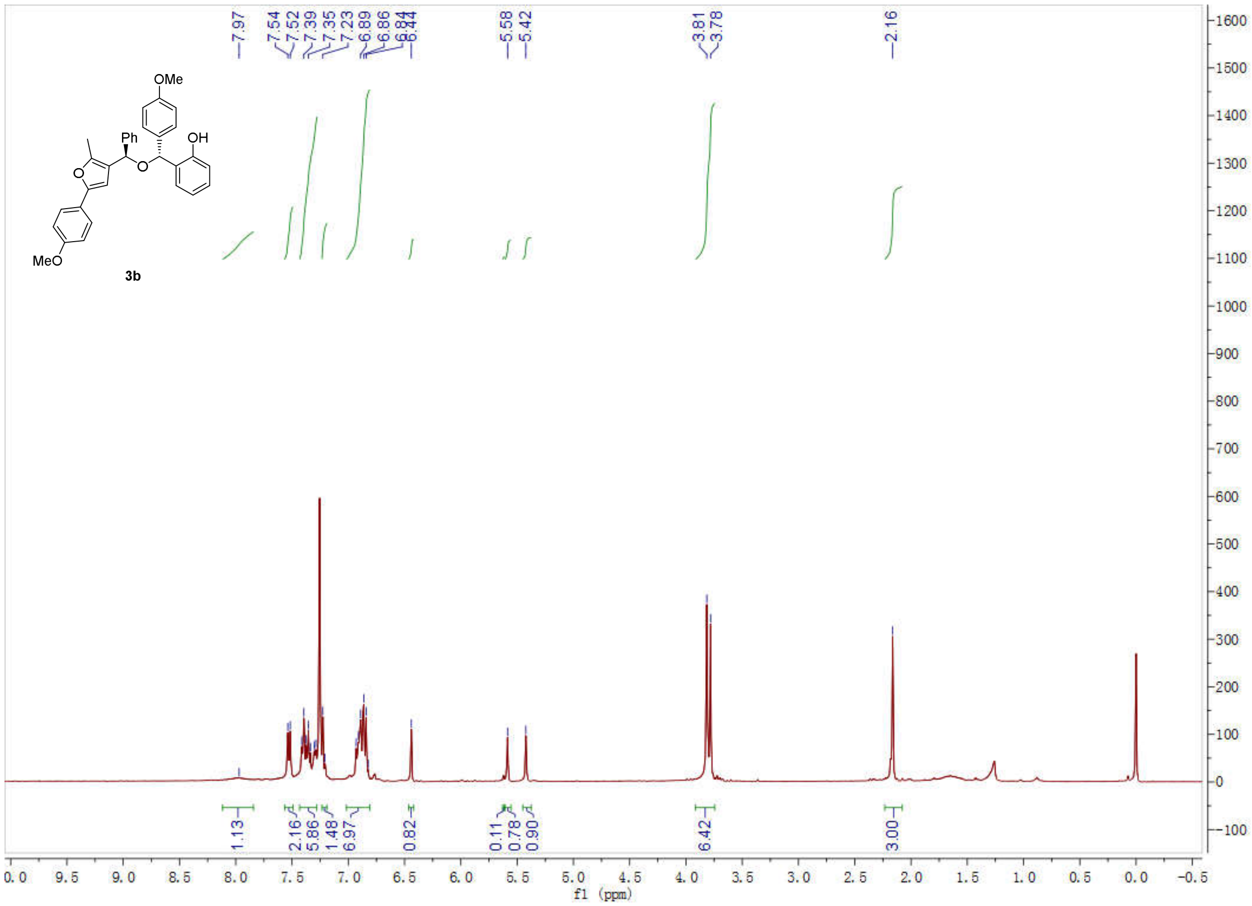

7.97
7.54
7.52
7.39
7.35
7.23
6.89
6.86
6.44
5.58
5.42
3.81
3.78
2.16
OMe
Ph
OH
O
MeO
3b
1.13
2.16
5.86
1.48
6.97
0.82
0.11
0.78
0.90
6.42
3.00
f1 (ppm)

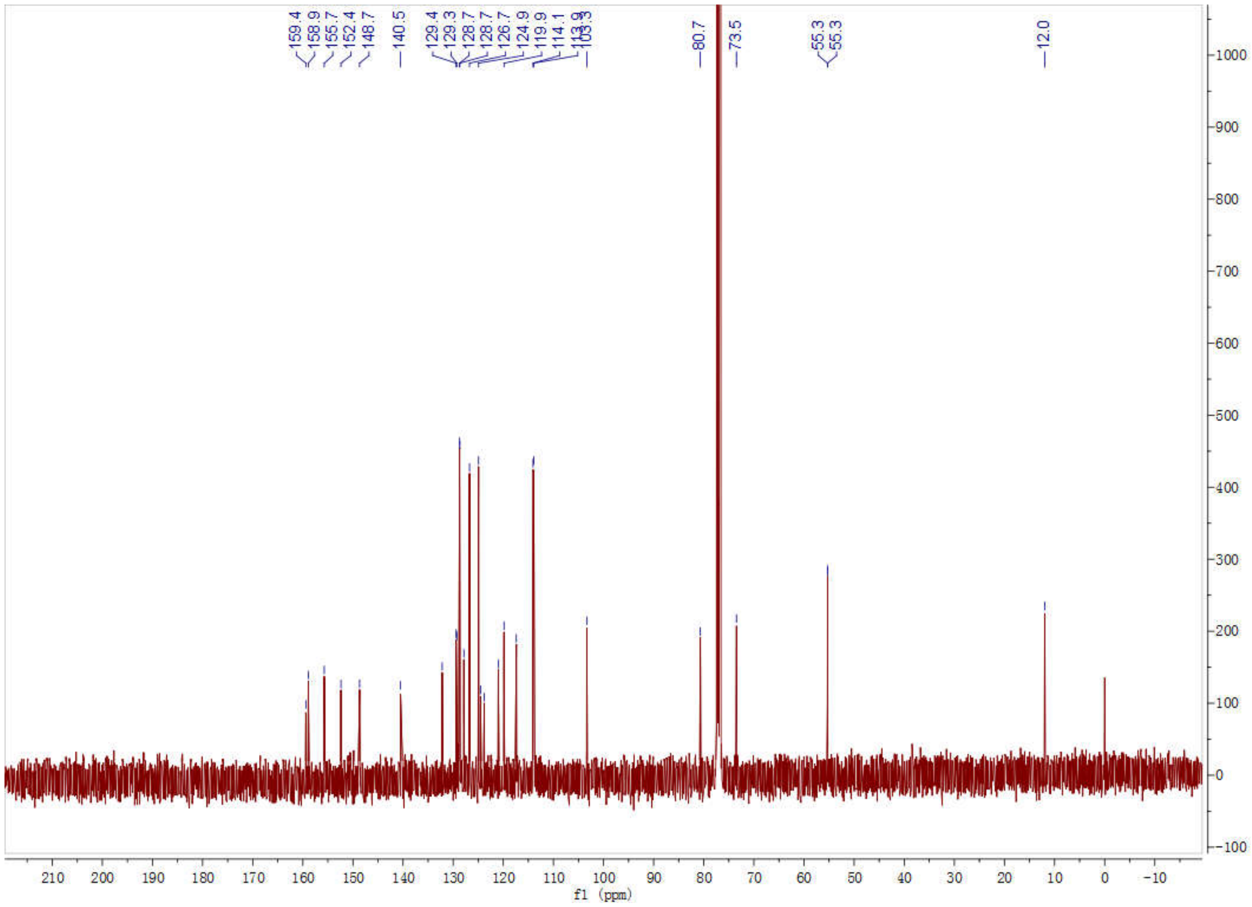

159.4
158.9
155.7
152.4
148.7
140.5
129.4
129.3
128.7
128.7
126.7
124.9
119.9
114.1
103.3
80.7
73.5
55.3
55.3
12.0
f1 (ppm)

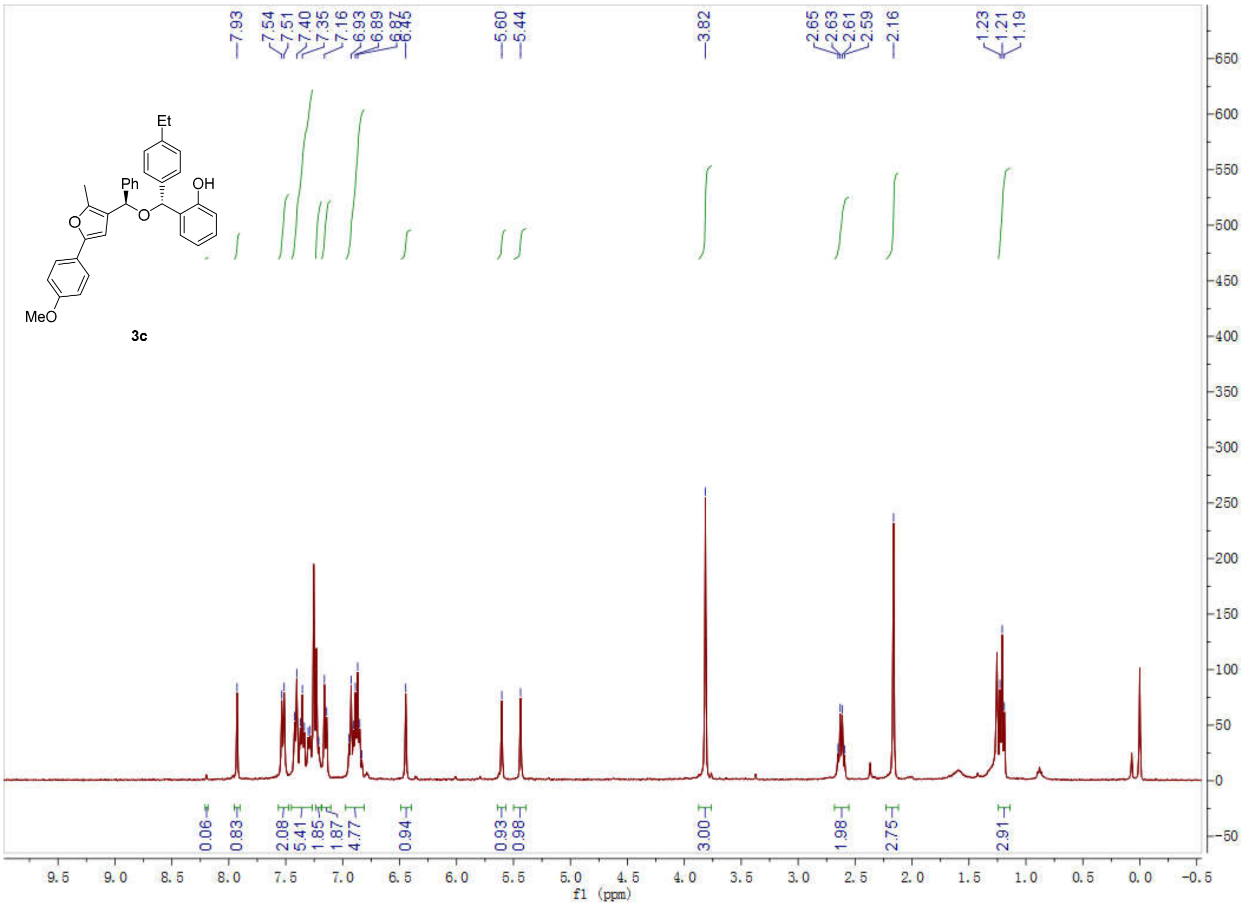
7.93
7.54
7.51
7.40
7.35
7.16
6.93
6.89
6.87
6.45
5.60
5.44
3.82
2.65
2.63
2.61
2.59
2.16
1.23
1.21
1.19
Et
Ph
OH
O
O
MeO
3c
0.06
0.83
2.08
5.41
1.85
1.87
4.77
0.94
0.93
0.98
3.00
1.98
2.75
2.91
f1 (ppm)

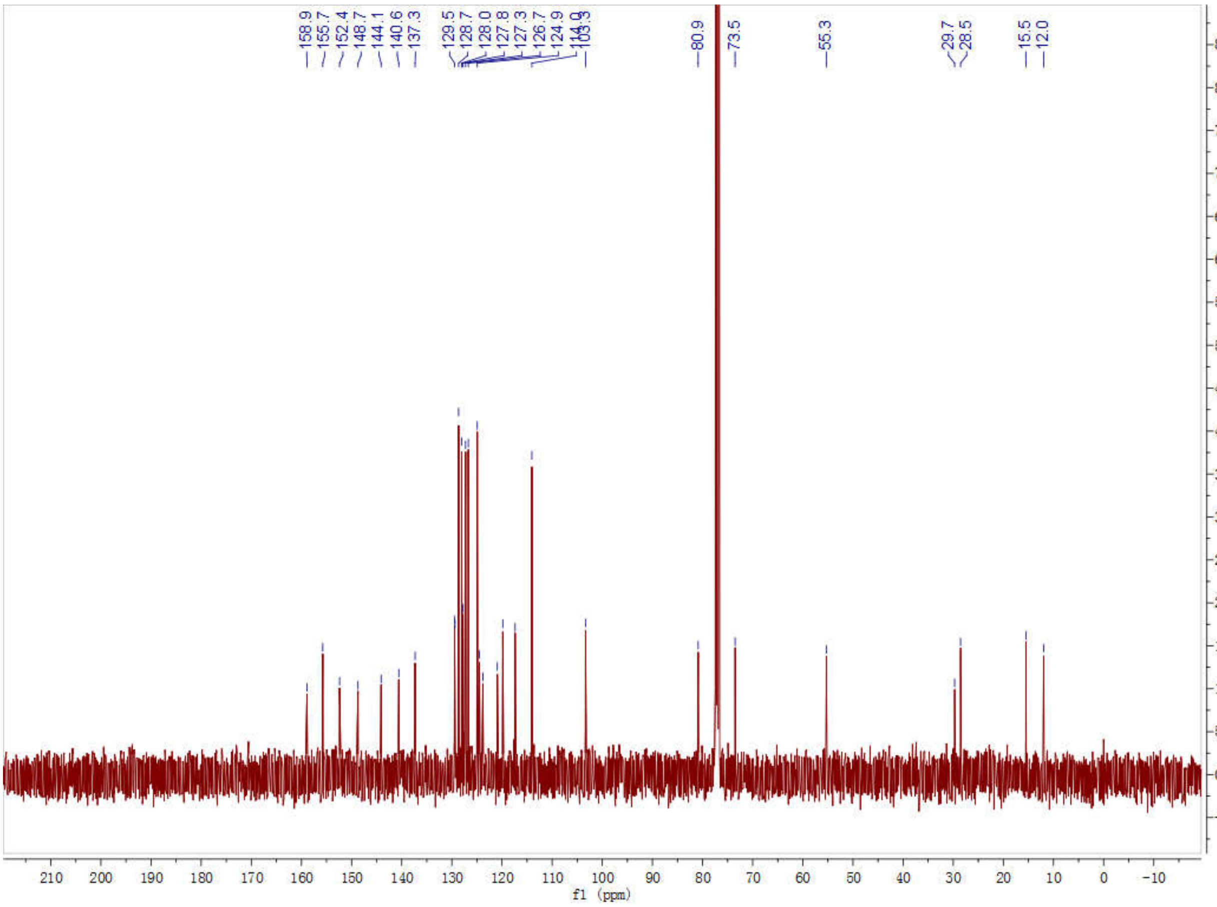
158.9
155.7
152.4
148.7
144.1
140.6
137.3
129.5
128.7
128.0
127.8
127.3
126.7
124.9
114.0
103.3
80.9
73.5
55.3
29.7
28.5
15.5
12.0
f1 (ppm)

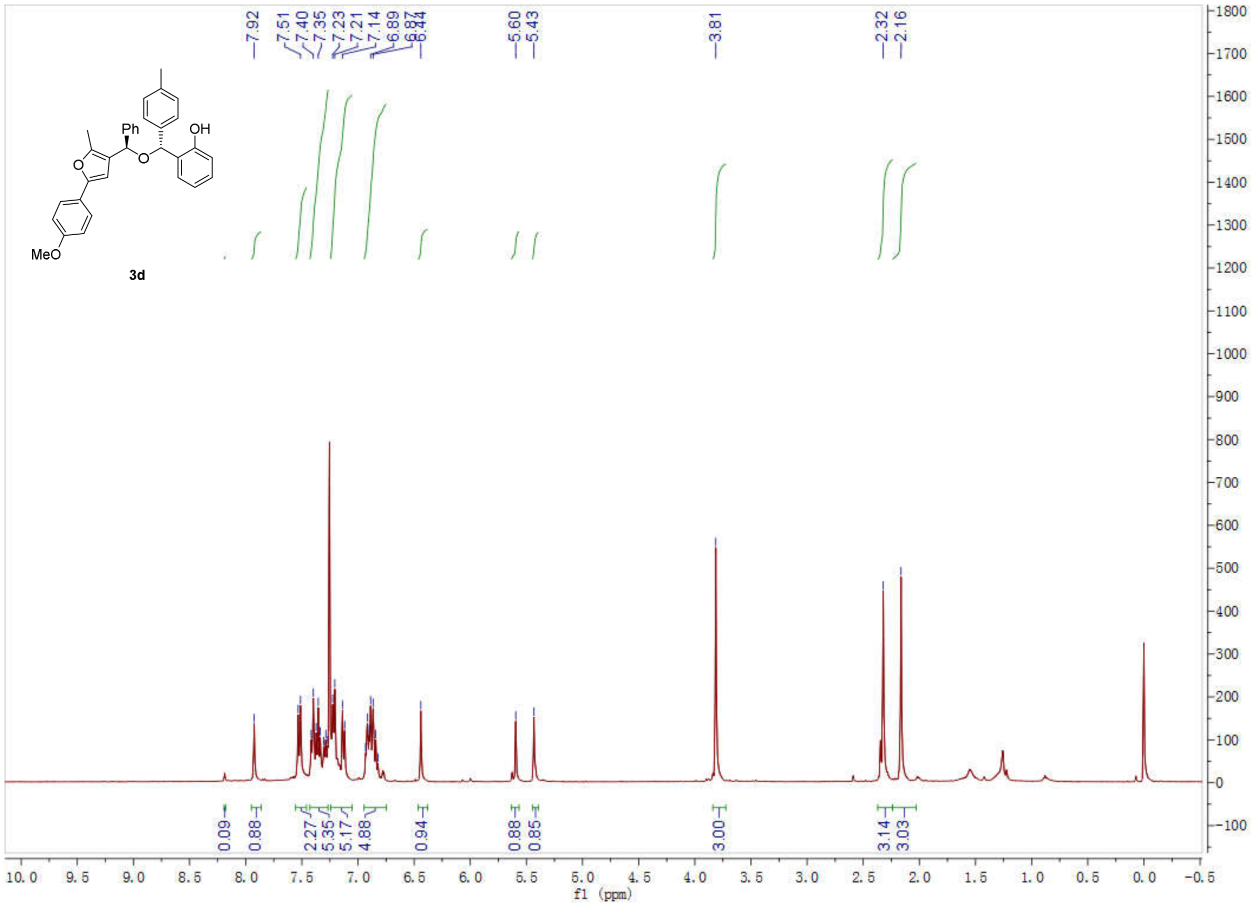
Ph
OH
O
MeO
3d
7.92
7.51
7.40
7.35
7.23
7.21
7.14
6.89
6.87
6.44
5.60
5.43
3.81
2.32
2.16
0.09
0.88
2.27
5.35
5.17
4.88
0.94
0.88
0.85
3.00
3.14
3.03
f1 (ppm)

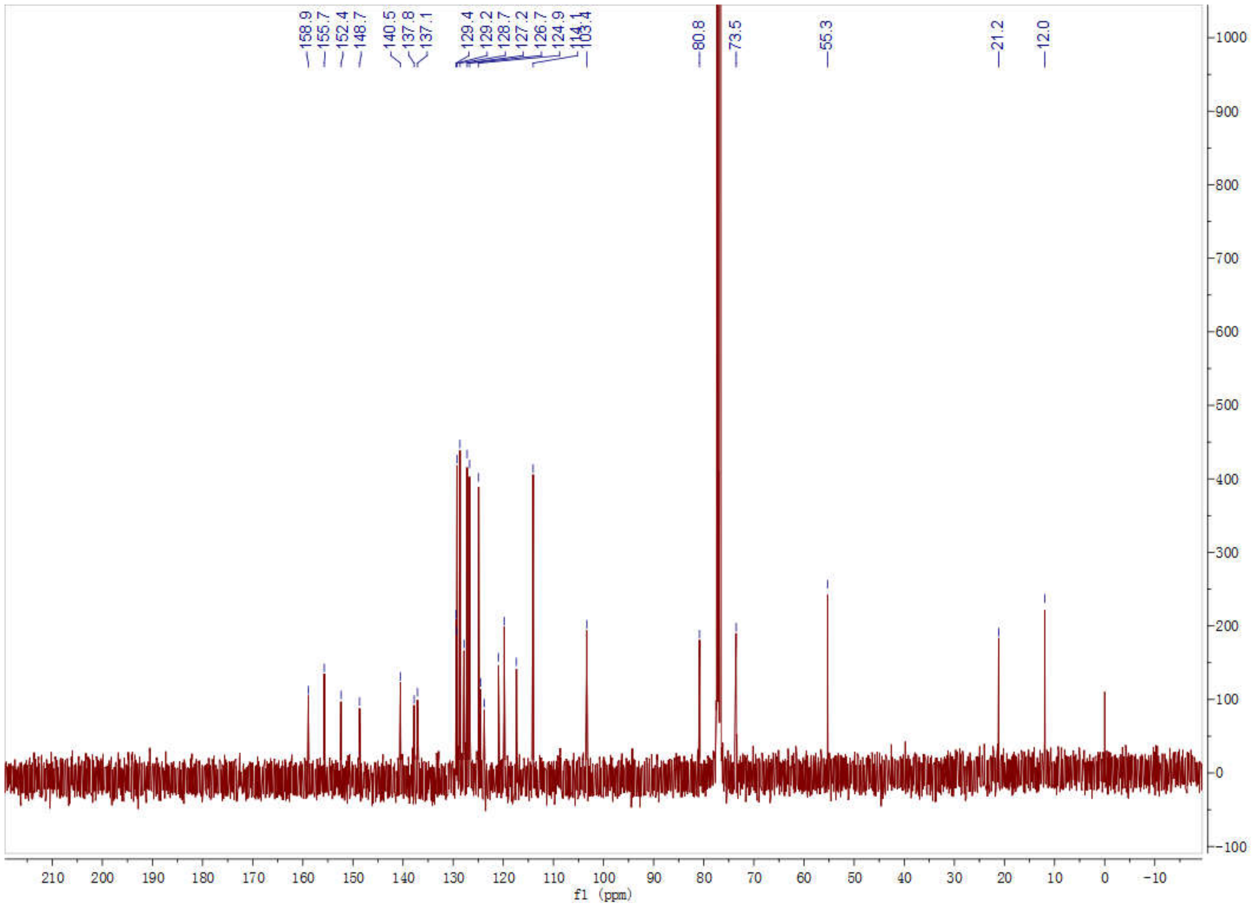
158.9
155.7
152.4
148.7
140.5
137.8
137.1
129.4
129.2
128.7
127.2
126.7
124.9
114.1
103.4
80.8
73.5
55.3
21.2
12.0
f1 (ppm)

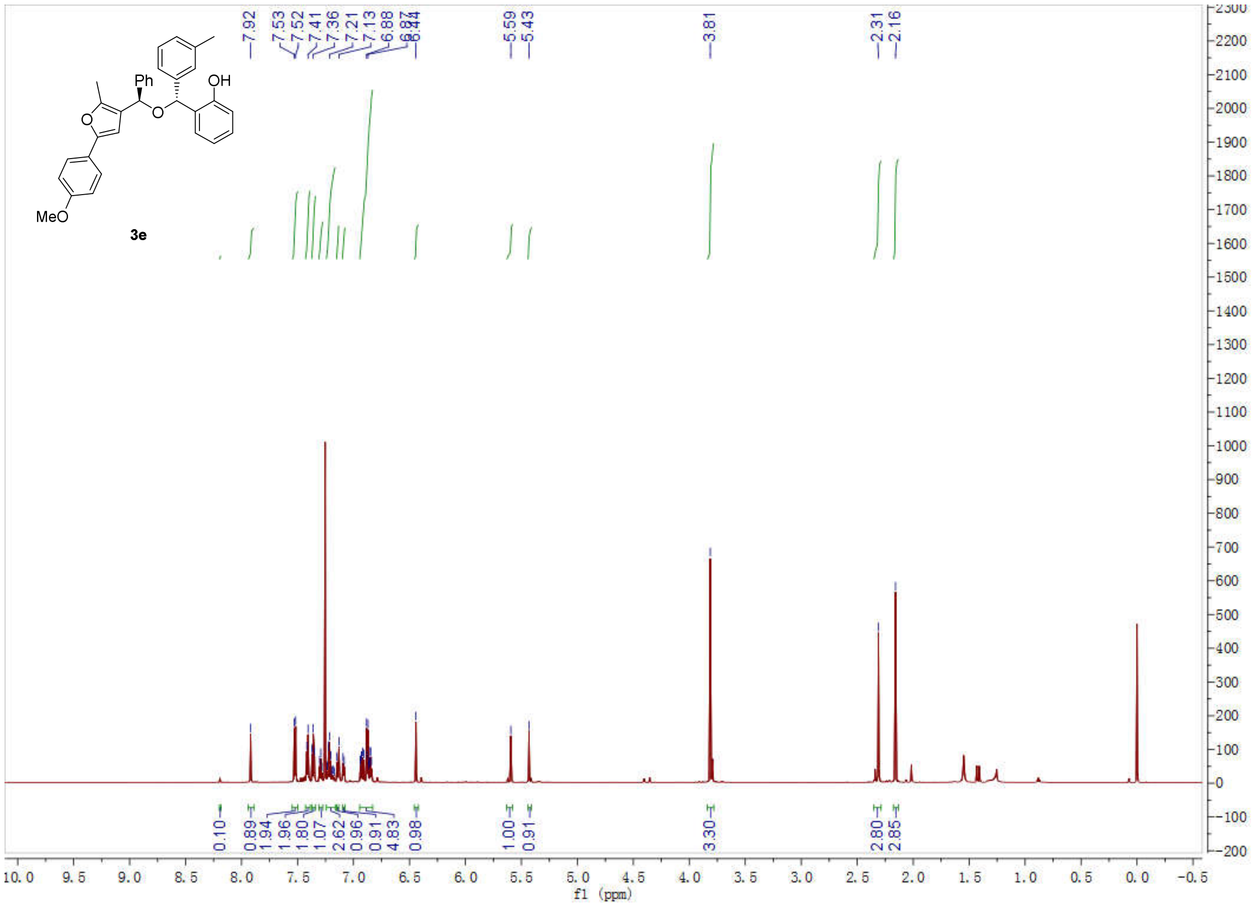

Ph
OH
O
MeO
3e
7.92
7.53
7.52
7.41
7.36
7.21
7.13
6.88
5.59
5.43
3.81
2.31
2.16
0.10
0.89
1.94
1.96
1.80
1.07
2.62
0.96
0.91
4.83
0.98
1.00
0.91
3.30
2.80
2.85
f1 (ppm)

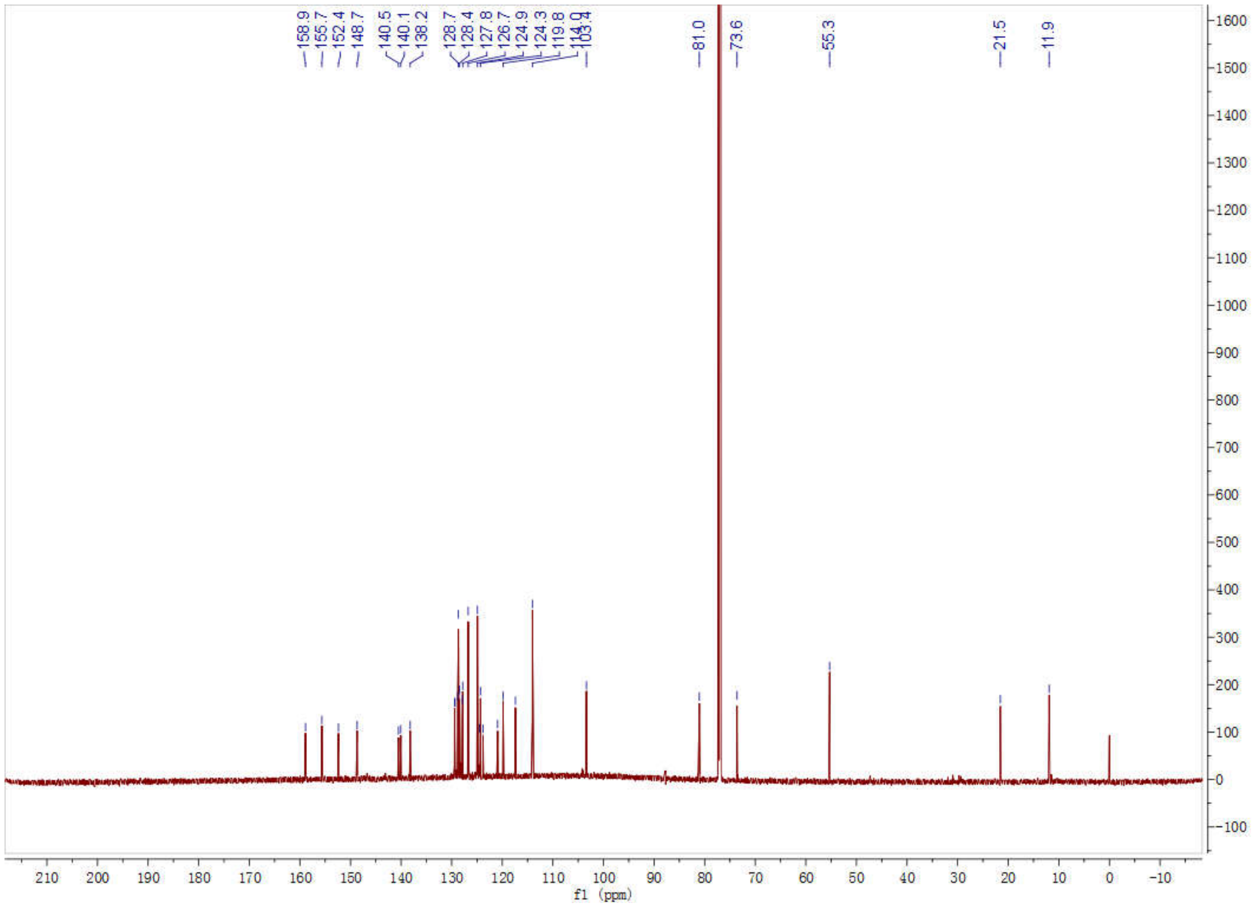

158.9
155.7
152.4
148.7
140.5
140.1
138.2
128.7
128.4
127.8
126.7
124.9
124.3
119.8
114.0
103.4
81.0
73.6
55.3
21.5
11.9
f1 (ppm)

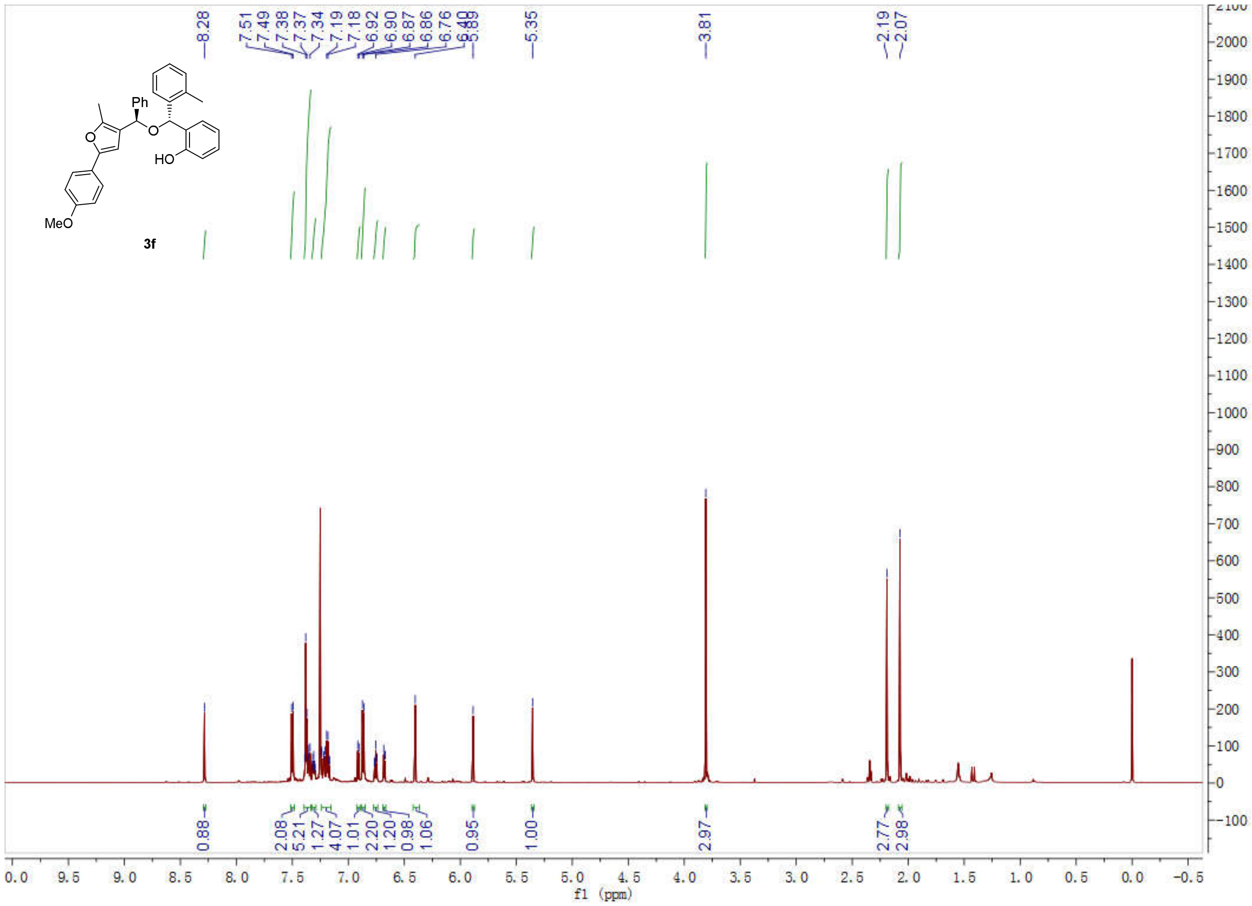
8.28
7.51
7.49
7.38
7.37
7.34
7.19
7.18
6.92
6.90
6.87
6.86
6.76
6.40
5.89
5.35
3.81
2.19
2.07
Ph
O
HO
MeO
3f
0.88
2.08
5.21
1.27
4.07
1.01
2.20
1.20
0.98
1.06
0.95
1.00
2.97
2.77
2.98
f1 (ppm)

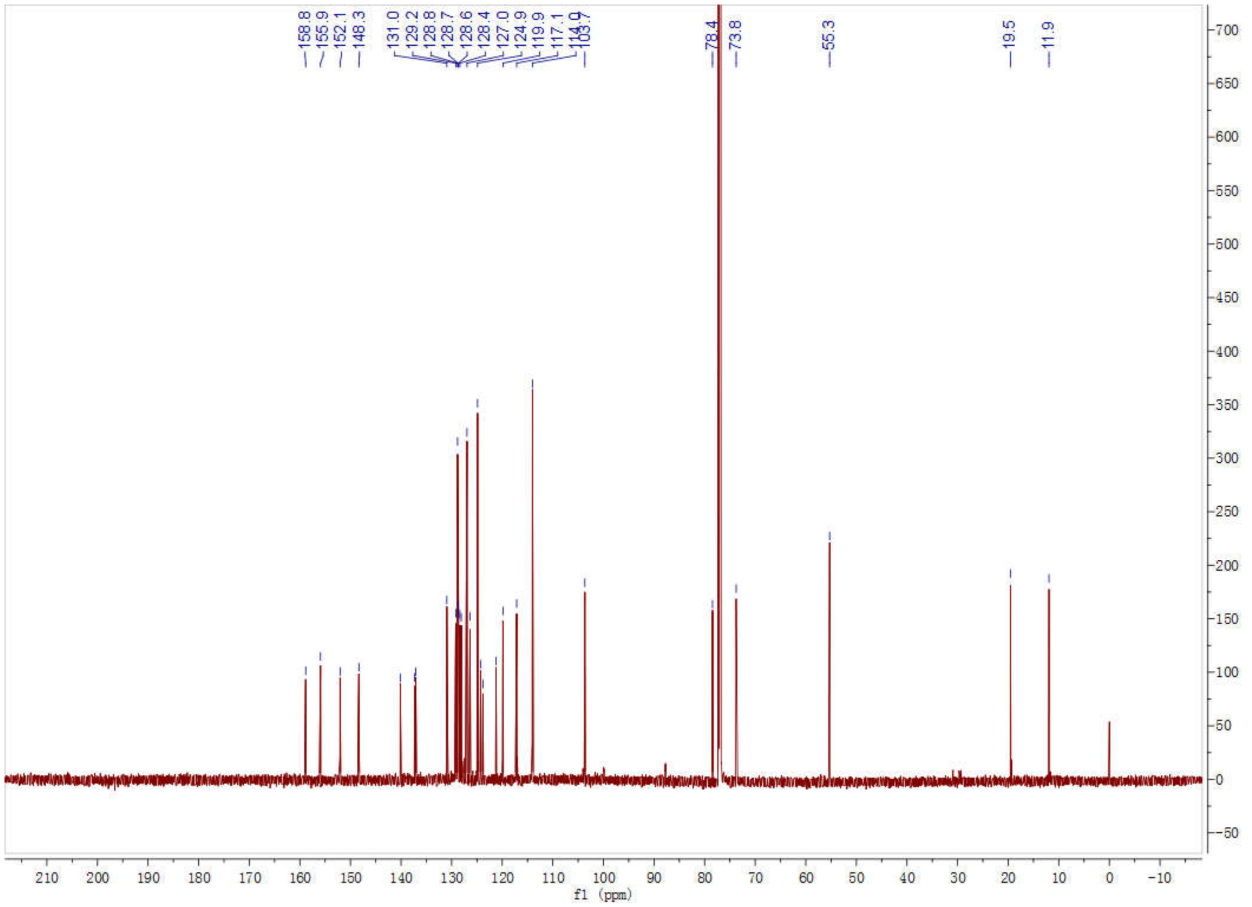
158.8
155.9
152.1
148.3
131.0
129.2
128.8
128.7
128.6
128.4
127.0
124.9
119.9
117.1
114.0
103.7
78.4
73.8
55.3
19.5
11.9
f1 (ppm)

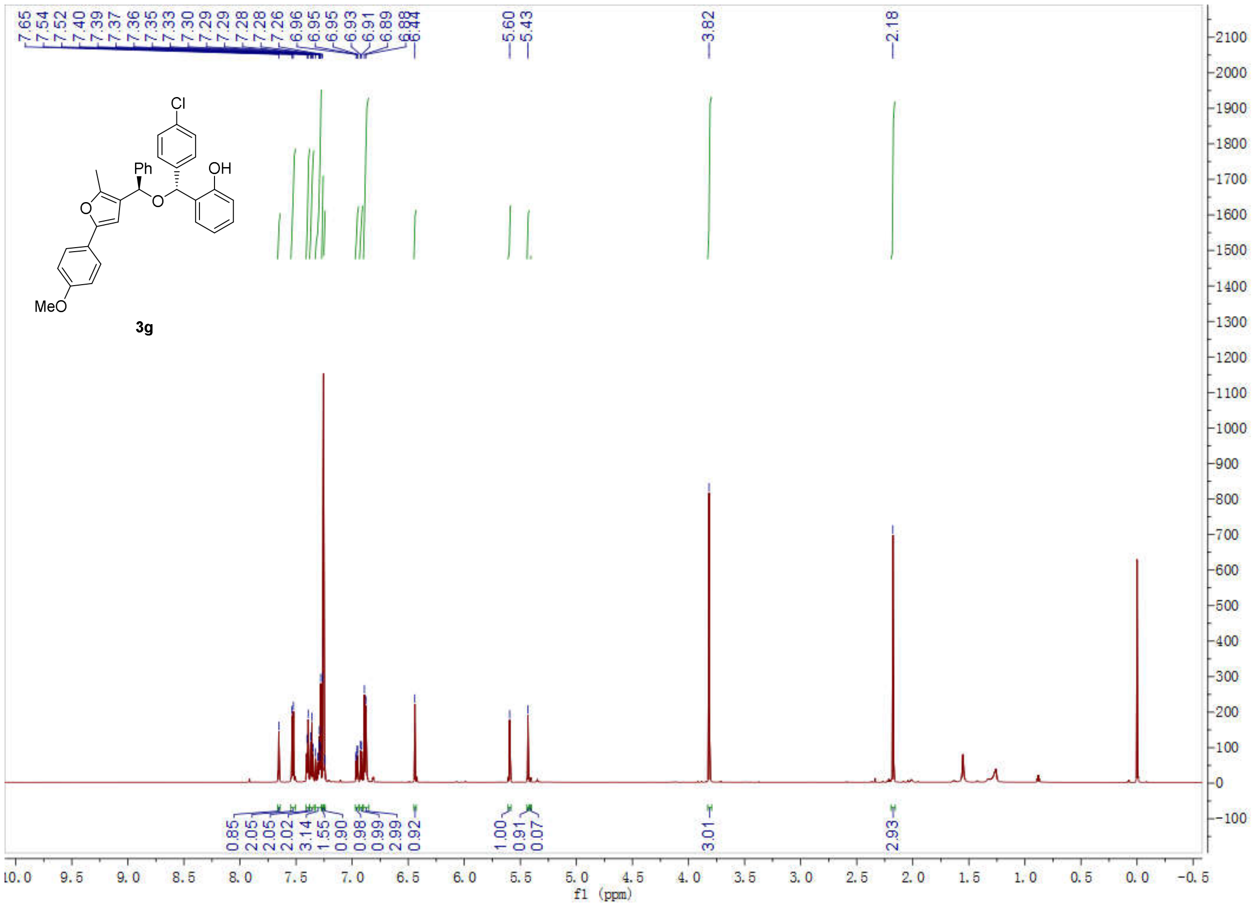

3g
MeO
Ph
OH
Cl
O
7.65 7.54 7.52 7.40 7.39 7.37 7.36 7.35 7.33 7.30 7.29 7.29 7.28 7.28 7.26 6.96 6.95 6.95 6.93 6.91 6.89 6.88 6.44
5.60 5.43
3.82
2.18
0.85 2.05 2.05 2.02 3.14 1.55 0.90 0.98 0.99 2.99 0.92
1.00 0.91 0.07
3.01
2.93
f1 (ppm)

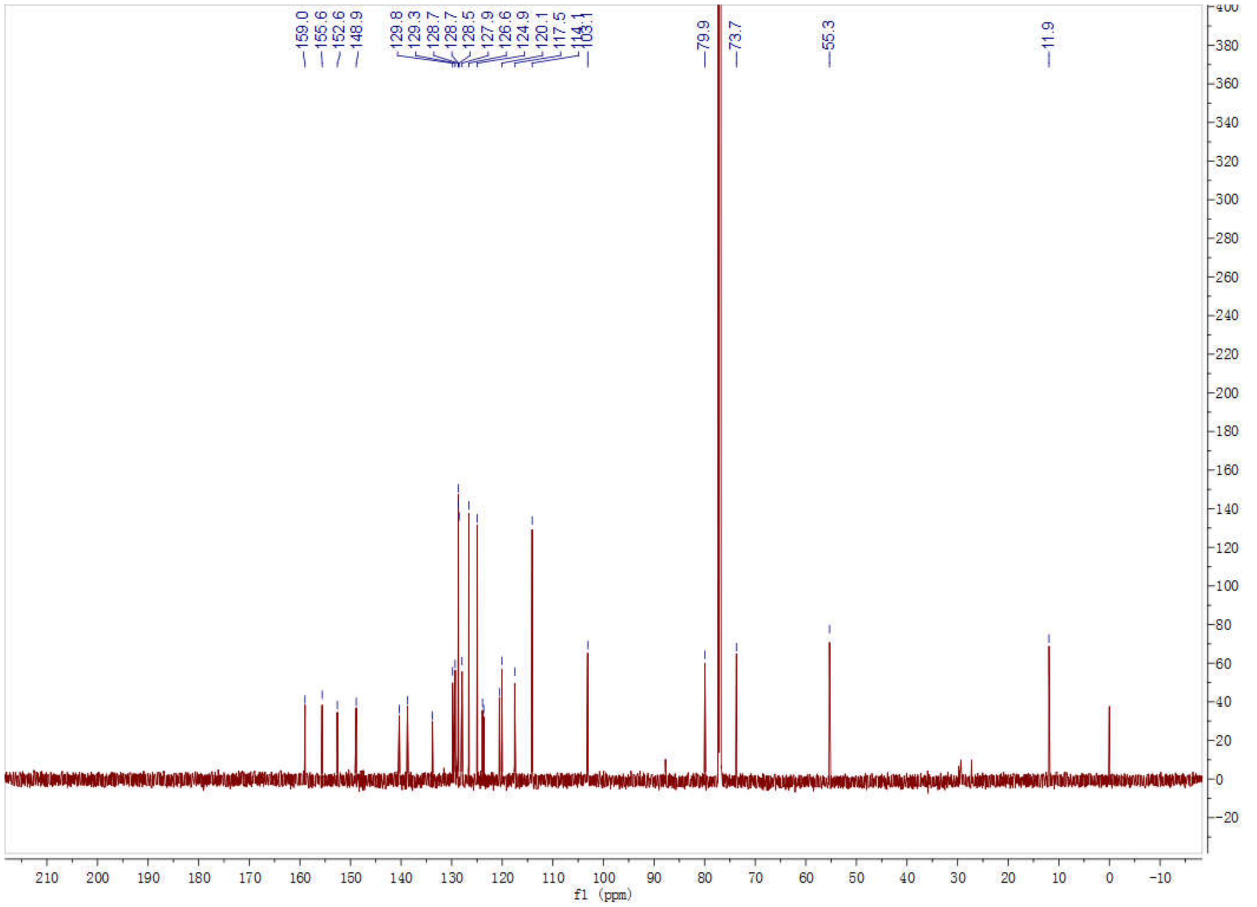

159.0 155.6 152.6 148.9
129.8 129.3 128.7 128.5 127.9 126.6 124.9 120.1 117.5 114.1 103.1
79.9 73.7
55.3
11.9
f1 (ppm)

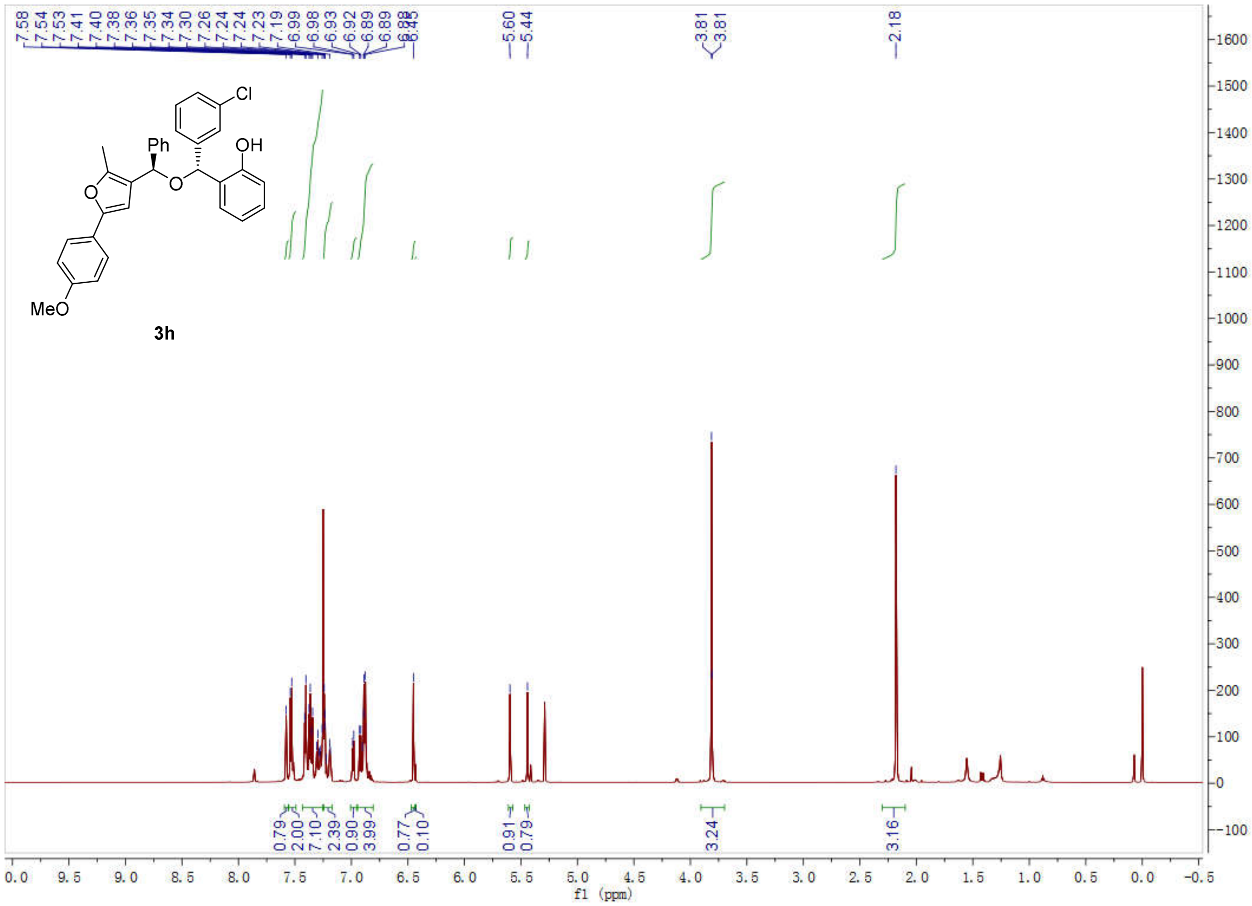
7.58 7.54 7.53 7.41 7.40 7.38 7.36 7.35 7.34 7.30 7.26 7.24 7.24 7.23 7.19 6.99 6.98 6.93 6.92 6.89 6.89 6.89
5.60 5.44
3.81 3.81
2.18
3h
0.79 2.00 7.10 2.39 0.90 3.99 0.77 0.10 0.91 0.79 3.24 3.16
f1 (ppm)

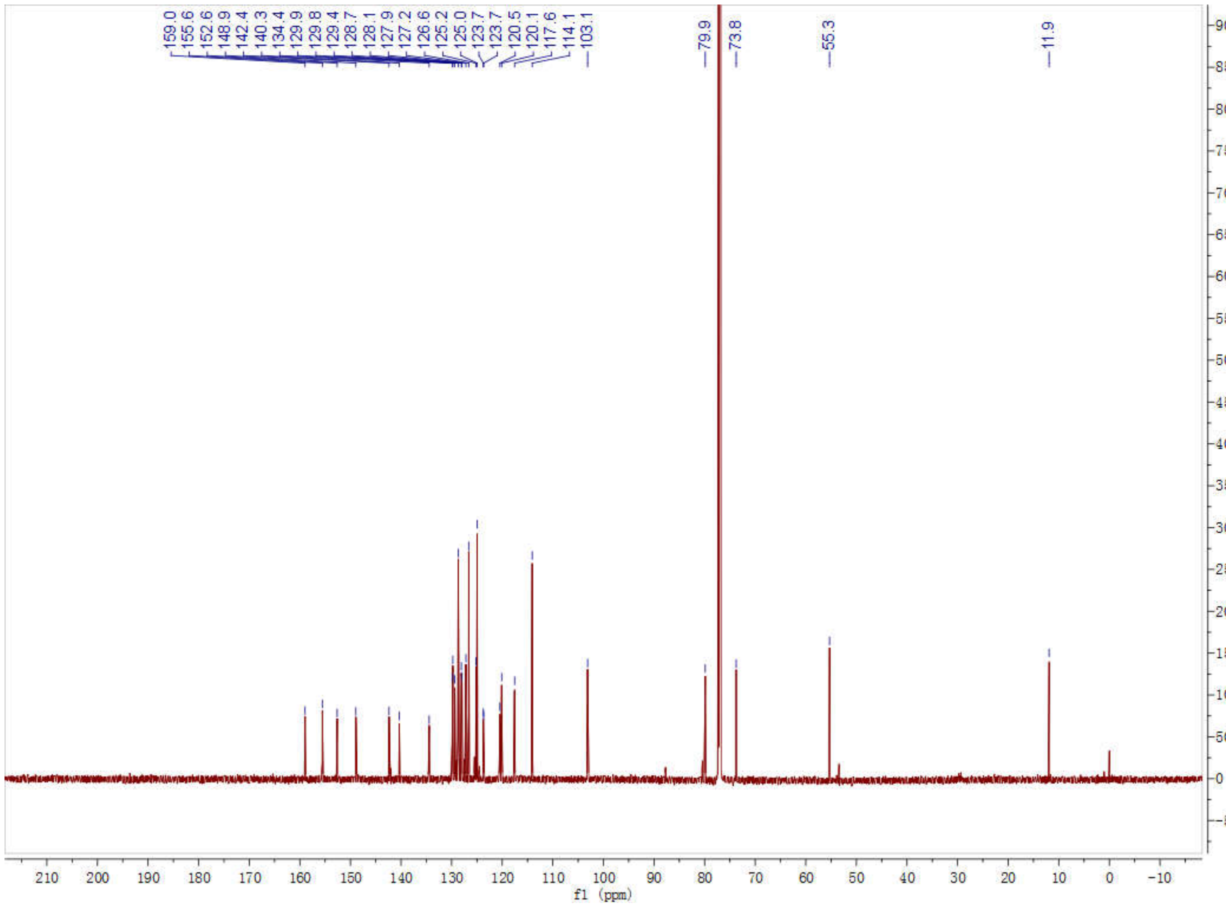
159.0 155.6 152.6 148.9 142.4 140.3 134.4 129.9 129.8 129.4 128.7 128.1 127.9 127.2 126.6 125.2 125.0 123.7 123.7 120.5 120.1 117.6 114.1 103.1
79.9 73.8
55.3
11.9
f1 (ppm)

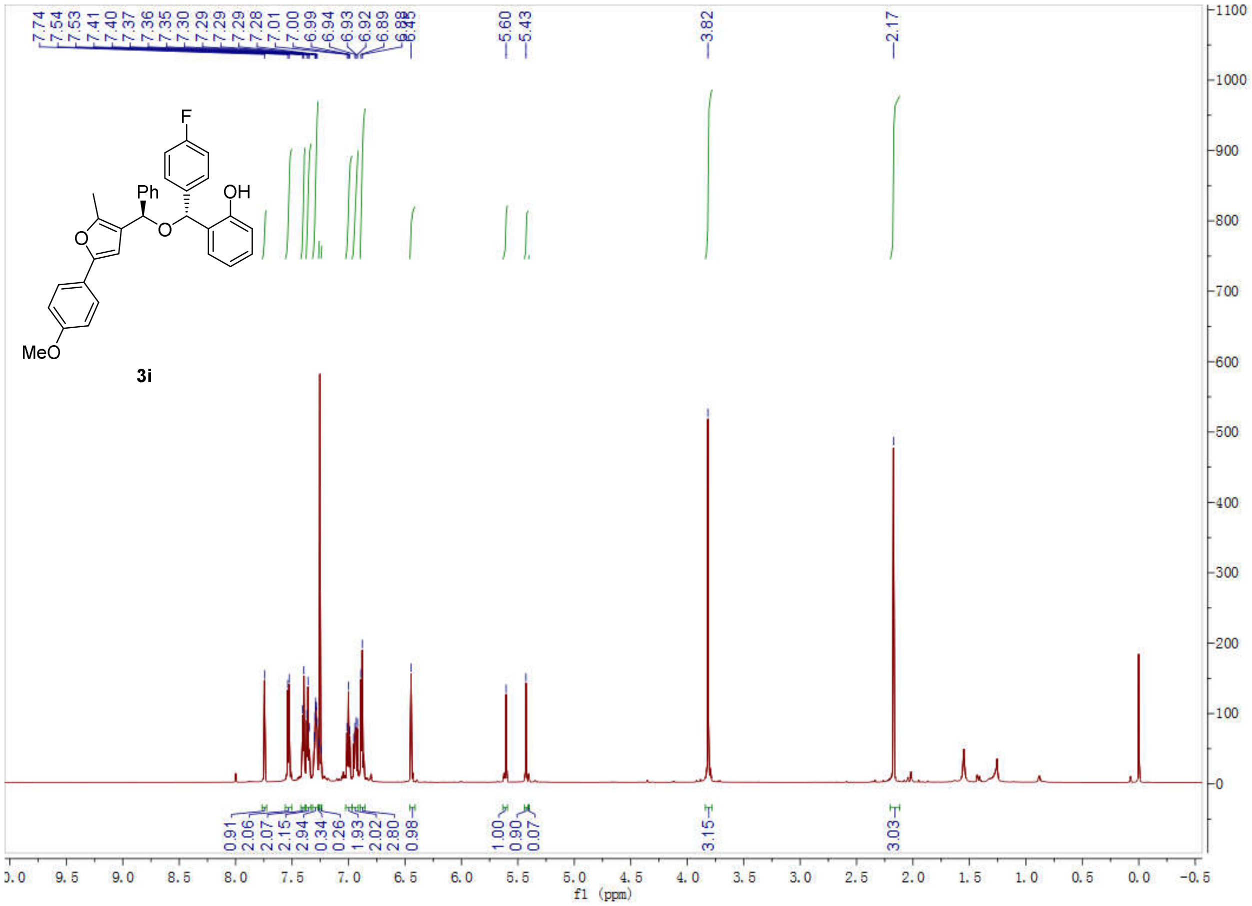
7.74
7.54
7.53
7.41
7.40
7.37
7.36
7.35
7.30
7.29
7.29
7.29
7.28
7.01
7.00
6.99
6.94
6.93
6.92
6.89
5.60
5.43
3.82
2.17
F
Ph
OH
O
MeO
3i
0.91
2.06
2.07
2.15
2.94
0.34
0.26
1.93
2.02
2.80
0.98
1.00
0.90
0.07
3.15
3.03
f1 (ppm)

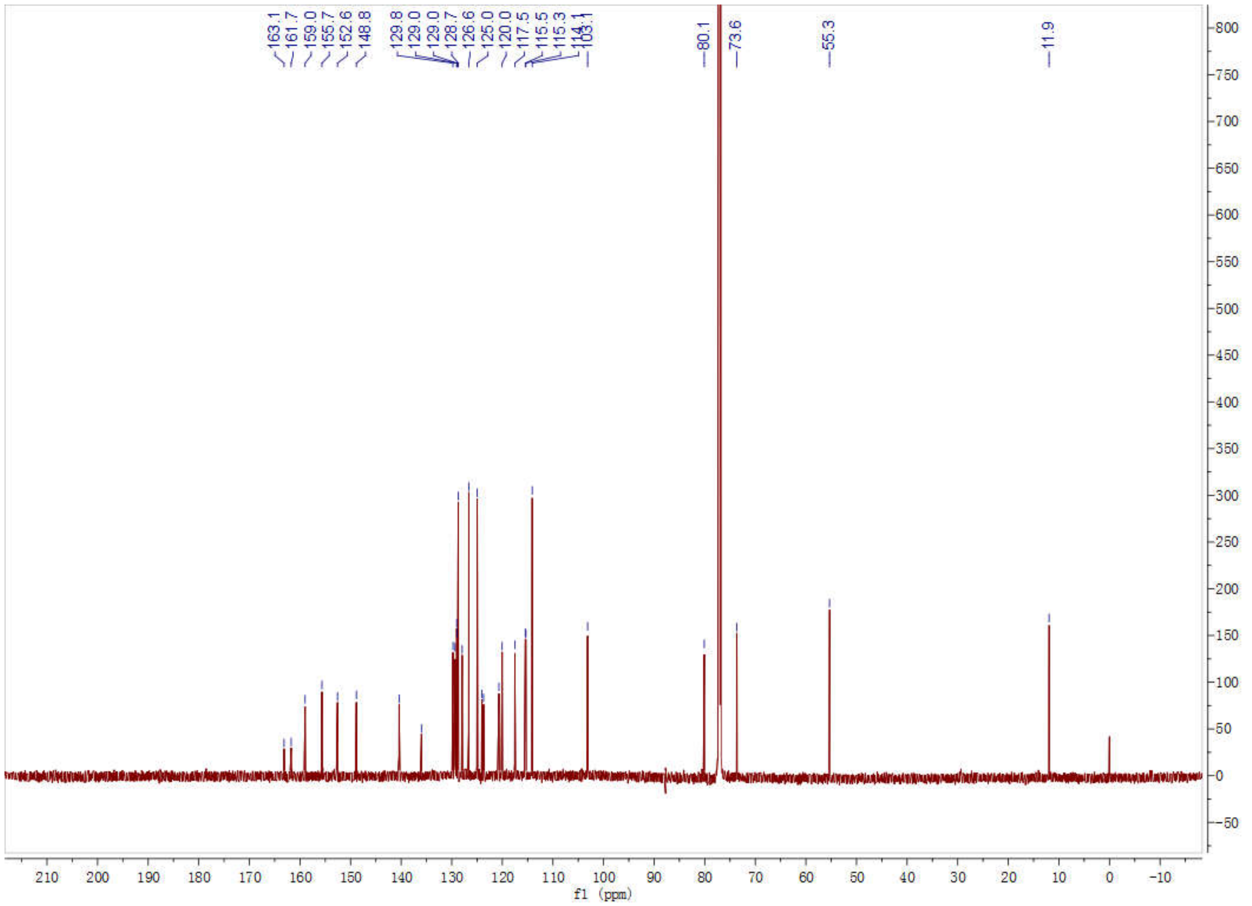
163.1
161.7
159.0
155.7
152.6
148.8
129.8
129.0
129.0
128.7
126.6
125.0
120.0
117.5
115.5
115.3
114.1
103.1
80.1
73.6
55.3
11.9
f1 (ppm)

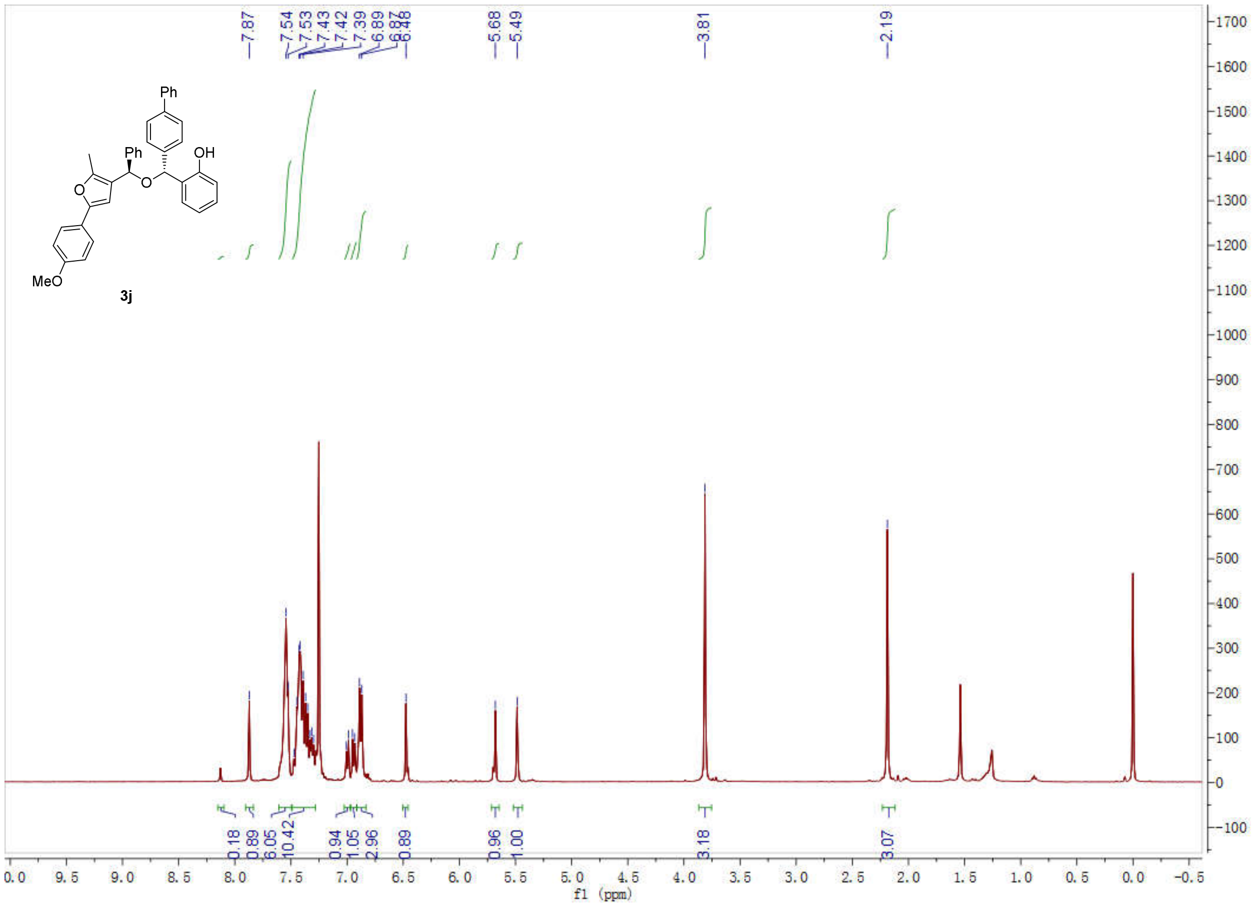
7.87
7.54
7.53
7.43
7.42
7.39
6.89
6.87
6.48
5.68
5.49
3.81
2.19
Ph
OH
Ph
O
O
MeO
3j
0.18
0.89
6.05
10.42
0.94
1.05
2.96
0.89
0.96
1.00
3.18
3.07
f1 (ppm)

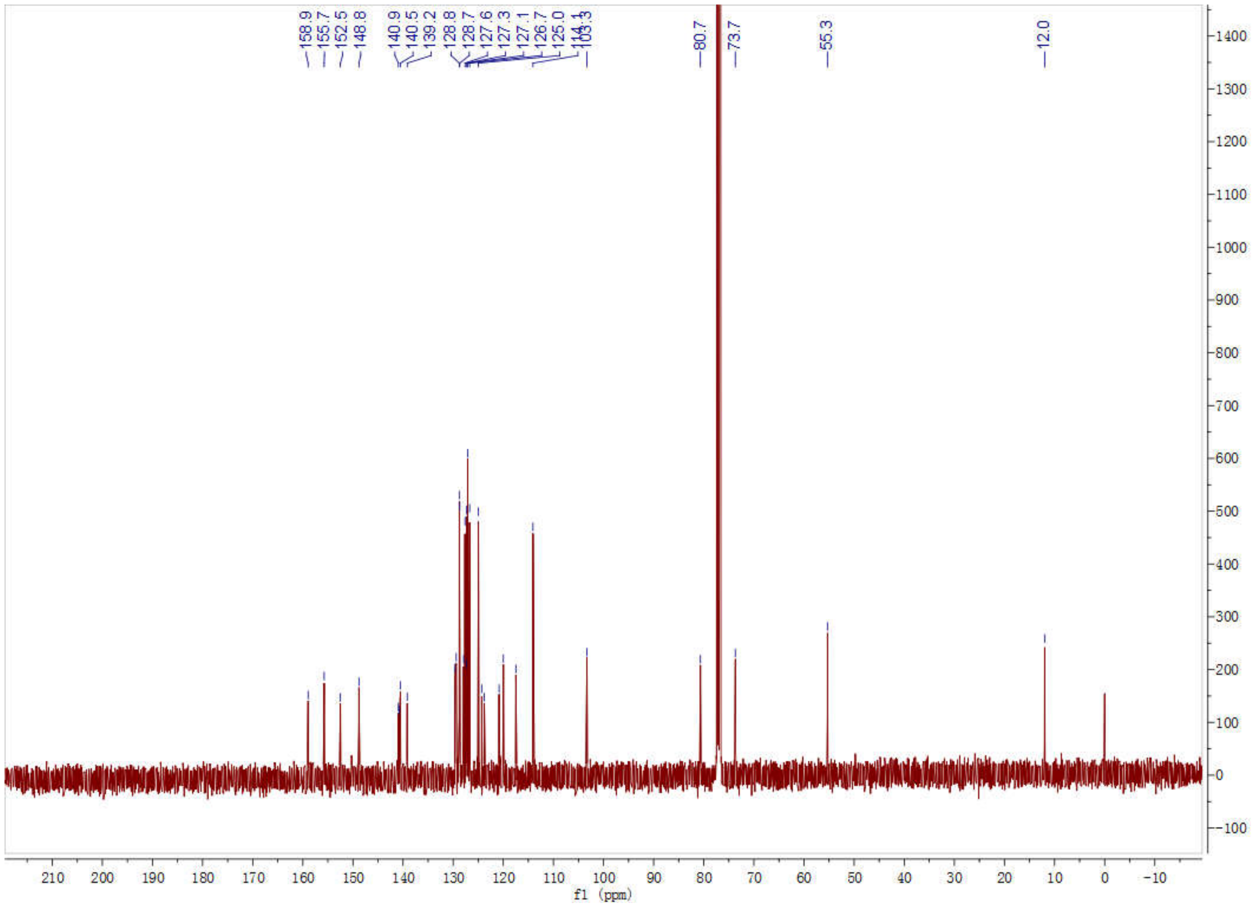
158.9
155.7
152.5
148.8
140.9
140.5
139.2
128.8
128.7
127.6
127.3
127.1
126.7
125.0
114.1
103.3
80.7
73.7
55.3
12.0
f1 (ppm)

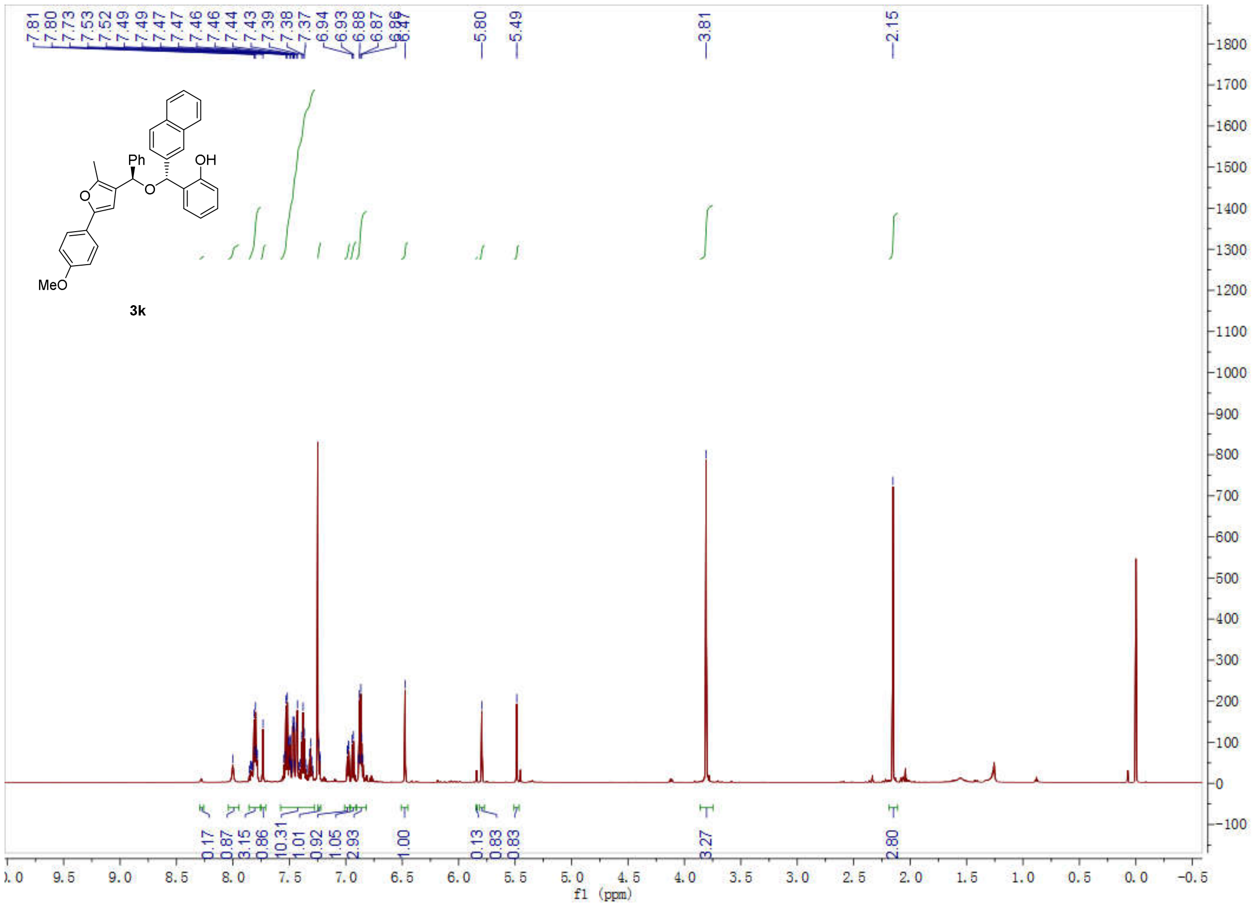
7.81 7.80 7.73 7.53 7.52 7.49 7.49 7.47 7.47 7.46 7.46 7.44 7.43 7.39 7.38 7.37 6.94 6.93 6.88 6.87 6.86 6.47
5.80
5.49
3.81
2.15
Ph
OH
O
MeO
3k
0.17 0.87 3.15 0.86 10.31 1.01 0.92 1.05 2.93 1.00 0.13 0.83 0.83 3.27 2.80
f1 (ppm)

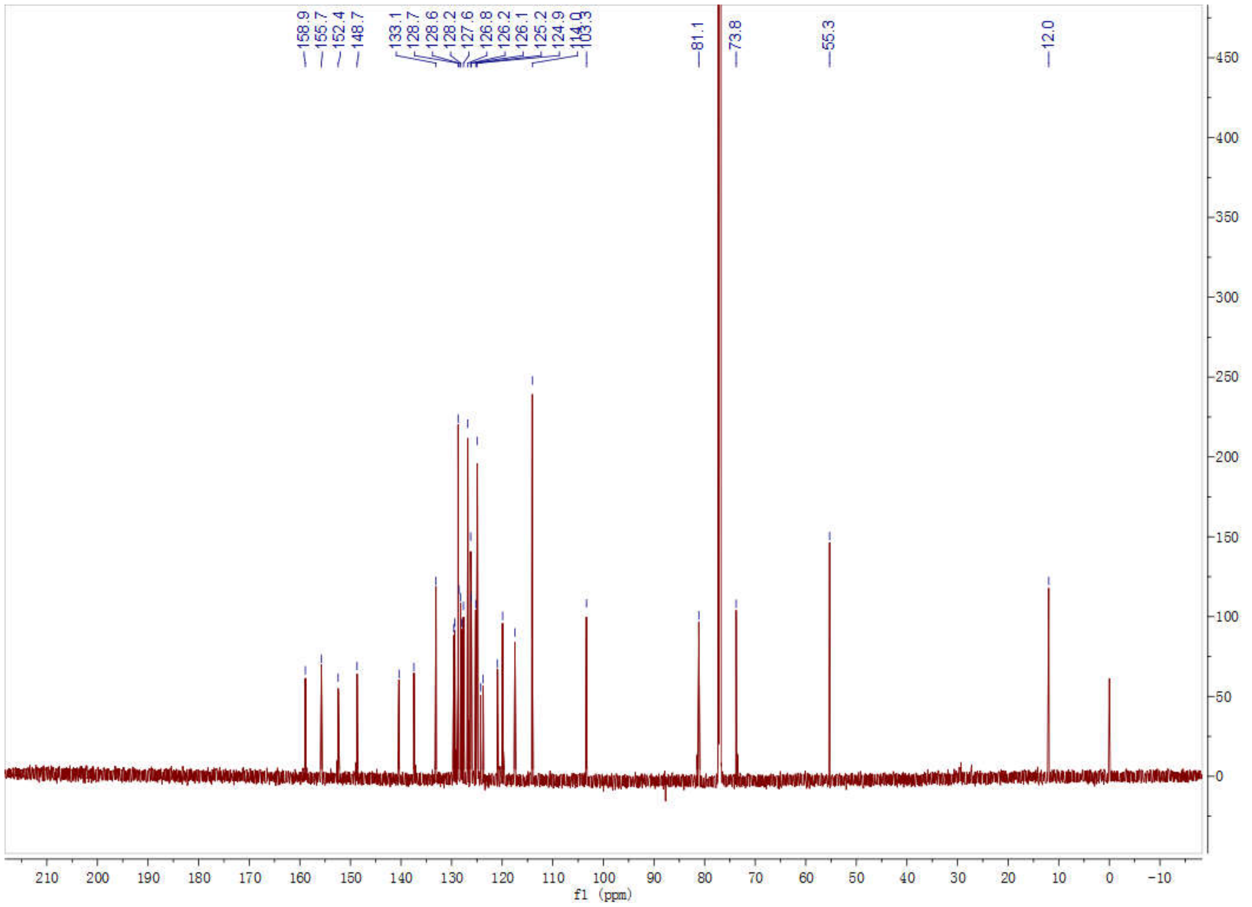
158.9 155.7 152.4 148.7
133.1 128.7 128.6 128.2 127.6 126.8 126.2 126.1 125.2 124.9 114.0 103.3
81.1
73.8
55.3
12.0
f1 (ppm)

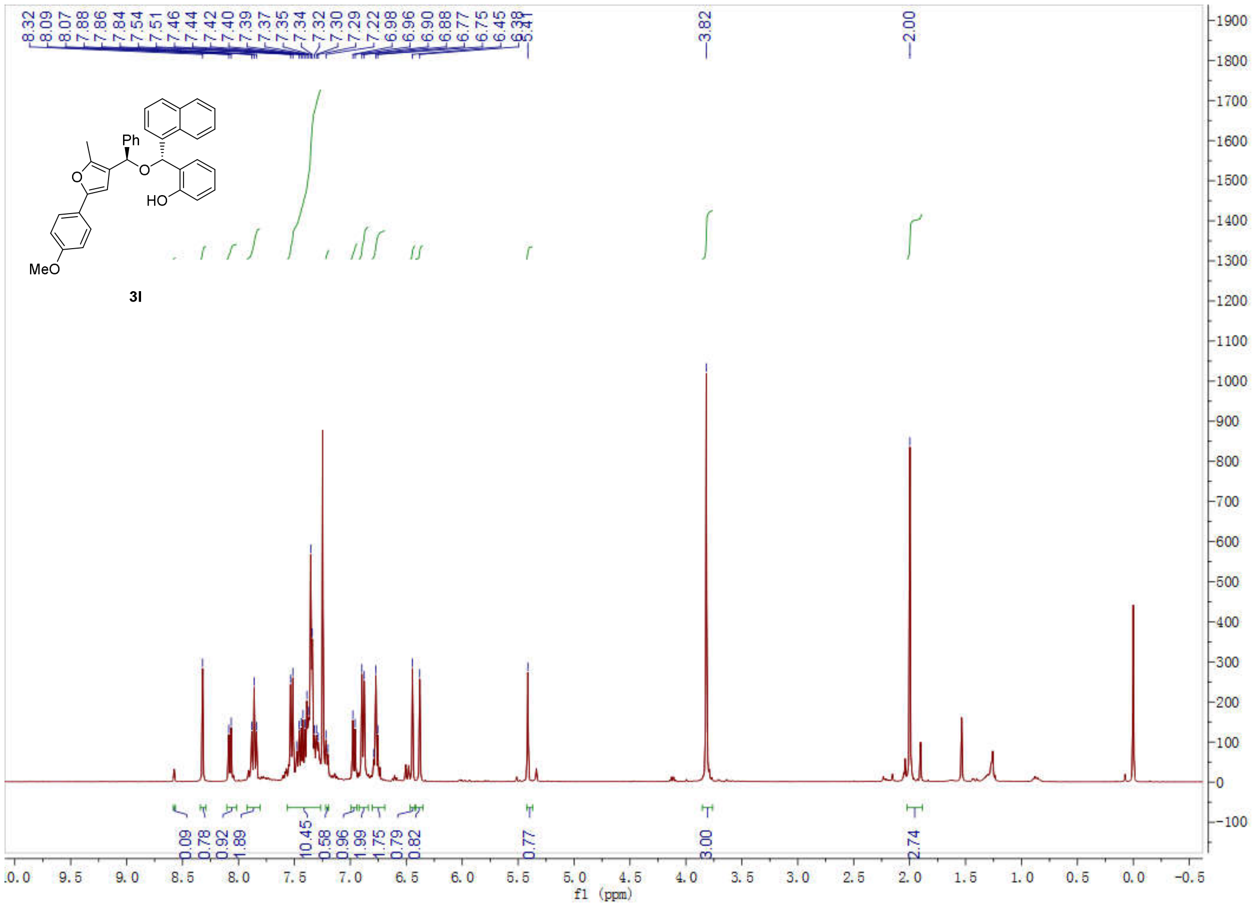

3l

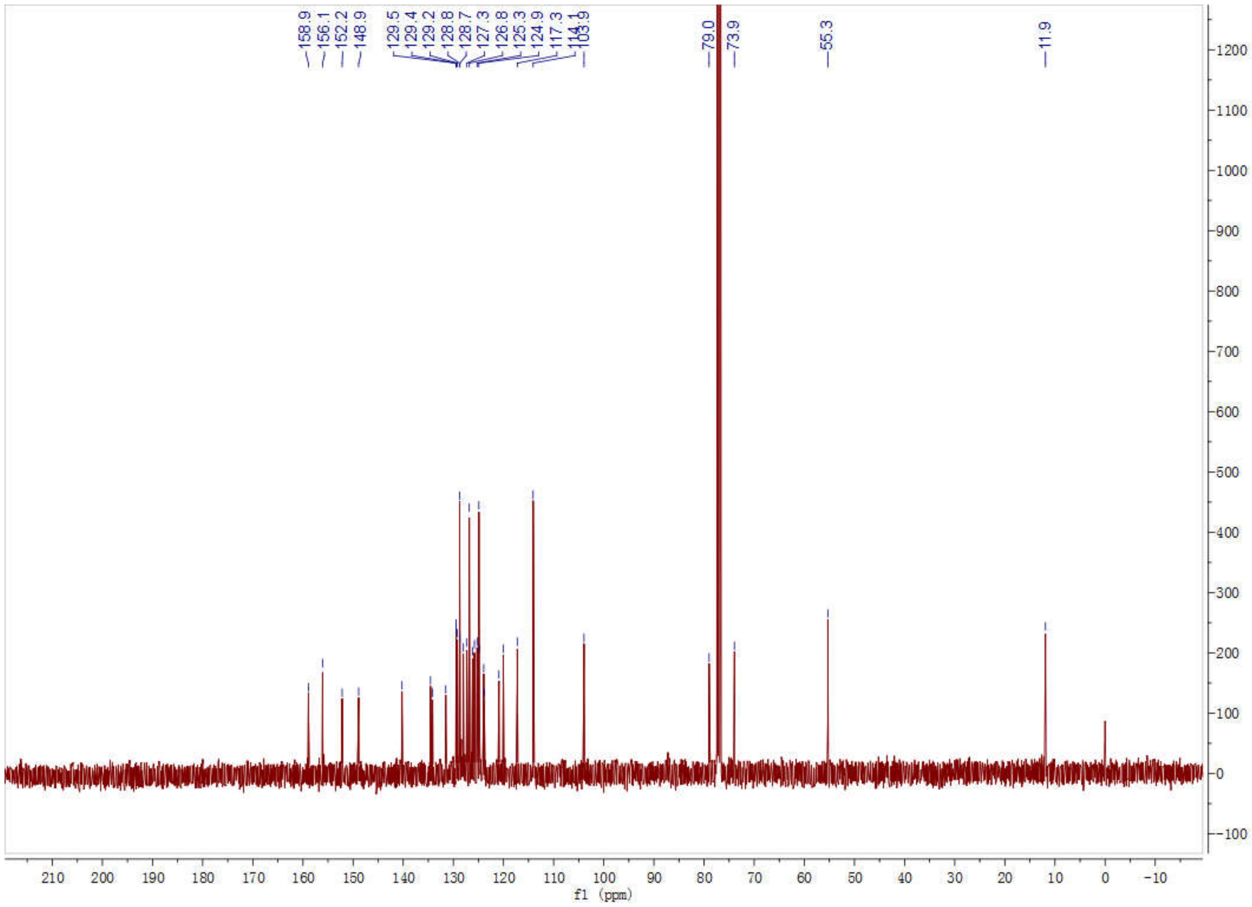

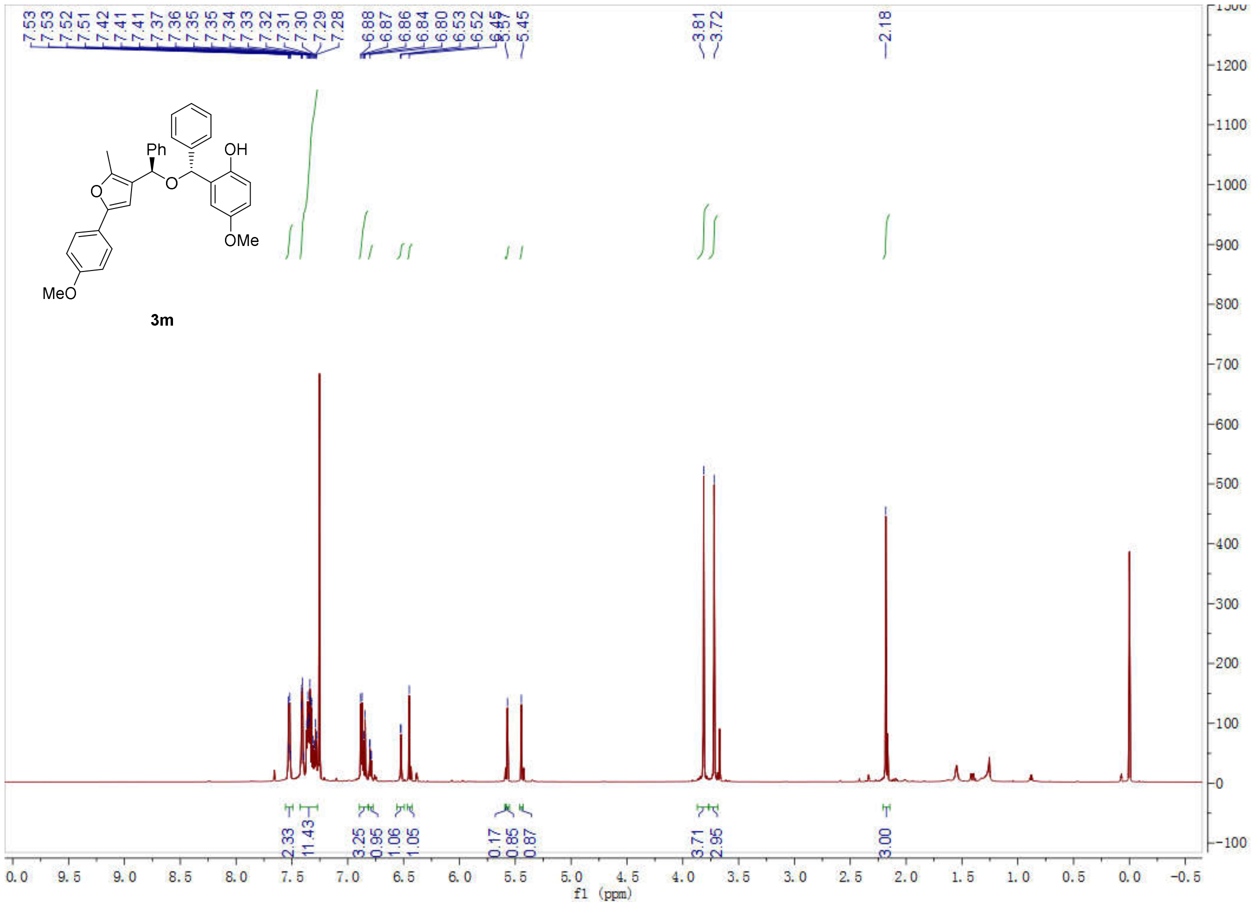

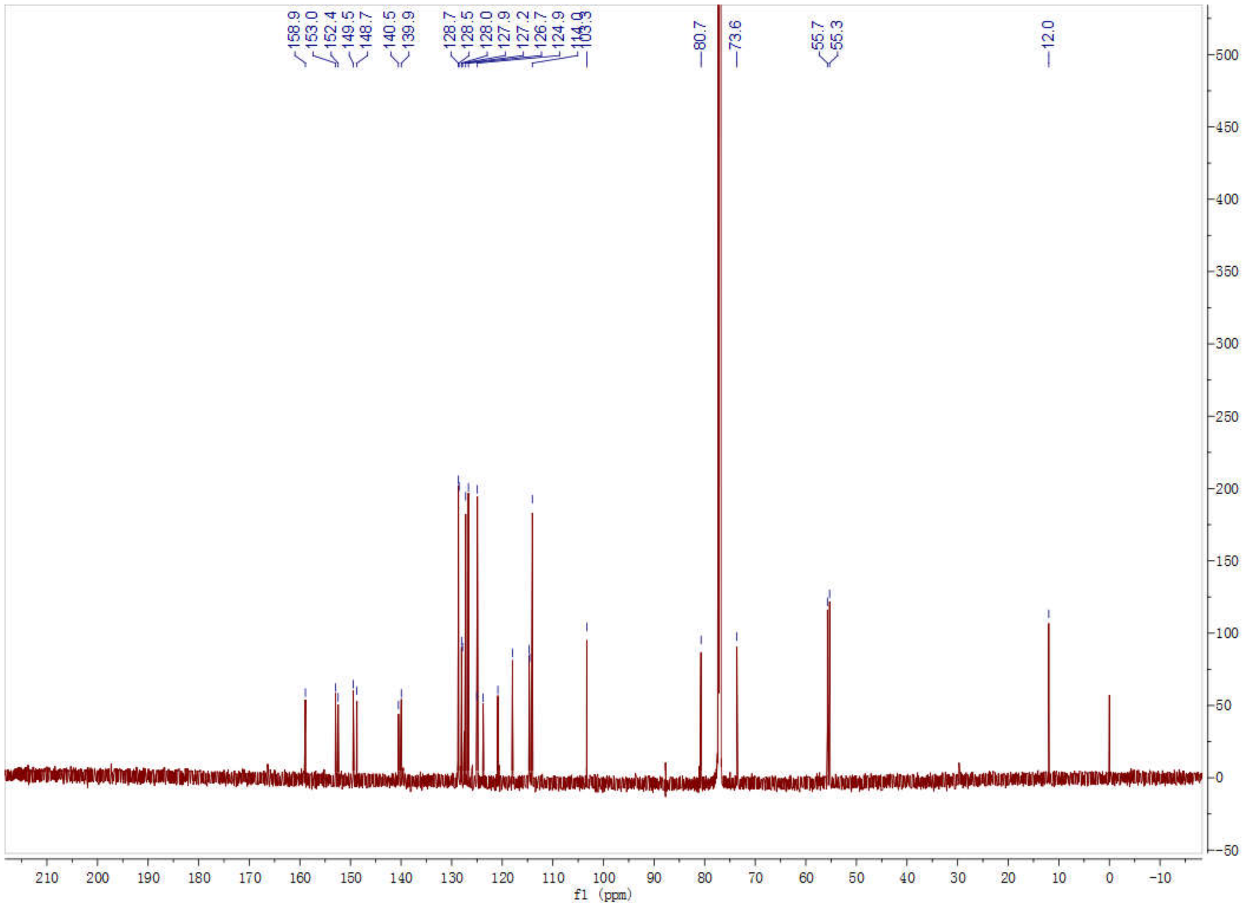

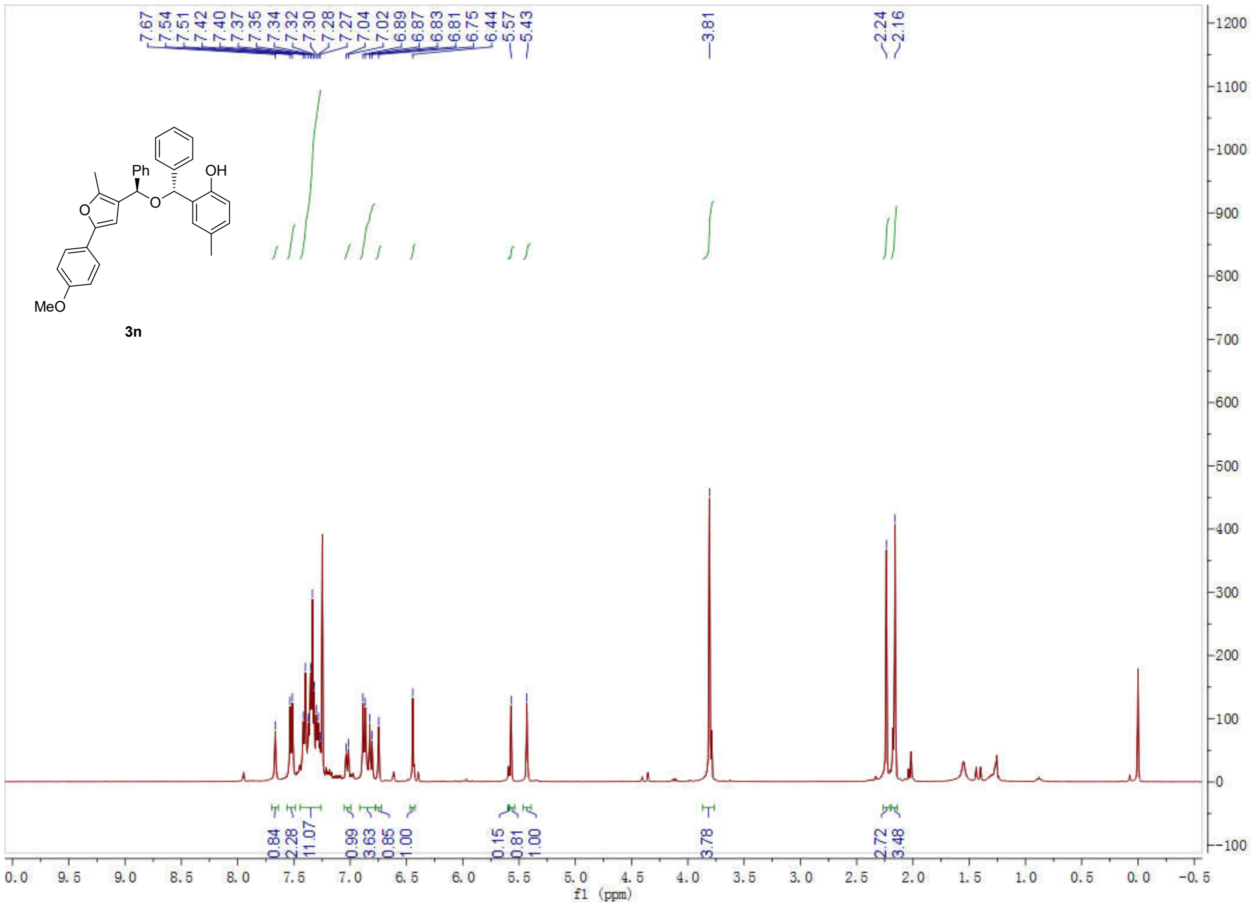
7.67 7.54 7.51 7.42 7.40 7.37 7.35 7.34 7.32 7.30 7.28 7.27 7.04 7.02 6.89 6.87 6.83 6.81 6.75 6.44 5.57 5.43
3.81
2.24 2.16
Ph
OH
O
MeO
3n
0.84 2.28 11.07 0.99 3.63 0.85 1.00 0.15 0.81 1.00 3.78 2.72 3.48
f1 (ppm)

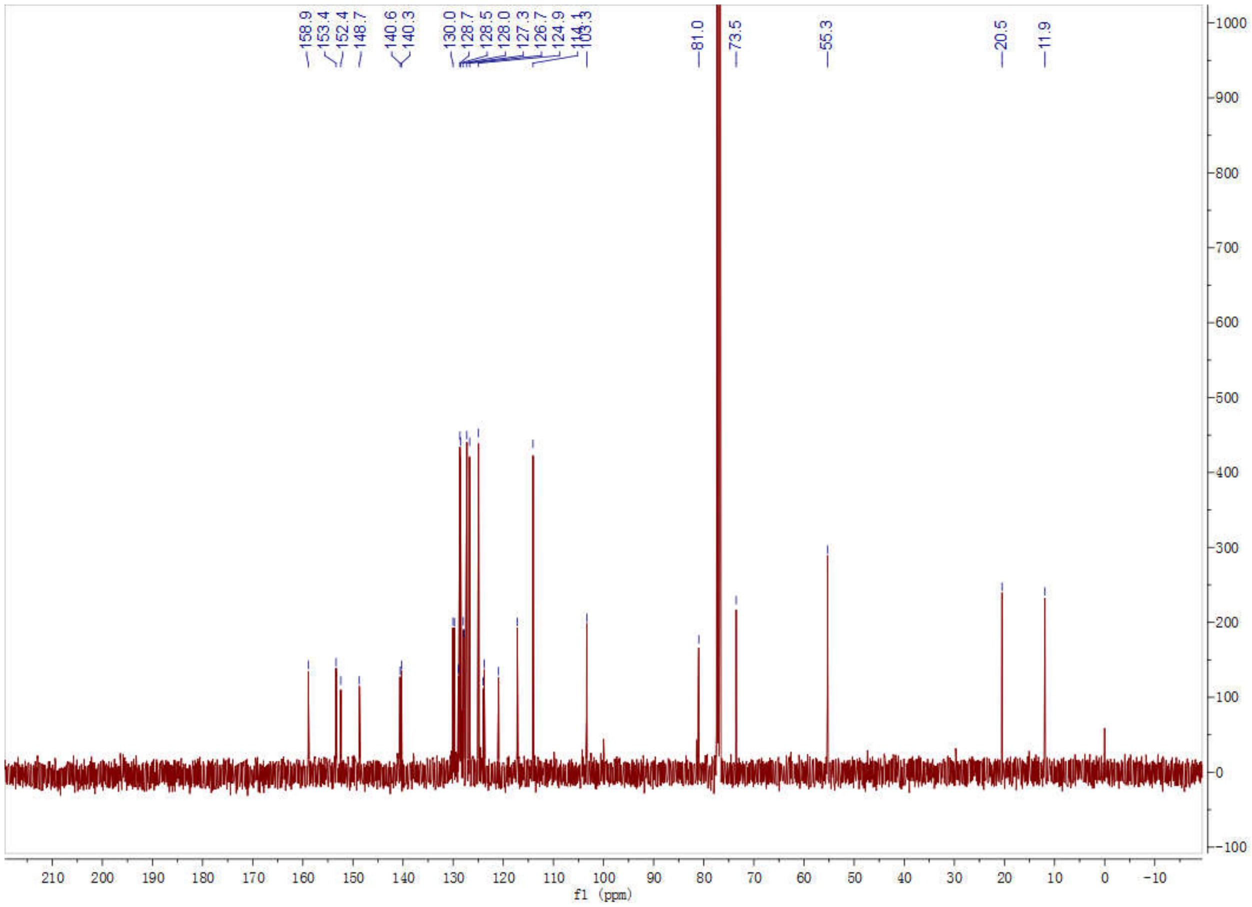
158.9 153.4 152.4 148.7 140.6 140.3 130.0 128.7 128.5 128.0 127.3 126.7 124.9 114.1 103.3 81.0 73.5 55.3 20.5 11.9
f1 (ppm)

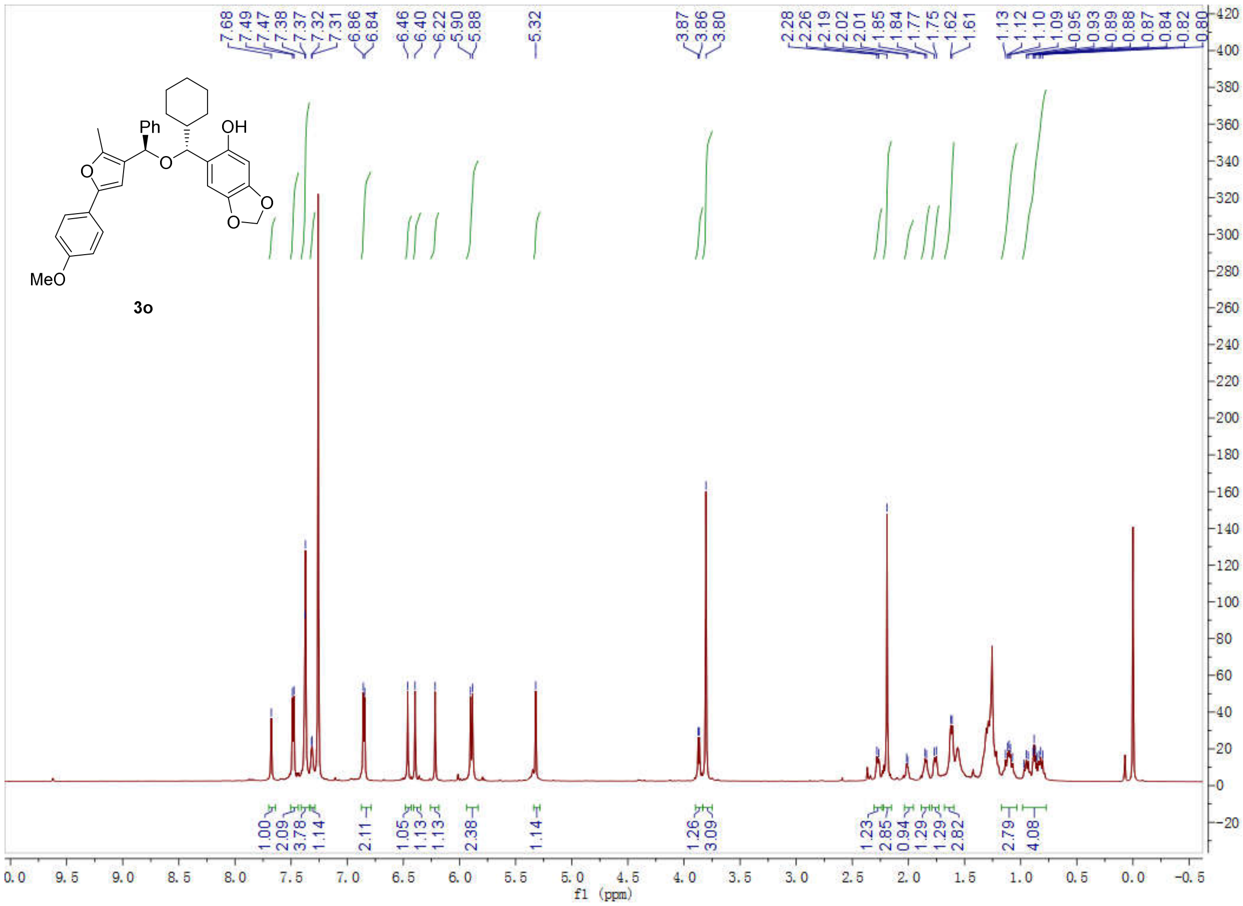

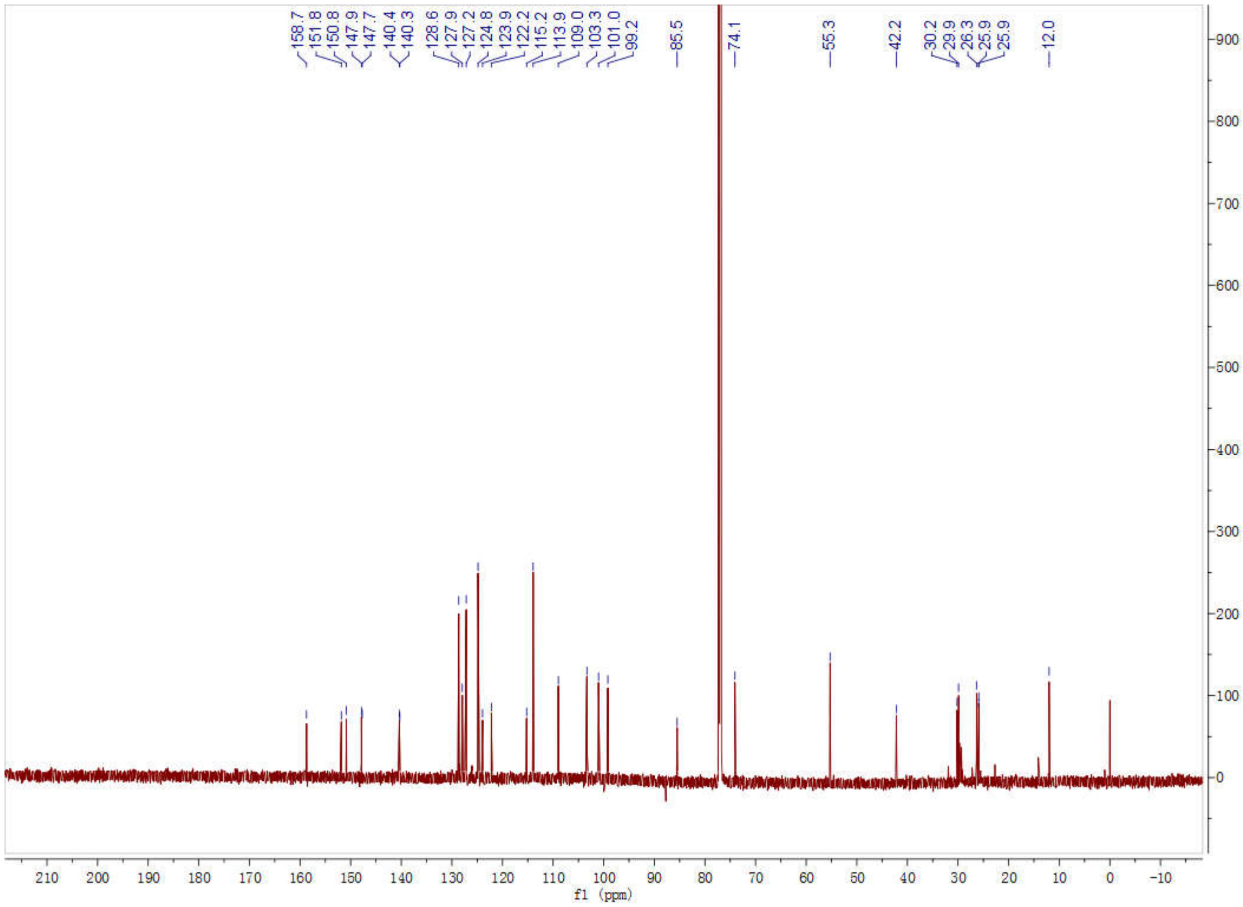

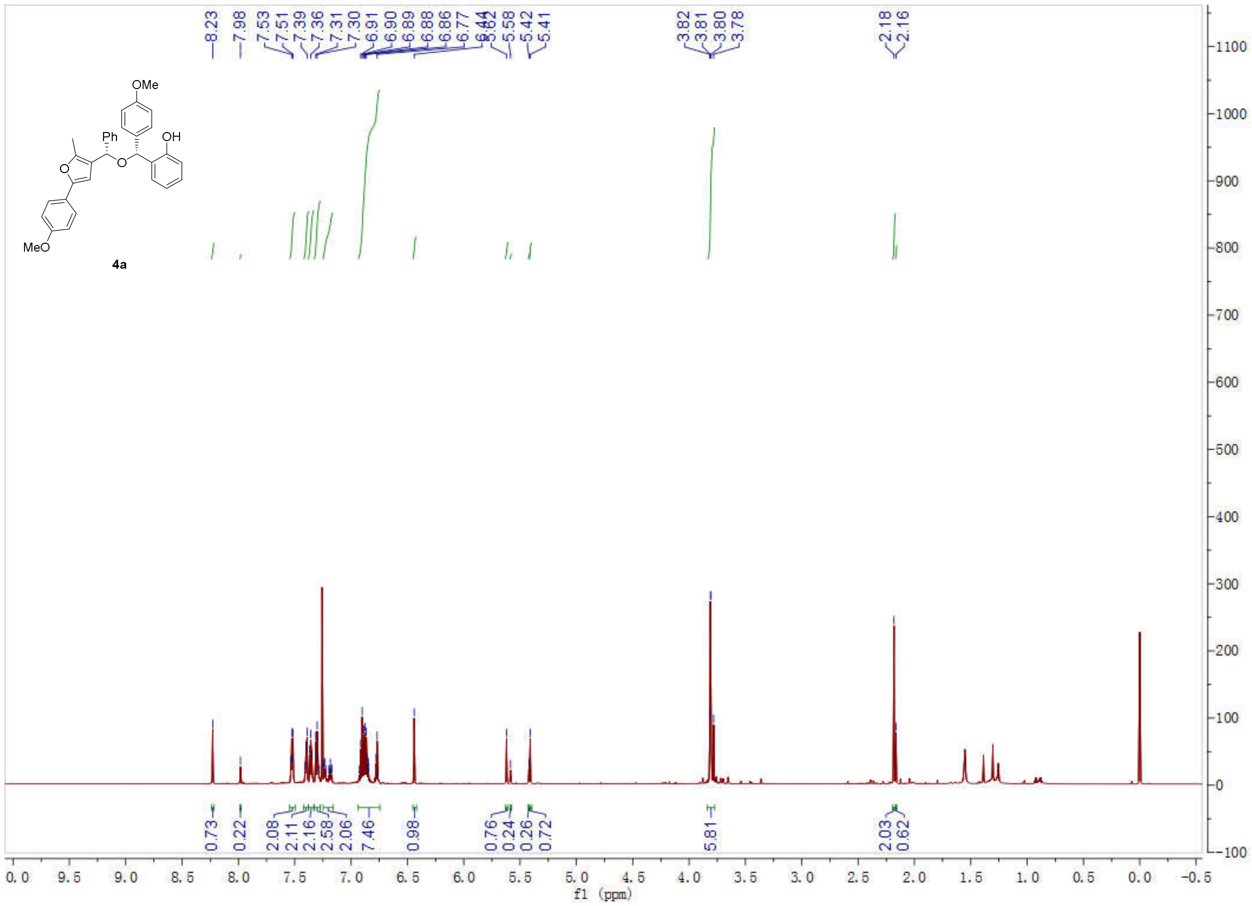
8.23
7.98
7.53
7.51
7.39
7.36
7.31
7.30
6.91
6.90
6.89
6.88
6.86
6.77
6.44
5.62
5.58
5.42
5.41
3.82
3.81
3.80
3.78
2.18
2.16
OMe
Ph
OH
O
O
MeO
4a
0.73
0.22
2.08
2.11
2.16
2.58
2.06
7.46
0.98
0.76
0.24
0.26
0.72
5.81
2.03
0.62
f1 (ppm)

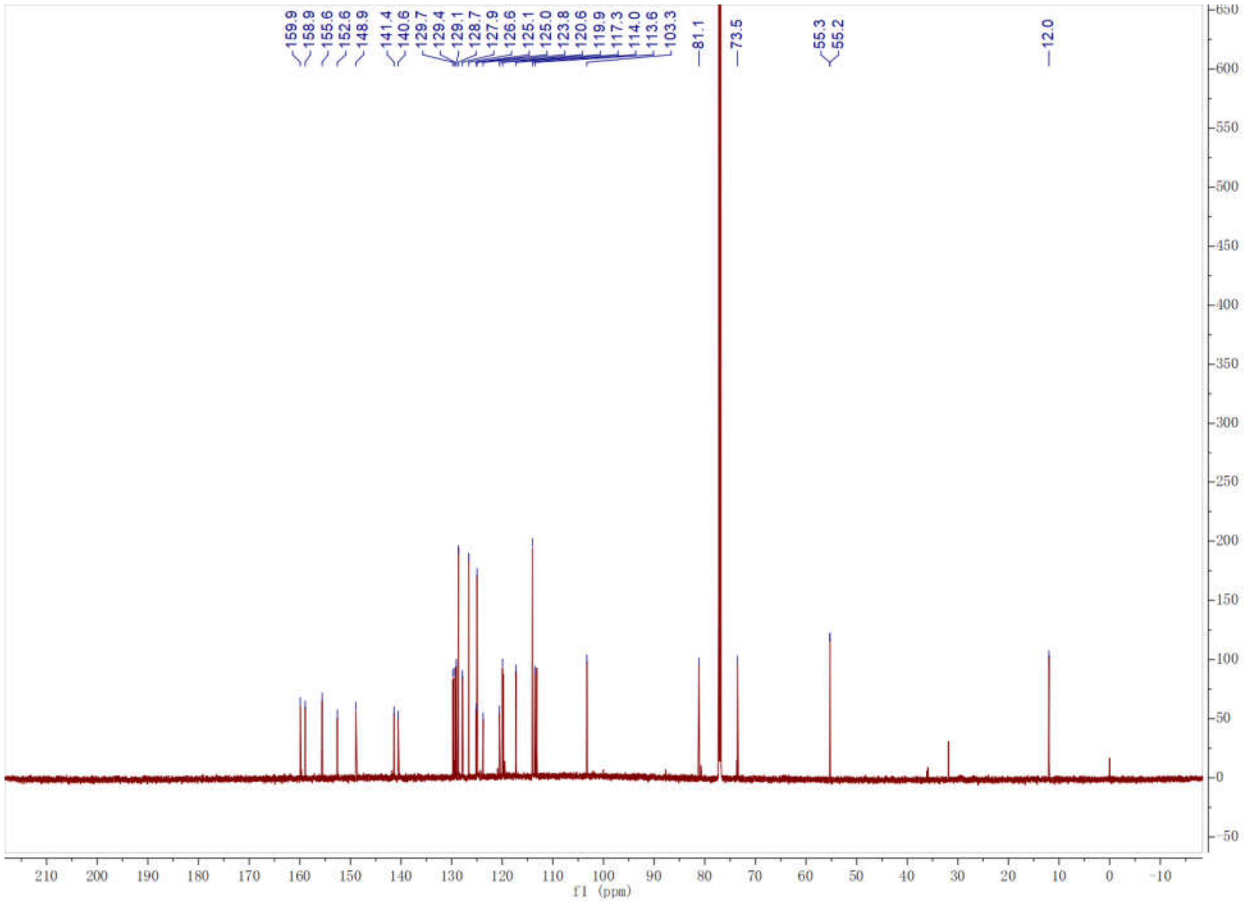
159.9
158.9
155.6
152.6
148.9
141.4
140.6
129.7
129.4
129.1
128.7
127.9
126.6
125.1
125.0
123.8
120.6
119.9
117.3
114.0
113.6
103.3
81.1
73.5
55.3
55.2
12.0
f1 (ppm)

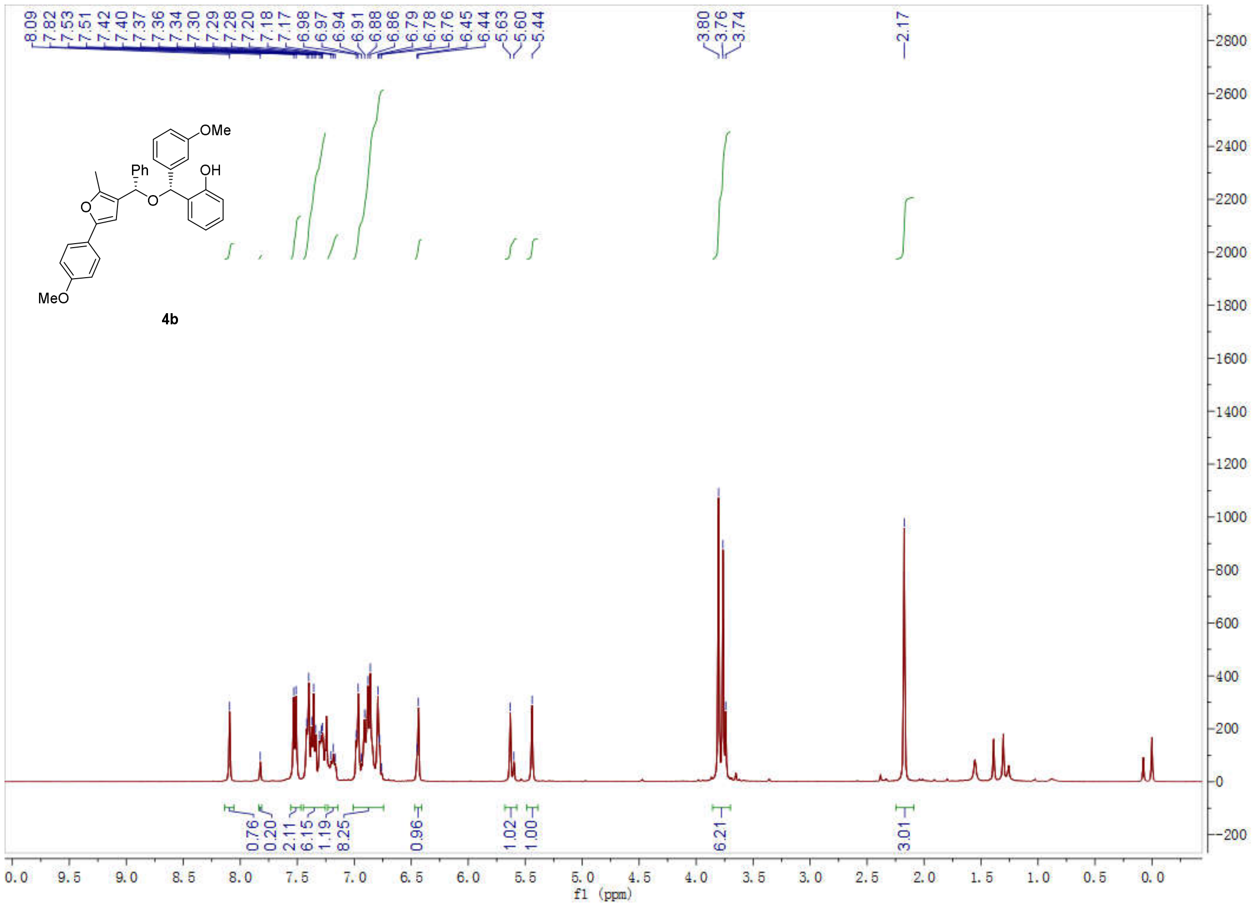

8.09 7.82 7.53 7.51 7.42 7.40 7.37 7.36 7.34 7.30 7.29 7.28 7.20 7.18 7.17 6.98 6.97 6.94 6.91 6.88 6.86 6.79 6.78 6.76 6.45 6.44 5.63 5.60 5.44
3.80 3.76 3.74
2.17
OMe
Ph
OH
O
MeO
4b
0.76 0.20 2.11 6.15 1.19 8.25 0.96 1.02 1.00 6.21 3.01
f1 (ppm)

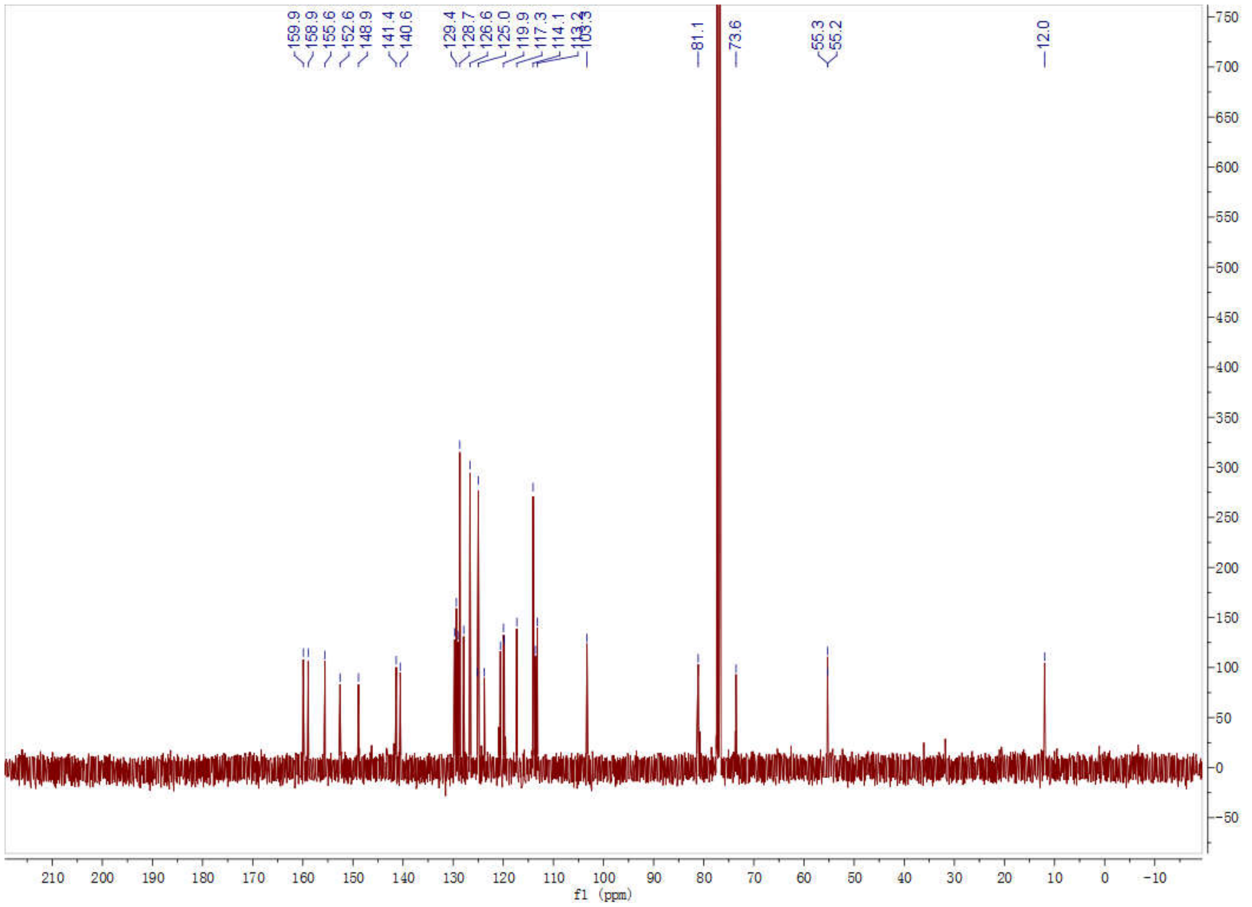

159.9 158.9 155.6 152.6 148.9 141.4 140.6 129.4 128.7 126.6 125.0 119.9 117.3 114.1 113.2 103.3
81.1
73.6
55.3 55.2
12.0
f1 (ppm)

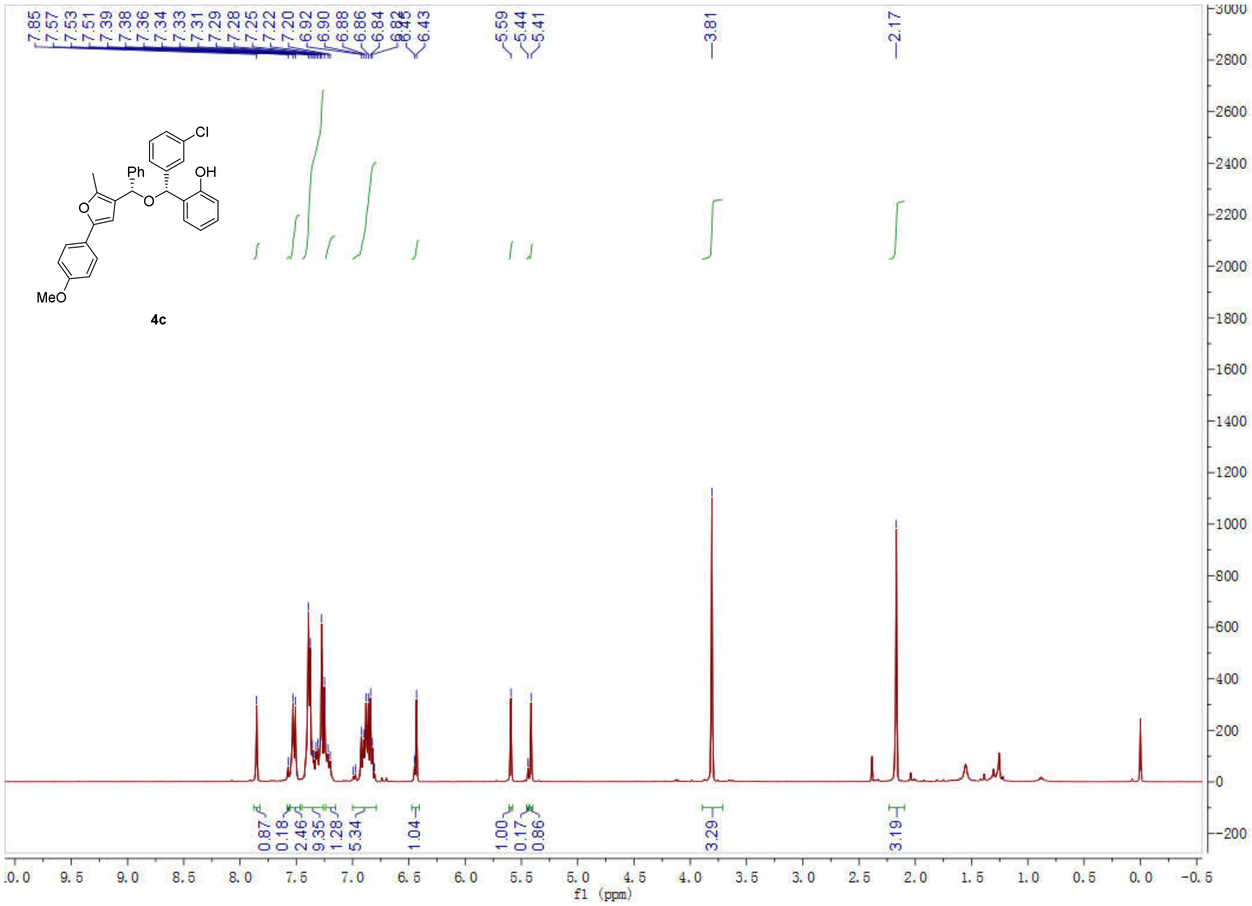
Cl
Ph
OH
O
O
MeO
4c
f1 (ppm)

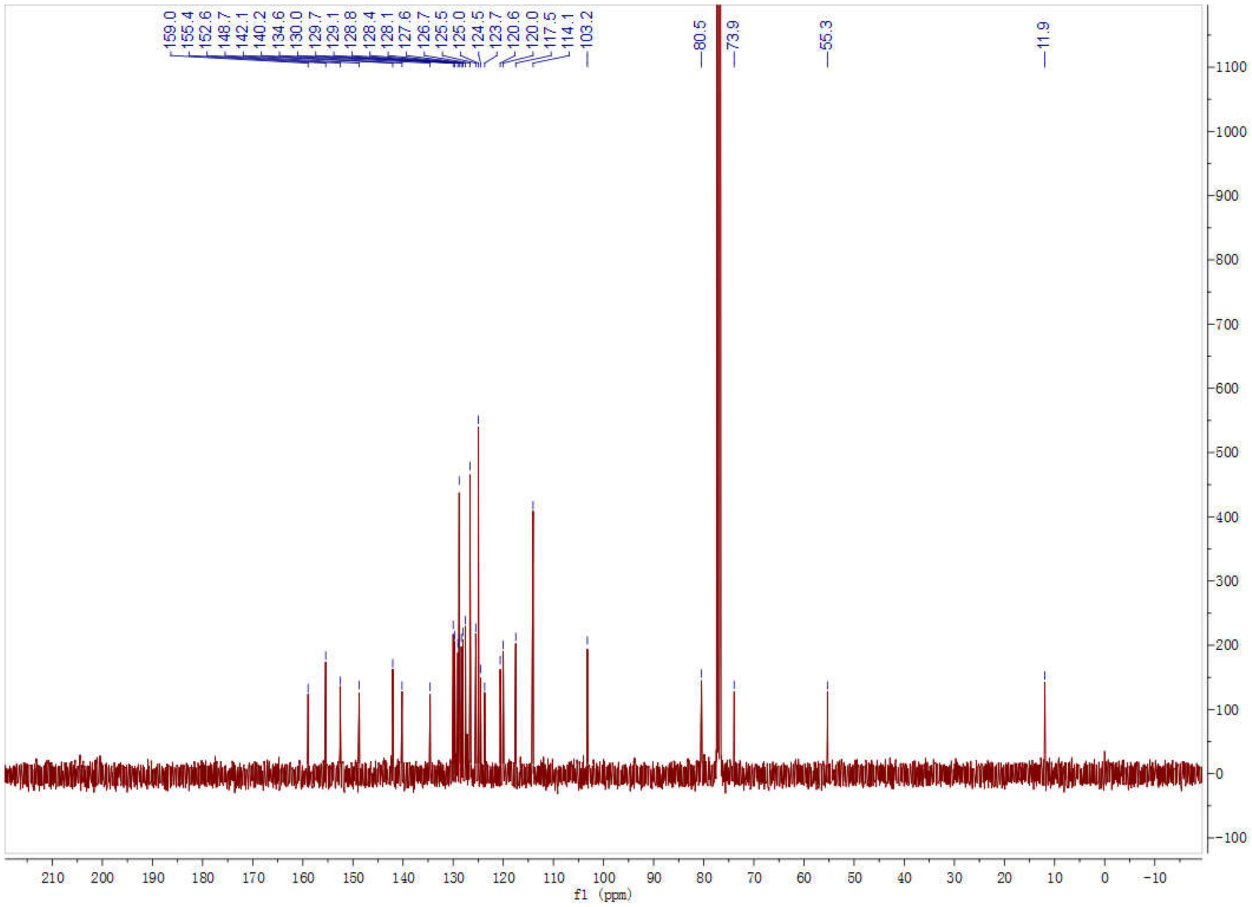
f1 (ppm)

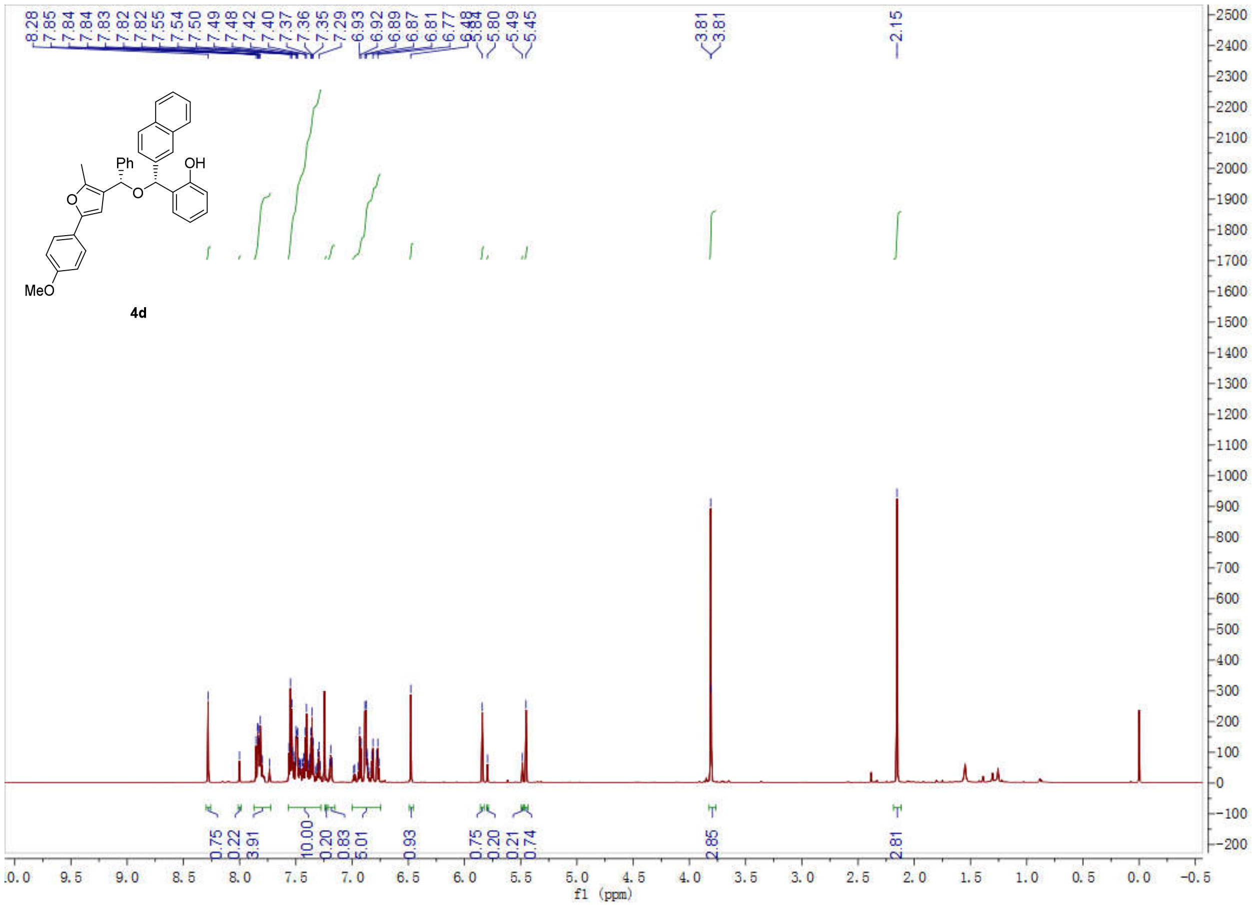
8.28
7.85
7.84
7.84
7.83
7.82
7.82
7.55
7.54
7.50
7.49
7.48
7.42
7.40
7.37
7.36
7.35
7.29
6.93
6.92
6.89
6.87
6.81
6.77
6.48
5.84
5.80
5.49
5.45
3.81
3.81
2.15
Ph
OH
MeO
4d
0.75
0.22
3.91
10.00
0.20
0.83
5.01
0.93
0.75
0.20
0.21
0.74
2.85
2.81
f1 (ppm)

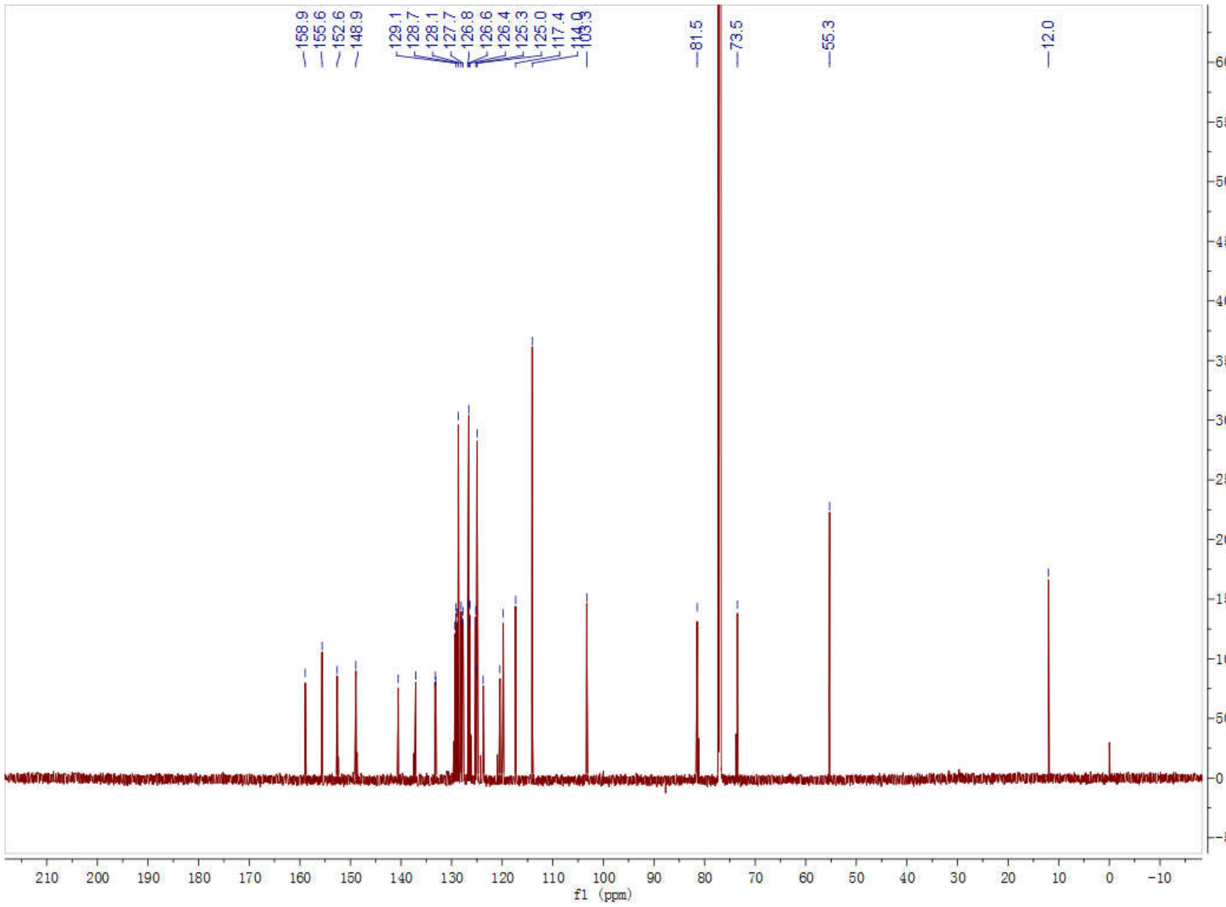
158.9
155.6
152.6
148.9
129.1
128.7
128.1
127.7
126.8
126.6
126.4
125.3
125.0
117.4
114.0
103.3
81.5
73.5
55.3
12.0
f1 (ppm)

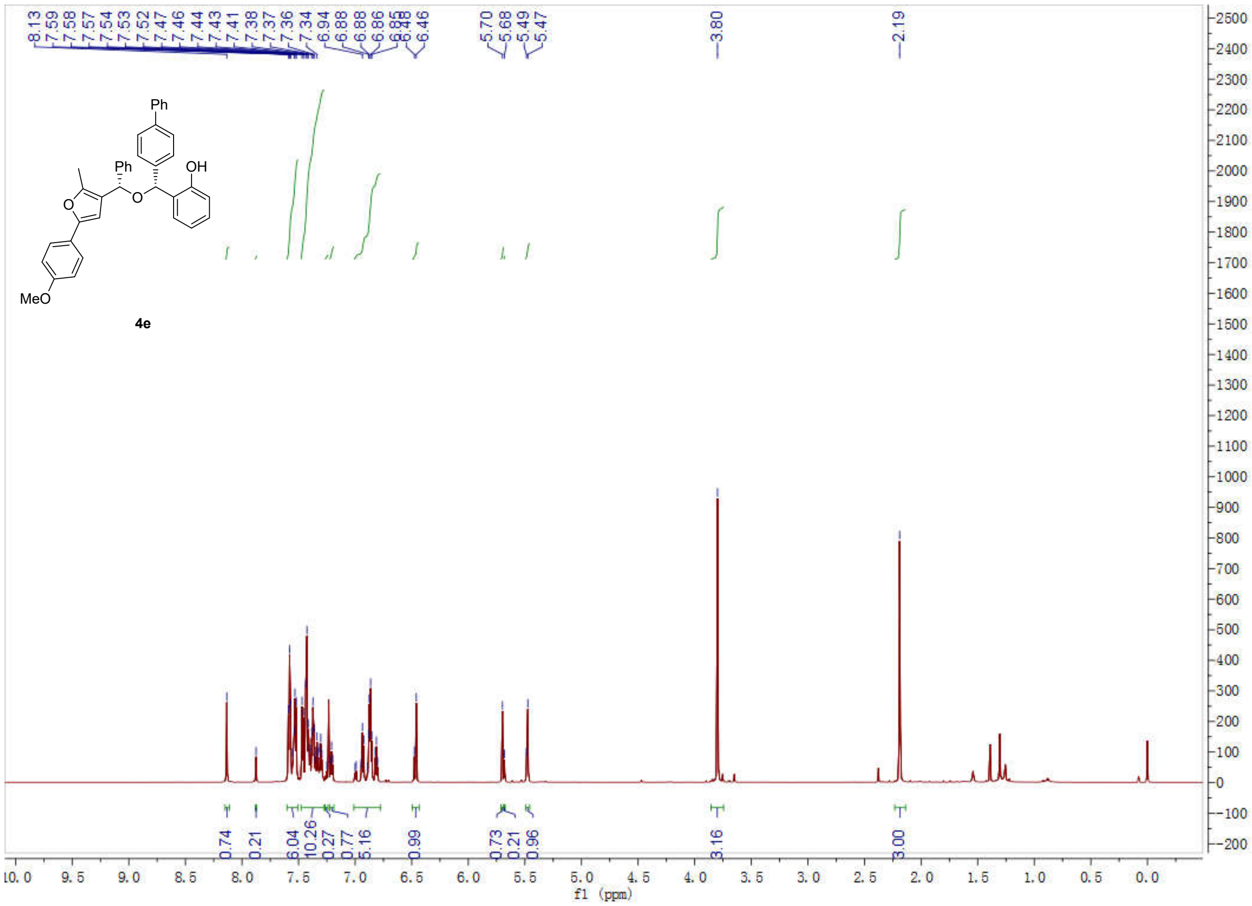

4e

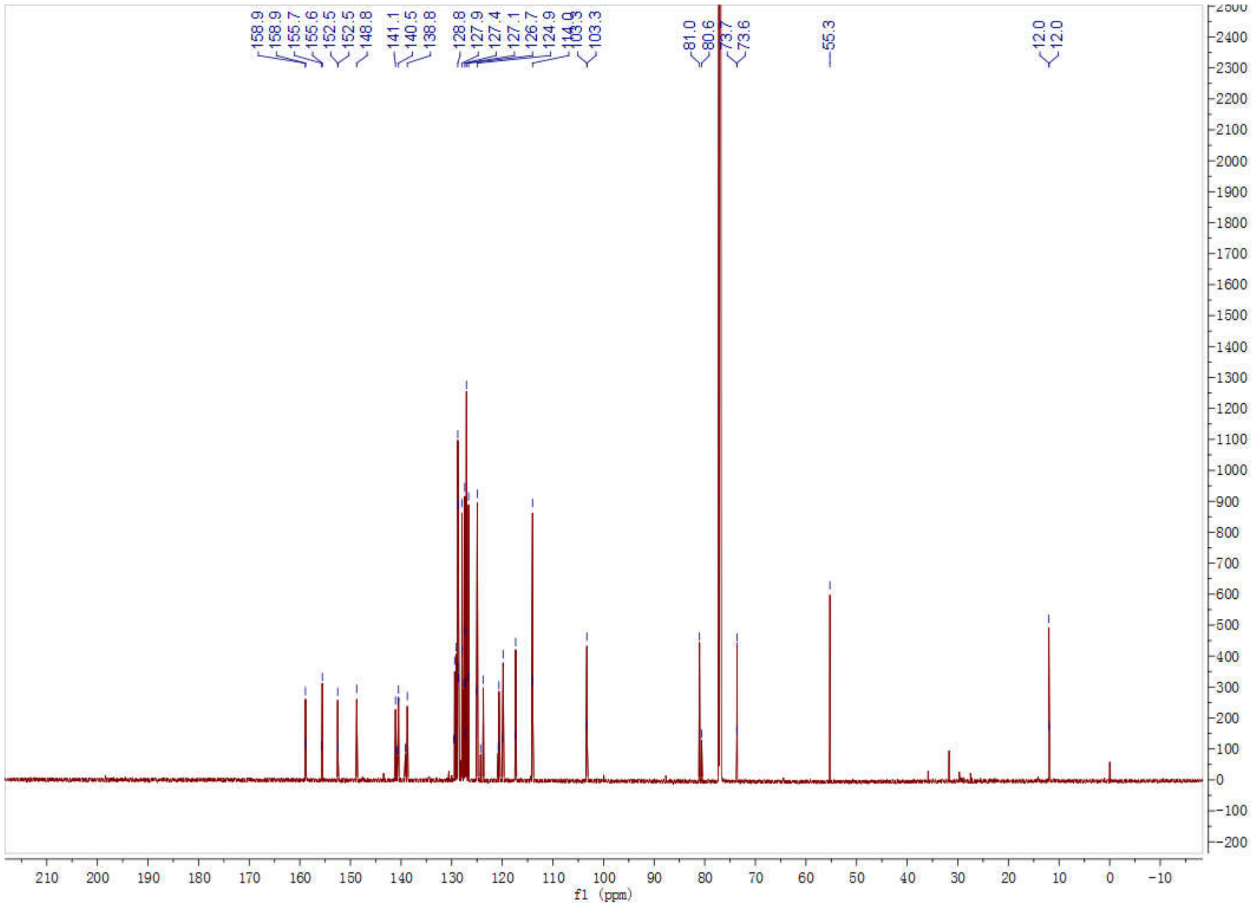

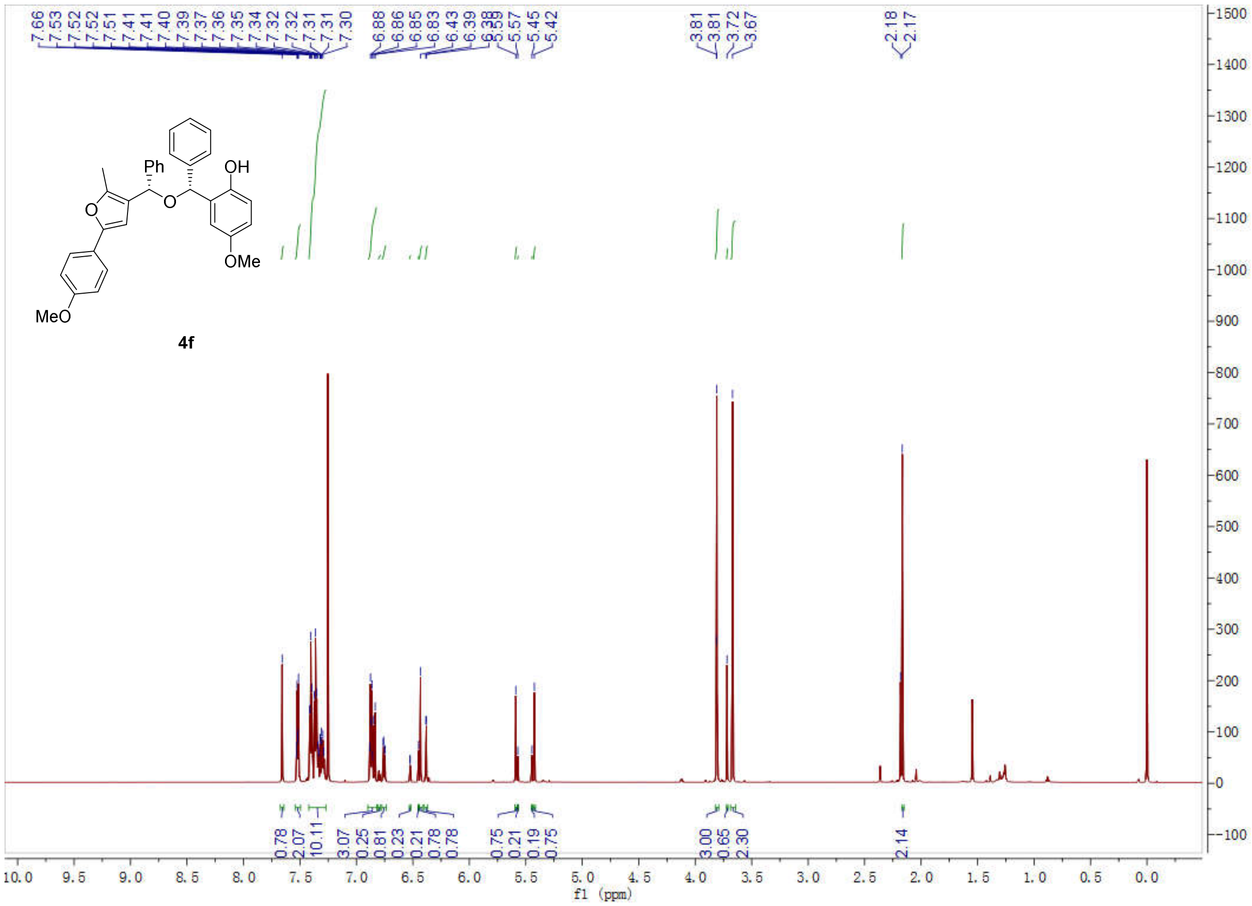
4f
OH
Ph
O
OMe
MeO
f1 (ppm)

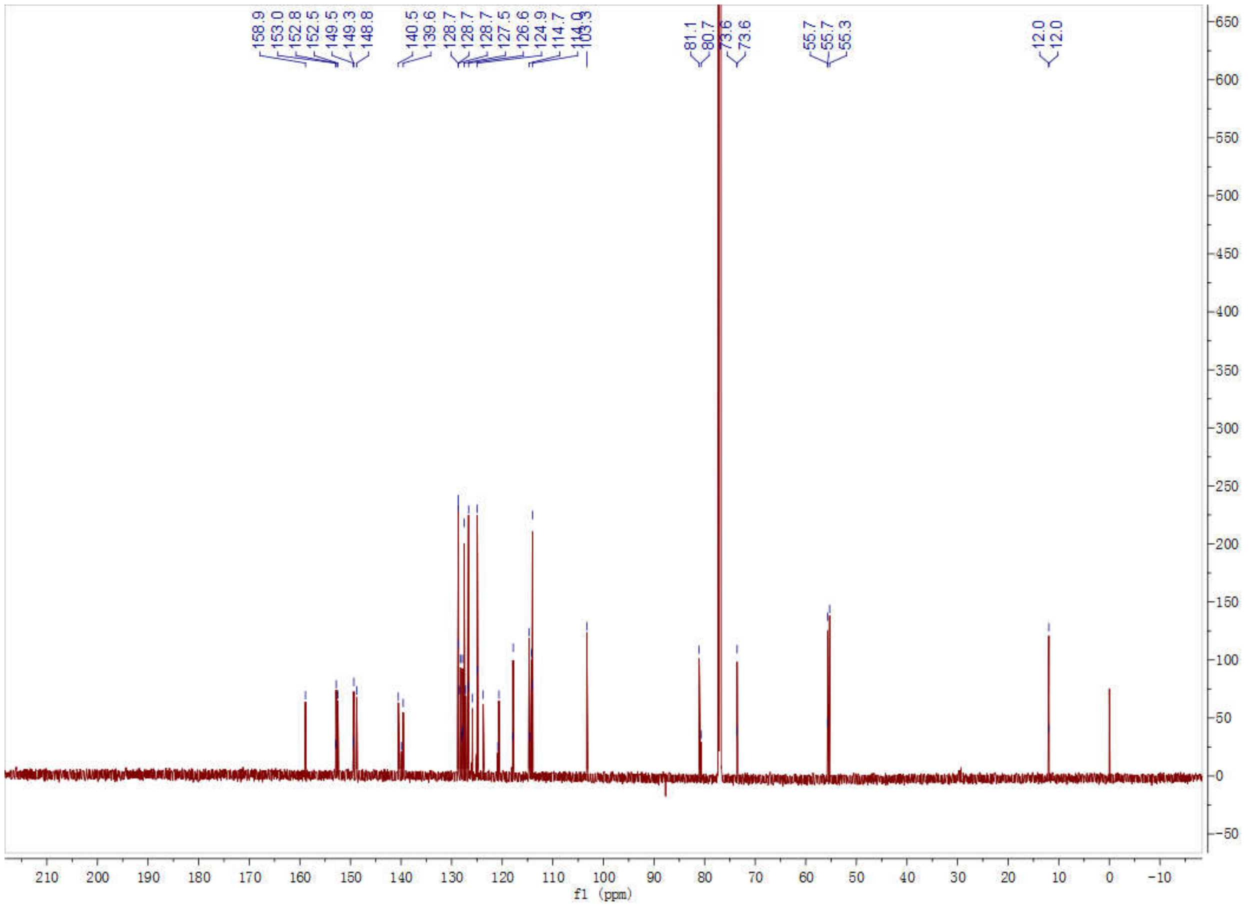
f1 (ppm)

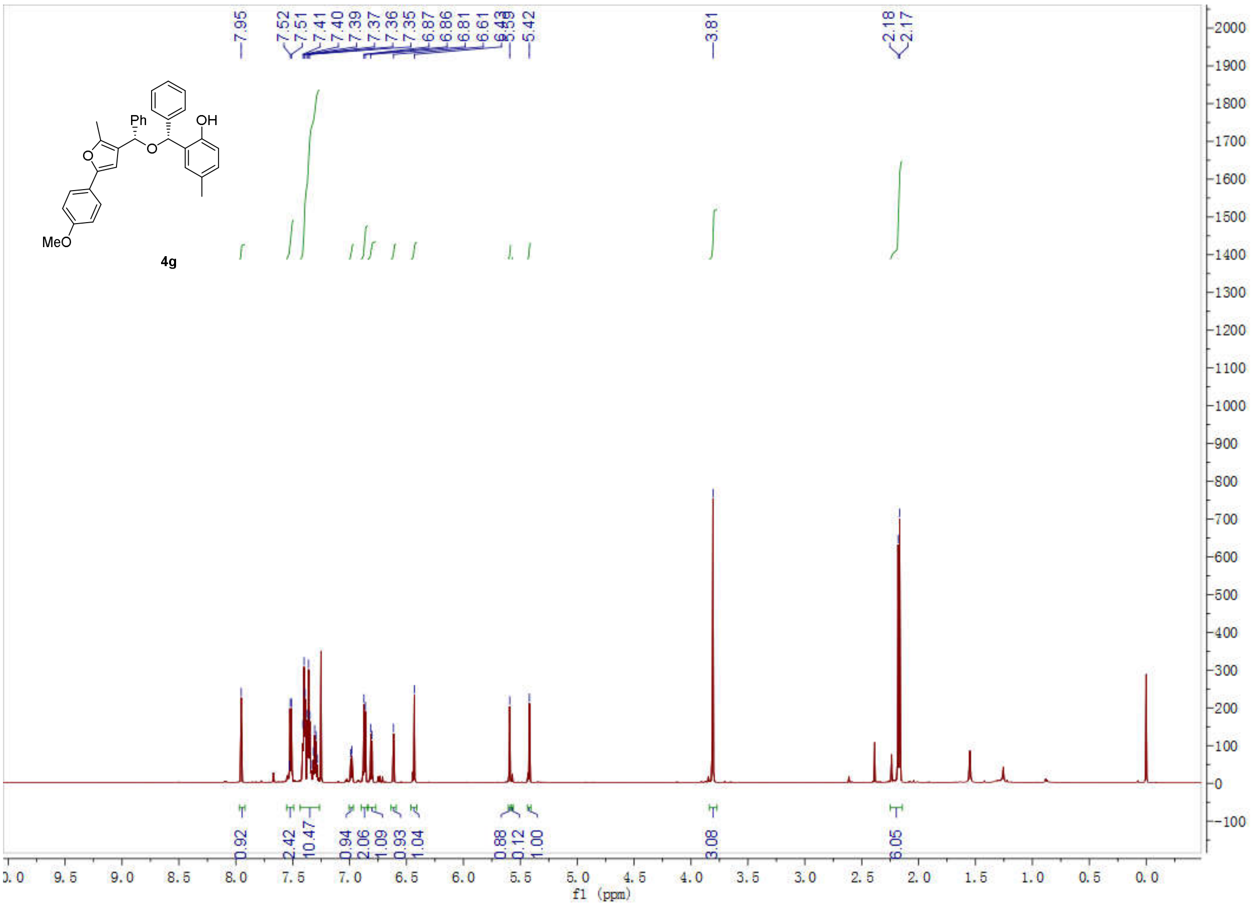
7.95
7.52
7.51
7.41
7.40
7.39
7.37
7.36
7.35
6.87
6.86
6.81
6.61
6.43
5.59
5.42
3.81
2.18
2.17
Ph
OH
O
O
MeO
4g
0.92
2.42
10.47
0.94
2.06
1.09
0.93
1.04
0.88
0.12
1.00
3.08
6.05
f1 (ppm)

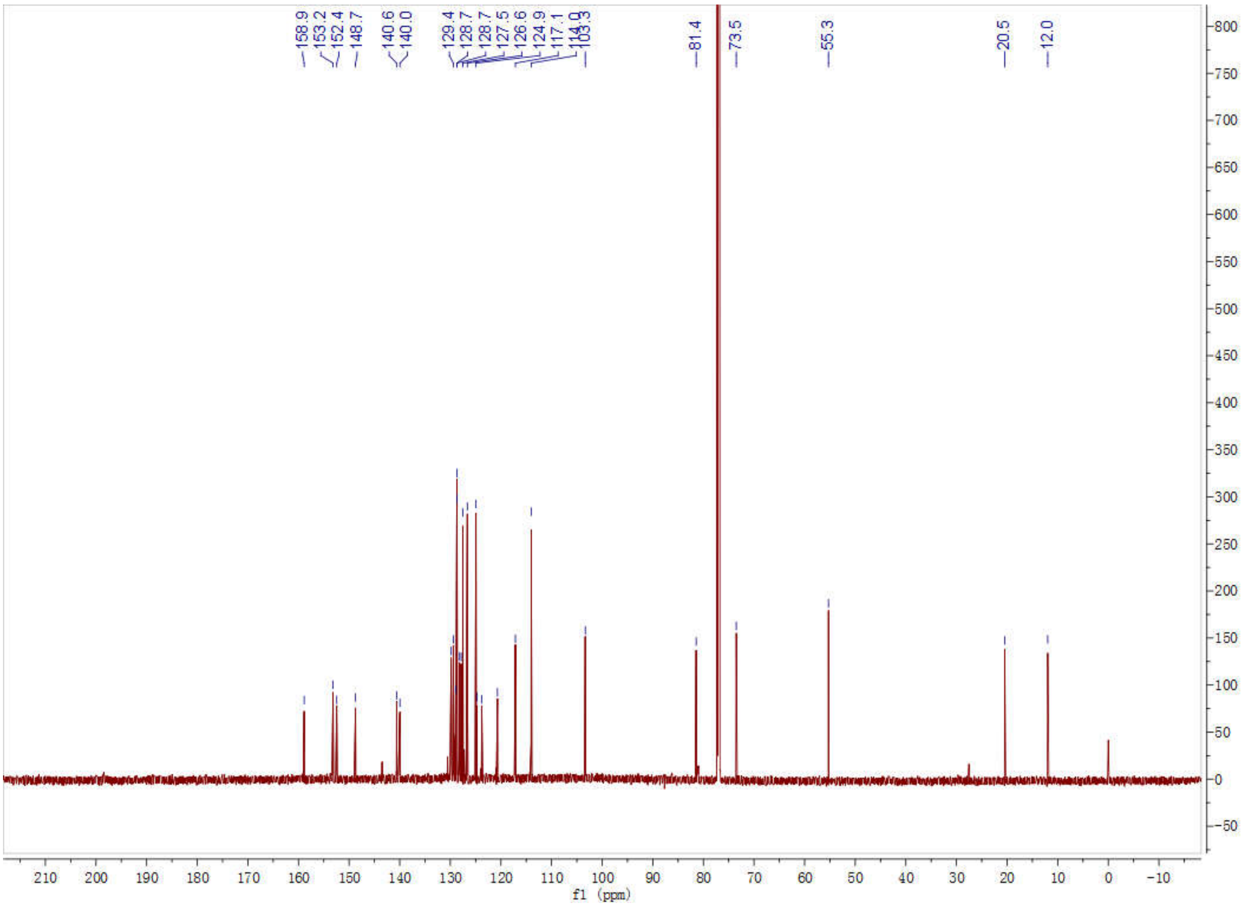
158.9
153.2
152.4
148.7
140.6
140.0
129.4
128.7
128.7
127.5
126.6
124.9
117.1
114.0
103.3
81.4
73.5
55.3
20.5
12.0
f1 (ppm)

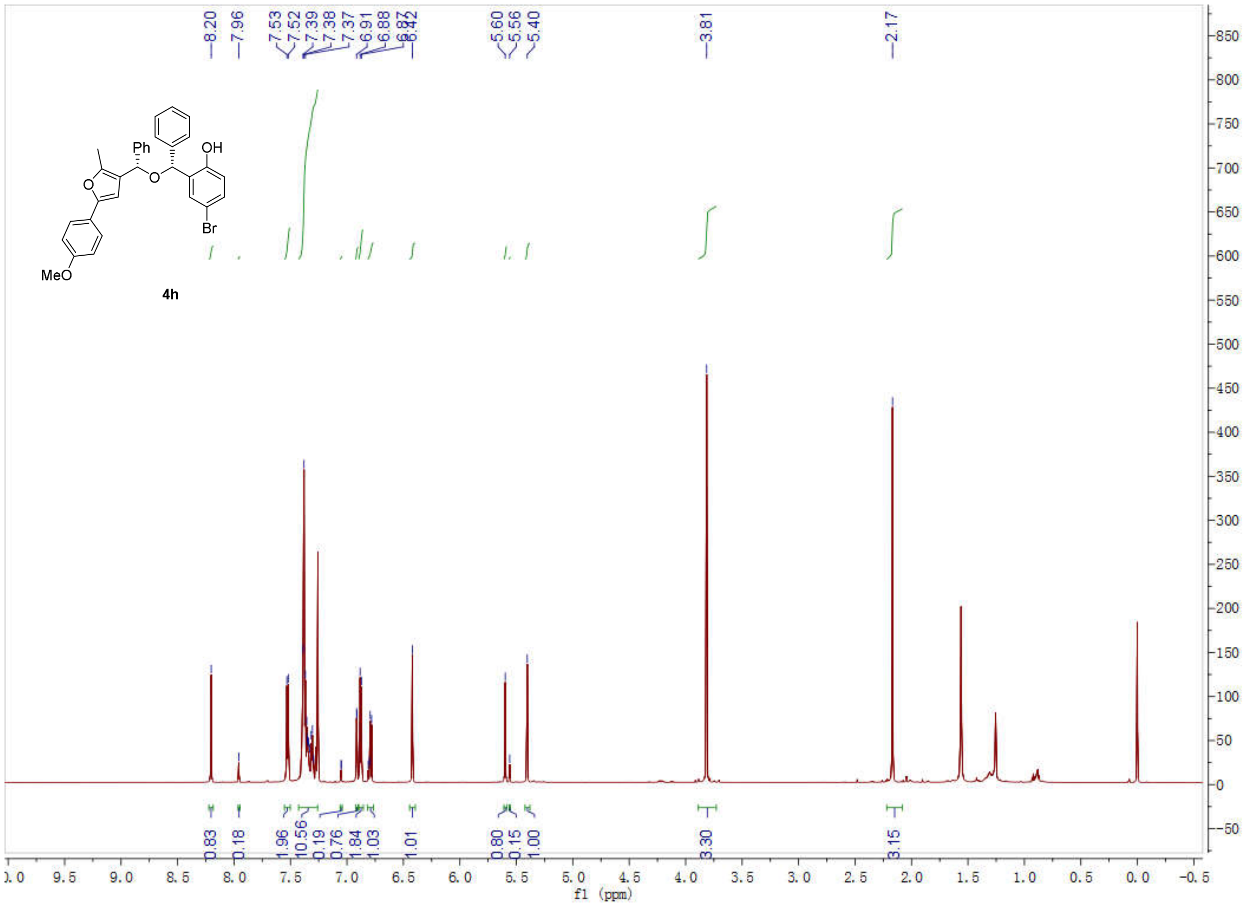
8.20
7.96
7.53
7.52
7.39
7.38
7.37
6.91
6.88
6.82
6.42
5.60
5.56
5.40
3.81
2.17
Ph
OH
O
Br
MeO
4h
0.83
0.18
1.96
10.56
0.19
0.76
1.84
1.03
1.01
0.80
0.15
1.00
3.30
3.15
f1 (ppm)

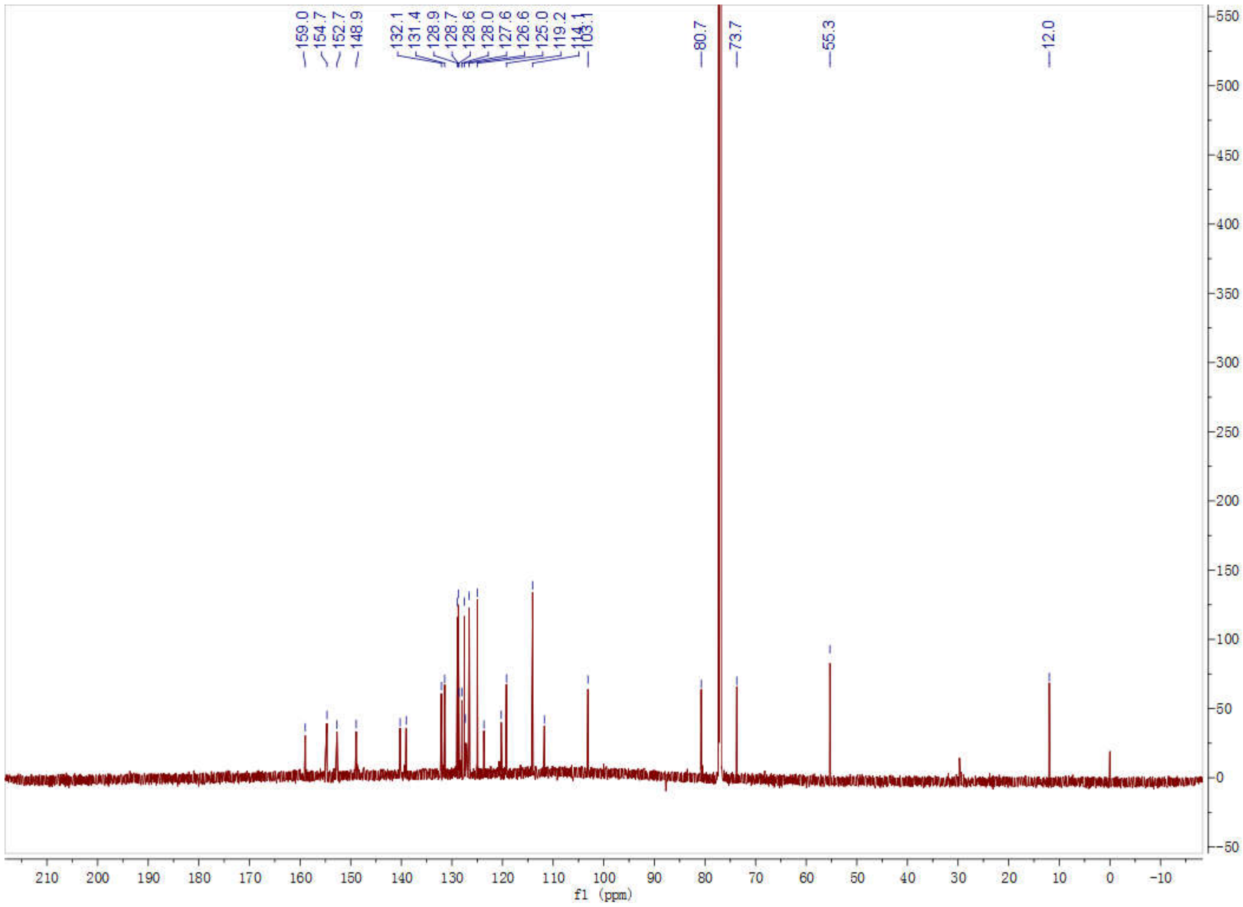
159.0
154.7
152.7
148.9
132.1
131.4
128.9
128.7
128.6
128.0
127.6
126.6
125.0
119.2
114.1
103.1
80.7
73.7
55.3
12.0
f1 (ppm)

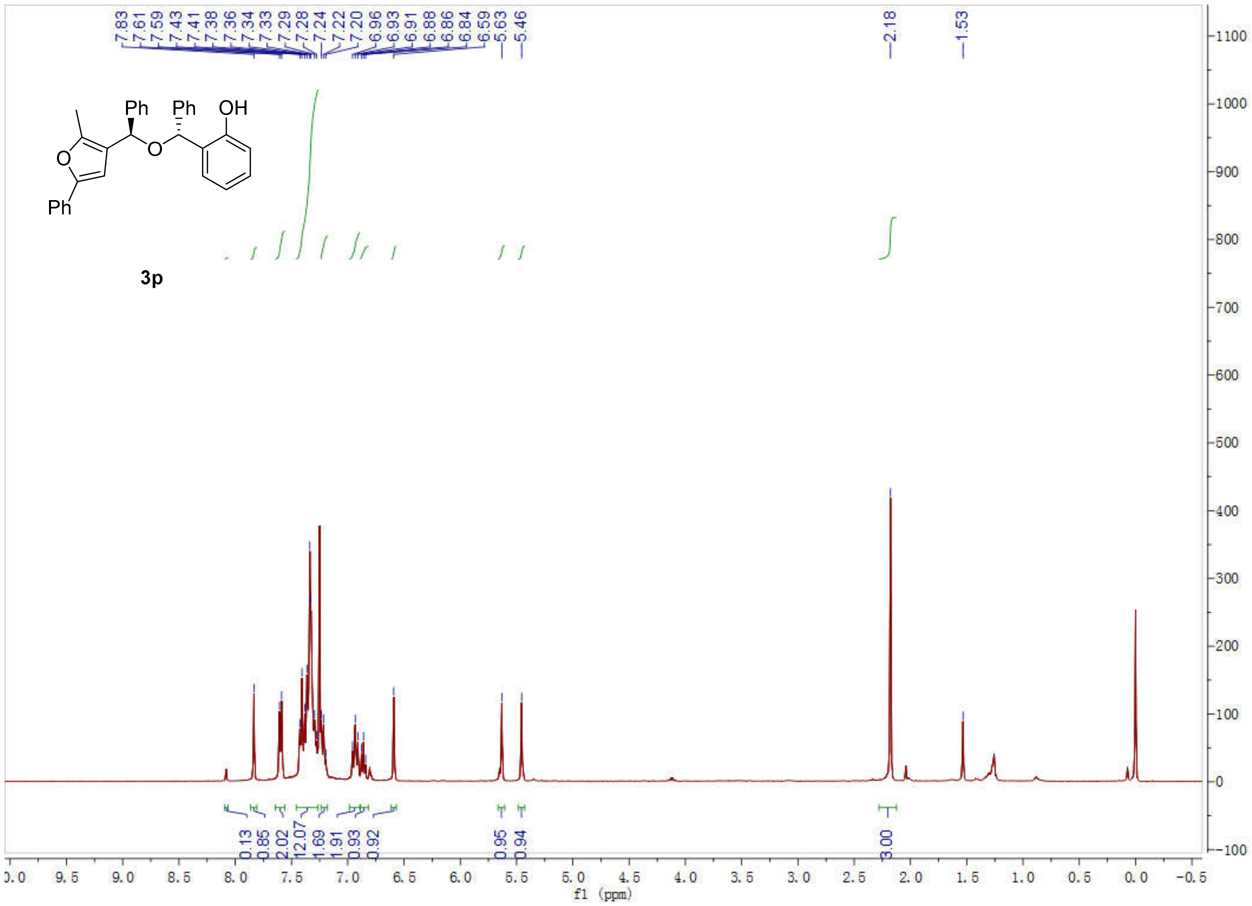
7.83 7.61 7.59 7.43 7.41 7.38 7.36 7.34 7.33 7.29 7.28 7.24 7.22 7.20 6.96 6.93 6.91 6.88 6.86 6.84 6.59 5.63 5.46
2.18
1.53
Ph
Ph
OH
O
O
Ph
3p
0.13 0.85 2.02 12.07 1.69 1.91 0.93 0.92 0.95 0.94 3.00
f1 (ppm)

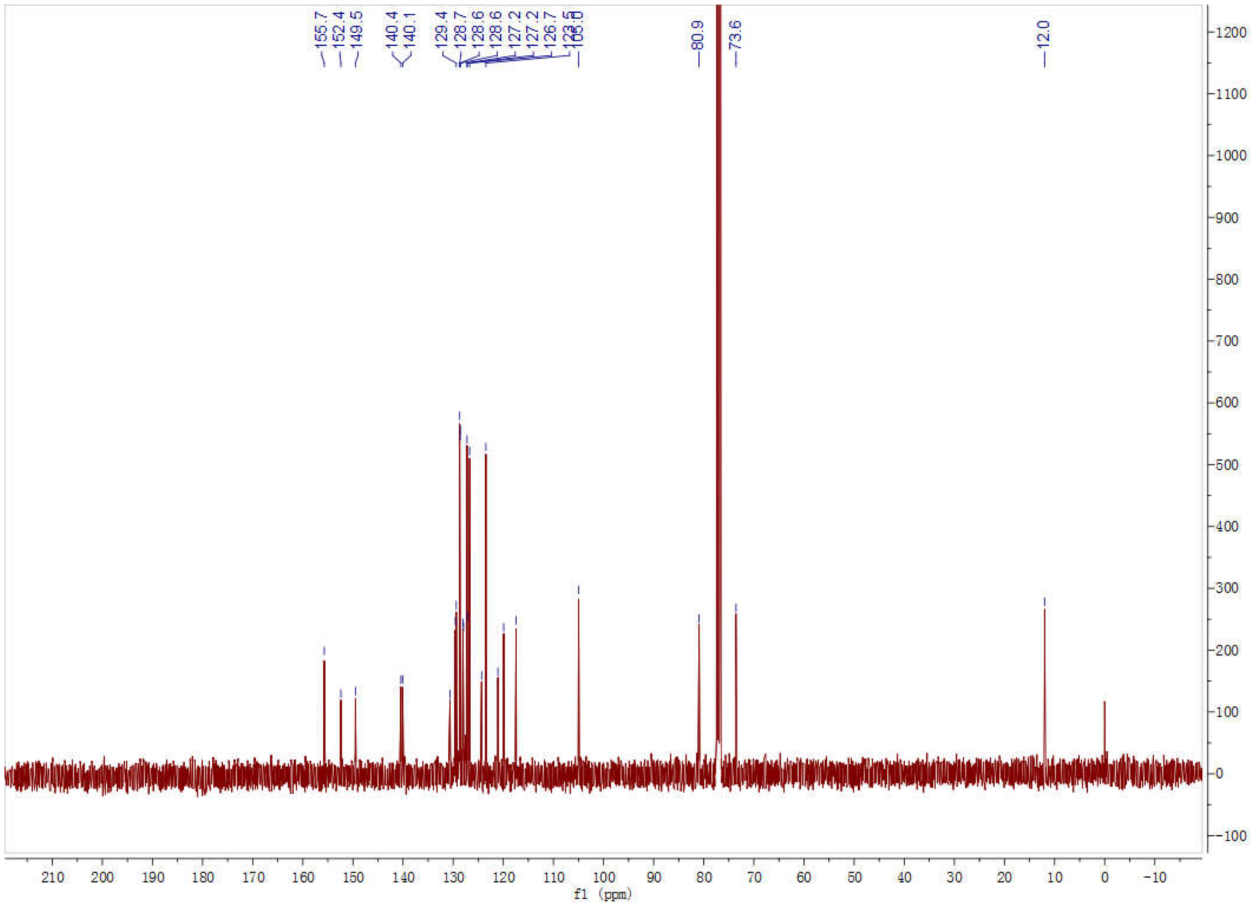
155.7 152.4 149.5
140.4 140.1
129.4 128.7 128.6 128.6 127.2 126.7
80.9
73.6
12.0
f1 (ppm)

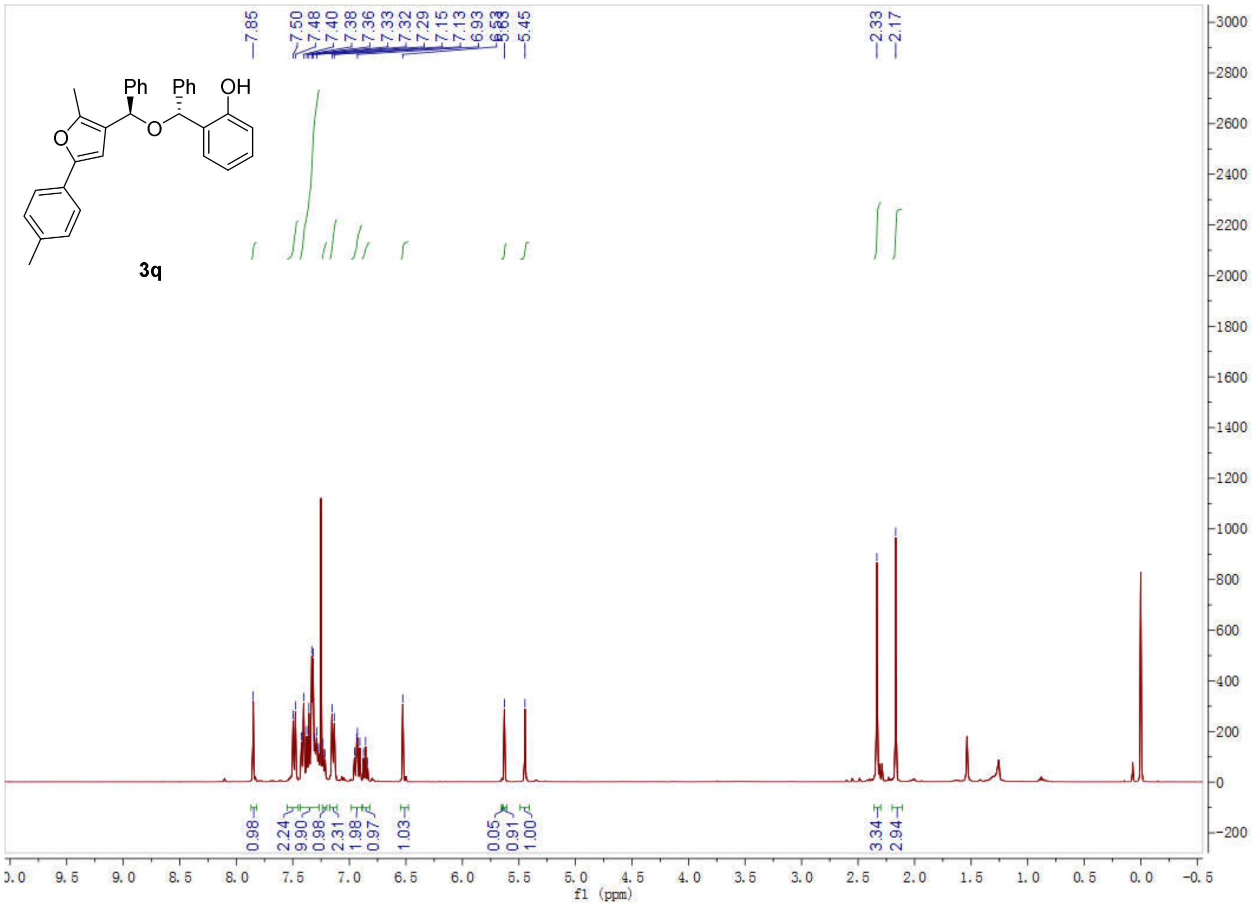
7.85
7.50
7.48
7.40
7.38
7.36
7.33
7.32
7.29
7.15
7.13
6.93
6.53
5.63
5.45
2.33
2.17
Ph
Ph
OH
O
O
3q
0.98
2.24
9.90
0.98
2.31
1.98
0.97
1.03
0.05
0.91
1.00
3.34
2.94
f1 (ppm)

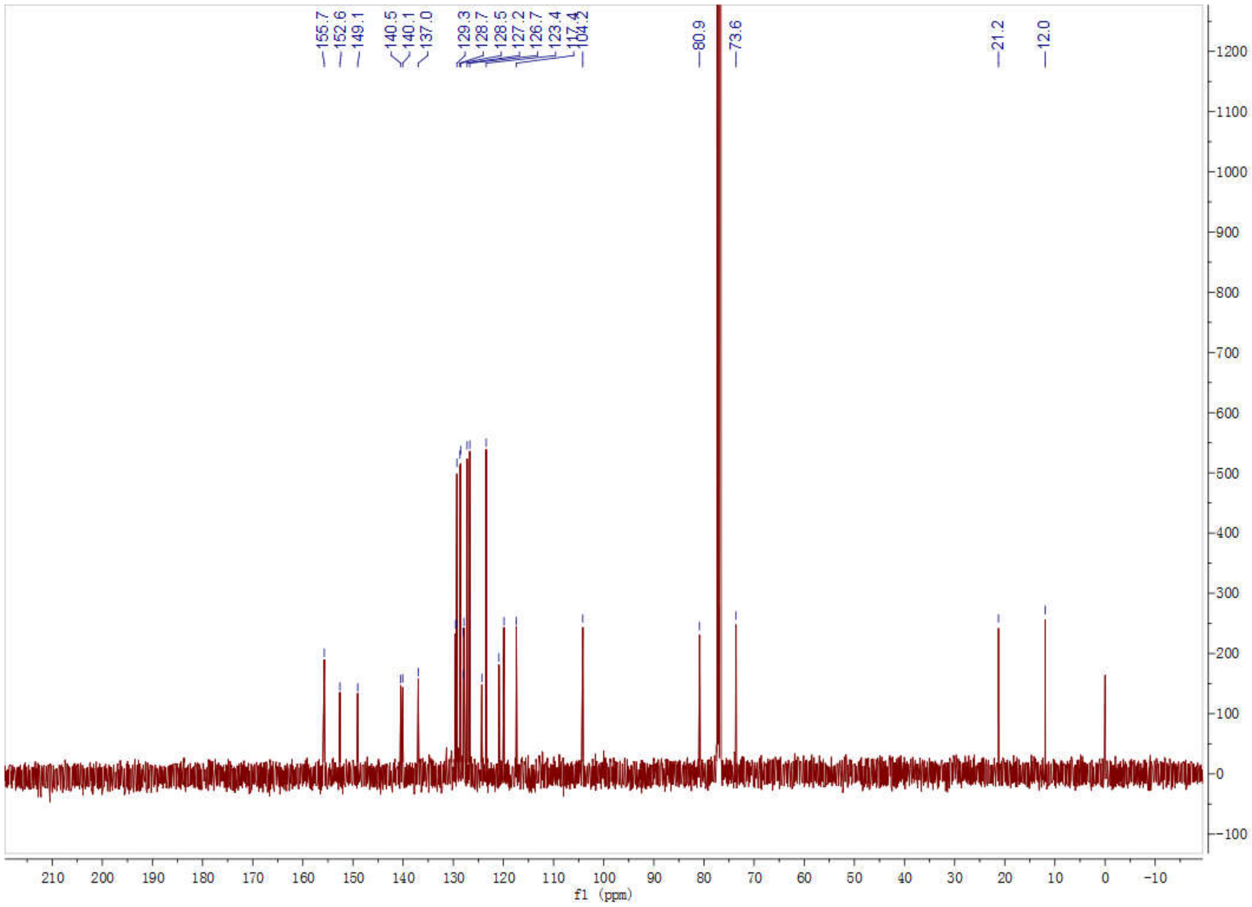
155.7
152.6
149.1
140.5
140.1
137.0
129.3
128.7
128.5
127.2
126.7
123.4
117.4
104.2
80.9
73.6
21.2
12.0
f1 (ppm)

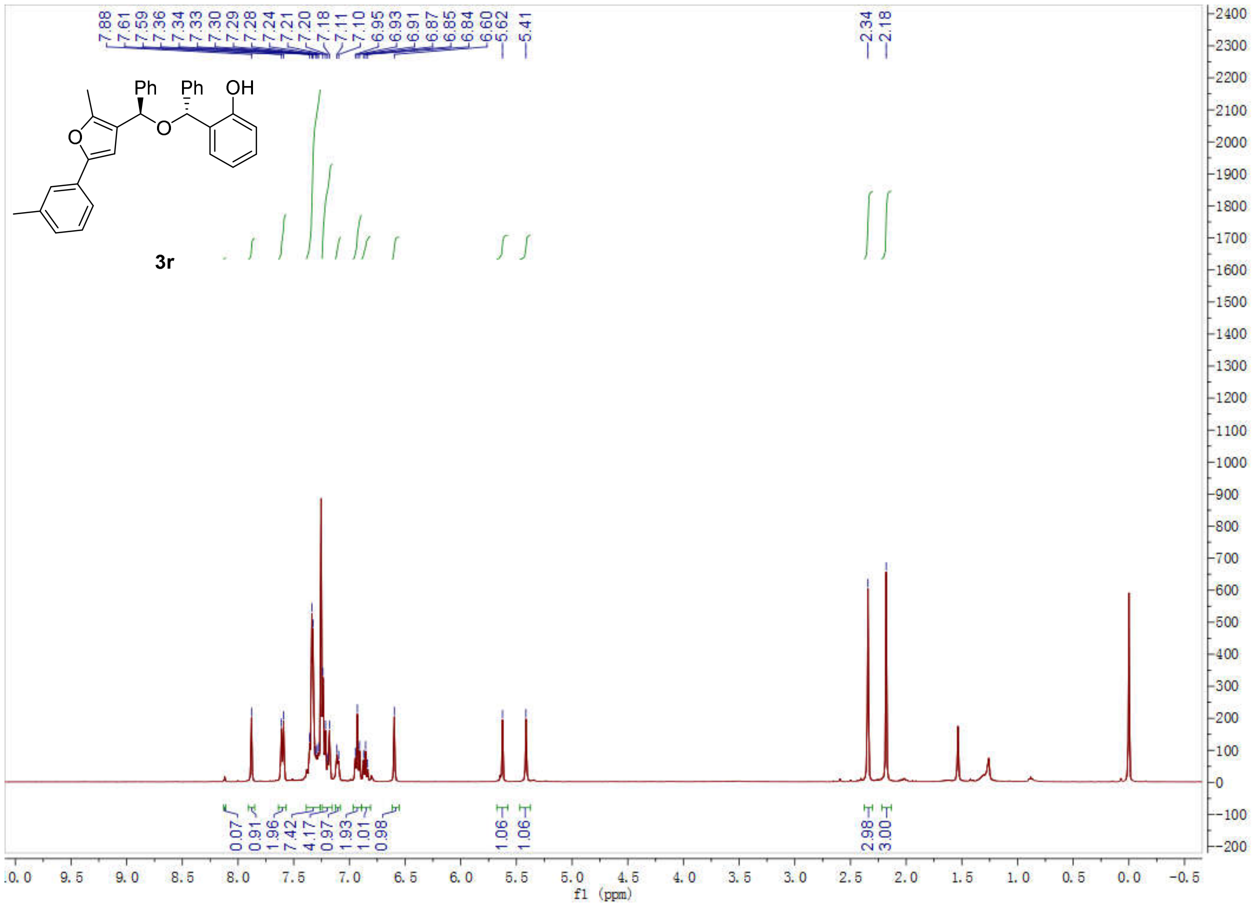
7.88 7.61 7.59 7.36 7.34 7.33 7.30 7.29 7.28 7.24 7.21 7.20 7.18 7.11 7.10 6.95 6.93 6.91 6.87 6.85 6.84 6.60 5.62 5.41
2.34 2.18
Ph
Ph
OH
O
O
3r
0.07 0.91 1.96 7.42 4.17 0.97 1.93 1.01 0.98 1.06 1.06 2.98 3.00
f1 (ppm)

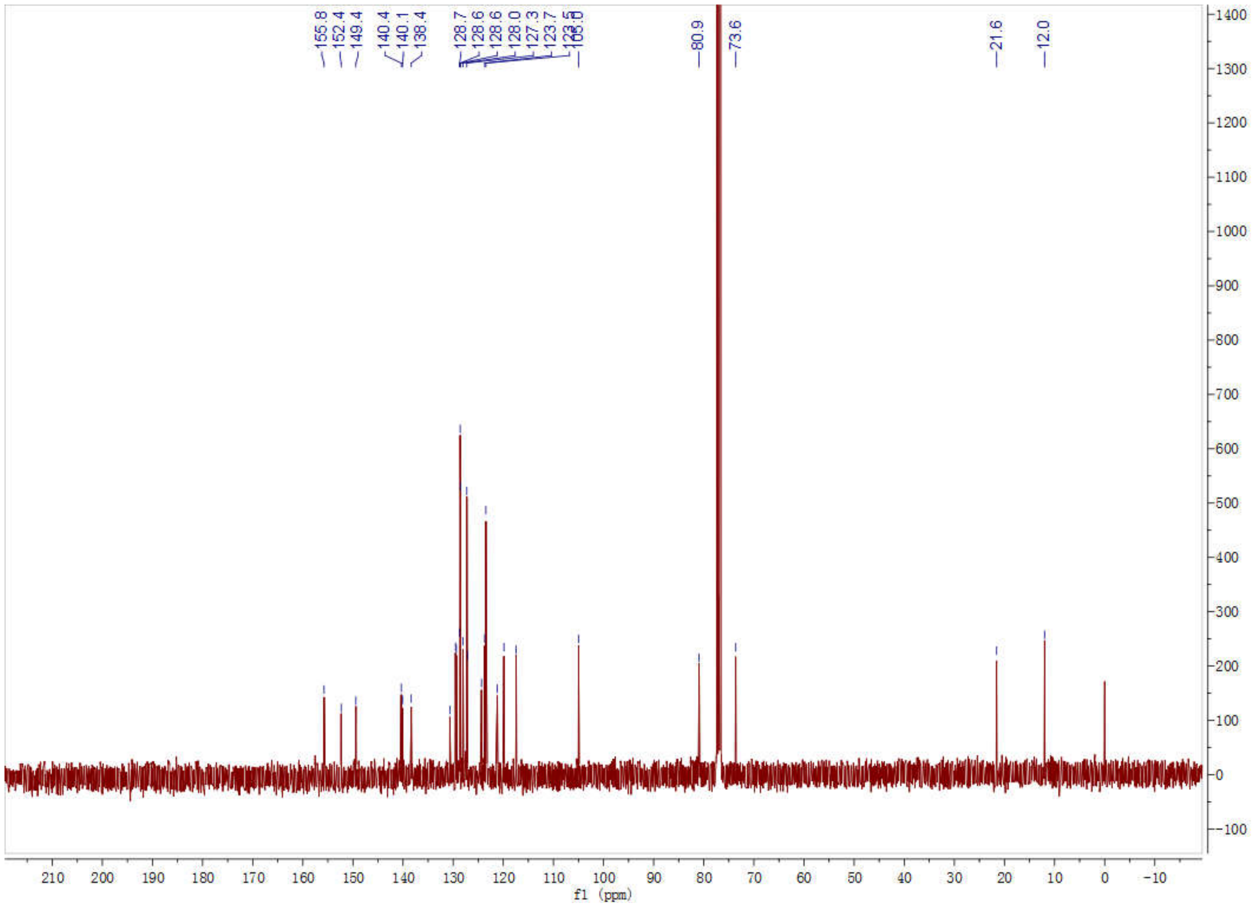
155.8 152.4 149.4 140.4 140.1 138.4 128.7 128.6 128.6 128.0 127.3 123.7 123.5 105.0
80.9 73.6 21.6 12.0
f1 (ppm)

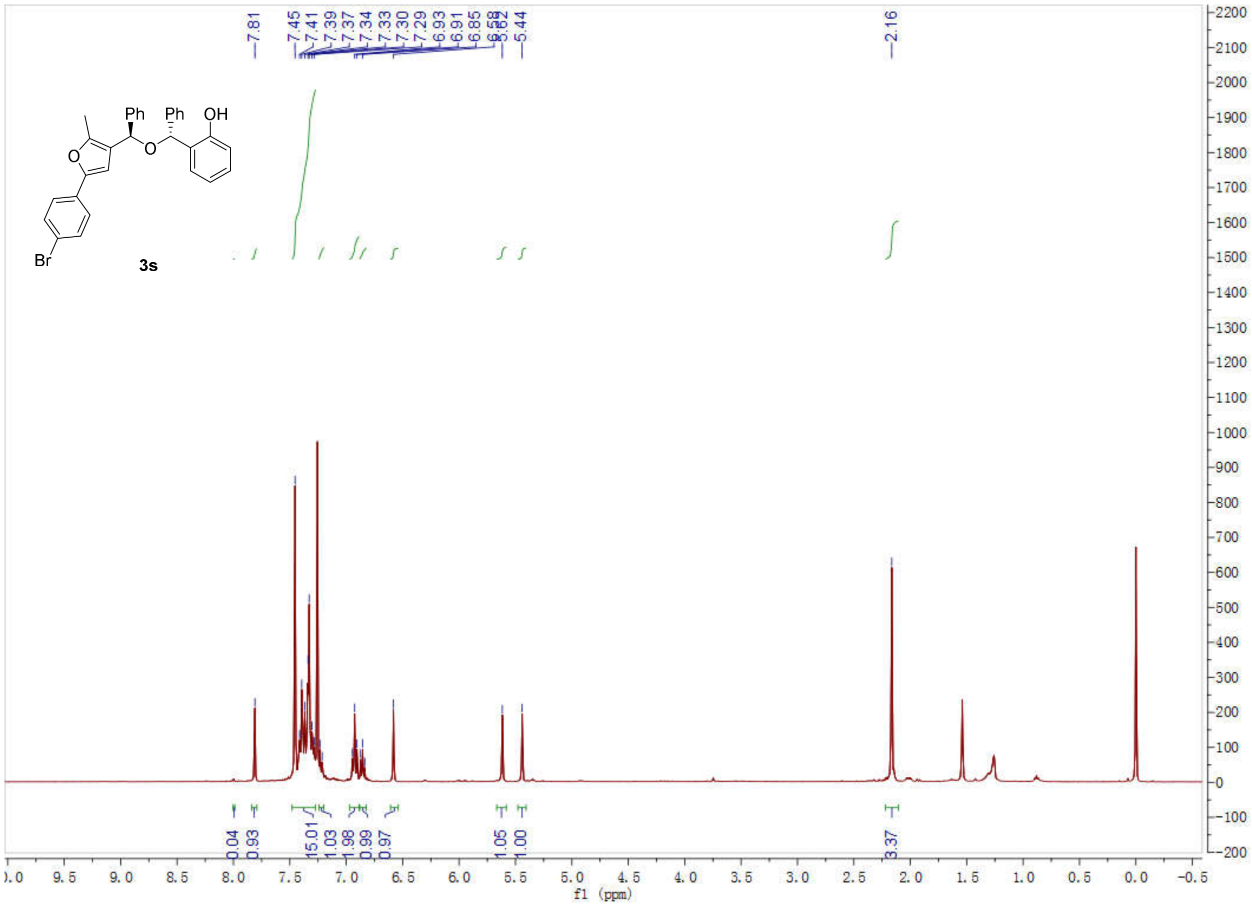
7.81
7.45
7.41
7.39
7.37
7.34
7.33
7.30
7.29
6.93
6.91
6.85
5.62
5.44
2.16
Ph
Ph
OH
O
O
Br
3s
0.04
0.93
15.01
1.03
1.98
0.99
0.97
1.05
1.00
3.37
f1 (ppm)

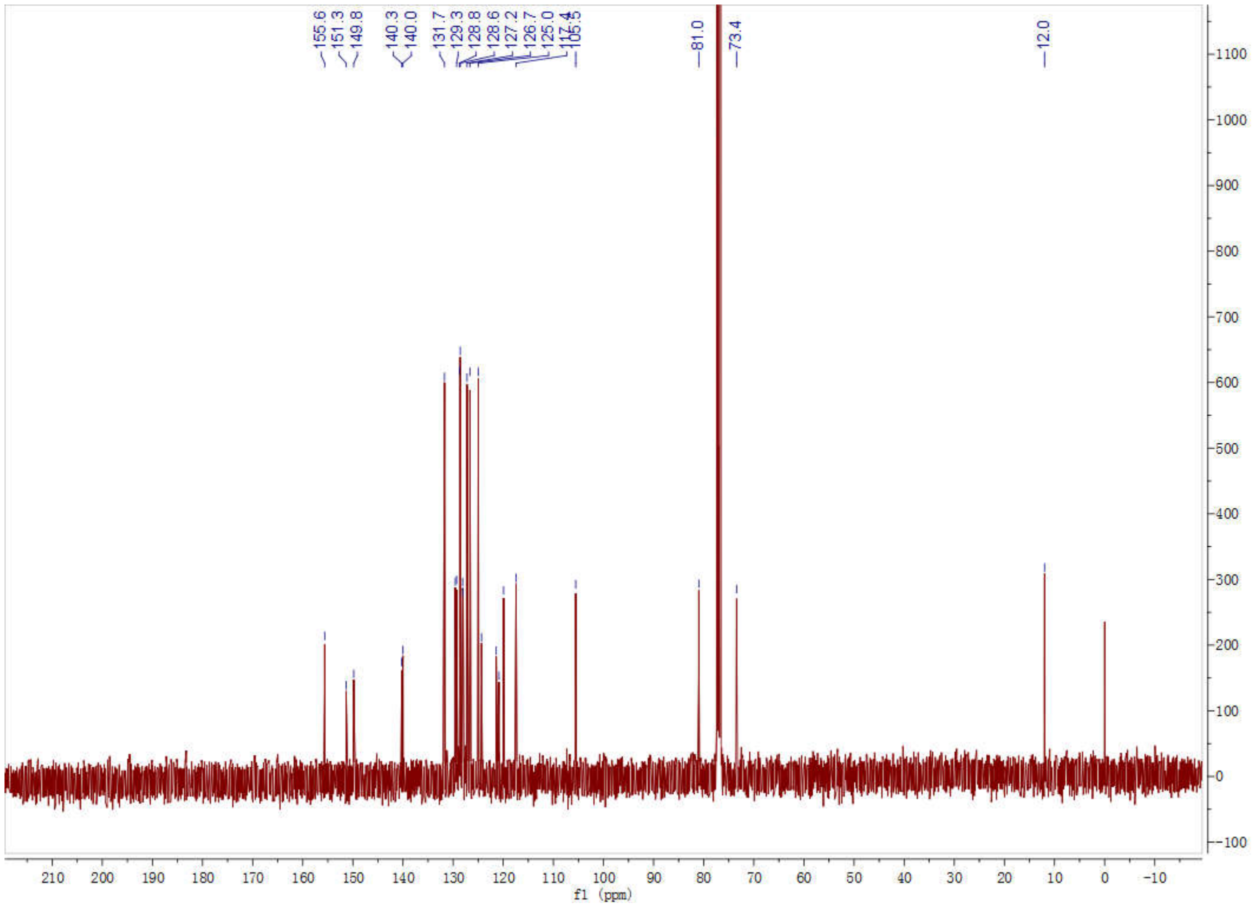
155.6
151.3
149.8
140.3
140.0
131.7
129.3
128.8
128.6
127.2
126.7
125.0
81.0
73.4
12.0
f1 (ppm)

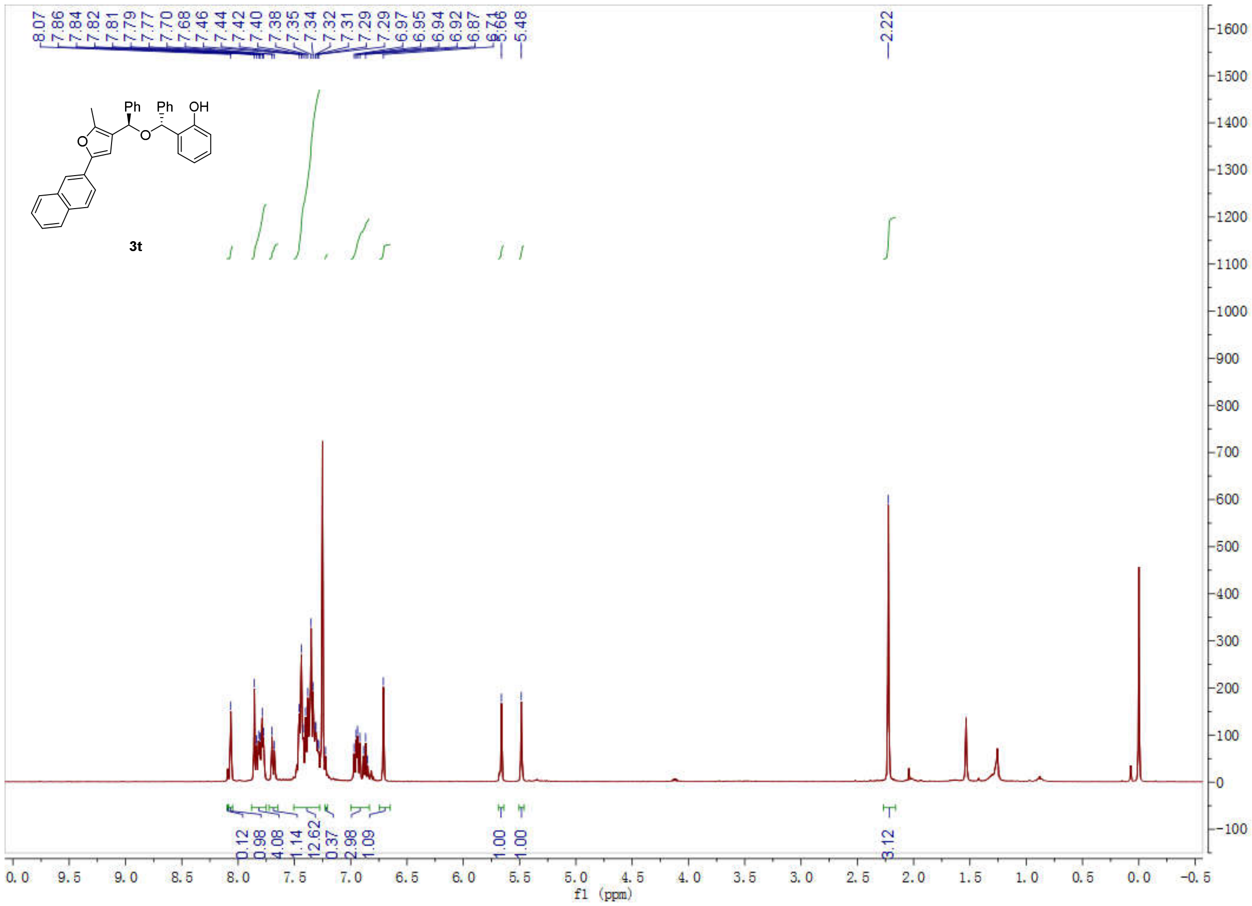
8.07 7.86 7.84 7.82 7.81 7.79 7.77 7.70 7.68 7.46 7.44 7.42 7.40 7.38 7.35 7.34 7.32 7.31 7.29 7.29 6.97 6.95 6.94 6.92 6.87 6.71 5.66 5.48
2.22
Ph Ph OH
O
3t
0.12 0.98 4.08 1.14 12.62 0.37 2.98 1.09
1.00 1.00
3.12
f1 (ppm)

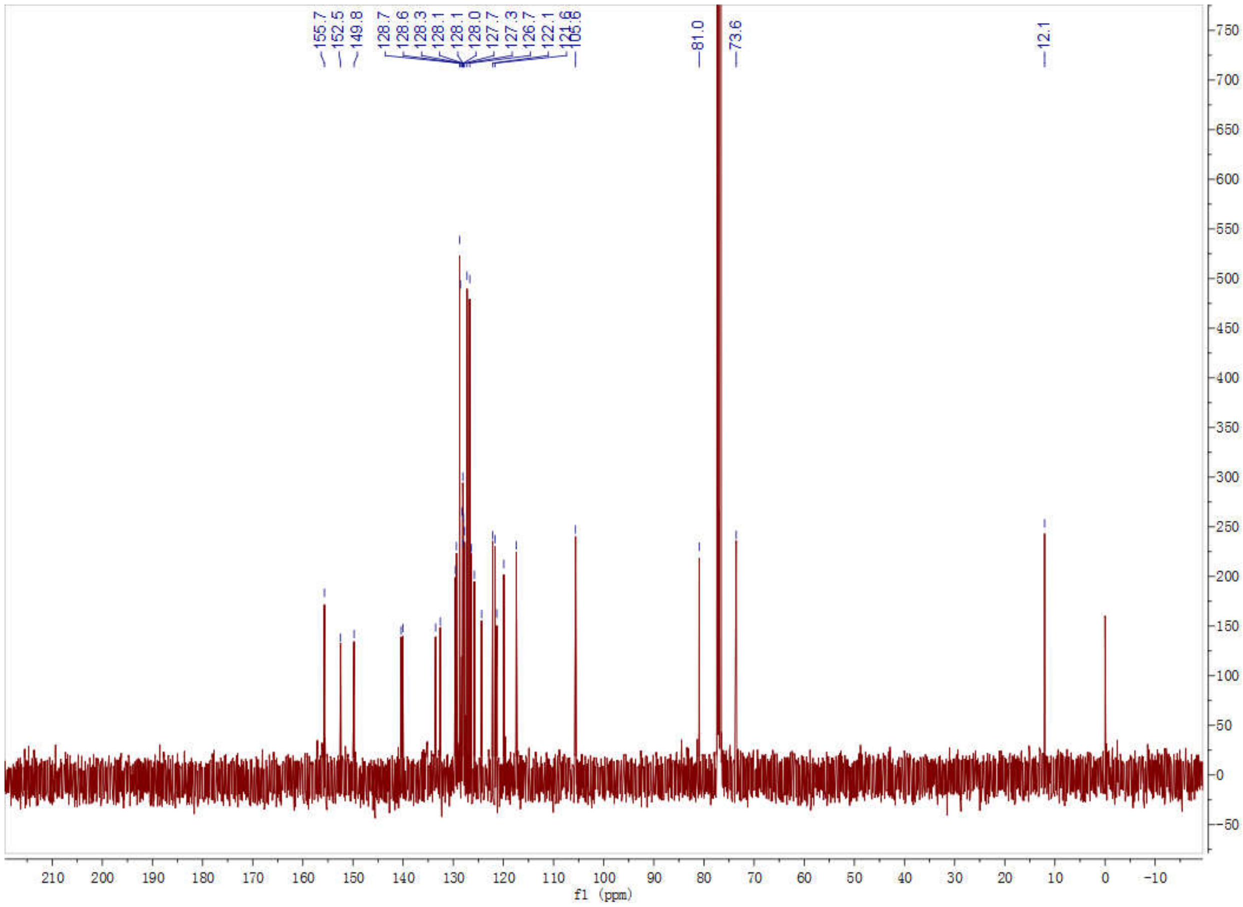
155.7 152.5 149.8
128.7 128.6 128.3 128.1 128.1 128.0 127.7 127.3 126.7 122.1 121.6 105.8
81.0 73.6
12.1
f1 (ppm)

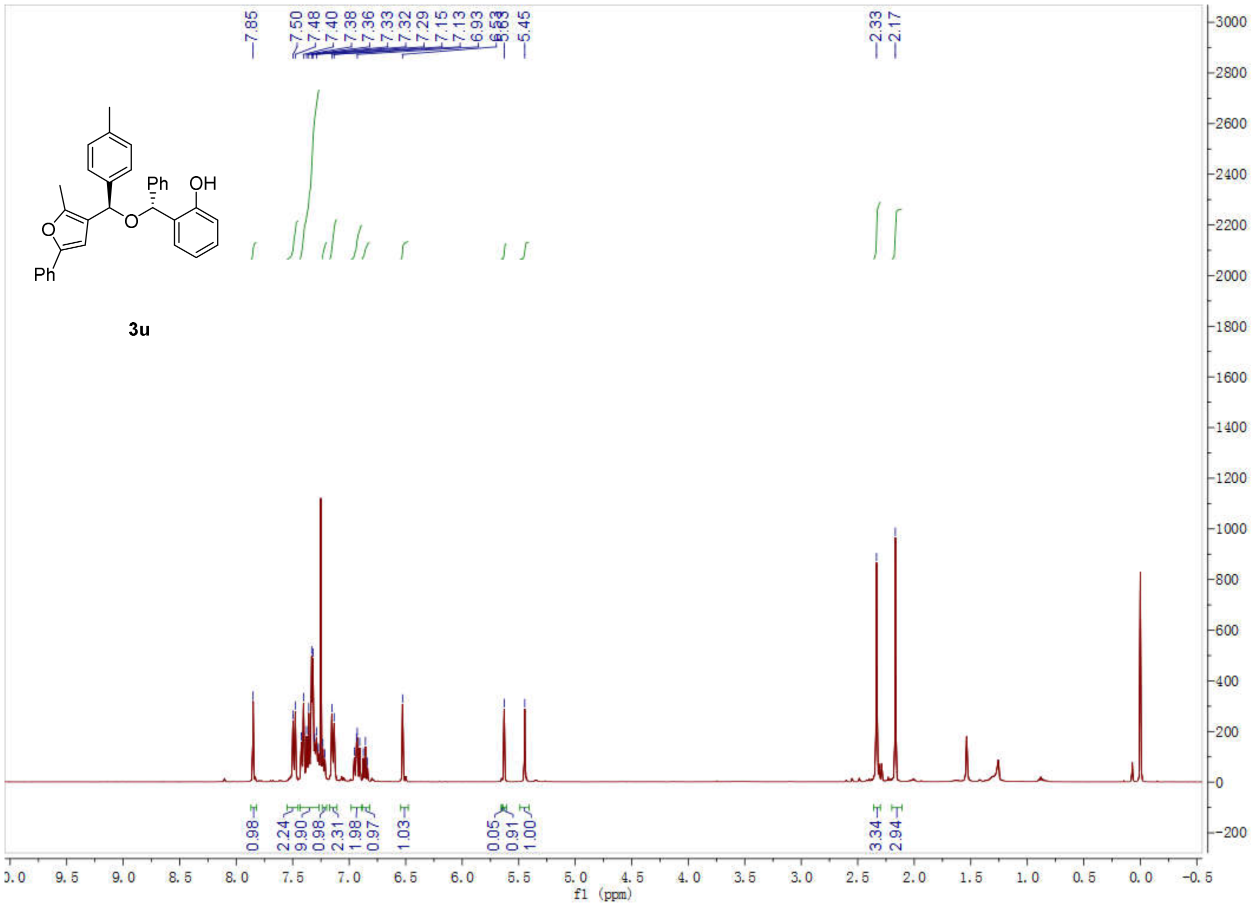
7.85
7.50
7.48
7.40
7.38
7.36
7.33
7.32
7.29
7.15
7.13
6.93
6.53
5.63
5.45
2.33
2.17
Ph
OH
O
O
Ph
3u
0.98
2.24
9.90
0.98
2.31
1.98
0.97
1.03
0.05
0.91
1.00
3.34
2.94
f1 (ppm)

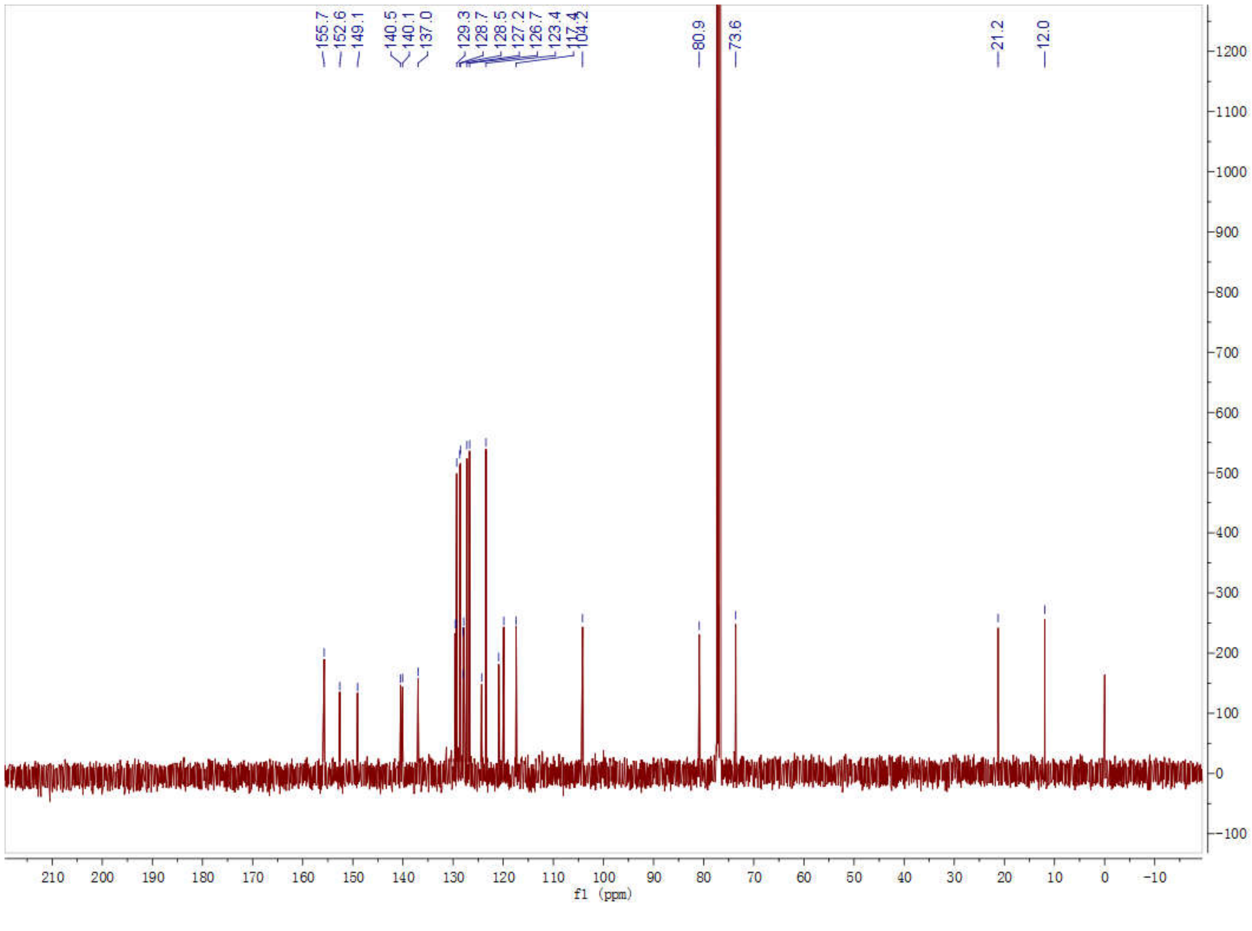
155.7
152.6
149.1
140.5
140.1
137.0
129.3
128.7
128.5
127.2
126.7
123.4
117.4
104.2
80.9
73.6
21.2
12.0
f1 (ppm)

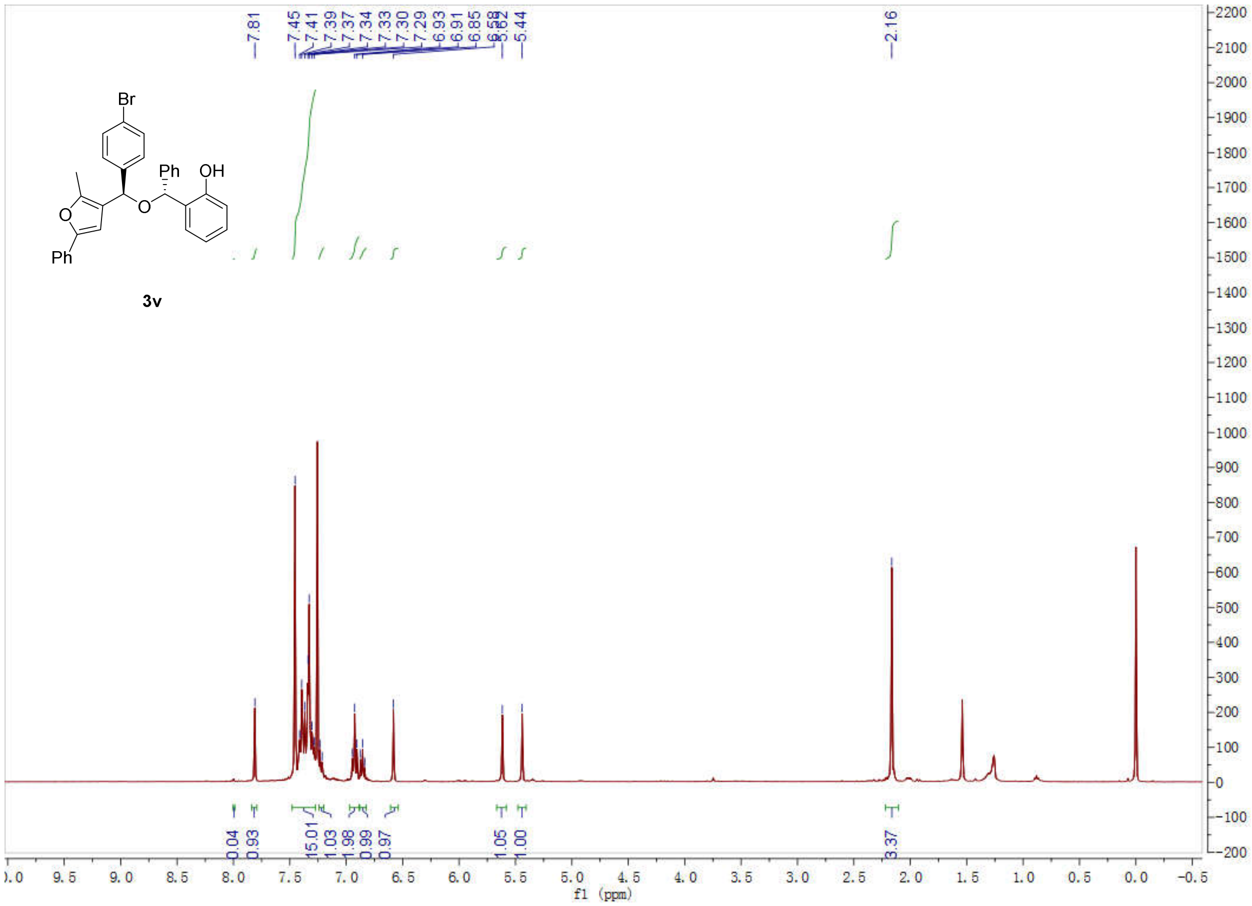

7.81
7.45
7.41
7.39
7.37
7.34
7.33
7.30
7.29
6.93
6.91
6.85
5.44
2.16
Br
Ph
OH
O
O
Ph
3v
0.04
0.93
15.01
1.03
1.98
0.99
0.97
1.05
1.00
3.37
f1 (ppm)

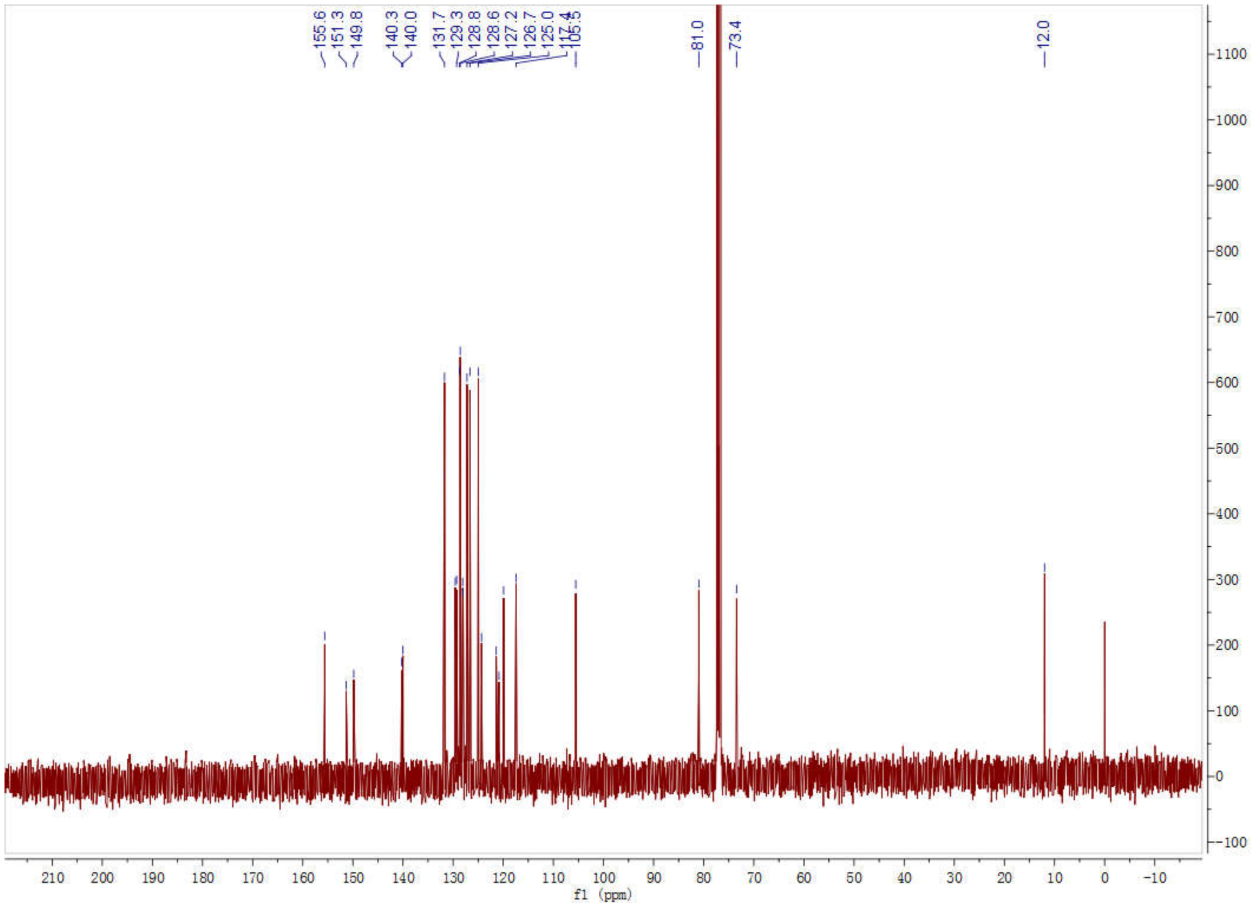

155.6
151.3
149.8
140.3
140.0
131.7
129.3
128.8
128.6
127.2
126.7
125.0
81.0
73.4
12.0
f1 (ppm)

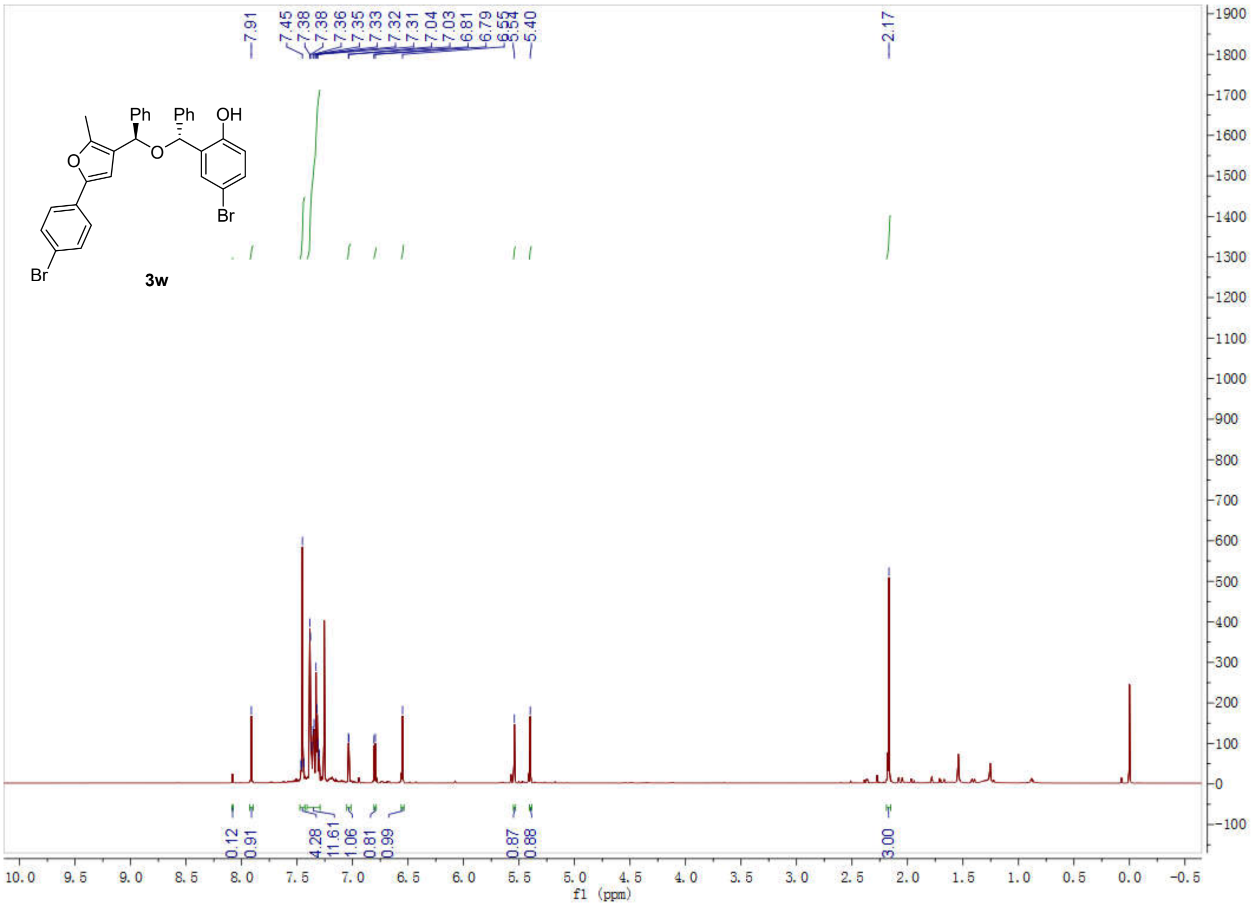

7.91
7.45
7.38
7.38
7.36
7.35
7.33
7.32
7.31
7.04
7.03
6.81
6.79
6.55
5.54
5.40
2.17
Ph
Ph
OH
O
O
Br
Br
3w
0.12
0.91
4.28
11.61
1.06
0.81
0.99
0.87
0.88
3.00
f1 (ppm)

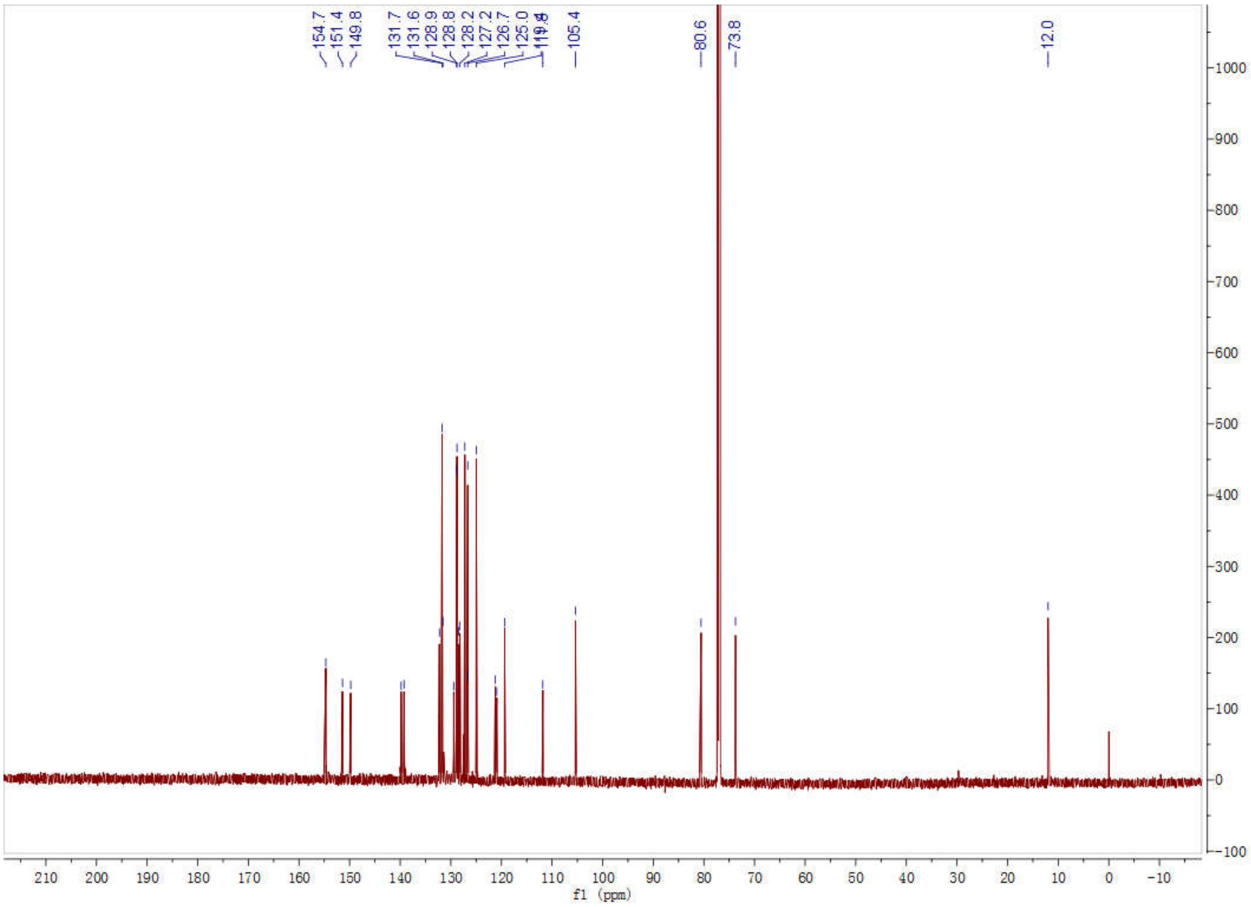

154.7
151.4
149.8
131.7
131.6
128.9
128.8
128.2
127.2
126.7
125.0
105.4
80.6
73.8
12.0
f1 (ppm)

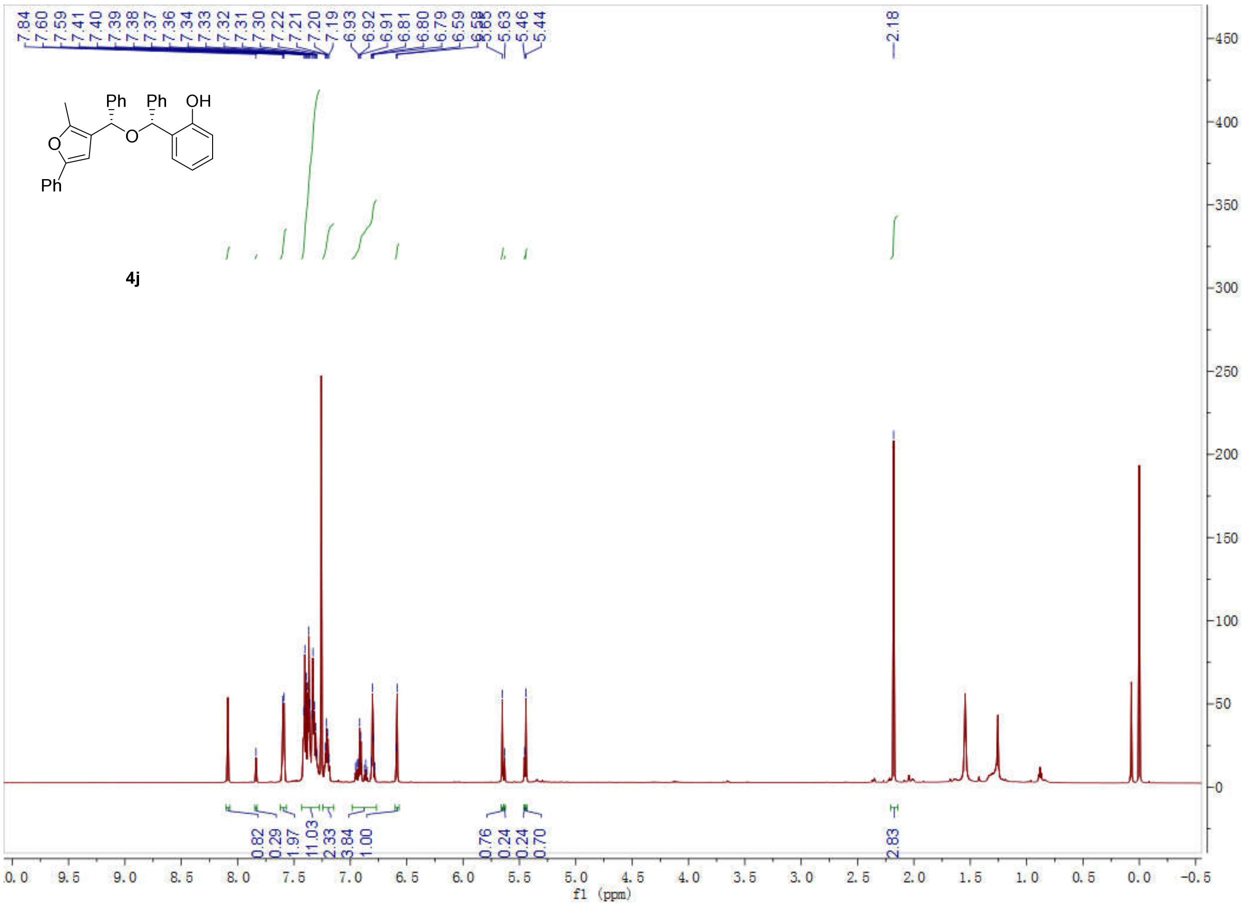
7.84 7.60 7.59 7.41 7.40 7.39 7.38 7.37 7.36 7.34 7.33 7.32 7.31 7.30 7.22 7.21 7.20 7.19 6.93 6.92 6.91 6.81 6.80 6.79 6.59 6.58 5.65 5.63 5.46 5.44
2.18
Ph
Ph
OH
O
O
Ph
4j
0.82 0.29 1.97 11.03 2.33 3.84 1.00 0.76 0.24 0.24 0.70
2.83
f1 (ppm)

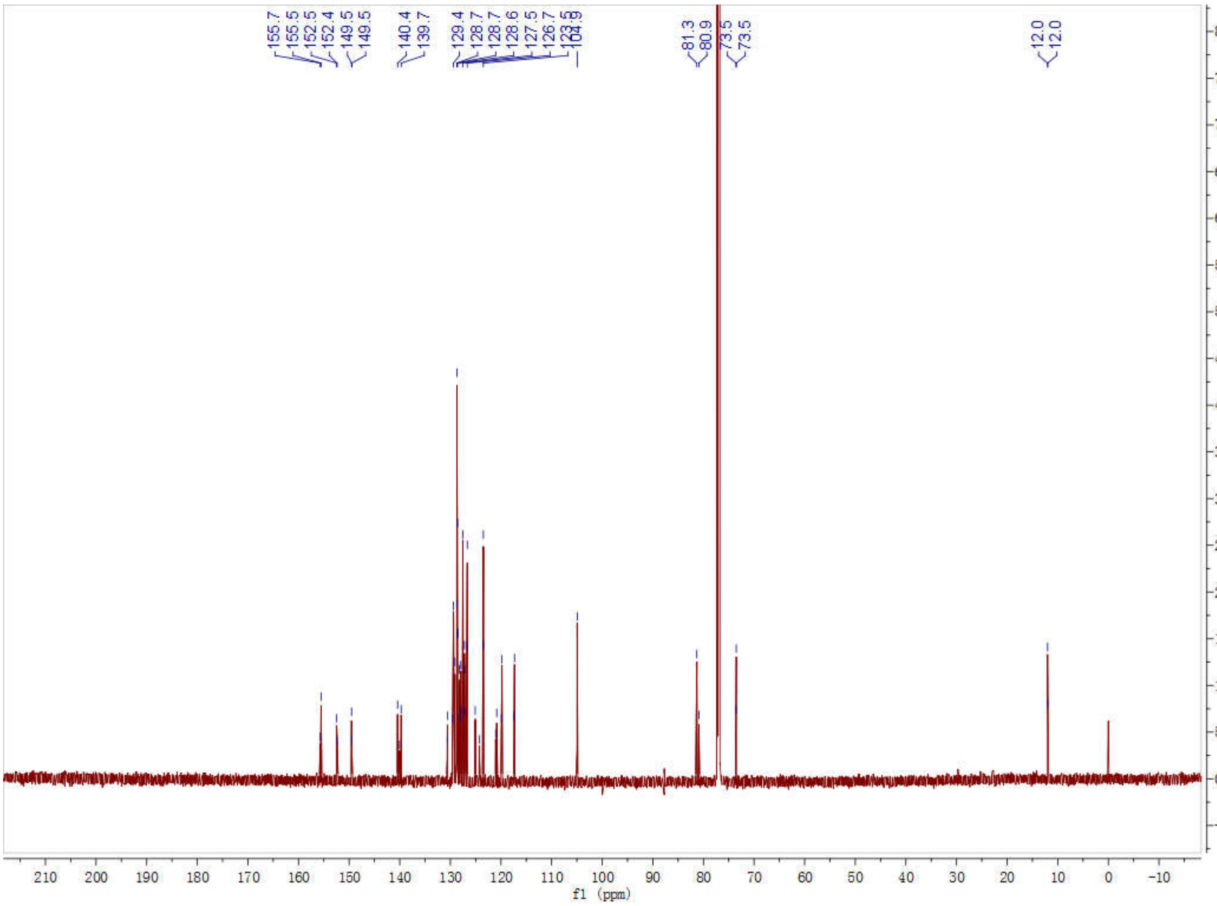
155.7 155.5 152.5 152.4 149.5 149.5
140.4 139.7
129.4 128.7 128.7 128.6 127.5 126.7 123.5 104.9
81.3 80.9 73.5 73.5
12.0 12.0
f1 (ppm)

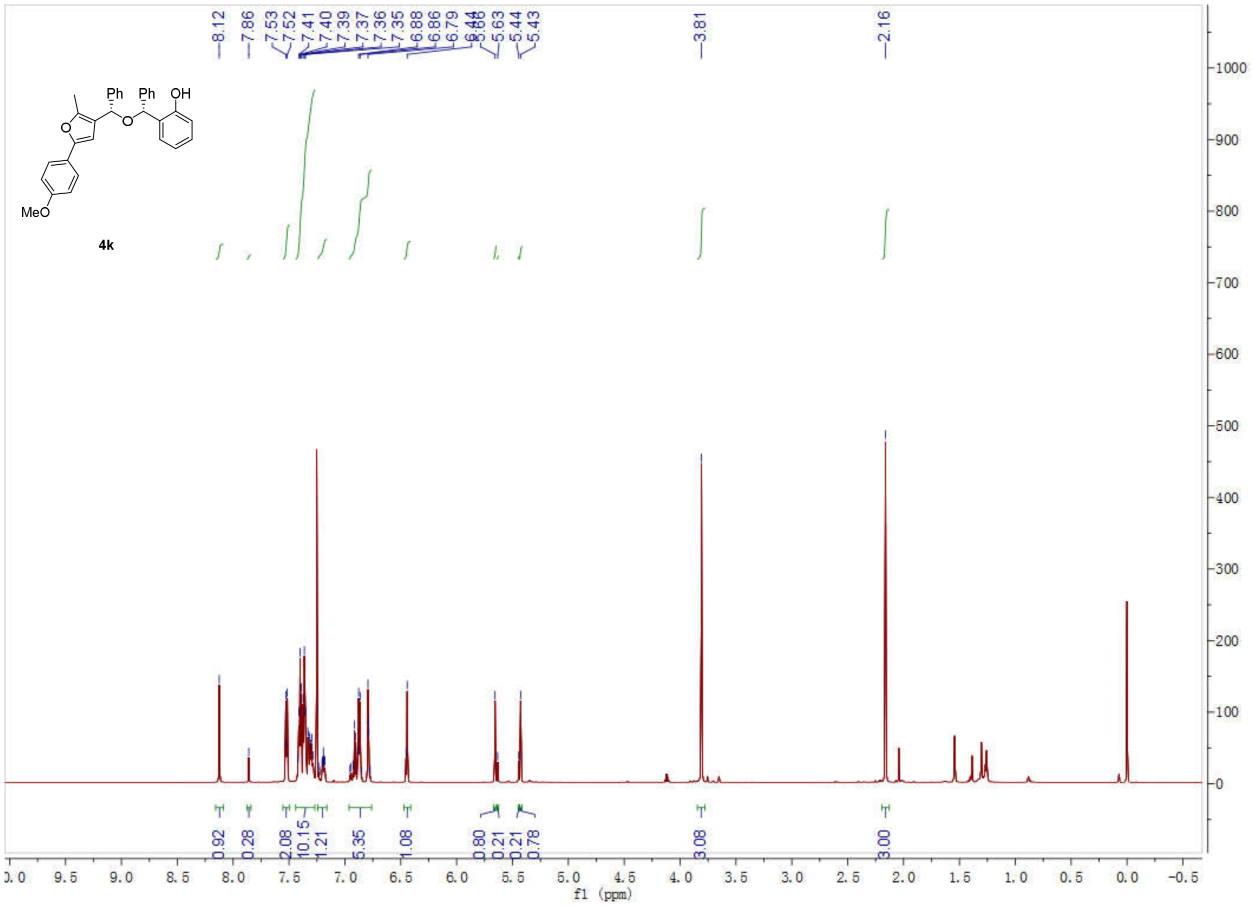
8.12
7.86
7.53
7.52
7.41
7.40
7.39
7.37
7.36
7.35
6.88
6.86
6.79
5.63
5.44
5.43
3.81
2.16
Ph
Ph
OH
O
O
MeO
4k
0.92
0.28
2.08
10.15
1.21
5.35
1.08
0.80
0.21
0.21
0.78
3.08
3.00
f1 (ppm)

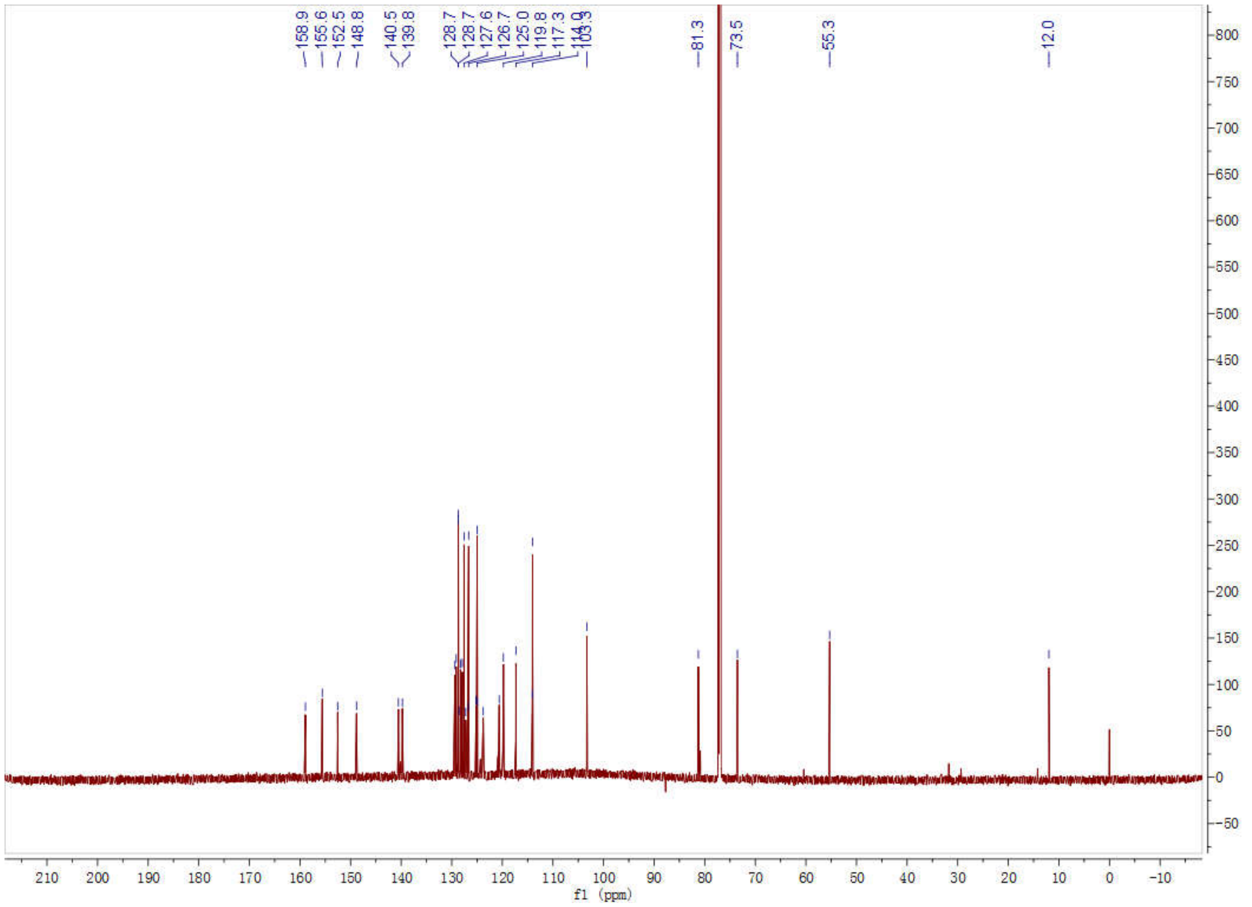
158.9
155.6
152.5
148.8
140.5
139.8
128.7
128.7
127.6
126.7
125.0
119.8
117.3
103.3
81.3
73.5
55.3
12.0
f1 (ppm)

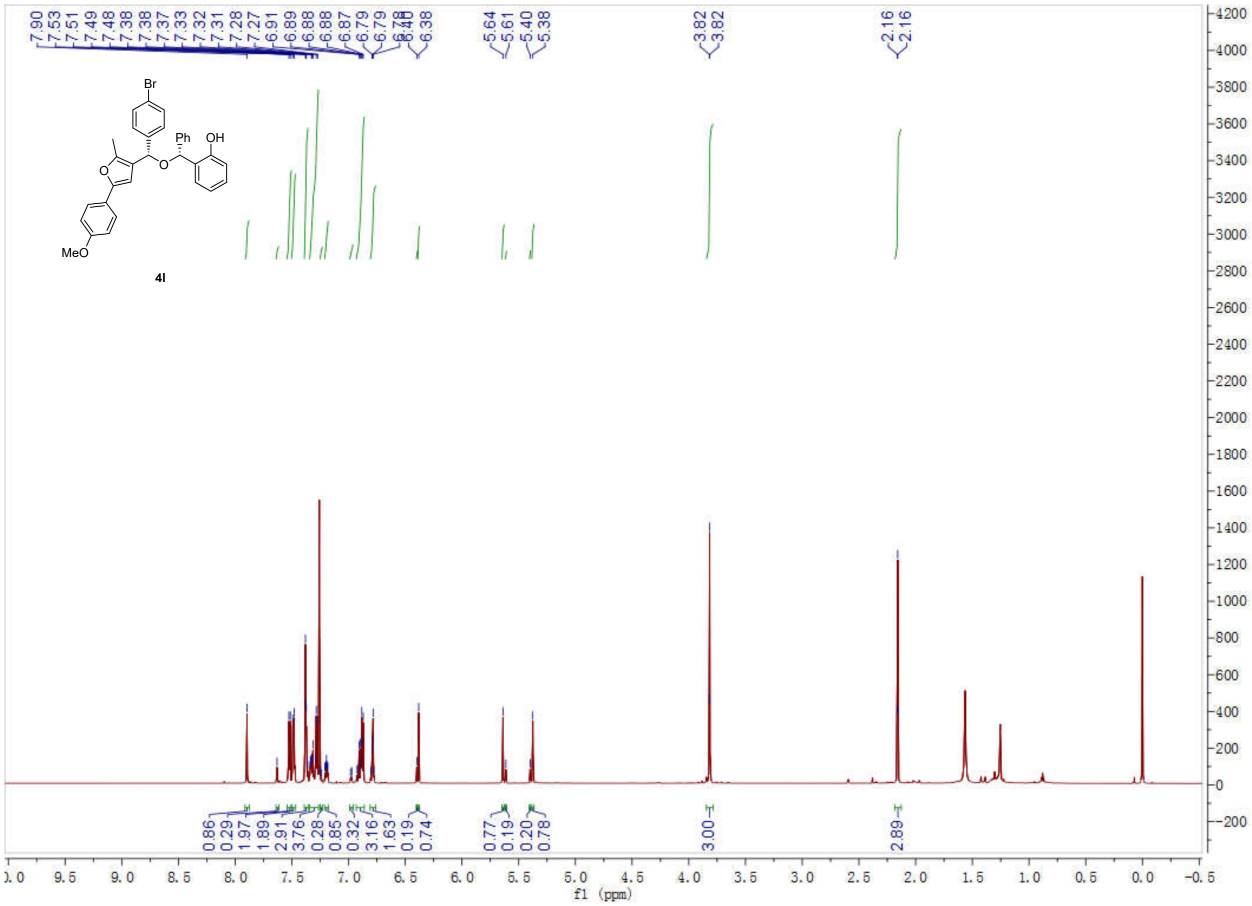
Br
Ph
OH
O
MeO
4l
f1 (ppm)

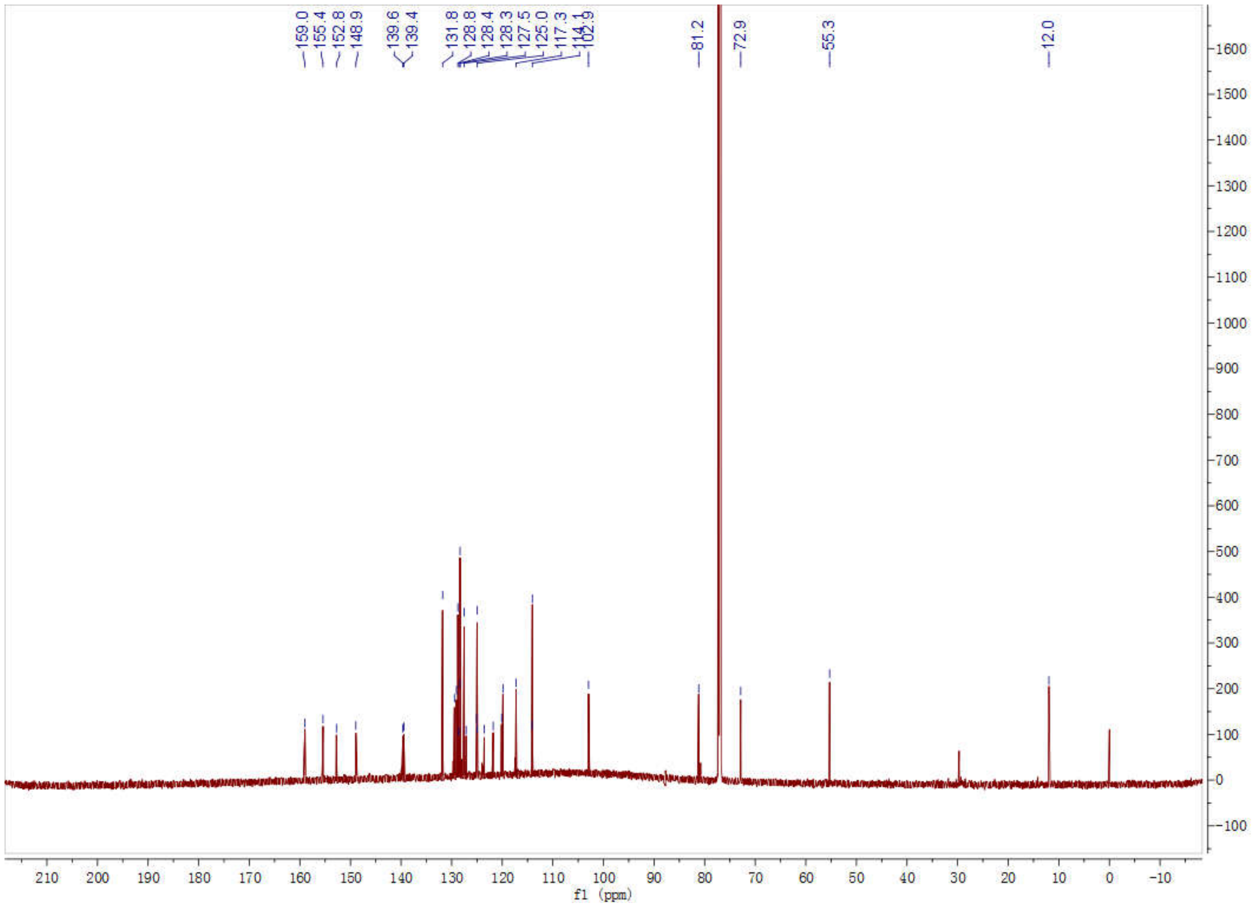
159.0
155.4
152.8
148.9
139.6
139.4
131.8
128.8
128.4
128.3
127.5
125.0
117.3
114.1
102.9
81.2
72.9
55.3
12.0
f1 (ppm)

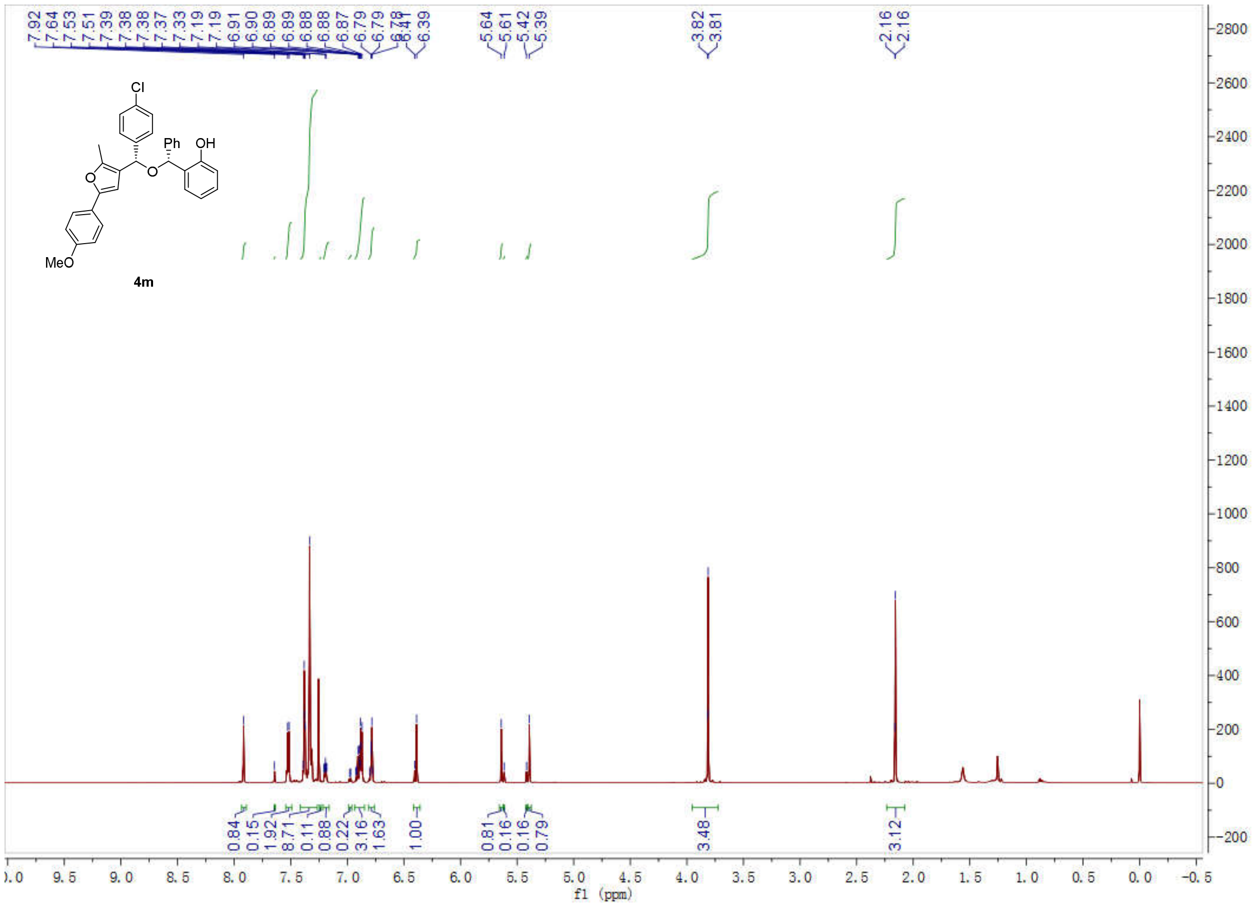

Cl
Ph
OH
O
O
MeO
4m

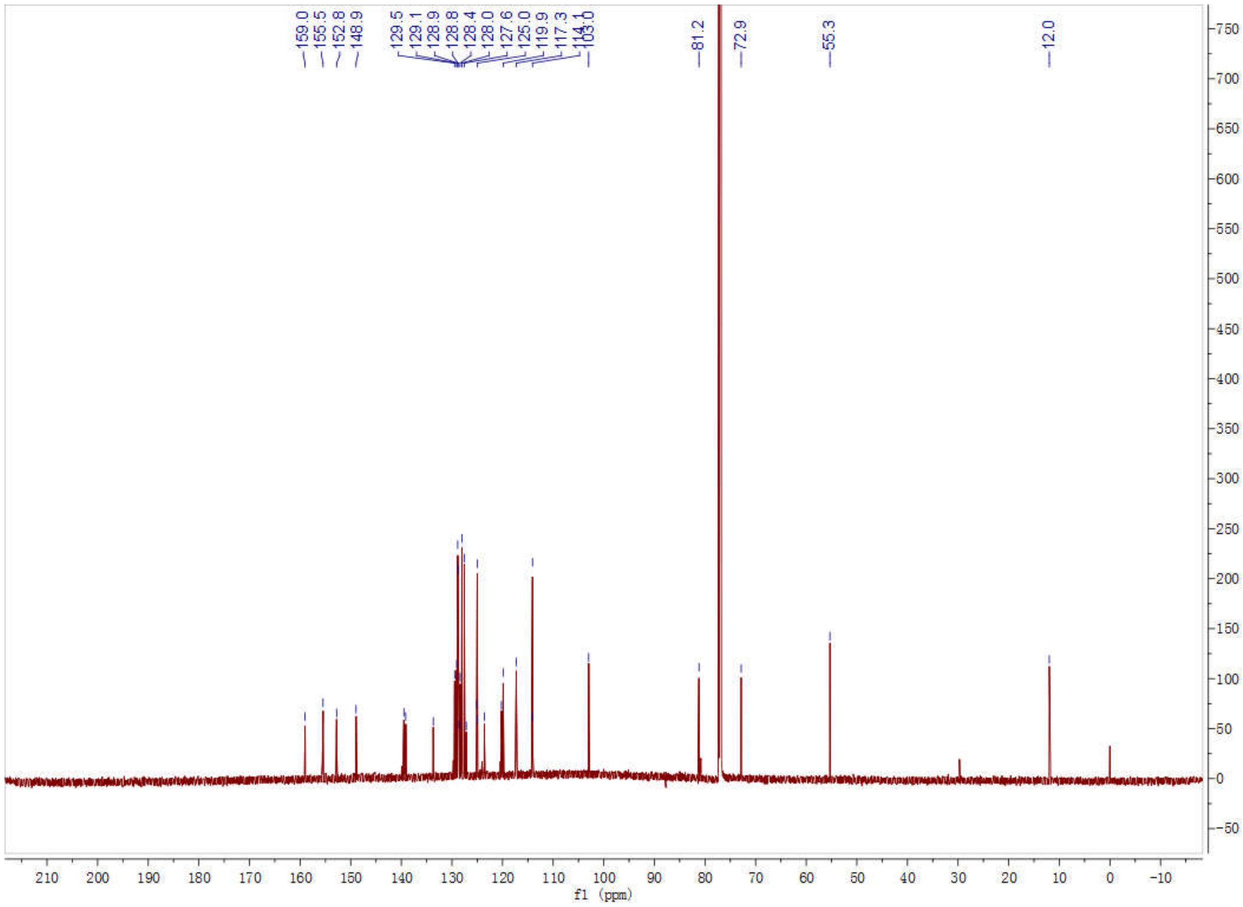

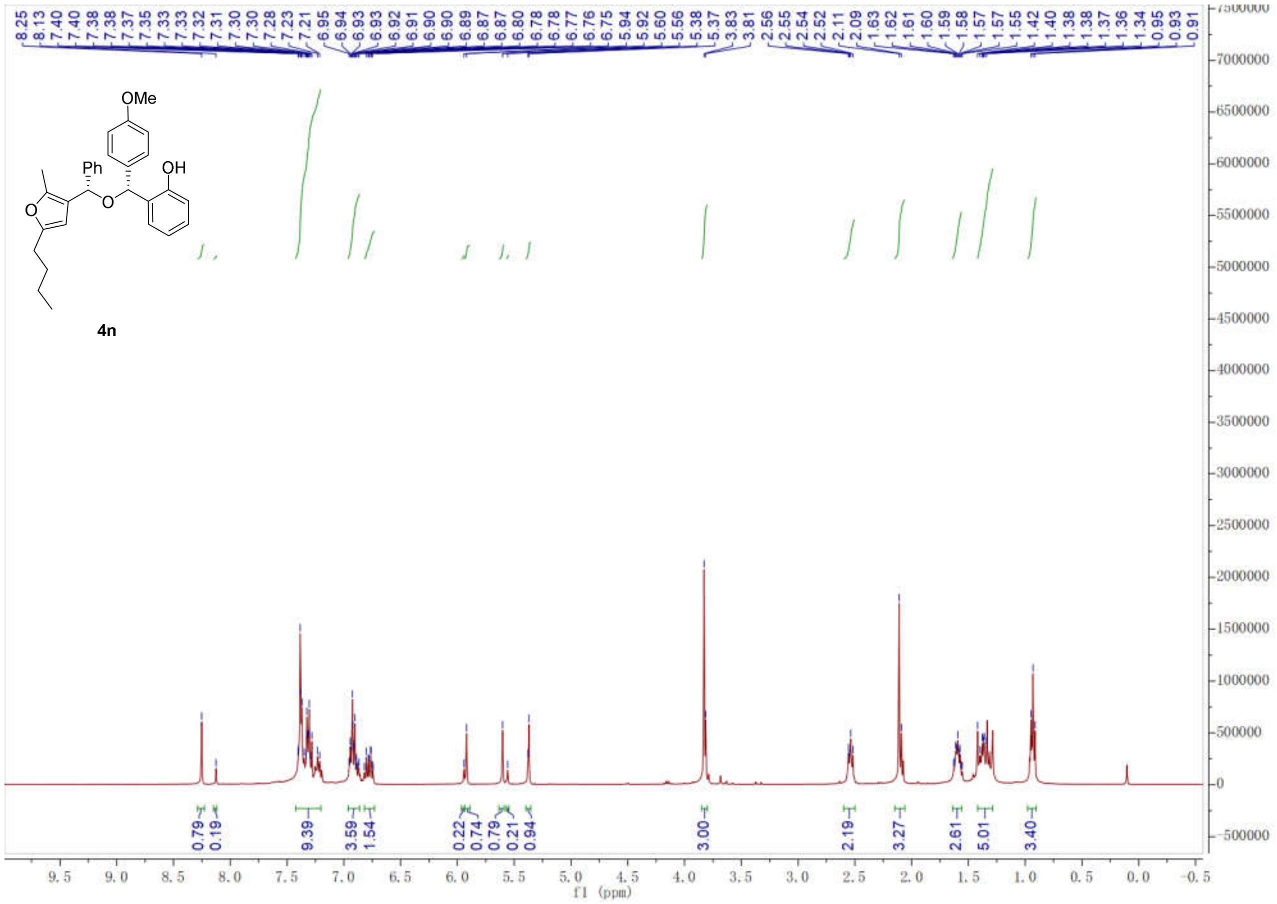
8.25
8.13
7.40
7.40
7.38
7.38
7.37
7.35
7.33
7.33
7.32
7.31
7.30
7.30
7.28
7.23
7.21
6.95
6.94
6.93
6.93
6.92
6.91
6.90
6.90
6.89
6.87
6.87
6.80
6.78
6.77
6.76
6.75
5.94
5.92
5.60
5.56
5.38
5.37
3.83
3.81
2.56
2.55
2.54
2.52
2.11
2.09
1.63
1.62
1.61
1.60
1.59
1.58
1.57
1.55
1.42
1.40
1.38
1.38
1.37
1.36
1.34
0.95
0.93
0.91
OMe
Ph
OH
O
O
4n
0.79
0.19
9.39
3.59
1.54
0.22
0.74
0.79
0.21
0.94
3.00
2.19
3.27
2.61
5.01
3.40
f1 (ppm)

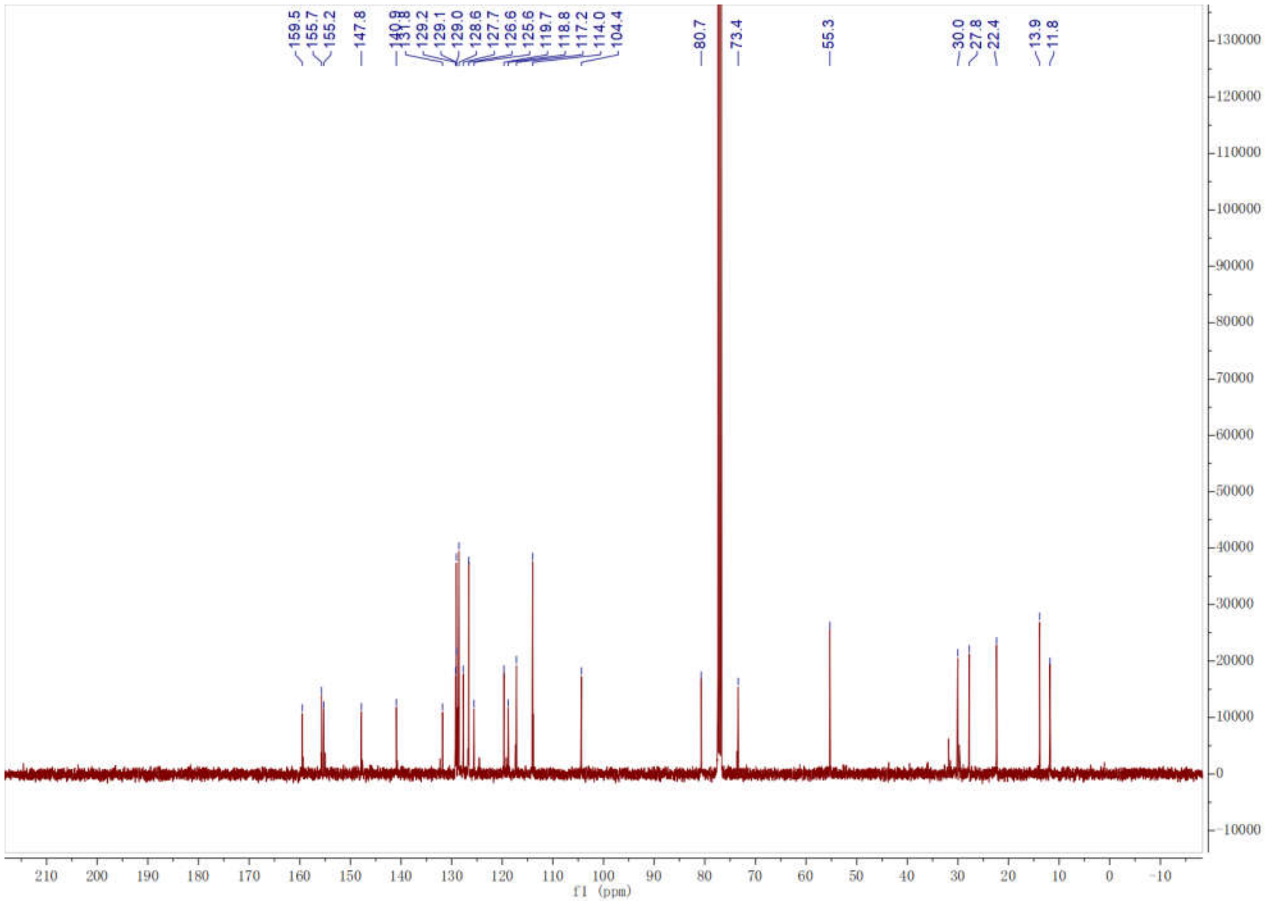
159.5
155.7
155.2
147.8
140.9
131.8
129.2
129.1
129.0
128.6
127.7
126.6
125.6
119.7
118.8
117.2
114.0
104.4
80.7
73.4
55.3
30.0
27.8
22.4
13.9
11.8
f1 (ppm)

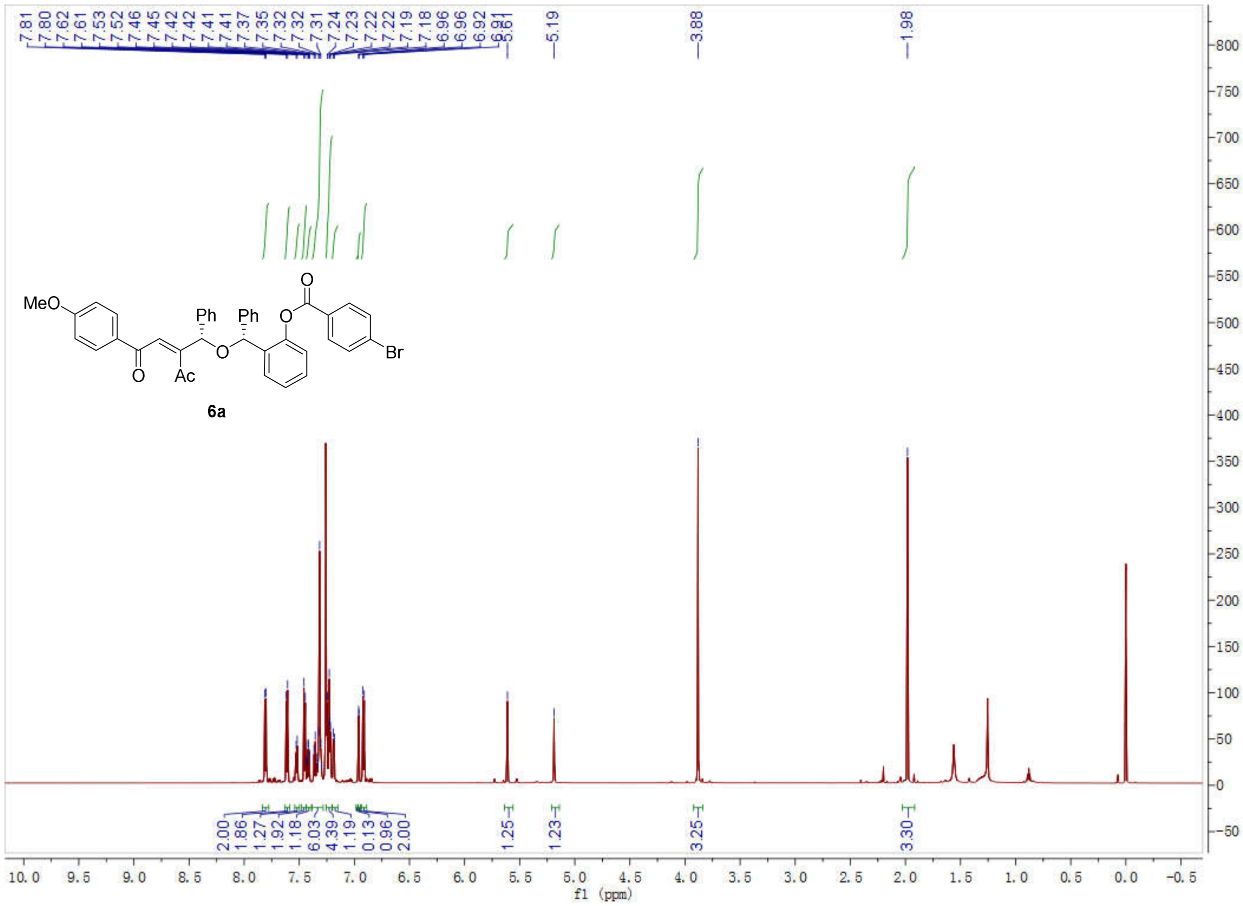

7.81 7.80 7.62 7.61 7.53 7.52 7.46 7.45 7.42 7.42 7.41 7.41 7.37 7.35 7.32 7.32 7.31 7.24 7.23 7.22 7.22 7.19 7.18 6.96 6.96 6.92 6.91 5.61 5.19 3.88 1.98
MeO
Ph
Ph
O
O
O
O
Ac
Br
6a
2.00 1.86 1.27 1.92 1.18 6.03 4.39 1.19 0.13 0.96 2.00 1.25 1.23 3.25 3.30
f1 (ppm)

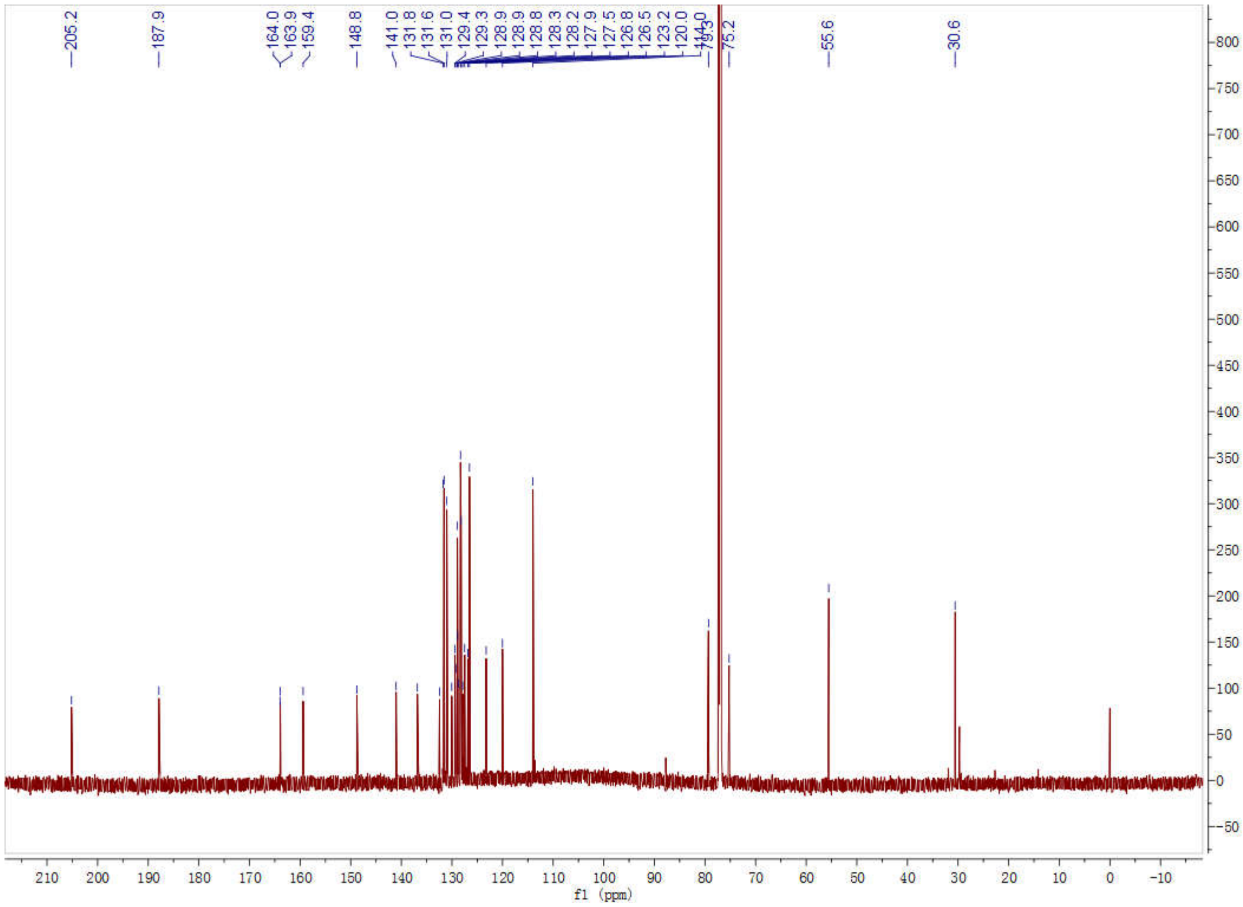

205.2 187.9 164.0 163.9 159.4 148.8 141.0 131.8 131.6 131.0 129.4 129.3 128.9 128.9 128.8 128.3 128.2 127.9 127.5 126.8 126.5 123.2 120.0 114.0 79.3 75.2 55.6 30.6
f1 (ppm)

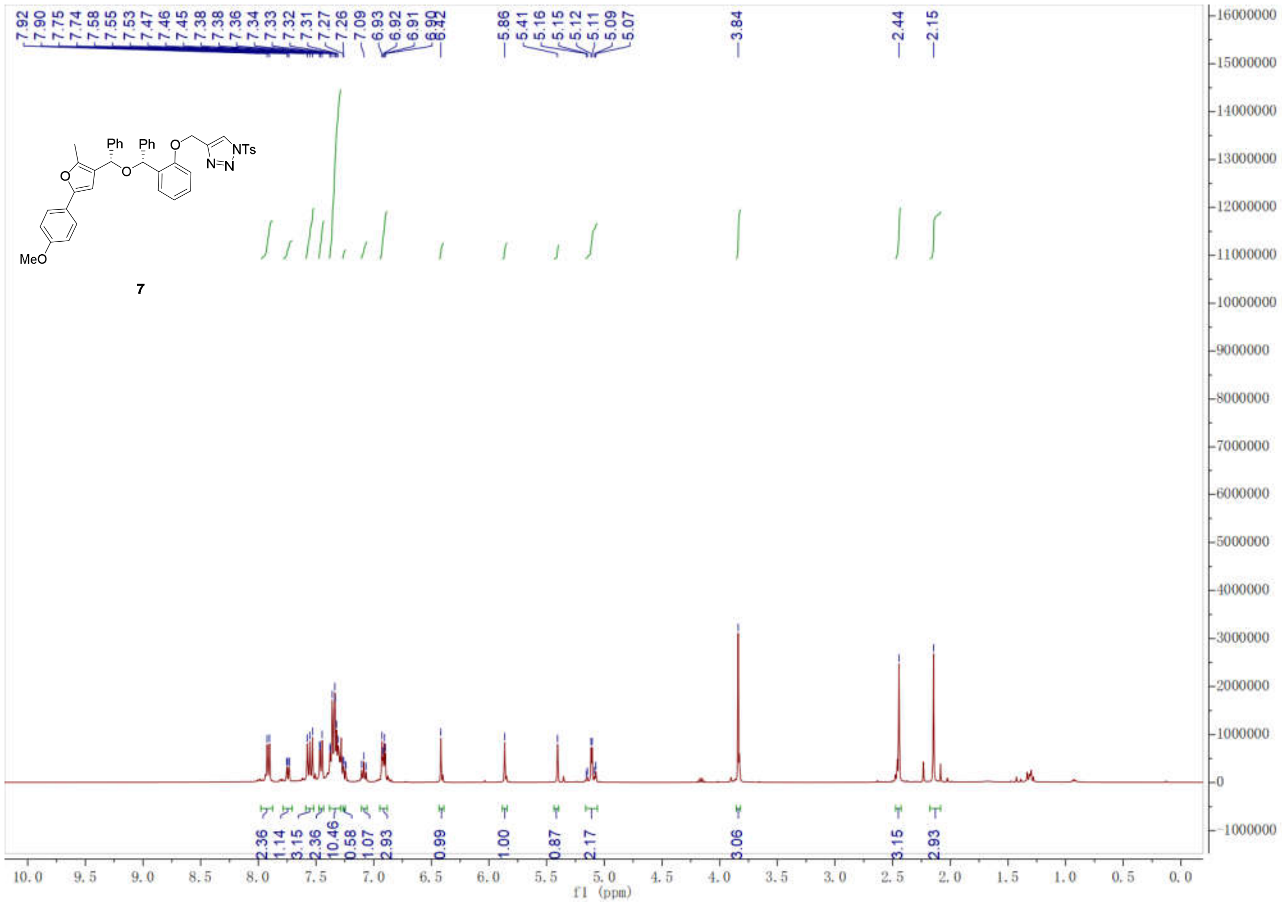
7.92 7.90 7.75 7.74 7.58 7.55 7.53 7.47 7.46 7.45 7.38 7.38 7.36 7.34 7.33 7.32 7.31 7.27 7.26 7.09 6.93 6.92 6.91 6.90 6.42
5.86 5.41 5.16 5.15 5.12 5.11 5.09 5.07
3.84
2.44 2.15
Ph
Ph
NTs
N=N
MeO
7
2.36 1.14 3.15 2.36 10.46 0.58 1.07 2.93 0.99 1.00 0.87 2.17 3.06 3.15 2.93
f1 (ppm)

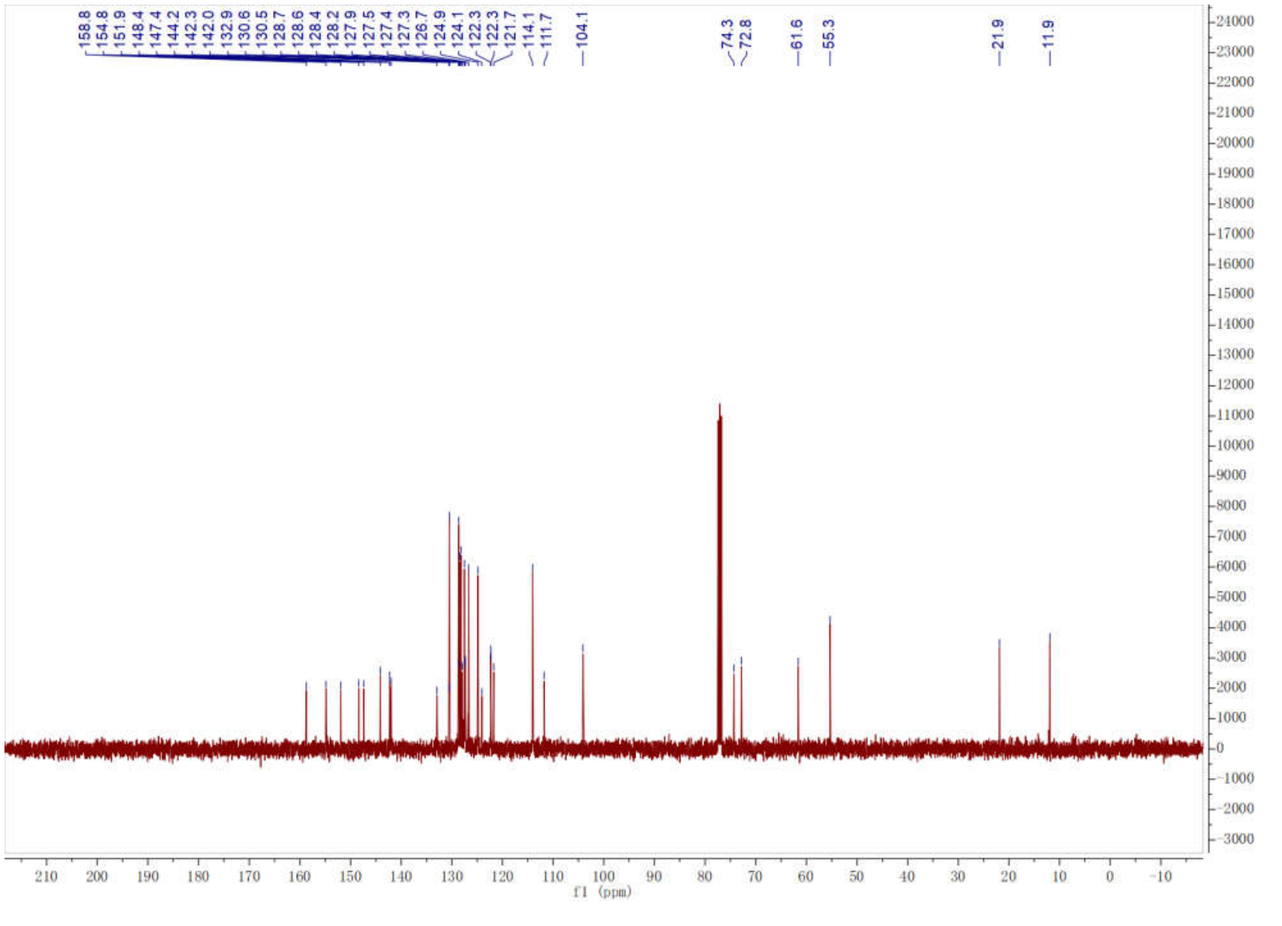
158.8 154.8 151.9 148.4 147.4 144.2 142.3 142.0 132.9 130.6 130.5 128.7 128.6 128.4 128.2 127.9 127.5 127.4 127.3 126.7 124.9 124.1 122.3 122.3 121.7 114.1 111.7 104.1
74.3 72.8 61.6 55.3
21.9 11.9
f1 (ppm)

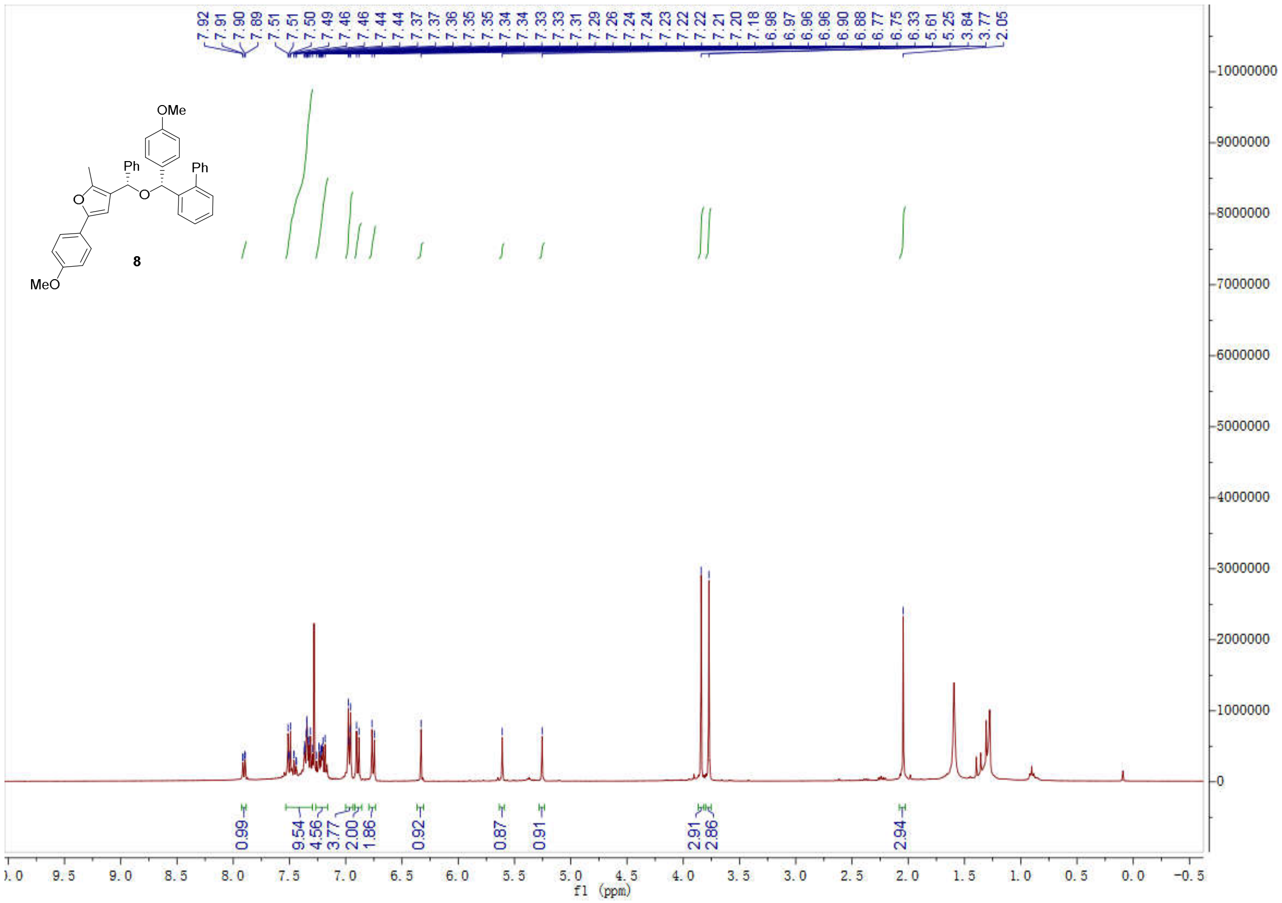

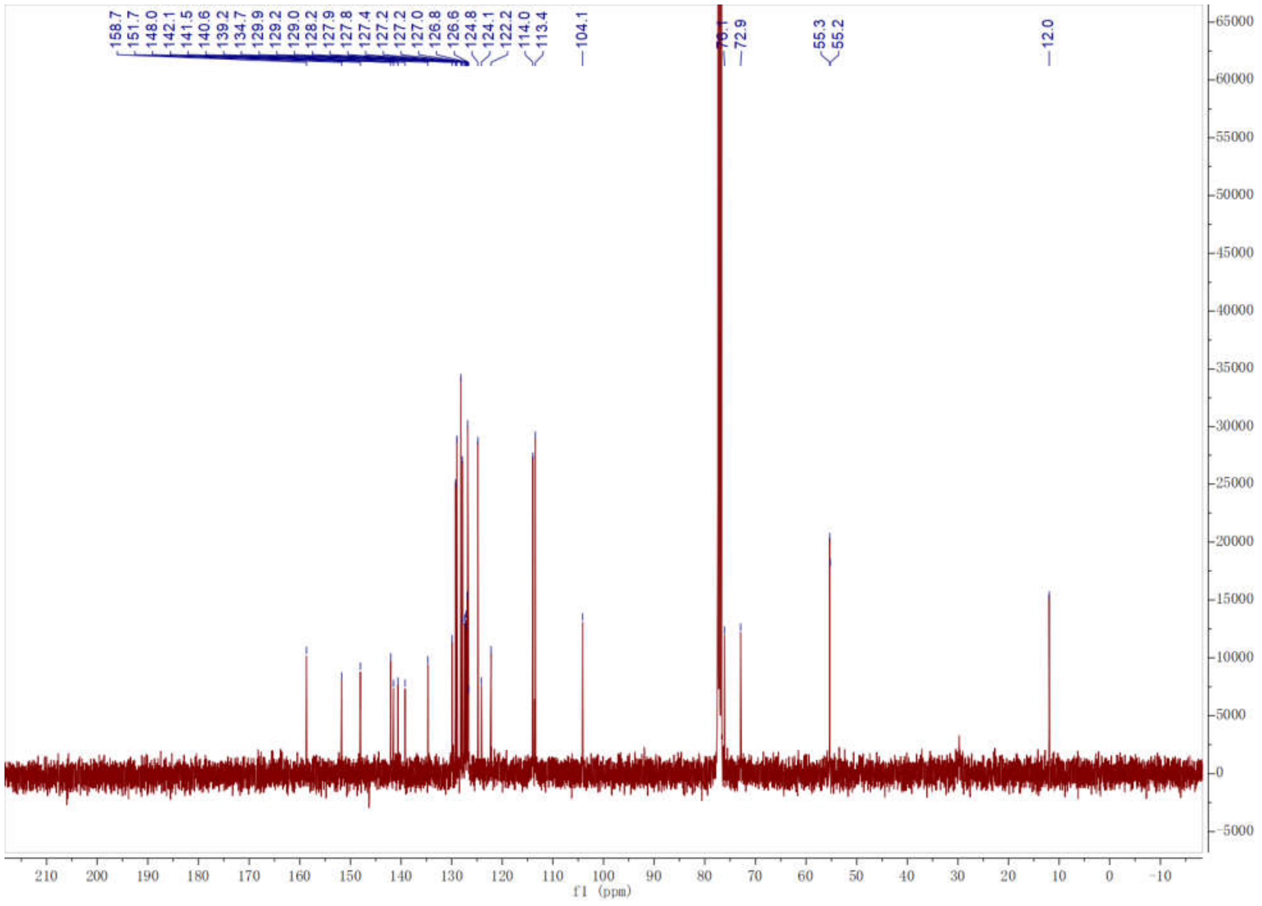

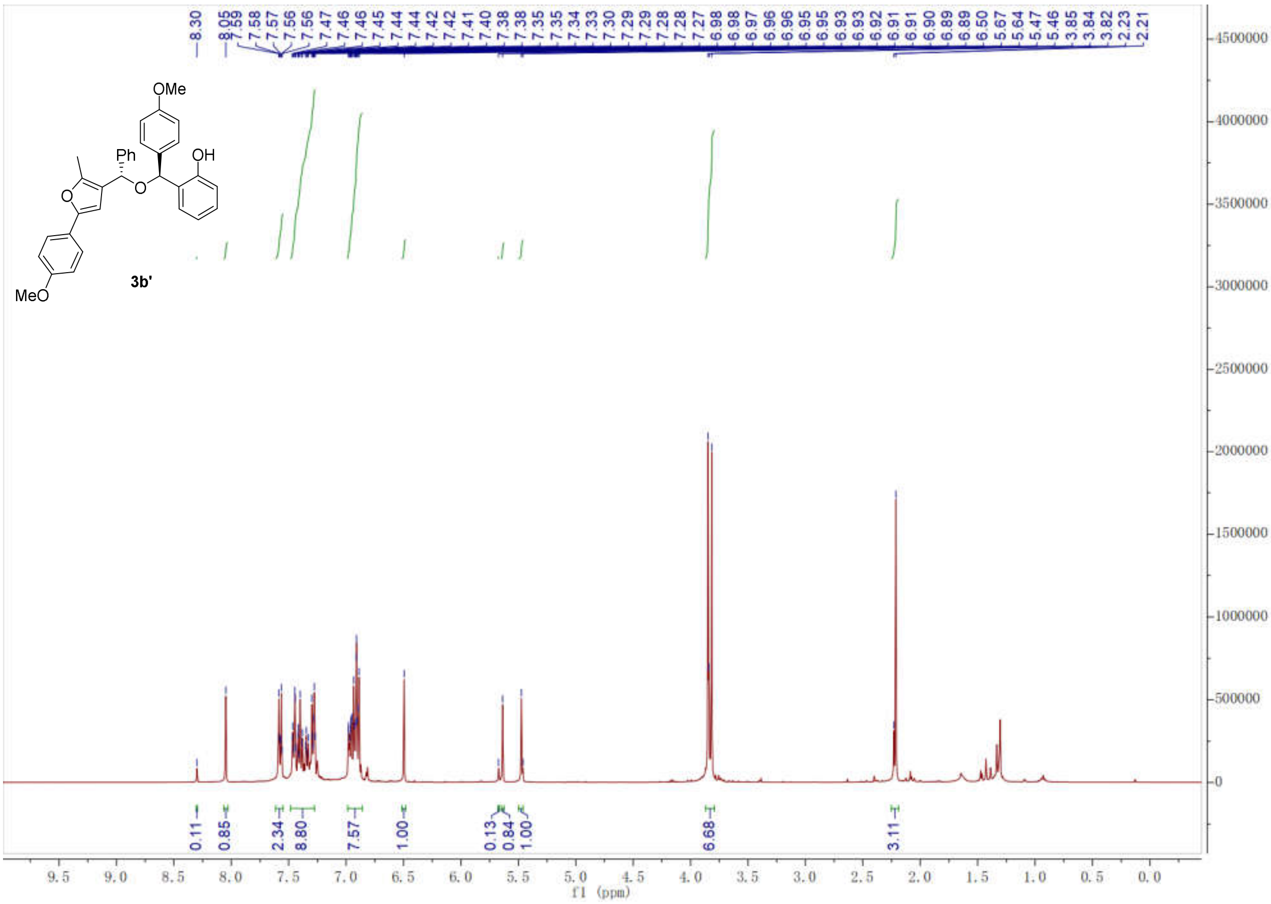

3b'
f1 (ppm)

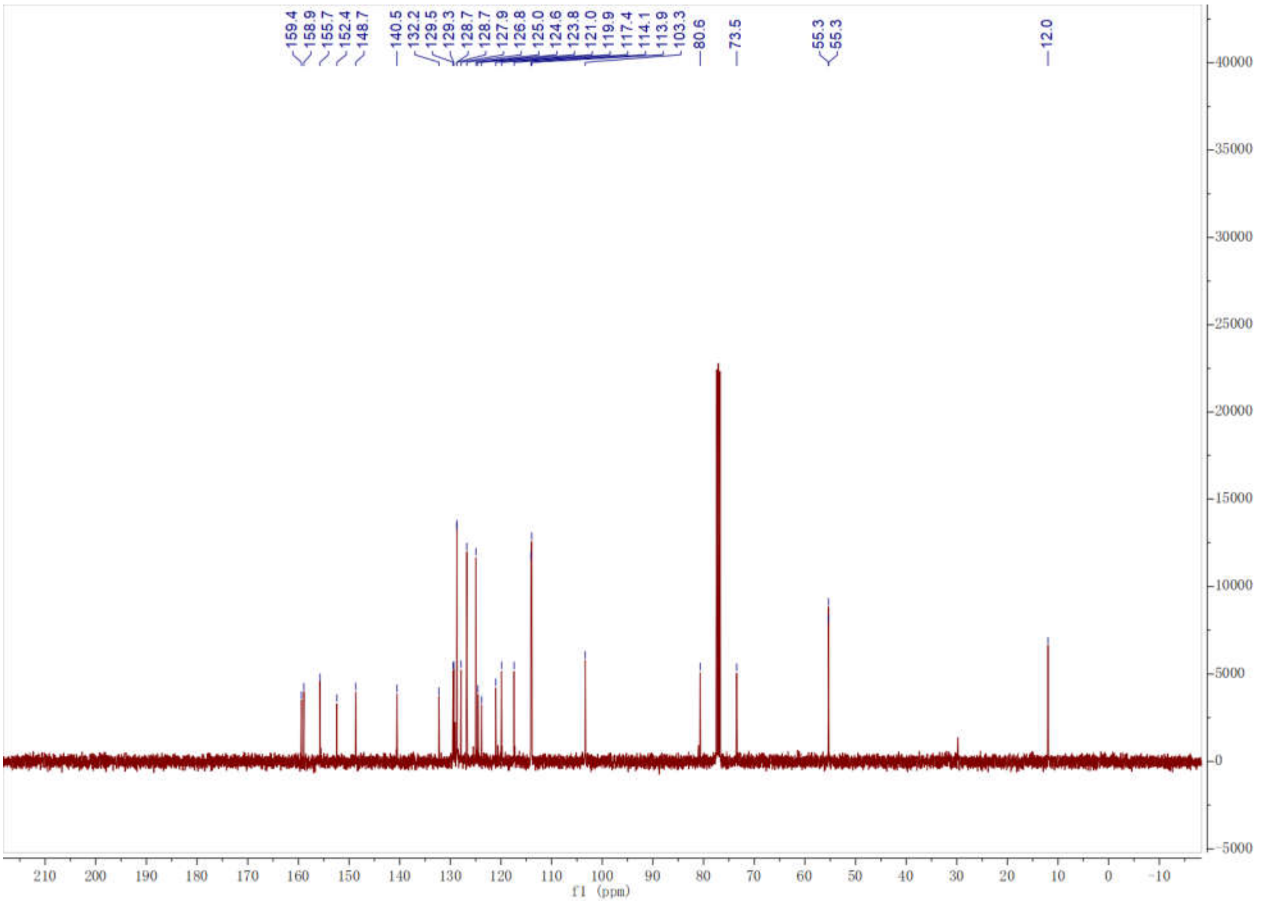

f1 (ppm)

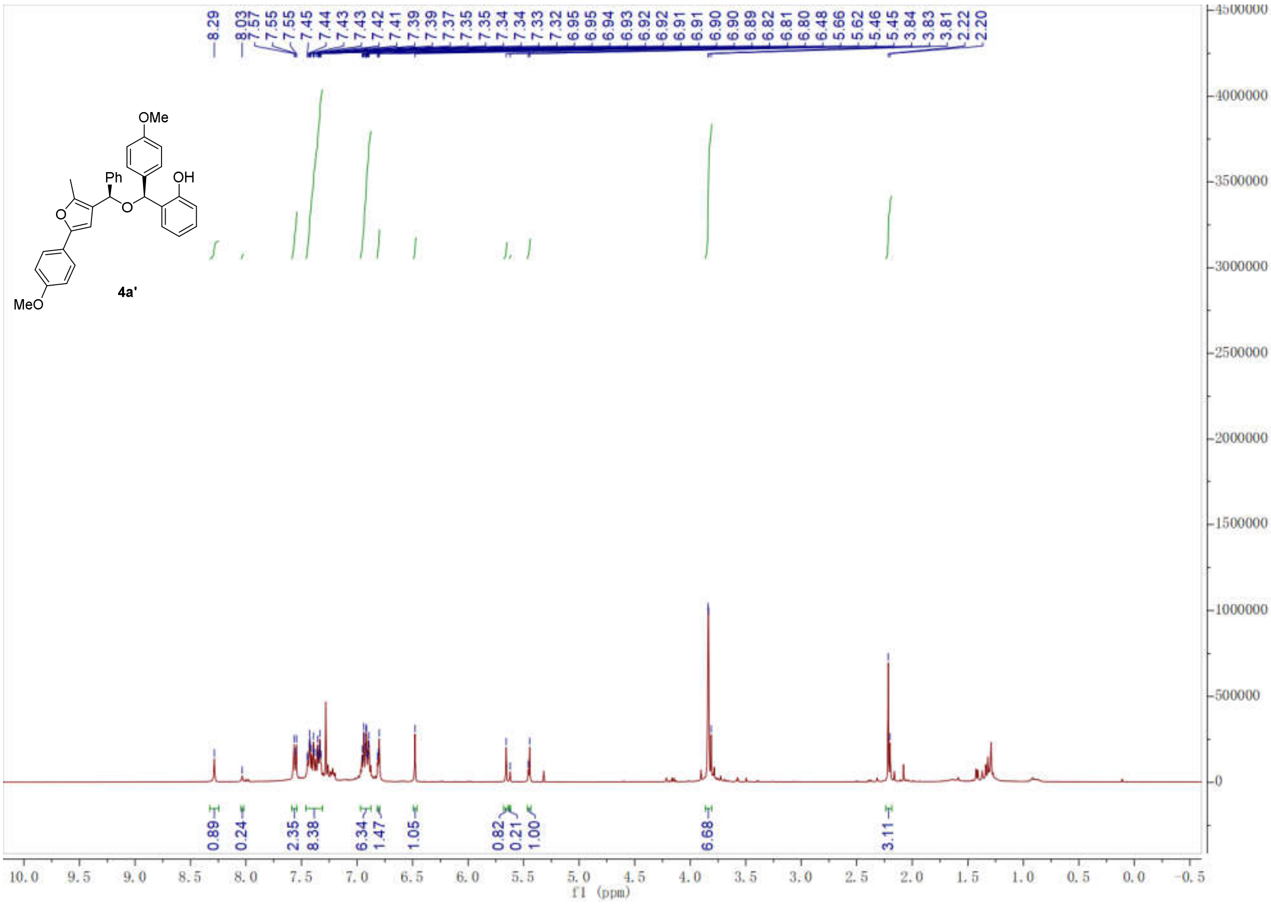

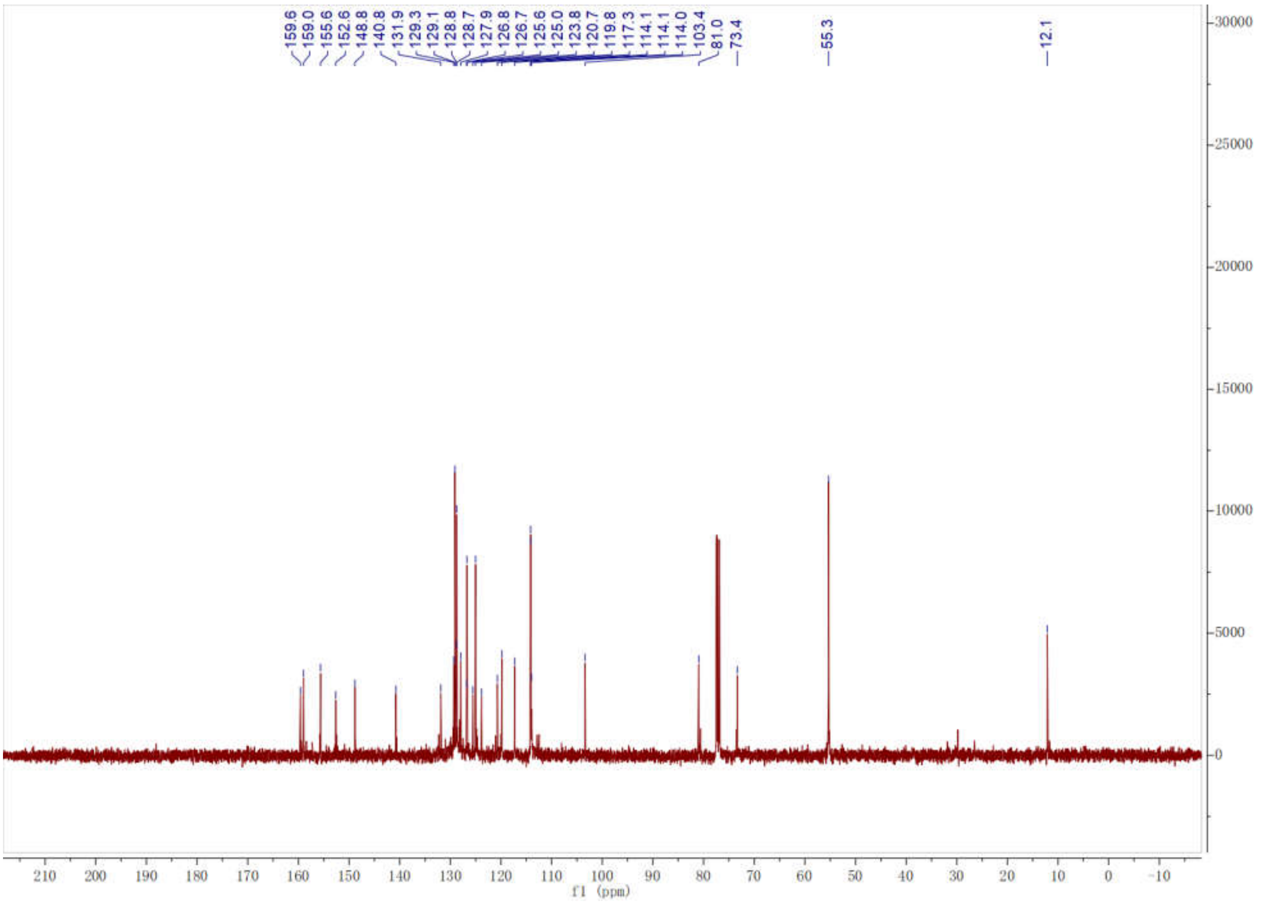

Supplementary Fig. 3 ^{1}H NMR and ^{13}C NMR spectra

9. HPLC spectra

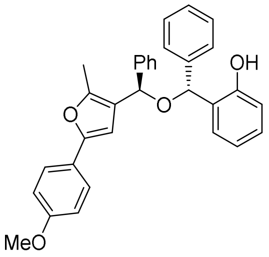

3a

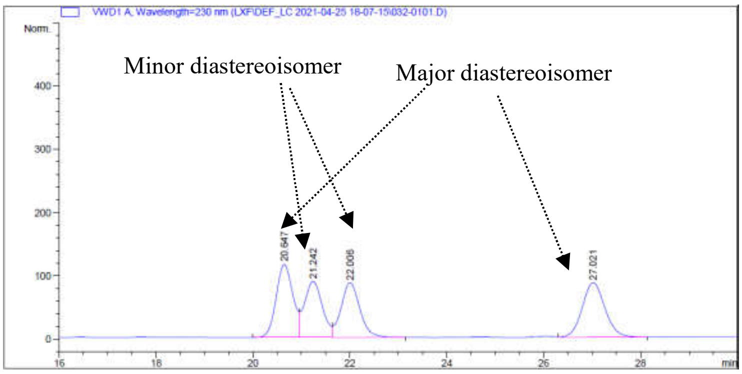

Peak #	RetTime [min]	Type	Width [min]	Area mAU *s	Height [mAU]	Area %
1	20.647	BV	0.3691	2791.61084	115.60535	27.3154
2	21.242	VV	0.3851	2270.55347	88.95551	22.2170
3	22.006	VB	0.4127	2341.90771	86.69201	22.9151
4	27.021	VB	0.5012	2815.84180	86.38092	27.5525

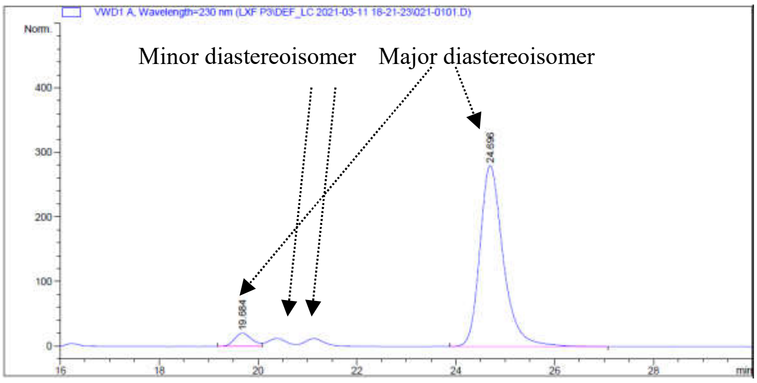

Peak #	RetTime [min]	Type	Width [min]	Area [mAU*s]	Height [mAU]	Area %
1	19.684	BV	0.3729	512.89008	20.74788	5.2530
2	24.696	BB	0.4956	9250.88672	280.39883	94.7470

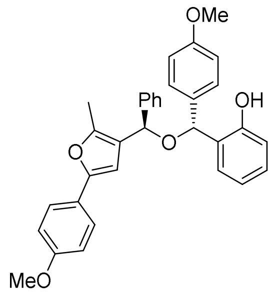

3b

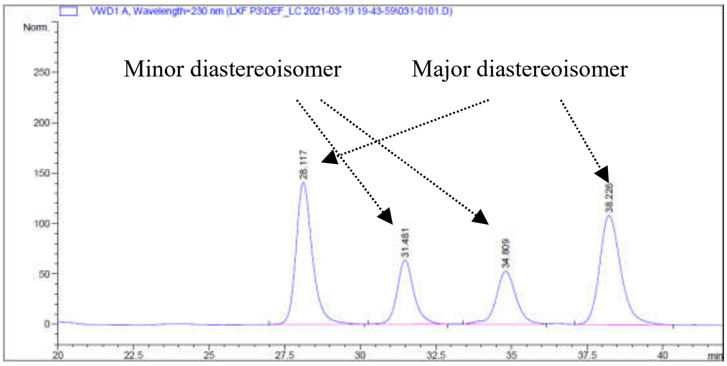

Peak #	RetTime [min]	Type	Width [min]	Area [mAU*s]	Height [mAU]	Area %
1	28.117	BB	0.5673	5314.15625	141.17642	34.2086
2	31.481	BB	0.5845	2477.57007	63.74636	15.9488
3	34.809	BB	0.7018	2439.03223	52.81876	15.7007
4	38.226	VB	0.7475	5303.78223	108.57338	34.1419

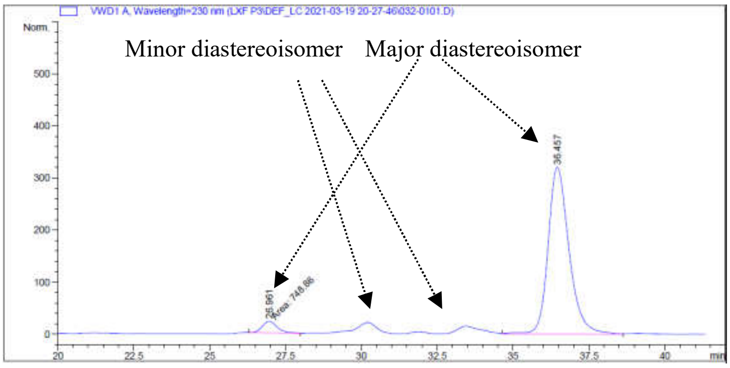

Peak #	RetTime [min]	Type	Width [min]	Area [mAU*s]	Height [mAU]	Area %
1	26.961	MM	0.5667	748.86005	22.02340	4.6203
2	36.457	VB	0.7311	1.54593e4	320.80374	95.3797

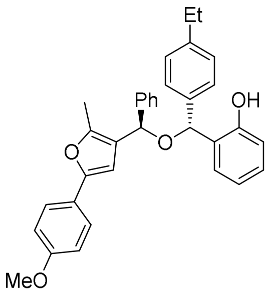

3c

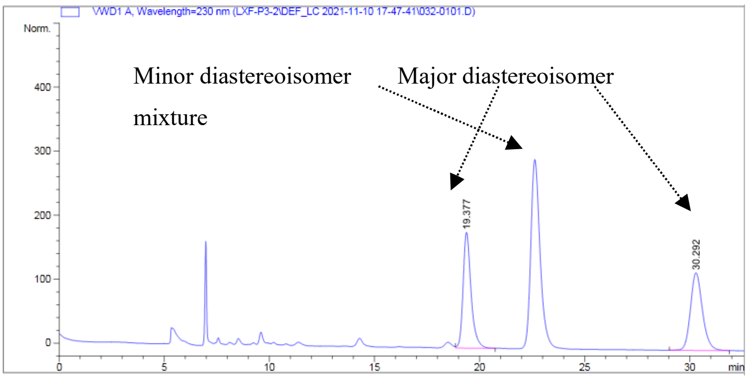

Peak #	RetTime [min]	Type	Width [min]	Area [mAU*s]	Height [mAU]	Area %
1	19.377	VB	0.3978	4760.94287	180.65874	49.7418
2	30.292	BB	0.6025	4810.37354	121.32053	50.2582

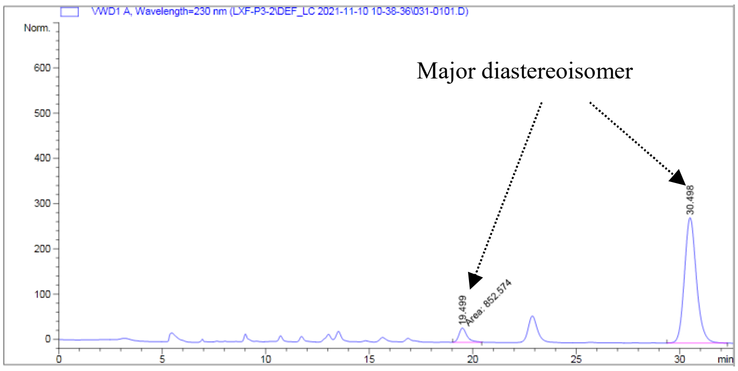

Peak #	RetTime [min]	Type	Width [min]	Area [mAU*s]	Height [mAU]	Area %
1	19.499	MM	0.4520	852.57391	31.43792	7.0851
2	30.498	BB	0.6178	1.11808e4	277.19788	92.9149

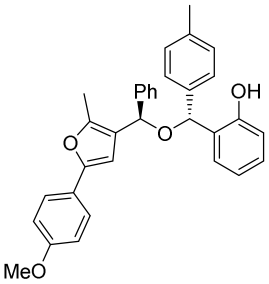

3d

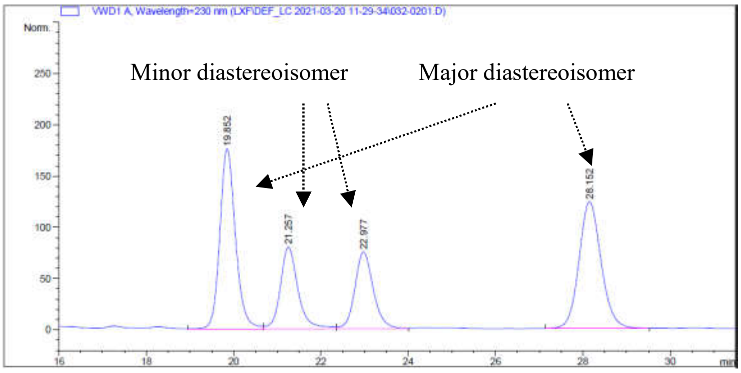

Peak #	RetTime [min]	Type	Width [min]	Area mAU *s	Height [mAU]	Area %
1	19.852	VV	0.3804	4368.26709	175.65125	33.1905
2	21.257	VV	0.4258	2237.56421	79.87933	17.0012
3	22.977	VV	0.4471	2201.43433	75.04173	16.7267
4	28.152	BB	0.5418	4353.93408	123.67632	33.0816

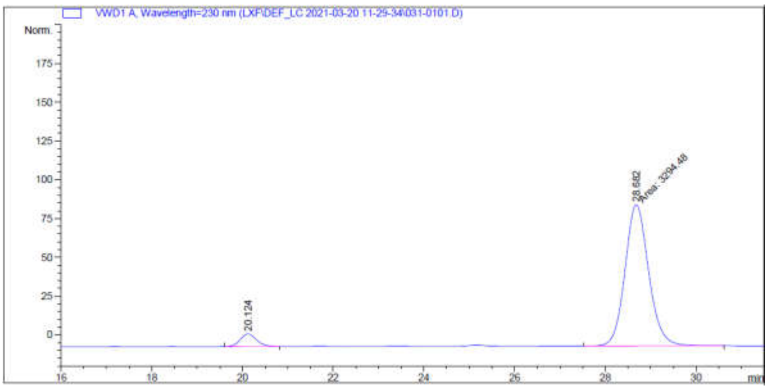

Peak #	RetTime [min]	Type	Width [min]	Area mAU *s	Height [mAU]	Area %
1	20.124	BB	0.3810	205.50191	8.16535	5.8715
2	28.682	MM	0.6031	3294.48242	91.04227	94.1285

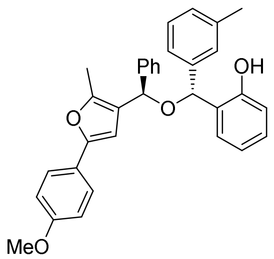

3e

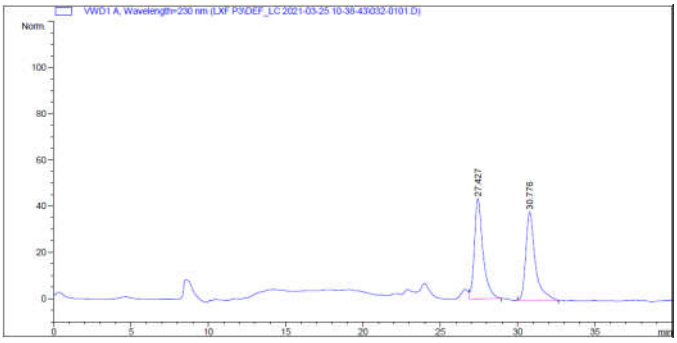

Peak #	RetTime [min]	Type	Width [min]	Area [mAU*s]	Height [mAU]	Area %
1	27.427	VB	0.5700	1672.45508	43.30463	50.6988
2	30.776	BB	0.6202	1626.34888	38.21817	49.3012

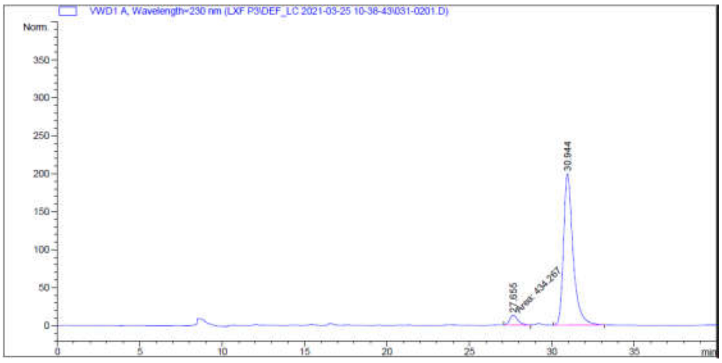

Peak #	RetTime [min]	Type	Width [min]	Area [mAU*s]	Height [mAU]	Area %
1	27.655	MM	0.5596	434.26694	12.93461	5.1310
2	30.944	BB	0.5933	8029.37158	199.52449	94.8690

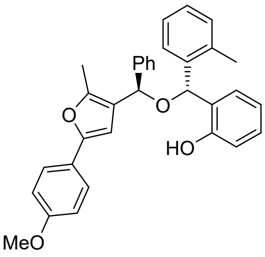

3f

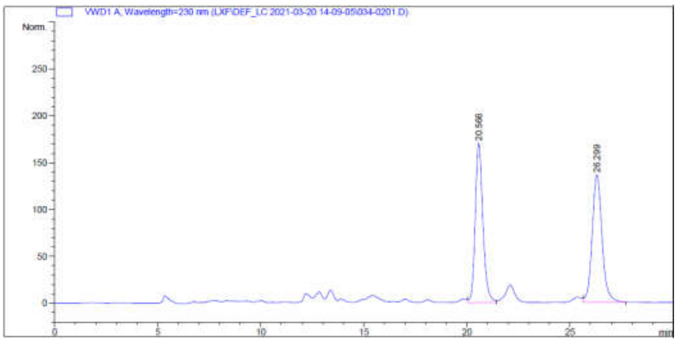

Peak #	RetTime [min]	Type	Width [min]	Area mAU *s	Height [mAU]	Area %
1	20.566	VV	0.3959	4417.11768	170.21945	49.3612
2	26.299	VB	0.5049	4531.44238	136.10970	50.6388

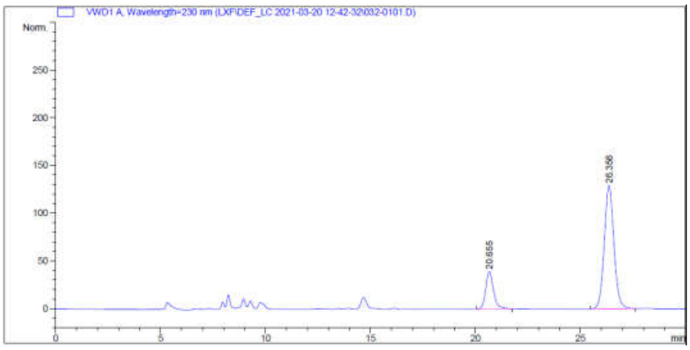

Peak #	RetTime [min]	Type	Width [min]	Area mAU *s	Height [mAU]	Area %
1	20.655	BB	0.3929	1017.13617	39.20855	19.7707
2	26.356	BB	0.4954	4127.53223	129.07196	80.2293

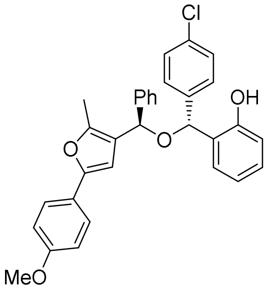

3g

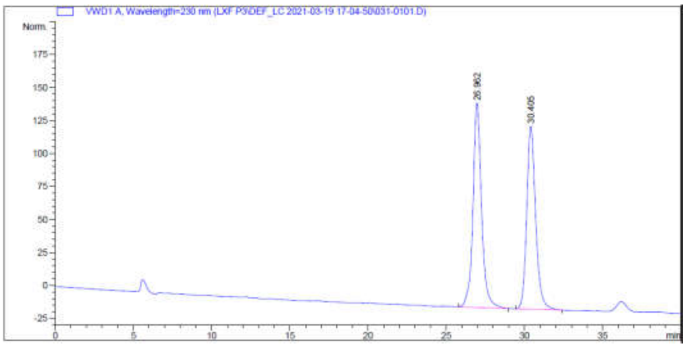

Peak #	RetTime [min]	Type	Width [min]	Area [mAU*s]	Height [mAU]	Area %
1	26.962	BB	0.5703	5909.92578	154.92708	51.7767
2	30.405	BB	0.6043	5504.32861	138.69279	48.2233

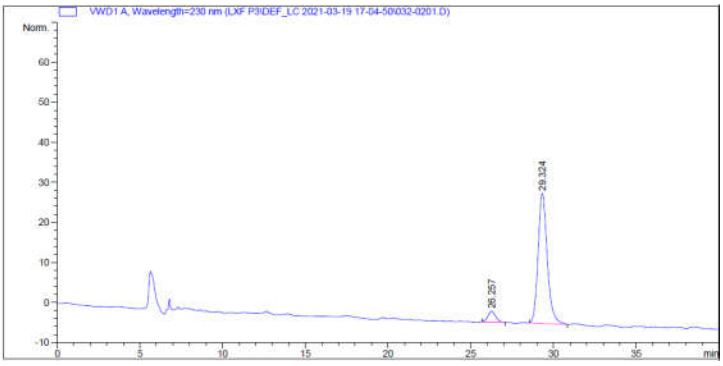

Peak #	RetTime [min]	Type	Width [min]	Area [mAU*s]	Height [mAU]	Area %
1	26.257	BB	0.4668	85.92699	2.69349	6.4206
2	29.324	BB	0.5863	1252.37842	32.51232	93.5794

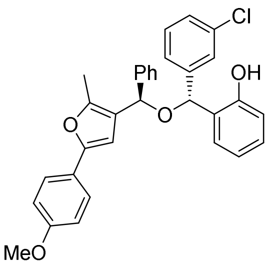

3h

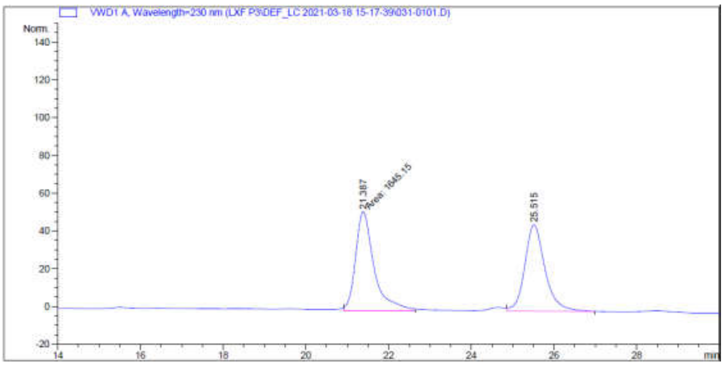

Peak #	RetTime [min]	Type	Width [min]	Area [mAU*s]	Height [mAU]	Area %
1	21.387	MM	0.5209	1645.14661	52.63701	51.4642
2	25.515	VB	0.5100	1551.53394	45.65846	48.5358

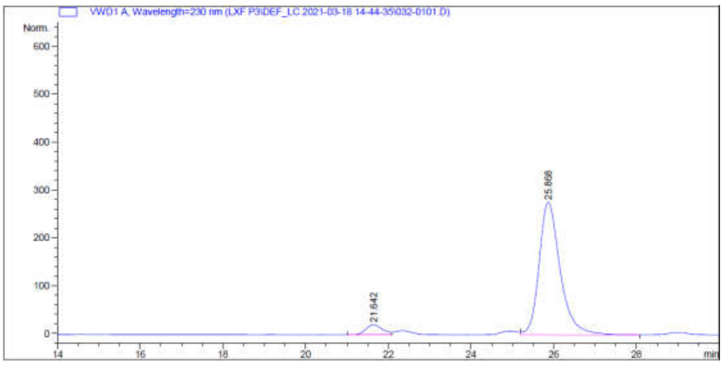

Peak #	RetTime [min]	Type	Width [min]	Area [mAU*s]	Height [mAU]	Area %
1	21.642	BV	0.4299	603.42798	21.46377	5.7554
2	25.868	VB	0.5428	9881.13184	277.04797	94.2446

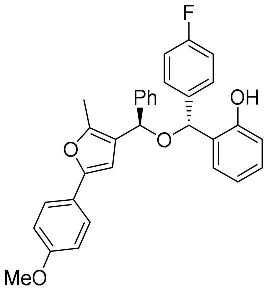

3i

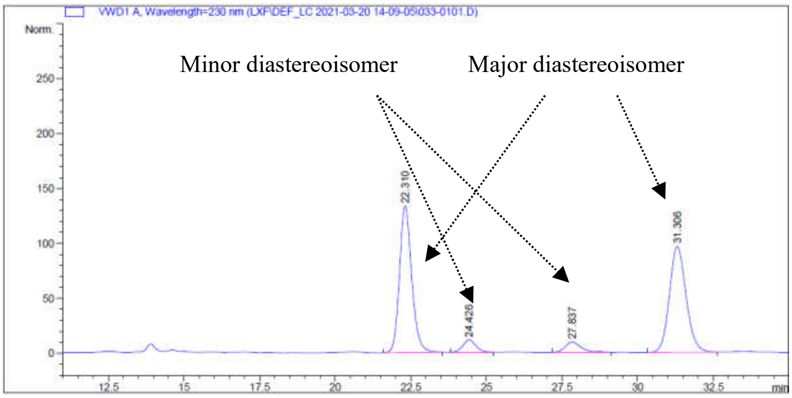

Peak #	RetTime [min]	Type	Width [min]	Area mAU *s	Height [mAU]	Area %
1	22.310	BB	0.4292	3740.15259	133.32584	46.0430
2	24.426	BB	0.4556	332.91116	11.16551	4.0983
3	27.837	BB	0.5548	350.19763	9.38287	4.3111
4	31.306	BB	0.5890	3699.91577	96.40179	45.5476

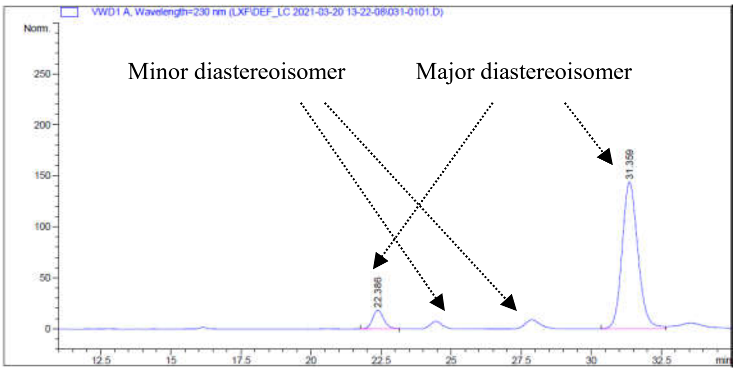

Peak #	RetTime [min]	Type	Width [min]	Area mAU *s	Height [mAU]	Area %
1	22.386	BB	0.4216	501.07230	18.28585	8.1654
2	31.359	BB	0.6059	5635.45947	143.75452	91.8346

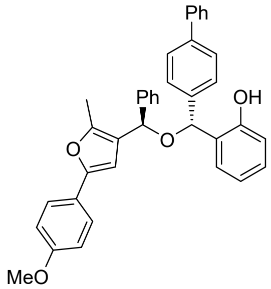

3j

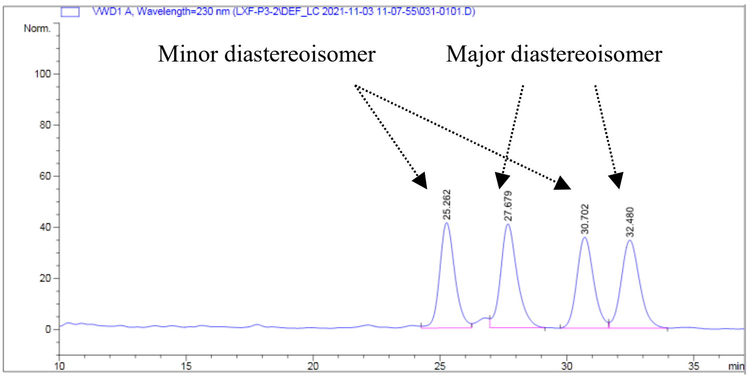

Peak #	RetTime [min]	Type	Width [min]	Area [mAU*s]	Height [mAU]	Area %
1	25.262	VV	0.6325	1703.56641	41.20281	24.7358
2	27.679	VB	0.6775	1835.28198	40.60452	26.6483
3	30.702	BV	0.7012	1626.03369	35.55472	23.6100
4	32.480	VB	0.7652	1722.16833	34.37715	25.0059

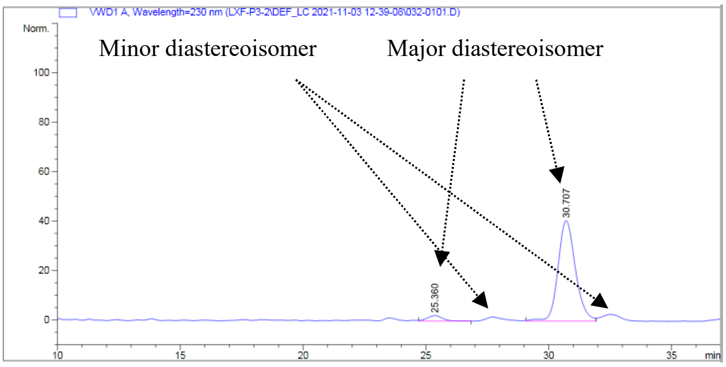

Peak #	RetTime [min]	Type	Width [min]	Area [mAU*s]	Height [mAU]	Area %
1	25.360	BB	0.5640	90.92668	2.17279	4.3268
2	30.707	BV	0.7578	2010.52820	40.65215	95.6732

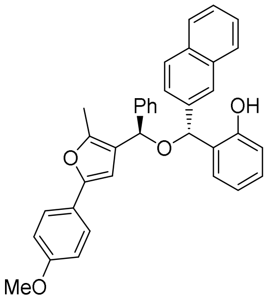

3k

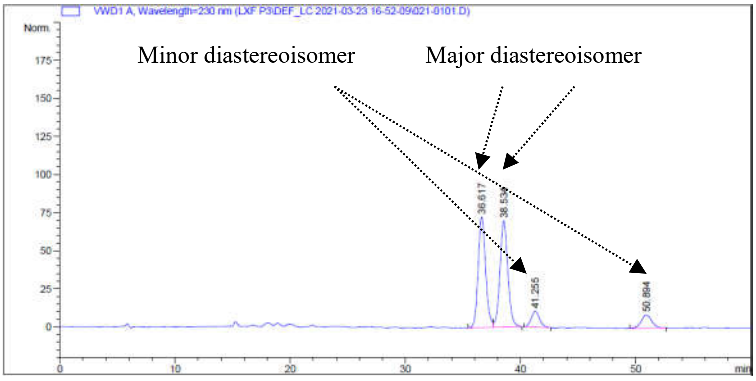

Peak #	RetTime [min]	Type	Width [min]	Area [mAU*s]	Height [mAU]	Area %
1	36.617	BV	0.7100	3278.62378	72.63994	42.7057
2	38.534	VB	0.7114	3315.06445	69.79155	43.1804
3	41.255	BB	0.7511	525.23096	10.47210	6.8414
4	50.894	BB	0.8586	558.32422	8.67523	7.2725

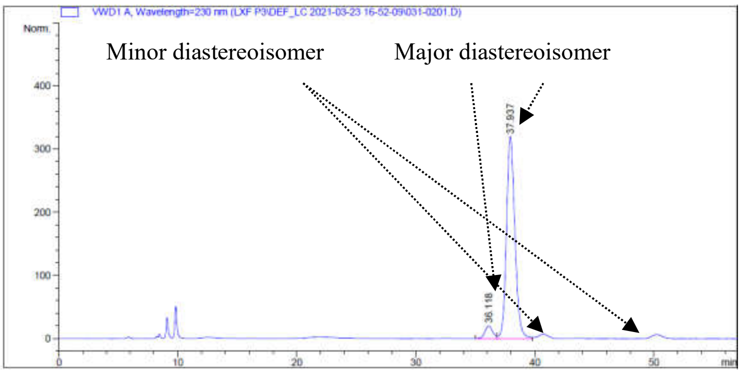

Peak #	RetTime [min]	Type	Width [min]	Area [mAU*s]	Height [mAU]	Area %
1	36.118	BV	0.6752	903.90649	20.02098	5.4513
2	37.937	VB	0.7215	1.56777e4	320.02008	94.5487

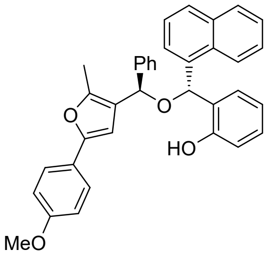

3l

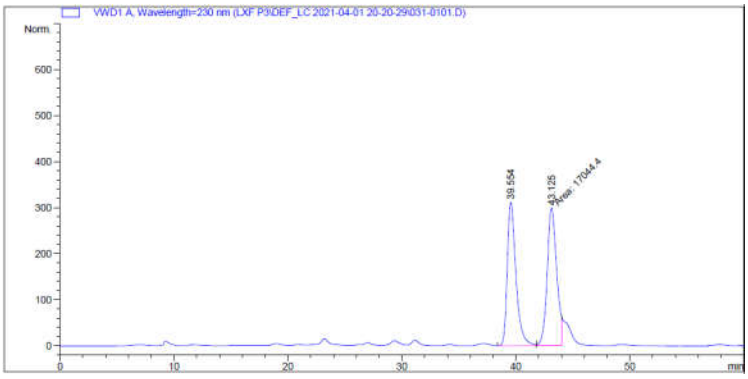

Peak #	RetTime [min]	Type	Width [min]	Area [mAU*s]	Height [mAU]	Area %
1	39.554	VB	0.7986	1.64664e4	311.54758	49.1376
2	43.125	MM	0.9516	1.70444e4	298.51614	50.8624

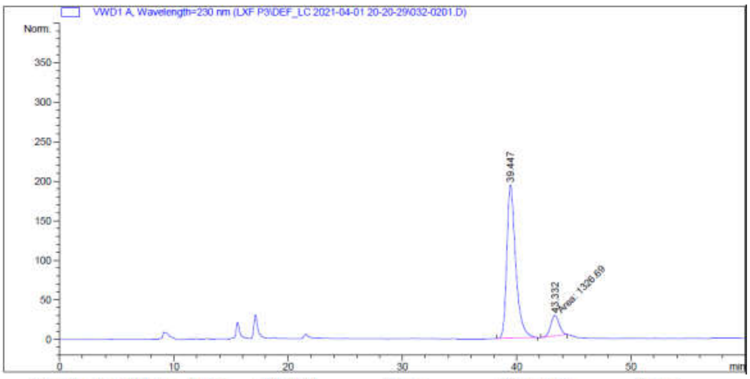

Peak #	RetTime [min]	Type	Width [min]	Area [mAU*s]	Height [mAU]	Area %
1	39.447	BB	0.7715	1.04492e4	193.85898	88.7338
2	43.332	MM	0.8524	1326.69055	25.93990	11.2662

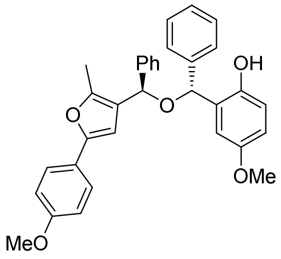

3m

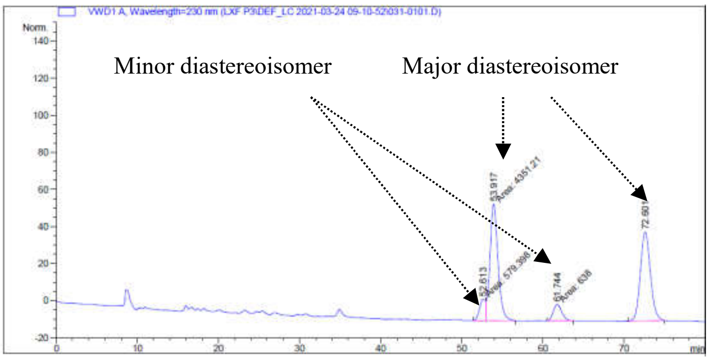

Peak #	RetTime [min]	Type	Width [min]	Area [mAU*s]	Height [mAU]	Area %
1	52.613	MM	0.8412	579.39758	11.47897	5.9763
2	53.917	MM	1.1525	4351.20801	62.92669	44.8815
3	61.744	MM	1.1886	637.99957	8.94625	6.5808
4	72.601	BB	1.2056	4126.26660	47.91573	42.5613

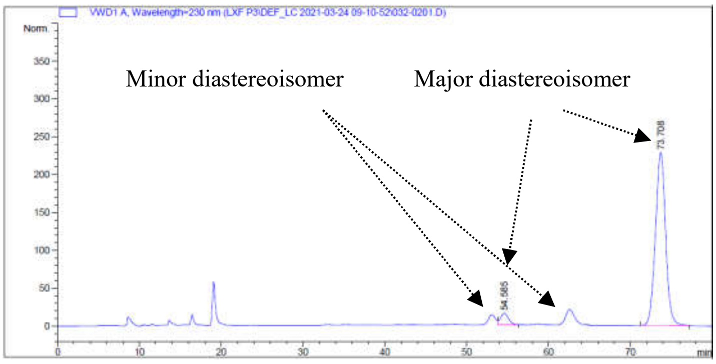

Peak #	RetTime [min]	Type	Width [min]	Area [mAU*s]	Height [mAU]	Area %
1	54.585	VB	0.9003	1038.36707	15.37053	4.9936
2	73.708	BB	1.1877	1.97555e4	228.51031	95.0064

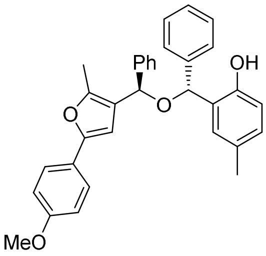

3n

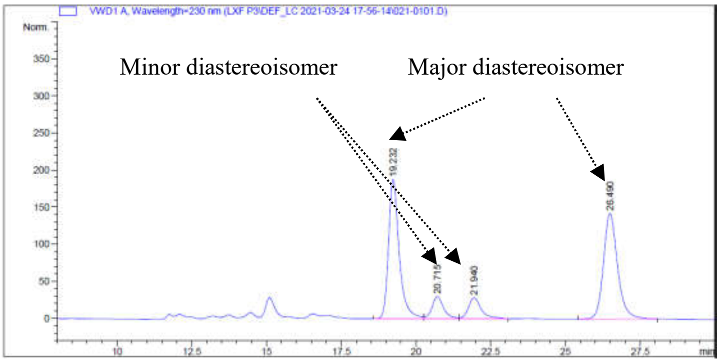

Peak #	RetTime [min]	Type	Width [min]	Area [mAU*s]	Height [mAU]	Area %
1	19.232	BV	0.3723	4657.16992	187.81303	42.3152
2	20.715	VV	0.4078	814.39325	29.92729	7.3996
3	21.940	VB	0.4399	840.39056	28.27720	7.6358
4	26.490	BB	0.5030	4693.94092	142.76804	42.6493

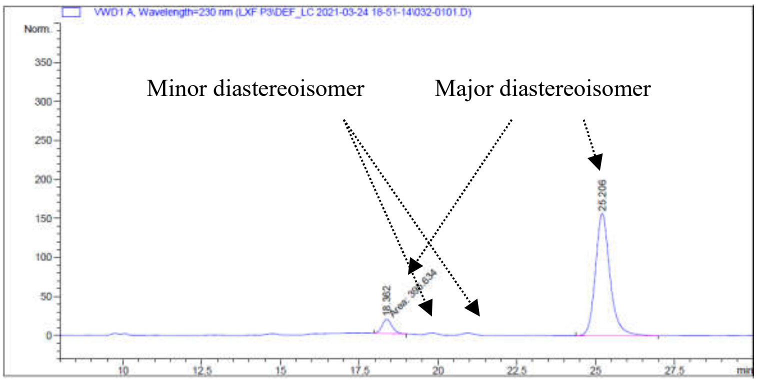

Peak #	RetTime [min]	Type	Width [min]	Area [mAU*s]	Height [mAU]	Area %
1	18.362	MM	0.3636	395.63394	18.13679	7.2591
2	25.206	BB	0.4873	5054.56982	156.58621	92.7409

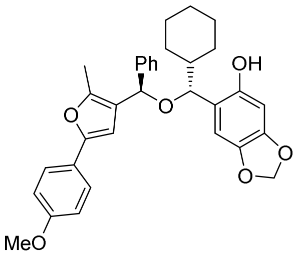

3o

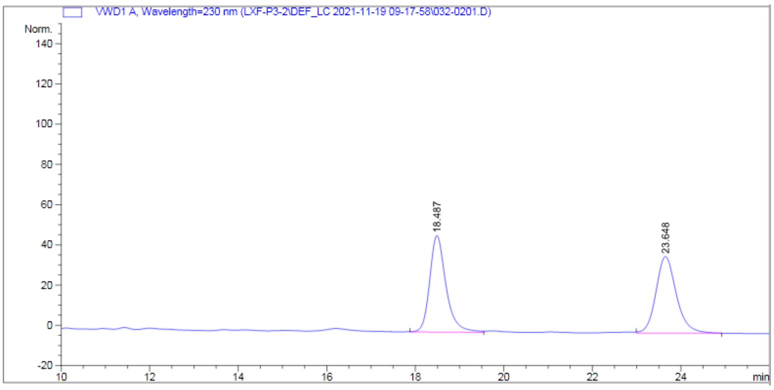

Peak #	RetTime [min]	Type	Width [min]	Area [mAU*s]	Height [mAU]	Area %
1	18.487	BB	0.3870	1225.60083	47.97268	50.2234
2	23.648	BB	0.4876	1214.69897	38.04829	49.7766

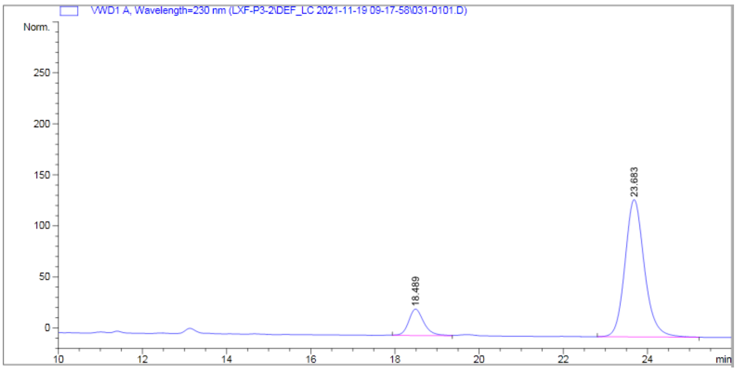

Peak #	RetTime [min]	Type	Width [min]	Area [mAU*s]	Height [mAU]	Area %
1	18.489	BB	0.3799	646.74011	25.80358	13.0017
2	23.683	BB	0.4905	4327.52295	134.52953	86.9983

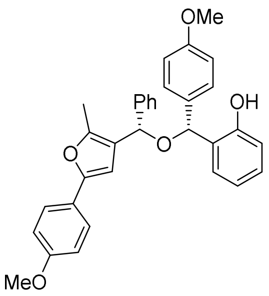

4a

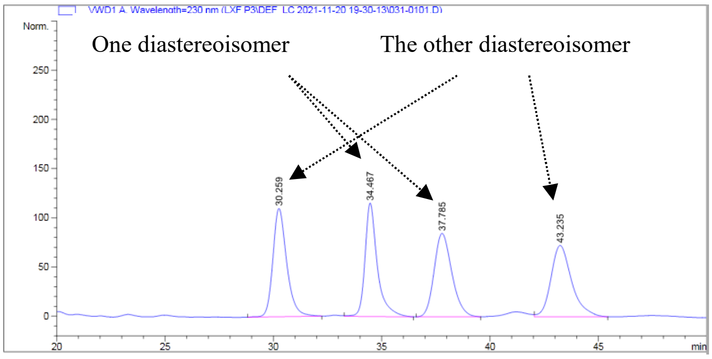

Peak #	RetTime [min]	Type	Width [min]	Area [mAU*s]	Height [mAU]	Area %
1	30.259	BB	0.6687	4894.01318	110.11251	25.8722
2	34.467	VB	0.5810	4572.96875	115.24876	24.1750
3	37.785	MM	0.9238	4618.13135	83.31624	24.4137
4	43.235	VB	1.0038	4831.02197	72.45552	25.5392

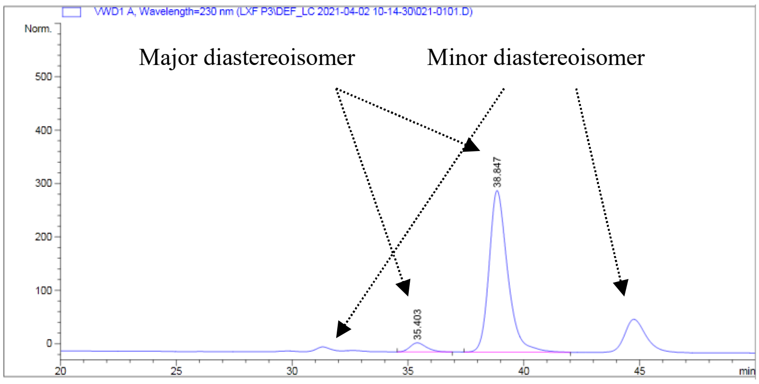

Peak #	RetTime [min]	Type	Width [min]	Area [mAU*s]	Height [mAU]	Area %
1	35.403	BB	0.7340	852.37390	17.07090	4.7916
2	38.847	BB	0.8363	1.69367e4	302.68491	95.2084

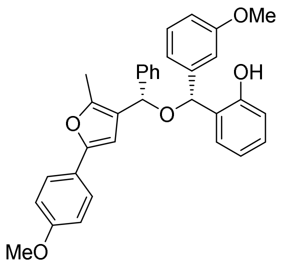

4b

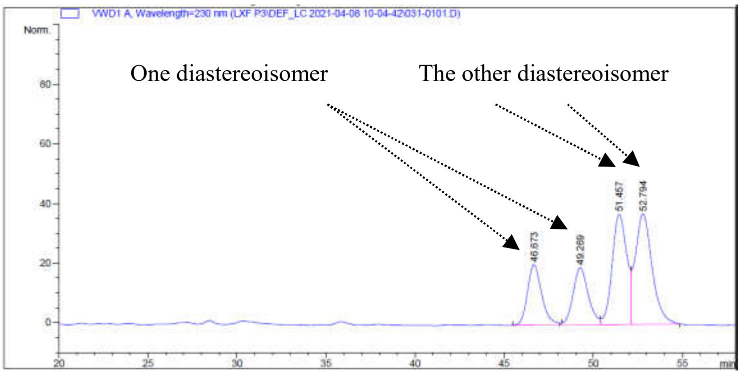

Peak #	RetTime [min]	Type	Width [min]	Area [mAU*s]	Height [mAU]	Area %
1	46.673	BB	0.8198	1130.35498	20.35012	17.4702
2	49.269	BV	0.8769	1137.06238	19.21162	17.5739
3	51.457	MM	0.9631	2124.37012	36.76338	32.8333
4	52.796	MM	0.9769	2078.38501	35.46057	32.1226

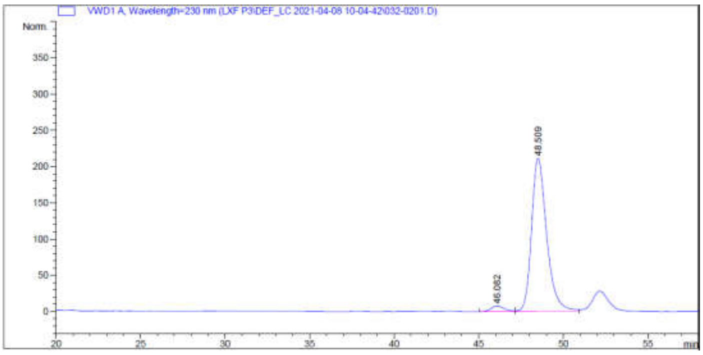

Peak #	RetTime [min]	Type	Width [min]	Area [mAU*s]	Height [mAU]	Area %
1	46.082	BV	0.7879	446.57117	7.86193	3.2740
2	48.509	VB	0.9493	1.31933e4	211.42802	96.7260

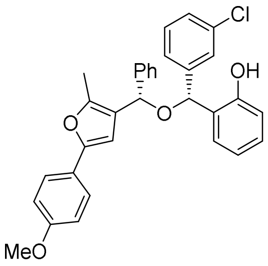

4c

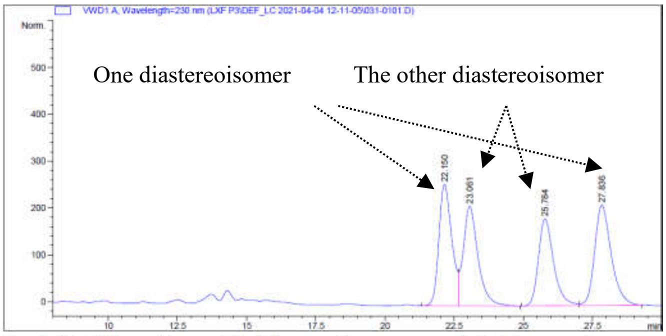

Peak #	RetTime [min]	Type	Width [min]	Area [mAU*s]	Height [mAU]	Area %
1	22.150	VV	0.4938	8420.00586	259.33408	26.7408
2	23.061	MM	0.5744	7064.07813	204.97186	22.4346
3	25.784	BV	0.5785	7097.08154	185.64586	22.5394
4	27.836	VB	0.6255	8906.31738	213.90396	28.2853

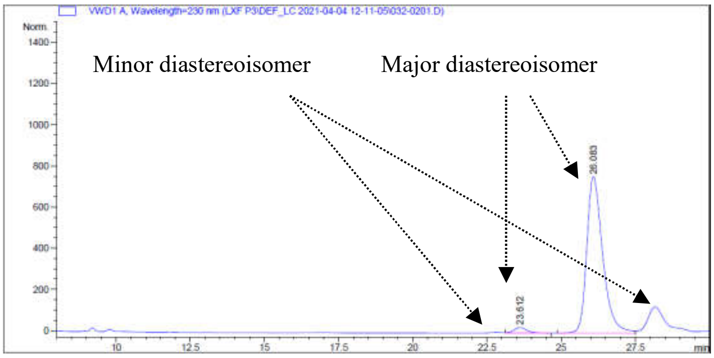

Peak #	RetTime [min]	Type	Width [min]	Area [mAU*s]	Height [mAU]	Area %
1	23.612	VB	0.4679	842.51147	27.07570	2.8841
2	26.083	BV	0.5668	2.83694e4	759.57214	97.1159

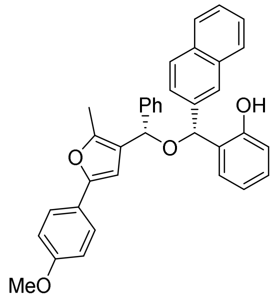

4d

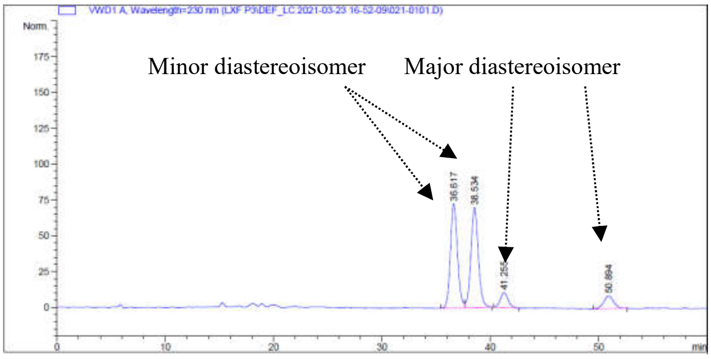

Peak #	RetTime [min]	Type	Width [min]	Area [mAU*s]	Height [mAU]	Area %
1	36.617	BV	0.7100	3278.62378	72.63994	42.7057
2	38.534	VB	0.7114	3315.06445	69.79155	43.1804
3	41.255	BB	0.7511	525.23096	10.47210	6.8414
4	50.894	BB	0.8586	558.32422	8.67523	7.2725

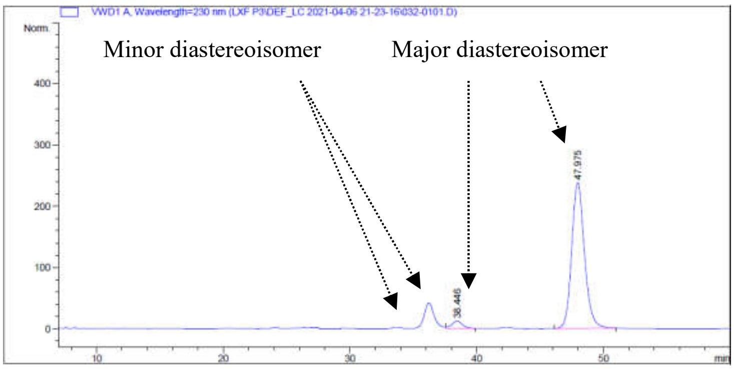

Peak #	RetTime [min]	Type	Width [min]	Area [mAU*s]	Height [mAU]	Area %
1	38.446	VB	0.8458	702.30627	11.87534	4.0360
2	47.975	BB	1.0638	1.66988e4	237.81503	95.9640

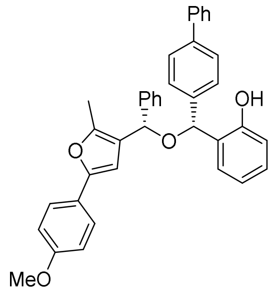

4e

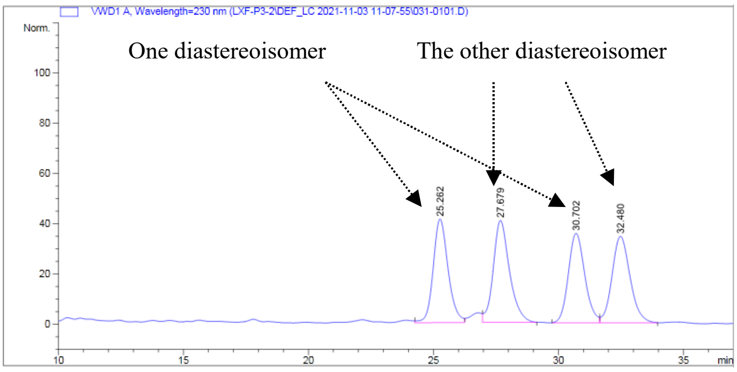

Peak #	RetTime [min]	Type	Width [min]	Area [mAU*s]	Height [mAU]	Area %
1	25.262	VV	0.6325	1703.56641	41.20281	24.7358
2	27.679	VB	0.6775	1835.28198	40.60452	26.6483
3	30.702	BV	0.7012	1626.03369	35.55472	23.6100
4	32.480	VB	0.7652	1722.16833	34.37715	25.0059

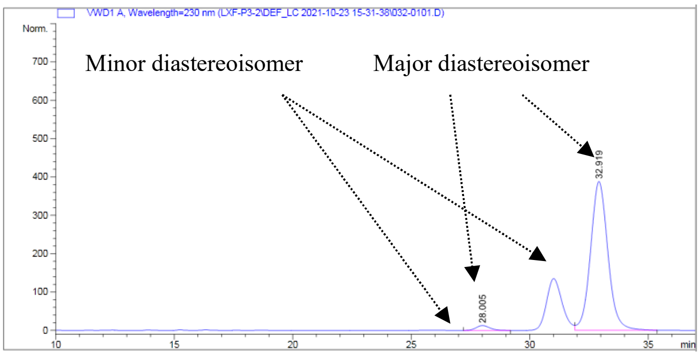

Peak #	RetTime [min]	Type	Width [min]	Area [mAU*s]	Height [mAU]	Area %
1	28.005	BB	0.6161	519.29626	12.76237	2.5546
2	32.919	VB	0.7949	1.98082e4	388.28714	97.4454

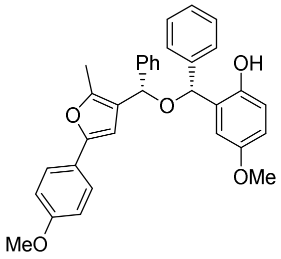

4f

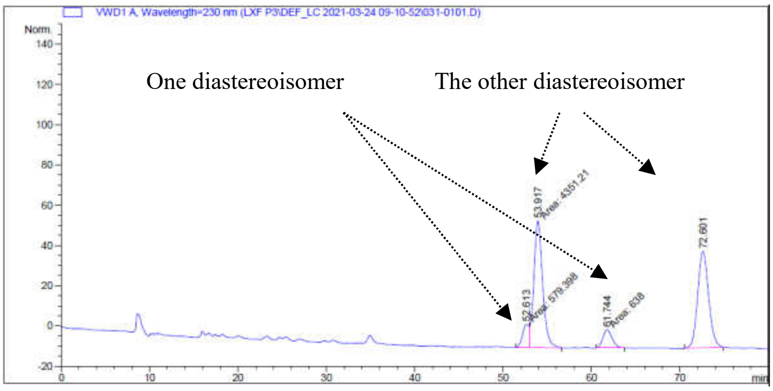

Peak #	RetTime [min]	Type	Width [min]	Area [mAU*s]	Height [mAU]	Area %
1	52.613	MM	0.8412	579.39758	11.47897	5.9763
2	53.917	MM	1.1525	4351.20801	62.92669	44.8815
3	61.744	MM	1.1886	637.99957	8.94625	6.5808
4	72.601	BB	1.2056	4126.26660	47.91573	42.5613

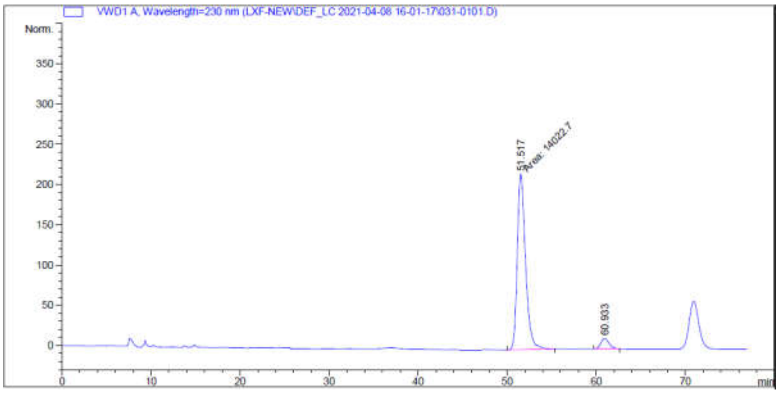

Peak #	RetTime [min]	Type	Width [min]	Area [mAU*s]	Height [mAU]	Area %
1	51.517	MM	1.0716	1.40227e4	218.09651	94.2666
2	60.933	BB	0.9341	852.87836	13.15393	5.7334

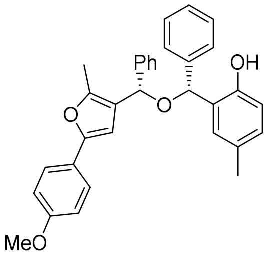

4g

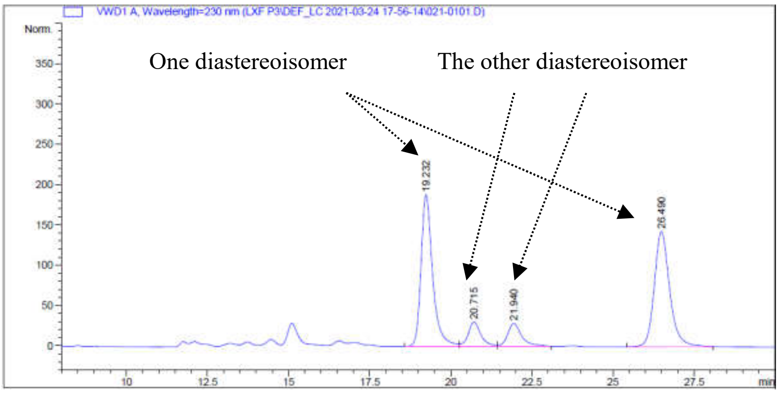

Peak #	RetTime [min]	Type	Width [min]	Area [mAU*s]	Height [mAU]	Area %
1	19.232	BV	0.3723	4657.16992	187.81303	42.3152
2	20.715	VV	0.4078	814.39325	29.92729	7.3996
3	21.940	VB	0.4399	840.39056	28.27720	7.6358
4	26.490	BB	0.5030	4693.94092	142.76804	42.6493

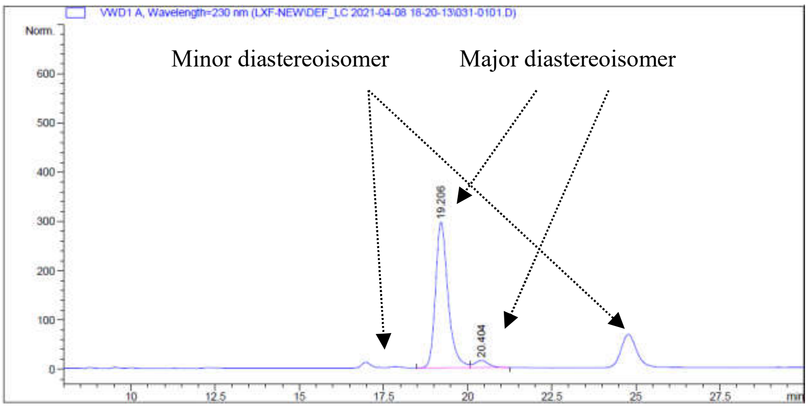

Peak #	RetTime [min]	Type	Width [min]	Area [mAU*s]	Height [mAU]	Area %
1	19.206	BV	0.3938	7665.63477	296.03210	94.4593
2	20.404	VB	0.4411	449.64206	15.07683	5.5407

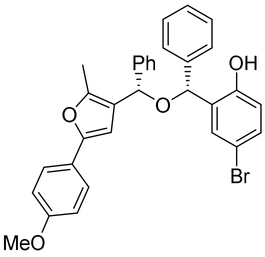

4h

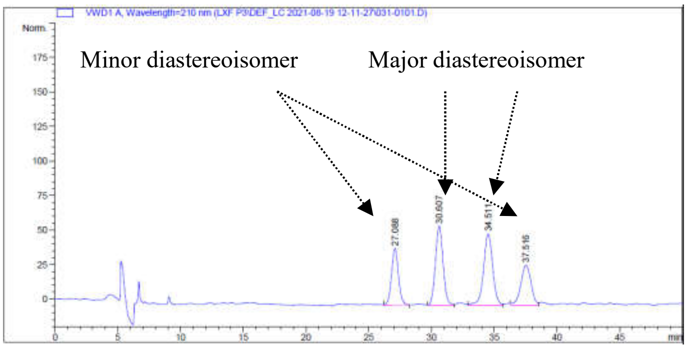

Peak #	RetTime [min]	Type	Width [min]	Area [mAU*s]	Height [mAU]	Area %
1	27.088	BV	0.5806	1573.62476	41.11745	19.4236
2	30.607	VB	0.6418	2420.53833	57.26717	29.8772
3	34.511	MM	0.8107	2471.35449	50.80802	30.5044
4	37.516	VV	0.8432	1636.10803	29.00706	20.1948

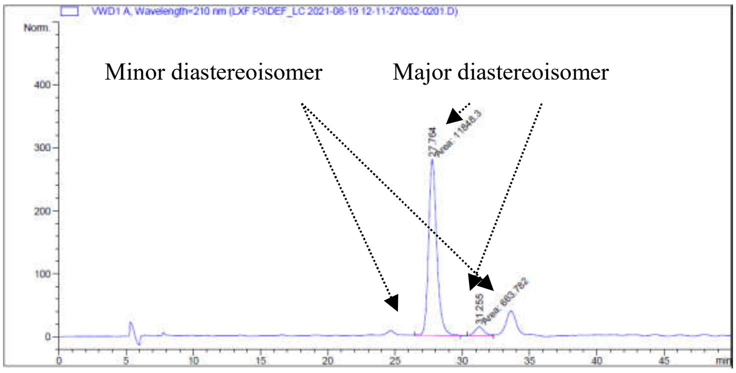

Peak #	RetTime [min]	Type	Width [min]	Area [mAU*s]	Height [mAU]	Area %
1	27.764	MM	0.7060	1.18483e4	279.70602	94.6949
2	31.255	MM	0.7945	663.78162	13.92378	5.3051

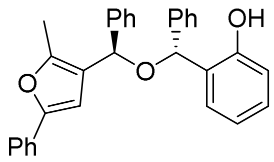

3p

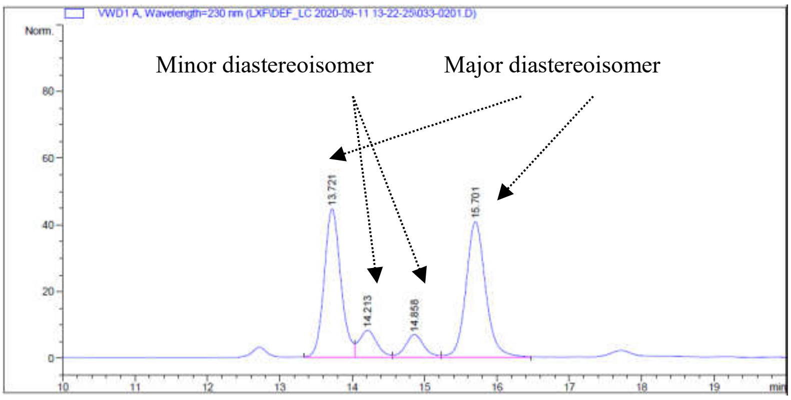

Peak #	RetTime [min]	Type	Width [min]	Area mAU *s	Height [mAU]	Area %
1	13.721	BV	0.2423	705.14490	44.33951	42.5654
2	14.213	VV	0.2544	134.73853	7.95613	8.1334
3	14.858	VV	0.2653	116.05917	6.68061	7.0058
4	15.701	MM	0.2960	700.67194	39.45028	42.2954

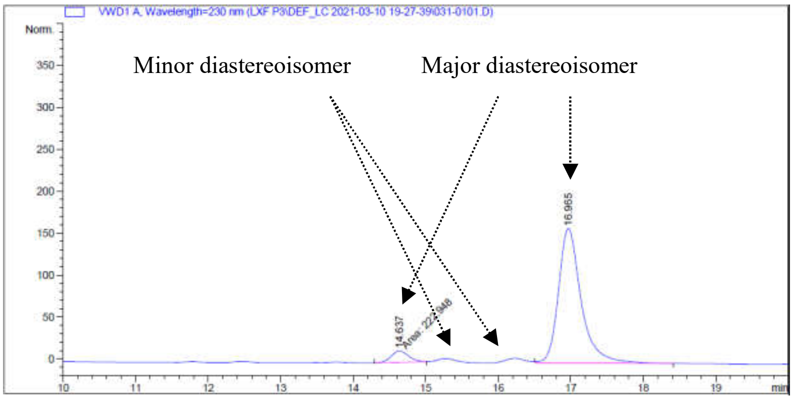

Peak #	RetTime [min]	Type	Width [min]	Area [mAU*s]	Height [mAU]	Area %
1	14.637	MM	0.2760	222.94827	13.46339	5.9794
2	16.965	VB	0.3275	3505.64307	160.59985	94.0206

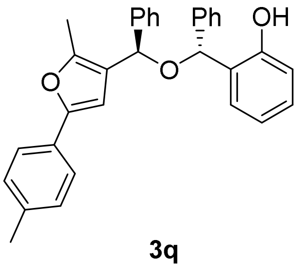

3q

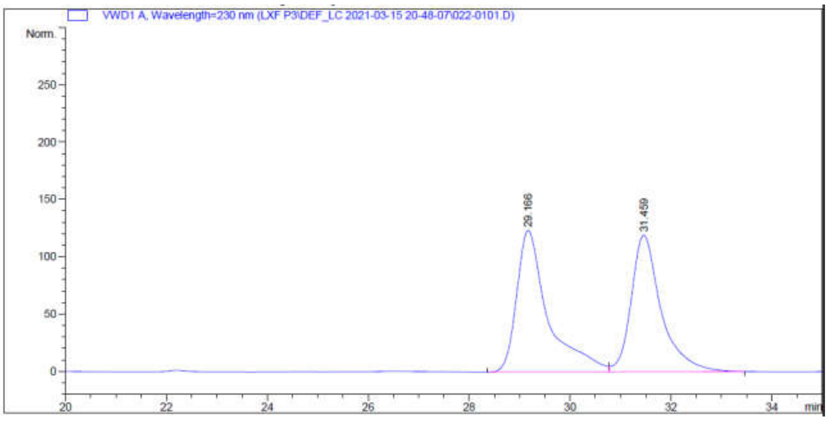

Peak #	RetTime [min]	Type	Width [min]	Area [mAU*s]	Height [mAU]	Area %
1	29.166	BV	0.6103	5322.76514	123.56953	51.9926
2	31.459	VB	0.6061	4914.77441	119.22501	48.0074

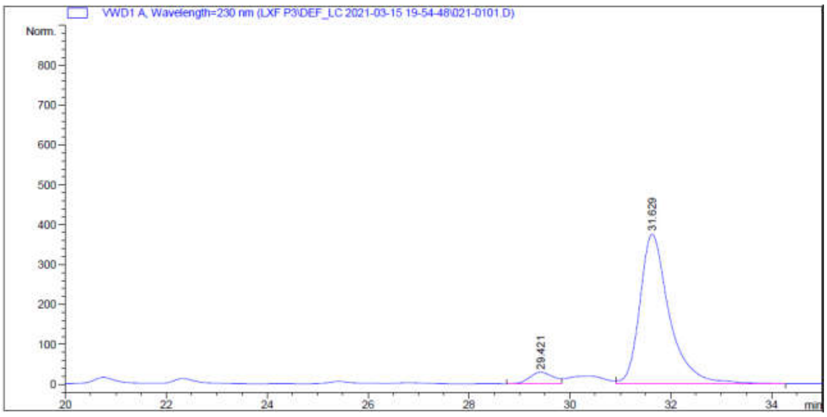

Peak #	RetTime [min]	Type	Width [min]	Area [mAU*s]	Height [mAU]	Area %
1	29.421	BV	0.4903	949.81134	29.07791	5.8682
2	31.629	VB	0.6077	1.52360e4	375.19360	94.1318

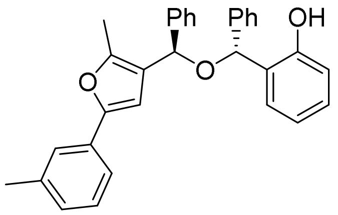

3r

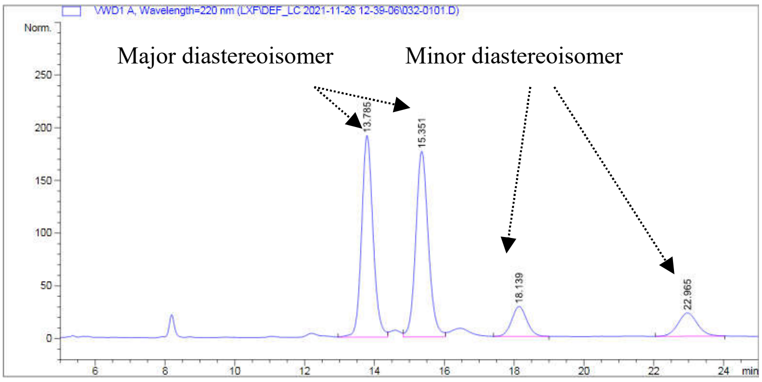

Peak #	RetTime [min]	Type	Width [min]	Area mAU *s	Height [mAU]	Area %
1	13.785	BV	0.3479	4339.67334	191.14394	41.4578
2	15.351	VV	0.3820	4402.83057	176.05757	42.0612
3	18.139	VB	0.4713	868.49268	28.43646	8.2969
4	22.965	BB	0.5918	856.68353	22.11147	8.1841

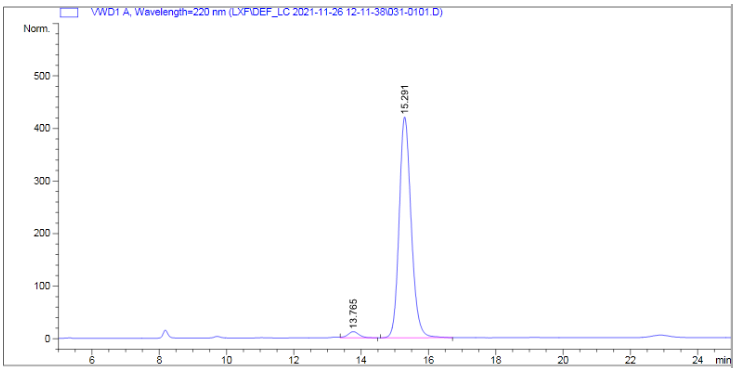

Peak #	RetTime [min]	Type	Width [min]	Area mAU *s	Height [mAU]	Area %
1	13.765	MM	0.4442	356.95877	13.39315	3.3100
2	15.291	BB	0.3814	1.04272e4	419.94357	96.6900

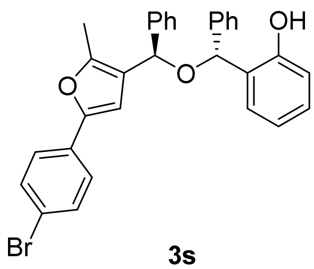

3s

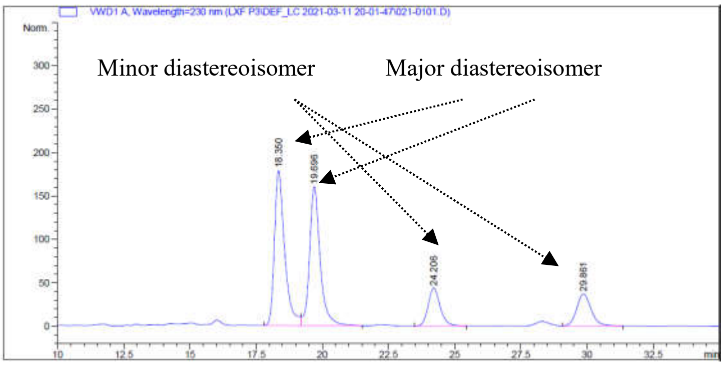

Peak #	RetTime [min]	Type	Width [min]	Area [mAU*s]	Height [mAU]	Area %
1	18.350	BV	0.3967	4804.60449	178.70738	38.8754
2	19.696	MM	0.4831	4758.07715	164.13400	38.4989
3	24.206	BB	0.4835	1386.11279	43.88640	11.2154
4	29.861	VB	0.5865	1410.19275	36.95341	11.4103

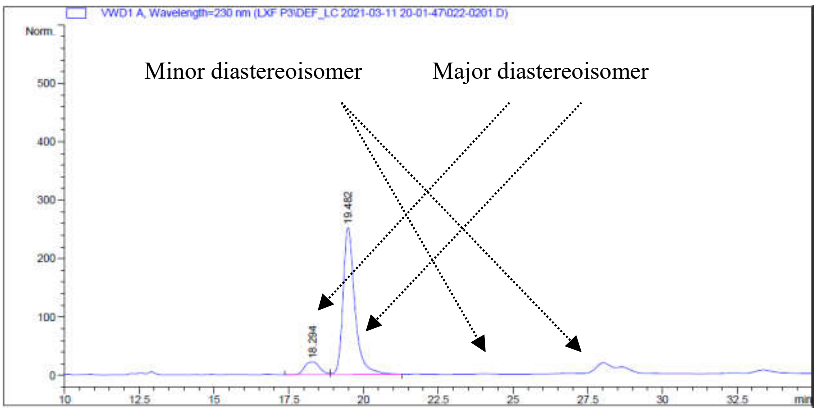

Peak #	RetTime [min]	Type	Width [min]	Area [mAU*s]	Height [mAU]	Area %
1	18.294	VV	0.5602	754.25140	21.60242	9.8737
2	19.482	VB	0.4097	6884.71143	251.45168	90.1263

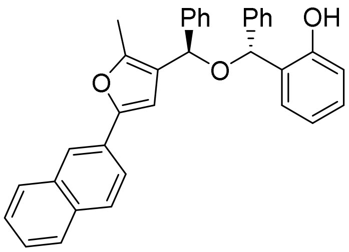

3t

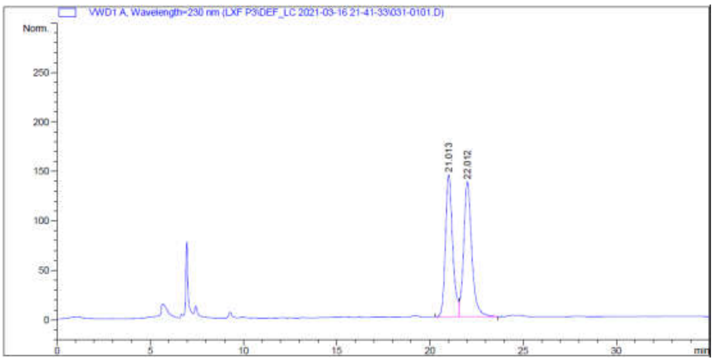

Peak #	RetTime [min]	Type	Width [min]	Area [mAU*s]	Height [mAU]	Area %
1	21.013	BV	0.4061	3839.21631	143.80278	48.1080
2	22.012	VB	0.4543	4141.19092	136.52982	51.8920

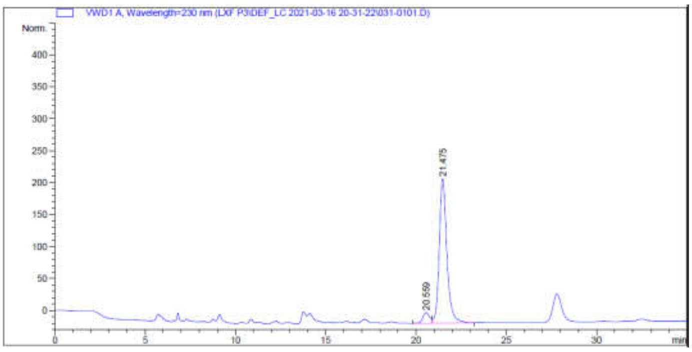

Peak #	RetTime [min]	Type	Width [min]	Area [mAU*s]	Height [mAU]	Area %
1	20.559	VV	0.3959	432.12772	16.49345	6.1296
2	21.475	VB	0.4406	6617.68799	226.02393	93.8704

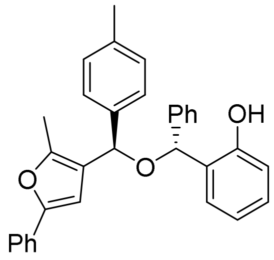

3u

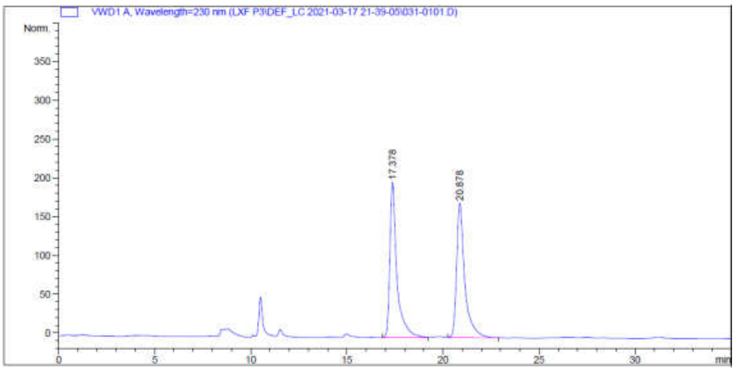

Peak #	RetTime [min]	Type	Width [min]	Area [mAU*s]	Height [mAU]	Area %
1	17.378	VB	0.3719	5138.00586	200.39360	50.0470
2	20.878	VB	0.4308	5128.35010	173.45680	49.9530

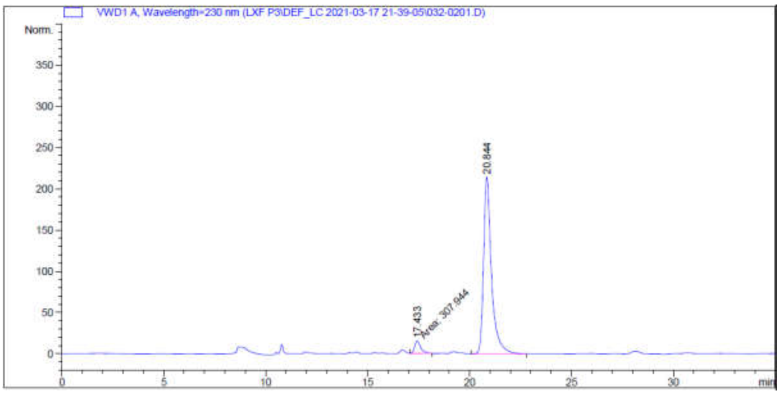

Peak #	RetTime [min]	Type	Width [min]	Area [mAU*s]	Height [mAU]	Area %
1	17.433	MM	0.3401	307.94409	15.09304	5.0155
2	20.844	BB	0.4029	5831.88965	214.67029	94.9845

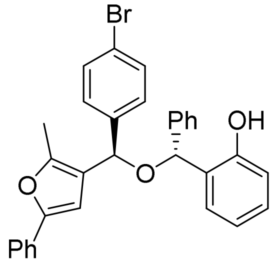

3v

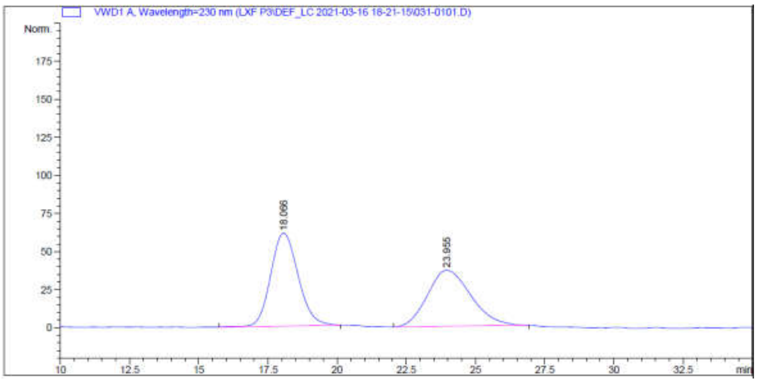

Peak #	RetTime [min]	Type	Width [min]	Area [mAU*s]	Height [mAU]	Area %
1	18.066	BB	1.0277	4216.25098	61.00327	51.3306
2	23.955	BB	1.4832	3997.66309	36.70660	48.6694

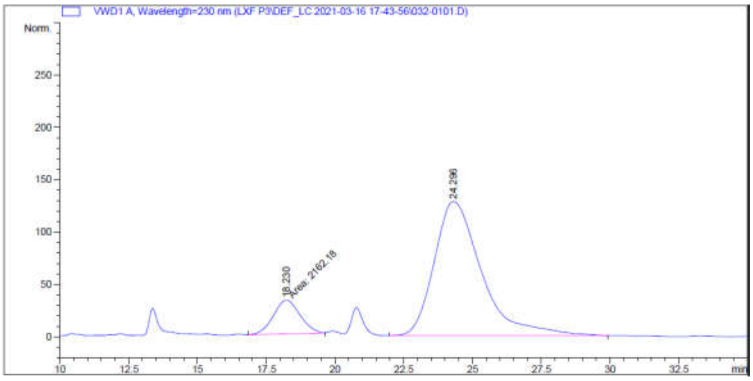

Peak #	RetTime [min]	Type	Width [min]	Area [mAU*s]	Height [mAU]	Area %
1	18.230	MM	1.1217	2162.18164	32.12528	12.3569
2	24.296	BB	1.7727	1.53356e4	127.93541	87.6431

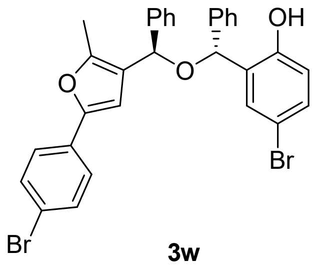

3w

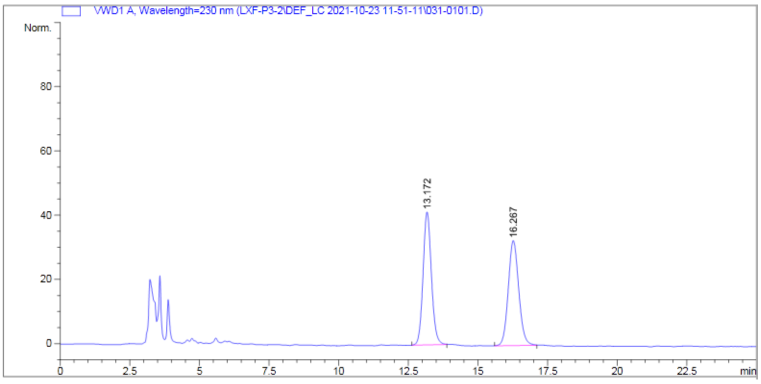

Peak #	RetTime [min]	Type	Width [min]	Area [mAU*s]	Height [mAU]	Area %
1	13.172	BB	0.3211	863.81793	41.32323	50.3066
2	16.267	BB	0.4040	853.28955	32.65511	49.6934

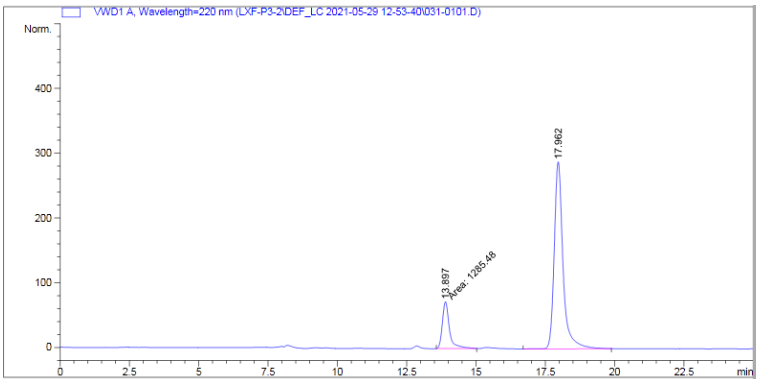

Peak #	RetTime [min]	Type	Width [min]	Area [mAU*s]	Height [mAU]	Area %
1	13.897	MM	0.2962	1285.48352	72.34298	16.7377
2	17.962	BB	0.3327	6394.69824	288.71954	83.2623

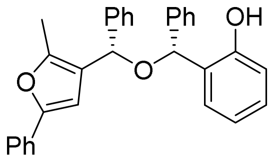

4j

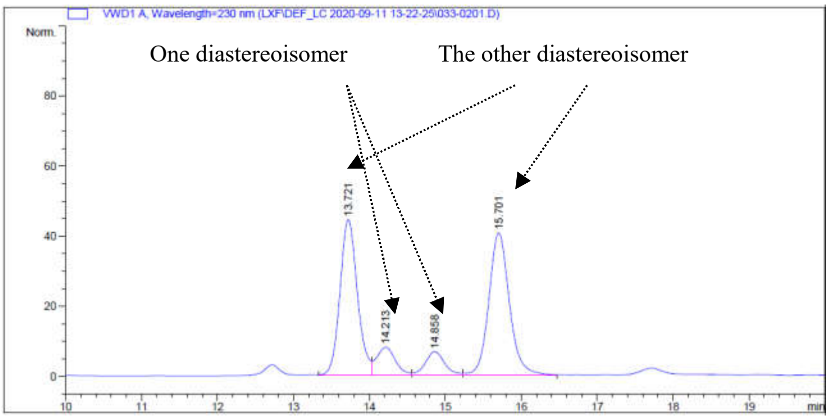

Peak #	RetTime [min]	Type	Width [min]	Area mAU *s	Height [mAU]	Area %
1	13.721	BV	0.2423	705.14490	44.33951	42.5654
2	14.213	VV	0.2544	134.73853	7.95613	8.1334
3	14.858	VV	0.2653	116.05917	6.68061	7.0058
4	15.701	MM	0.2960	700.67194	39.45028	42.2954

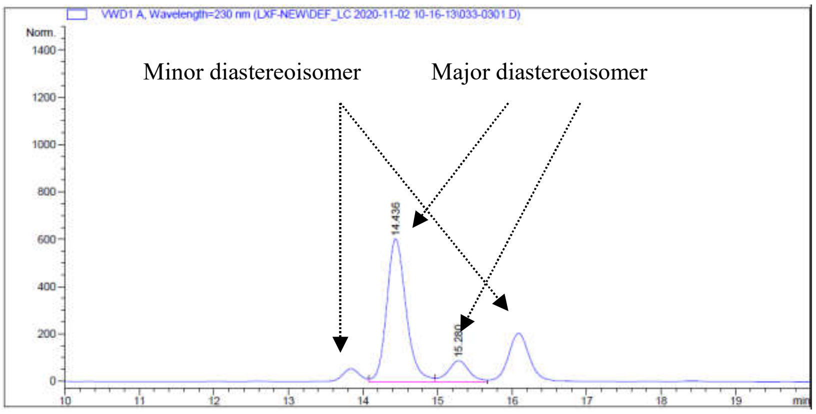

Peak #	RetTime [min]	Type	Width [min]	Area [mAU*s]	Height [mAU]	Area %
1	14.436	VV	0.2779	1.09217e4	604.18451	86.2064
2	15.280	VV	0.2926	1747.53809	89.22388	13.7936

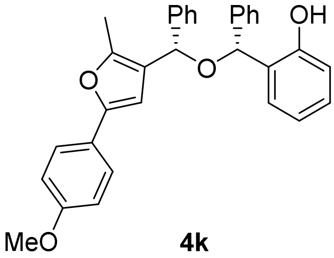

4k

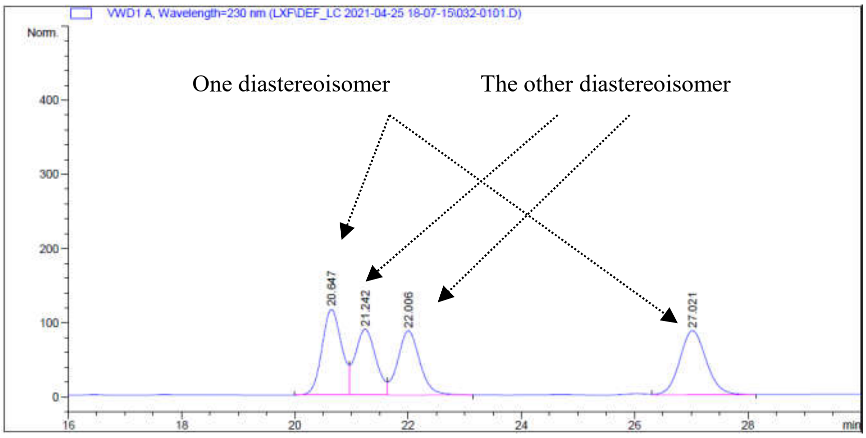

Peak #	RetTime [min]	Type	Width [min]	Area mAU *s	Height [mAU]	Area %
1	20.647	BV	0.3691	2791.61084	115.60535	27.3154
2	21.242	VV	0.3851	2270.55347	88.95551	22.2170
3	22.006	VB	0.4127	2341.90771	86.69201	22.9151
4	27.021	VB	0.5012	2815.84180	86.38092	27.5525

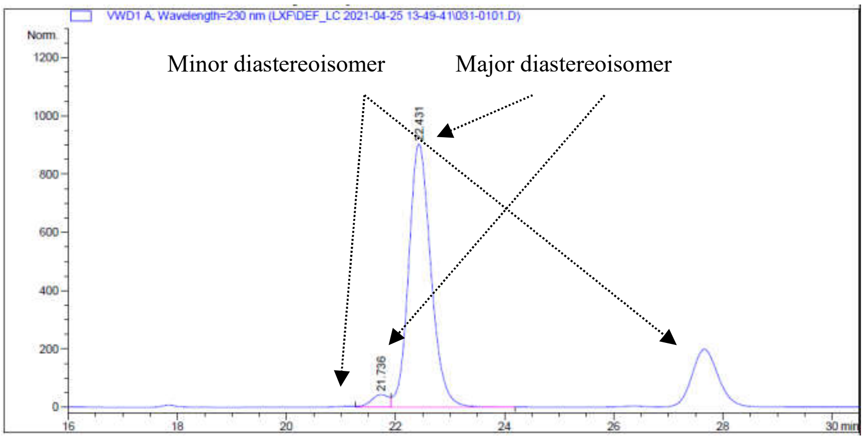

Peak #	RetTime [min]	Type	Width [min]	Area mAU *s	Height [mAU]	Area %
1	21.736	VV	0.3383	968.85681	43.53631	3.6458
2	22.431	VB	0.4356	2.56056e4	903.24915	96.3542

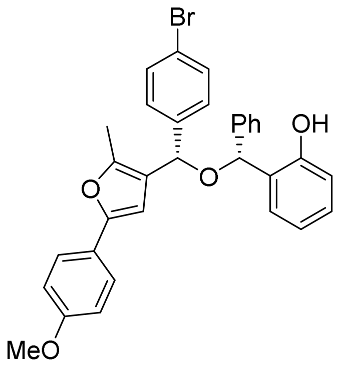

4l

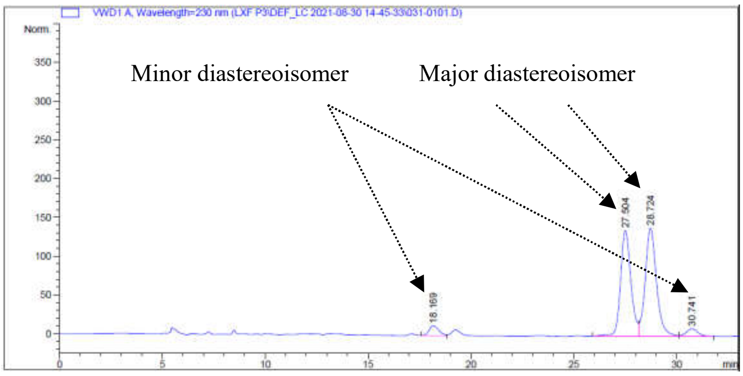

Peak #	RetTime [min]	Type	Width [min]	Area [mAU*s]	Height [mAU]	Area %
1	18.169	VV	0.5132	441.88669	12.99849	4.1993
2	27.504	BV	0.5252	4765.68848	136.56836	45.2889
3	28.724	MM	0.6020	4934.87891	136.62082	46.8967
4	30.741	BB	0.5935	380.41647	9.59996	3.6151

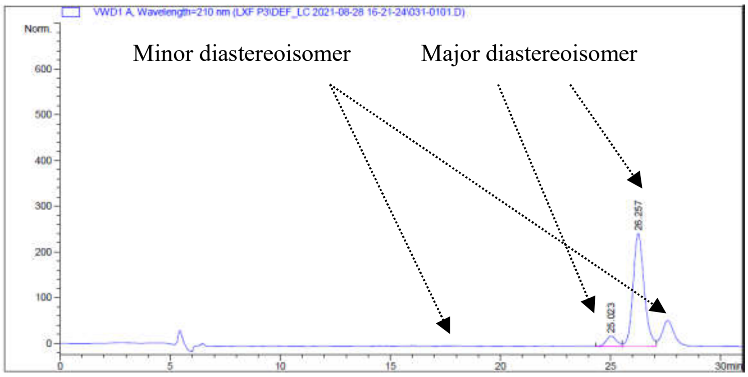

Peak #	RetTime [min]	Type	Width [min]	Area [mAU*s]	Height [mAU]	Area %
1	25.023	BV	0.4909	737.92627	22.65132	7.8376
2	26.257	VV	0.5328	8677.32715	246.69221	92.1624

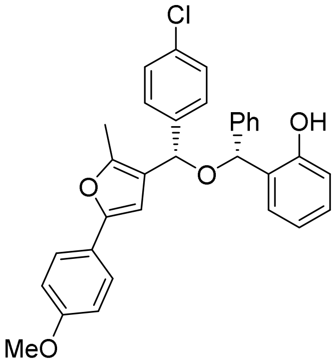

4m

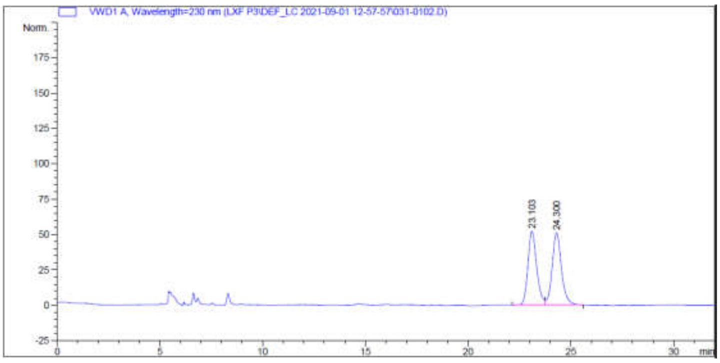

Peak #	RetTime [min]	Type	Width [min]	Area [mAU*s]	Height [mAU]	Area %
1	23.103	BV	0.4557	1558.00928	52.25458	49.9597
2	24.300	MM	0.5135	1560.52026	50.65052	50.0403

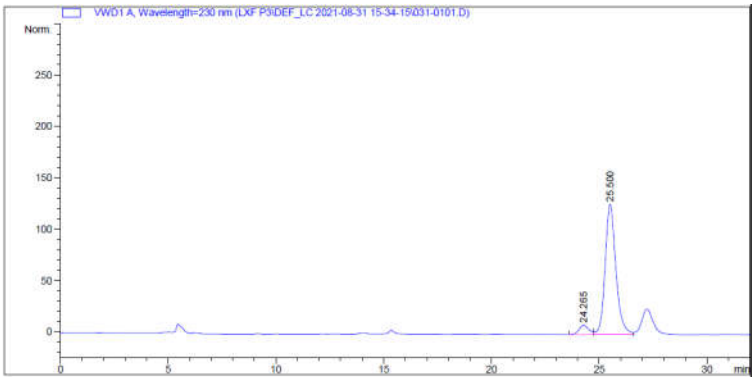

Peak #	RetTime [min]	Type	Width [min]	Area [mAU*s]	Height [mAU]	Area %
1	24.265	BV	0.4763	281.09241	8.93535	6.0367
2	25.500	VV	0.5204	4375.29346	126.42367	93.9633

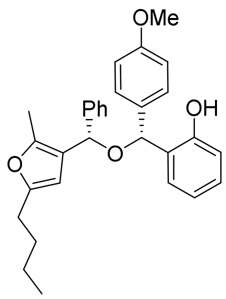

4n

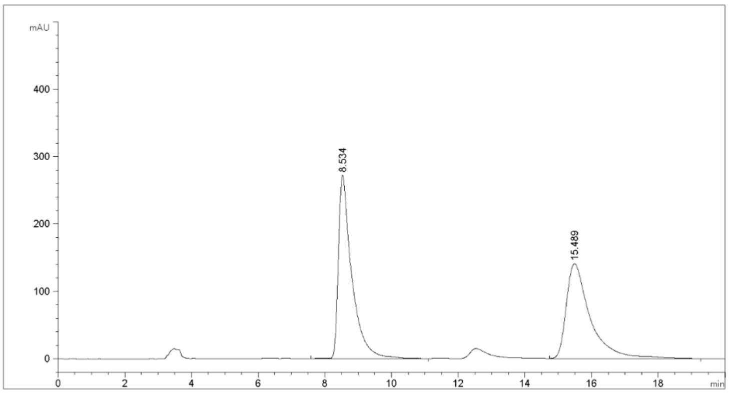

Peak #	RetTime [min]	Type	Width [min]	Area [mAU*s]	Height [mAU]	Area %
1	8.534	BB	0.3975	7566.15186	271.93198	52.0755
2	15.489	BB	0.7205	6963.03369	140.62779	47.9245

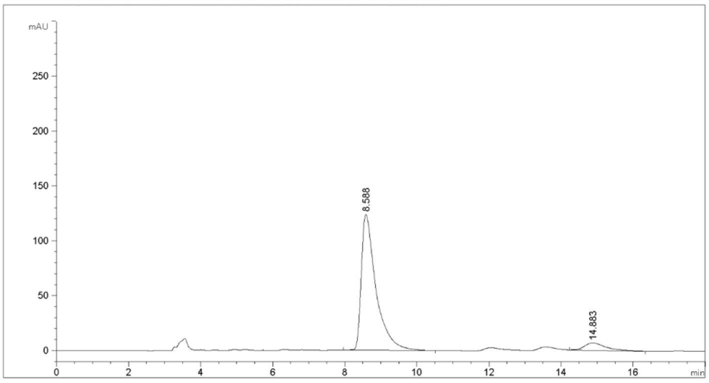

Peak #	RetTime [min]	Type	Width [min]	Area [mAU*s]	Height [mAU]	Area %
1	8.588	BB	0.4208	3505.85132	123.77887	92.7655
2	14.883	BB	0.6098	273.41000	6.70583	7.2345

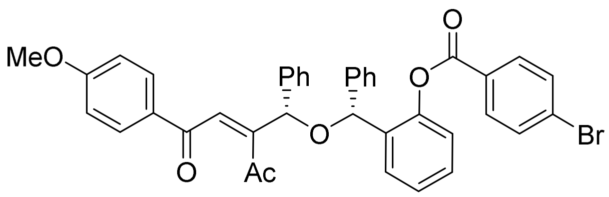

6a

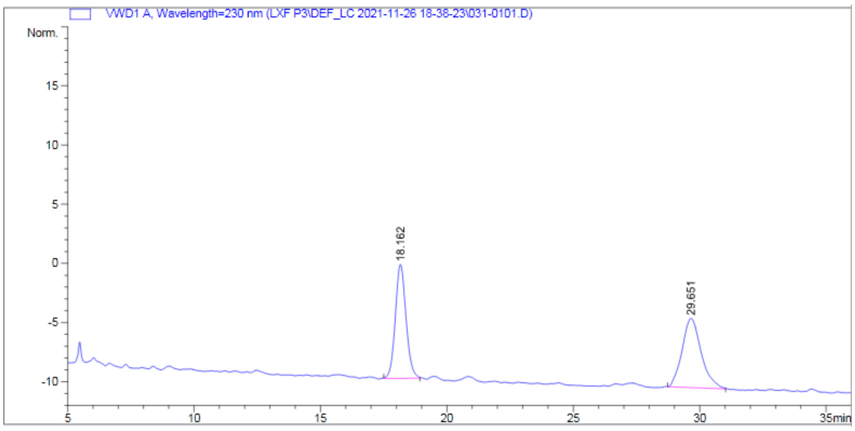

Peak #	RetTime [min]	Type	Width [min]	Area [mAU*s]	Height [mAU]	Area %
1	18.162	MM	0.5095	297.88489	9.74491	49.1651
2	29.651	BB	0.7534	308.00220	5.84330	50.8349

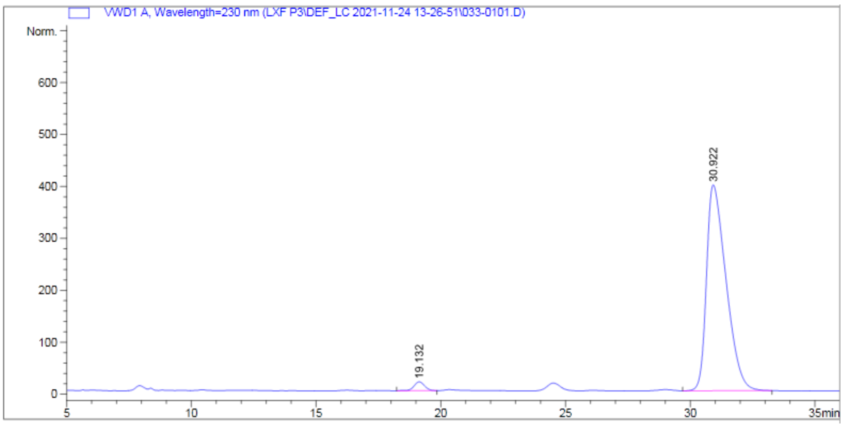

Peak #	RetTime [min]	Type	Width [min]	Area [mAU*s]	Height [mAU]	Area %
1	19.132	MM	0.6308	769.10107	20.32237	3.4389
2	30.922	VB	0.8179	2.15956e4	396.32980	96.5611

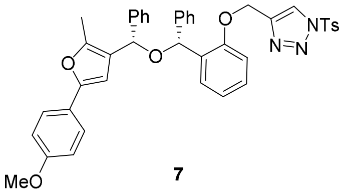

7

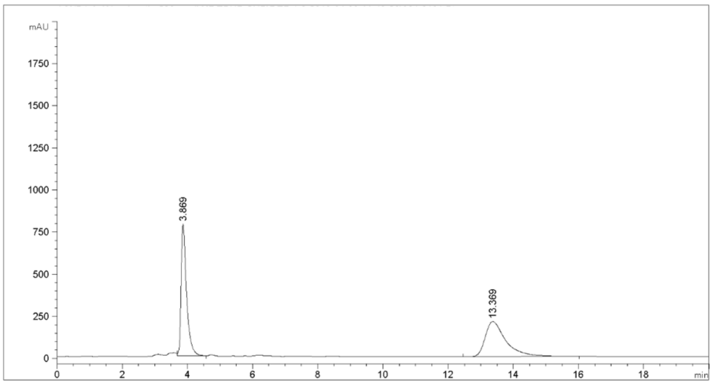

Peak #	RetTime [min]	Type	Width [min]	Area [mAU*s]	Height [mAU]	Area %
1	3.869	VB	0.1719	9057.01953	780.08643	49.6118
2	13.369	BB	0.6558	9198.75098	208.02707	50.3882

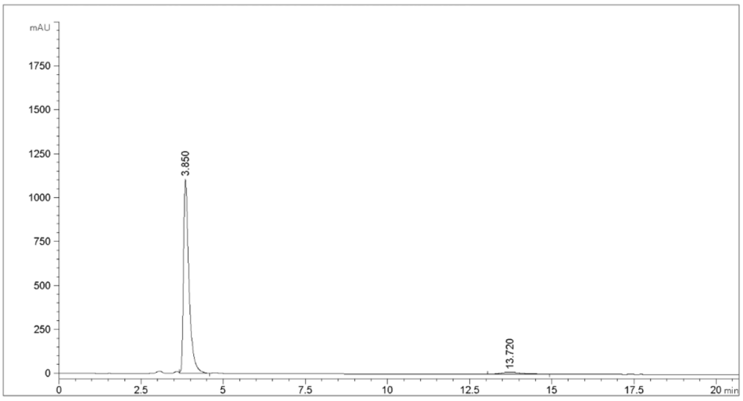

Peak #	RetTime [min]	Type	Width [min]	Area [mAU*s]	Height [mAU]	Area %
1	3.850	VB	0.1691	1.27010e4	1104.96533	96.2666
2	13.720	BB	0.6548	492.56866	11.25781	3.7334

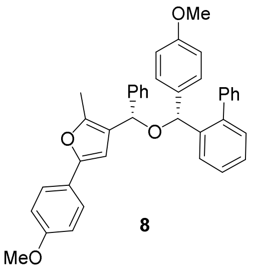

8

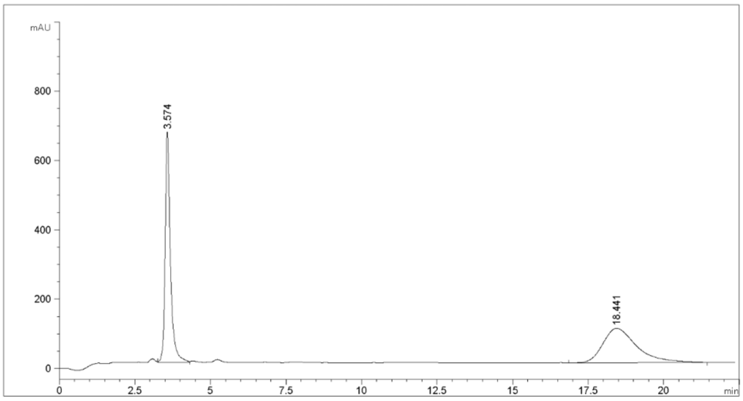

Peak #	RetTime [min]	Type	Width [min]	Area [mAU*s]	Height [mAU]	Area %
1	3.574	VV	0.1758	8020.77734	664.47339	51.0405
2	18.441	BB	1.1675	7693.74414	99.33416	48.9595

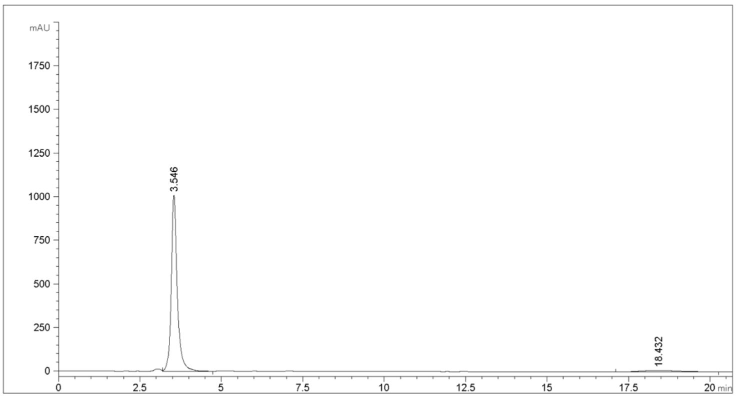

Peak #	RetTime [min]	Type	Width [min]	Area [mAU*s]	Height [mAU]	Area %
1	3.546	VV	0.1849	1.29810e4	1010.38245	95.0587
2	18.432	BB	0.9625	674.77045	9.10299	4.9413

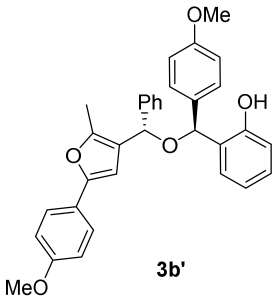

3b'

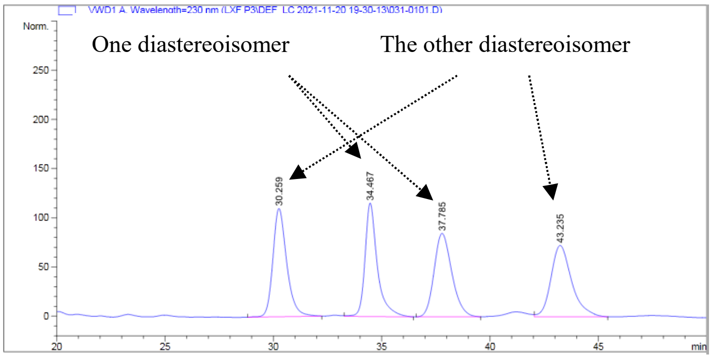

Peak #	RetTime [min]	Type	Width [min]	Area [mAU*s]	Height [mAU]	Area %
1	30.259	BB	0.6687	4894.01318	110.11251	25.8722
2	34.467	VB	0.5810	4572.96875	115.24876	24.1750
3	37.785	MM	0.9238	4618.13135	83.31624	24.4137
4	43.235	VB	1.0038	4831.02197	72.45552	25.5392

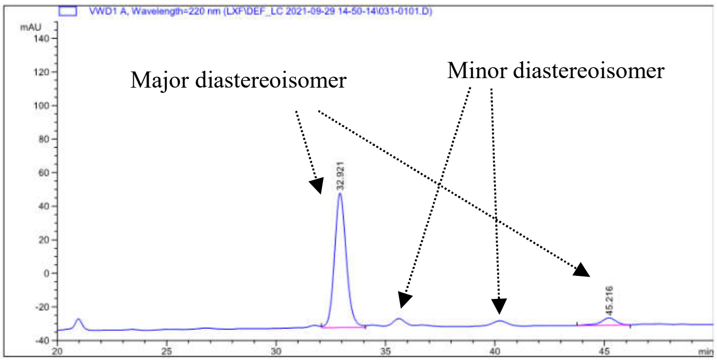

Peak #	RetTime [min]	Type	Width [min]	Area mAU *s	Height [mAU]	Area %
1	32.921	VB	0.5752	2990.44385	80.12359	93.2356
2	45.216	BV	0.7250	216.96097	4.28280	6.7644

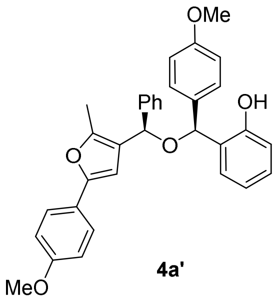

4a'

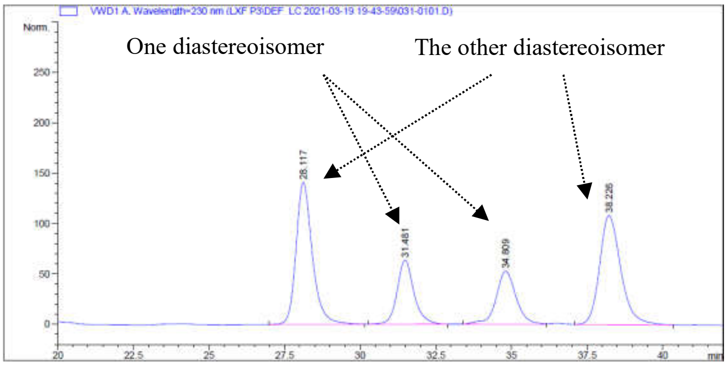

Peak #	RetTime [min]	Type	Width [min]	Area [mAU*s]	Height [mAU]	Area %
1	28.117	BB	0.5673	5314.15625	141.17642	34.2086
2	31.481	BB	0.5845	2477.57007	63.74636	15.9488
3	34.809	BB	0.7018	2439.03223	52.81876	15.7007
4	38.226	VB	0.7475	5303.78223	108.57338	34.1419

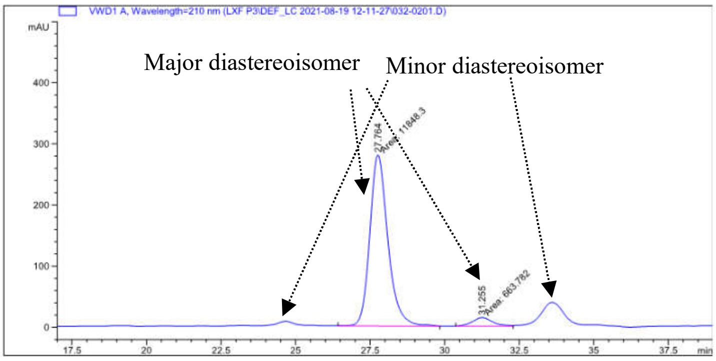

Peak #	RetTime [min]	Type	Width [min]	Area [mAU* s]	Height [mAU]	Area %
1	27.764	MM	0.7060	1.18483e4	279.70602	94.6949
2	31.255	MM	0.7945	663.78162	13.92378	5.3051

Supplementary Fig. 4 HPLC spectra

10. References

(1) Lin, X. et al. Biomimetic approach to the catalytic enantioselective synthesis of tetracyclic isochroman. *Nat. Commun.* **12**, 4958–4967 (2021).

(2) Hsiao, C. C., Liao, H. H. & Rueping, M. Enantio- and diastereoselective access to distant stereocenters embedded within tetrahydroxanthenes: utilizing *ortho*-quinone methides as reactive intermediates in asymmetric Brønsted acid catalysis. *Angew. Chem. Int. Ed.* **53**, 13258–13263 (2014).

(3) Wang, L. et al. Diastereodivergent synthesis of chromeno[2,3-b]chromenes by tuning all of the reactivity centers of isocyanoacetate. *Chem. Commun.* **58**, 6433–6436 (2022).

(4) Sun, M. et al. Catalytic Asymmetric (4+3) Cyclizations of In Situ Generated ortho-Quinone Methides with 2-Indolylmethanols. *Angew. Chem. Int. Ed.* **58**, 8703–8708 (2019).

(5) Zheng, Y., Clarkson, G. J. & Wills, M. Asymmetric transfer hydrogenation of *o*-hydroxyphenyl ketones: utilizing directing effects that optimize the asymmetric synthesis of challenging alcohols. *Org. Lett.* **22**, 3717–3721 (2020).

(6) Liu, F., Qian, D., Li, L., Zhao, X. & Zhang, J. Diastereo- and enantioselective gold(I)-catalyzed intermolecular tandem cyclization/[3+3]cycloadditions of 2-(1-alkynyl)-2-alken-1-ones with nitrones. *Angew. Chem. Int. Ed.* **48**, 5505–5508 (2019).

(7) Tanpure, S. D., Kuo, T. -C., Cheng, M. -J. & Liu, R. -S. Gold(I)-catalyzed highly diastereo- and enantioselective constructions of bicyclo[3.2.1]oct-6-ene frameworks via (4 + 3)-cycloadditions. *ACS Catal.* **12**, 536–543 (2022).

(8) Zhang, M. & Zhang, J. Gold(I)-catalyzed cyclization of 2-(1-alkynyl)-alk-2-en-1-one oximes: a facile access to highly substituted N-alkoxypyrroles. **48**, 6399–6401 (2012).

(9) Hatano, M., Moriyama, K., Maki, T. & Ishihara K. Which is the actual catalyst: chiral phosphoric acid or chiral calcium phosphate? *Angew. Chem. Int. Ed.* **49**, 3823–3826 (2010).

(10) Zhdanko, A. & Maier, M. E. Synthesis of gem-diaurated species from alkynols. *Chem. Eur. J.* **19**, 3932–3942 (2013).

(11) Frisch, M. J. et al. Gaussian 16 Rev. C.01, Wallingford, CT, 2016.

(12) Raghavachari, K. Perspective on "Density functional thermochemistry. III. The role of exact exchange" - Becke AD (1993) J Chem Phys 98:5648-52. *Theor. Chem. Acc.* **103**, 361–363 (2000).

(13) Hehre, W. J., Ditchfield, R. & Pople, J. A. Self-consistent molecular orbital methods. XII. Further extensions of Gaussian-type basis sets for use in molecular orbital studies of organic molecules. *J. Chem. Phys.* **56**, 2257–2261 (1972).

(14) Francl, M. M. et al. Self-consistent molecular orbital methods. XXIII. A polarization-type basis set for second-row elements. *J. Chem. Phys.* **77**, 3654–3665 (1982).

(15) Scalmani, G. & Frisch, M. J. Continuous surface charge polarizable continuum models of solvation. I. General formalism. *J. Chem. Phys.* **132**, 114110 (2010).

(16) McCann, D. M., Stephens, P. J. & Cheeseman, J. R. Determination of absolute configuration using density functional theory calculation of optical rotation: Chiral Alkanes. *J. Org. Chem.* **69**, 8709–8717 (2004).
